# Impact of resistance training intensity on body composition and nutritional intake among college women with overweight and obesity: a cluster randomized controlled trial

**DOI:** 10.3389/fpubh.2025.1589036

**Published:** 2025-05-30

**Authors:** Qiang Wang, Wen Sheng Xiao, Mahmoud Danaee, Soh Kim Geok, Wan Ying Gan, Wang Li Zhu, Yi Qiang Mai

**Affiliations:** ^1^Department of Sport Studies, Faculty of Educational Studies, Universiti Putra Malaysia, Serdang, Malaysia; ^2^Faculty of Physical Education, Yichun Early Childhood Teacher College, Yichun, China; ^3^School of Physical Education, Huzhou University, Huzhou, China; ^4^Department of Social and Preventive Medicine, Faculty of Medicine, Universiti of Malaya, Kuala Lumpur, Malaysia; ^5^Department of Nutrition, Faculty of Medicine and Health Sciences, Universiti Putra Malaysia, Serdang, Malaysia; ^6^Institute of Education Development, Nanchang University, Nanchang, China; ^7^School of Physical Education and Taijiquan, Henan Polytechnic University, Jiaozuo, China

**Keywords:** overweight, obesity, college women, nutritional intake, body composition, resistance training, intensity

## Abstract

**Introduction:**

The prevalence of overweight and obesity among college women is a public health concern. This study examined the effects of different intensities of resistance training on body composition and nutritional intake in overweight and obese college women.

**Methods:**

A cluster-randomized controlled trial with a 12-week intervention included 72 participants, divided into low, moderate, and high-intensity resistance training groups, along with a control group. The 3-day food record and other standardized instruments measured the corresponding variables.

**Results:**

Post-test results showed a significant reduction in body fat percentage for the high-intensity group compared with the low-intensity (*p* = 0.035) and control groups (*p* = 0.026). Significant reductions in energy and protein intake for the moderate-intensity group compared to the low-intensity (both *p* < 0.022) and control groups (both *p* < 0.007). In the high-intensity group, energy intake was significantly reduced compared to the control group (*p* < 0.001). Fat intake decreased in the moderate-intensity group compared to the high-intensity (*p* = 0.017) and control groups (*p* = 0.002). Carbohydrate intake was significantly lower in the moderate-intensity group compared to the control group (*p* = 0.001), while in the high-intensity group compared with the low-intensity (*p* = 0.049) and control groups (p < 0.001). The correlation between changes in body composition and nutritional intake was positive in the high-intensity group (r = 0.513–0.839, all *p* < 0.05) but negative in the control group (r = −0.606–−0.838, all *p* < 0.01).

**Discussion:**

These findings suggest that high-intensity resistance training is most effective for improving body composition; both moderate- (especially) and high-intensity resistance training are the most influential in modifying nutritional intake; and high-intensity resistance training demonstrates the best correlation between changes in body composition and nutritional intake. Further research is required to address the contradictory result regarding body fat percentage compared to before, as well as to explore limitations related to population diversity, alternative exercise comparisons, rigorous dietary assessment methods, and underlying mechanisms.

**Systematic review registration:**

ClinicalTrials.gov, NCT05530629.

## 1 Introduction

The prevalence of obesity has increased dramatically worldwide, with over 2 billion adults overweight and 700 million obese by 2017—a threefold rise since 1980 (1). Overweight and obesity contribute to over 4 million deaths annually and are linked to more than 20 chronic diseases, including diabetes and cardiovascular conditions (2). Furthermore, its psychological impact—such as increased anxiety and depression—further exacerbates the global burden (3). The primary type of overweight and obesity constitutes the majority, accounting for over 95% of the total cases (4). This form of overweight and obesity stems mainly from modifiable lifestyle factors, such as high-calorie diets, sedentary behavior, and lack of regular physical activity (5). These behaviors disrupt energy balance, underscoring the importance of effective, sustainable weight-loss strategies.

Management approaches for obesity include diet modification, pharmacological treatments, surgeries, and exercise (6). While drugs and surgeries can deliver significant weight loss, such approaches are often associated with severe side effects and high costs (7). Dieting, though non-invasive, is difficult to maintain and may lead to risks like anorexia (8). Exercise-based strategies, including high-intensity interval training (HIIT), aerobic training (AT), and resistance training (RT), have become increasingly favored for their safety and efficacy (9). HIIT yields substantial fat reduction but suffers from poor adherence rates due to its high-intensity cardiorespiratory load (10). AT is widely practiced but often results in weight regain post-intervention due to slowed metabolism and increased appetite (11).

According to Obesity and The First Law of Thermodynamics, RT aids weight loss and can be explained by the change of energy intake and energy expenditure (12). RT facilitates significant calorie burn during sessions and sustains elevated metabolism afterward due to muscle hypertrophy. While similar to RT, some other training, such as high-intensity functional training, can also generate analogous calorie-burning outcomes (13). However, the researchers found that RT can also reduce appetite, even better than other types of exercise. One article reports short-term RT intervention was associated with reduced reported energy intake among young women [Barros (14)]. Another study has also pointed out that both full-and split-body RT can reduce the diet intake of untrained men after 8 weeks (15). A meta-analysis study by Panissa et al. (16) found that the absolute amount of energy consumed after acute RT was different from that after other types of exercise.

Although RT has a remarkable effect on fat loss, there are too many types of explanations for the definition of fat (17). Reasonably selecting fat indicators is essential for achieving a complete understanding of the impact of RT on fat loss. As an authority in the health field, one World Health Organization consultation report on obesity, Preventing and Managing the Global Epidemic, recommends using BMI, waist circumference, and body fat percentage to assess overweight and obesity (18). However, many relevant studies in the field of RT plus overweight and obesity only used body composition as a demographic variable (19, 20). The studies that include a change in body composition usually only involve one or two variables as secondary objectives in the above field (21, 22). There are only a limited number of existing articles that address all three fat-level variables that RT affects among overweight and obese individuals (23). So, in order to get a better picture of how RT affects body composition as a whole, researchers need to look into all three variables that are related to fat levels and how RT affects these variables among the high-weight population.

As previously described, RT is more critical than other forms of exercise in terms of changes in energy intake for weight loss. According to the Dietary Balance Model, the reason for the change in energy intake can be explained by changes in macronutrient intake (24). Studies indicate that RT not only affects total calorie intake but also alters the macronutrient intake. A study by Kim and Kim (25) demonstrated a 12-week low-intensity RT program led to a marginally significant reduction in protein intake among inactive men with obesity. Baer et al. (26) reported a marginally significant reduction in fat intake among burn rats following 14 days of daily insulin and RT. Additionally, according to Halliday et al. (27), 9 months of RT can significantly reduce the fat intake among overweight and obese adults.

The appropriate intensity for reducing body composition and energy intake is essential for RT. Regarding body composition, Wewege et al. (28) demonstrated that high-intensity RT effectively reduces body fat percentage and waist circumference compared to lower-intensity RT. Meanwhile, as explored in the research by Sahin et al. (29), low-intensity RT might yield less dramatic changes in body mass index (BMI). While still beneficial, the low intensity might not trigger the same degree of fat metabolism as higher-intensity workouts. Shiotsu and Yanagita (30) also said that moderate-intensity RT can better improve waist-to-hip ratio than low-intensity RT because it increases oxygen consumption after exercise, which indirectly leads to a faster metabolism of fat.

However, a systematic review shows that current research in this field mostly involves only one or two intensities. There is limited research on the effects of three different intensities of RT on body composition among overweight and obese individuals (23). Comparing the three different intensity effects by just different articles with one or two intensities cannot prove that the differences aren’t caused by differences in duration, population, or something else besides intensity. It also cannot scientifically measure the difference between the effects of three intensities. Therefore, it is necessary to compare the effects of three different intensities of RT on body composition among overweight and obese individuals.

Regarding nutritional intake, research showed different RT intensities affect many physiological and biochemical indicators reflecting energy intake among overweight and obese individuals (31). The result of one study by Liu et al. (32) showed that high-intensity RT has greater beneficial effects than low-to-moderate intensity RT in the attenuation of insulin in patients with Type 2 Diabetes, which suggests improved glucose metabolism and a potential reduction in energy intake. A recent study by Liu et al. (33) found that after acute moderate-intensity RT, the recovery of the autonomic nervous system to appetite suppression is slower compared to low-intensity RT among young adult males, which may result in less energy intake. A study by Fan et al. (34) demonstrated that the effects of high-intensity RT compared to medium-and low-intensity RT on HOMA-IR in patients with Type 2 Diabetes Mellitus were distinct, with only medium-and low-intensity RT resulting in a significant decrease in HOMA-IR, which suggests enhanced insulin sensitivity and lowered systemic inflammation, leading to lower energy intake.

However, a recent systematic review reported that most current relevant studies focus solely on the effect of a single intensity of RT on nutritional intake among overweight and obese individuals (23, 25). Only one study examined two RT intensities, but it failed to report the exact intensity levels (27). Research comparing the effects of three different RT intensities in this area is limited. Comparing the effects of three intensities through separate studies, each involving only one or two intensities, cannot rule out the possibility that observed differences are due to factors such as duration, population, or other variables beyond intensity. Furthermore, it does not allow for a scientific quantification of the differences in effects. Therefore, it is essential to investigate the comparative effects of three RT intensities on nutritional intake in overweight and obese populations.

The above analysis considered overweight and obese populations broadly, yet it is important to account for the differences in status and intervention outcomes across various groups, particularly in overweight and obese female college students (35, 36). Specifically, college students have not been spared from this epidemic. Research shows that the proportion of overweight and obese students aged 18–24 rose by 13% over the past two decades, reaching 31% globally (37). For example, U.S. college students gain an average of 2 kg (4.4 pounds) during their first year, with studies indicating that over 60% report reduced physical activity and 45% increase their calorie intake from processed foods (38). The combined effect significantly elevates their risk of long-term obesity.

Female college students face a disproportionate burden. Jiang et al. (39) observed that the prevalence of overweight and obesity among female Chinese college students with low (6.3%) and moderate (10.6%) per capita household income is higher than that of male counterparts (5.5 and 7.3%), respectively. Similarly, Farrag et al. (40) reported that truncal obesity among Egyptian university students was significantly more prevalent in females (51.7%) than in males (38.8%). These findings highlight the need for age-and gender-specific interventions to address this growing issue. However, a systematic review by Qiang et al. (23), identified a gap in research on females and youth in the field of RT’s impact on nutritional intake in overweight and obese populations. Therefore, examining the effects of varying RT intensities on body composition and nutritional intake in overweight and obese college women is essential for addressing this critical health issue.

## 2 Methods

### 2.1 Participants and design

This study employed a cluster randomized controlled trial (CRCT) design to minimize contamination effects between groups during the training intervention. Four groups were included: high-intensity RT (HI), moderate-intensity RT (MI), low-intensity RT (LI), and control groups (CG). Four campuses in Yichun, Jiangxi, China, were randomly assigned to these groups: the old campus (HI) and new campus (MI) of Yichun College, and the new campus (LI) and Gaoan campus (CG) of Yichun Early Childhood Teacher College. Recruitment, interventions, and measurements were conducted simultaneously in fitness centers on each campus.

In August 2022, 122 overweight or obese female college students aged 18–22 with a BMI > 25 were recruited through campus leaflets and recruitment meetings. Initial group sizes were n_(HI)_ = 32, n_(MI)_ = 30, n_(LI)_ = 27, and n_(CG)_ = 33. Eligible participants were healthy, had no regular and active exercise routines in the previous 6 months, and had not followed restrictive diets within 3 months. Students who had sports disorders, eating disorders, or irregular menstrual cycles were also excluded (18, 41). Following the screening process, 102 participants were deemed eligible, with n_(HI)_ = 27, n_(MI)_ = 24, n_(LI)_ = 21, and n_(CG)_ = 30.

Sample size was calculated using G*Power based on previous studies, with the lowest reported effect size (*f* = 0.24) for energy intake by (169). Sample size calculations were performed using repeated measures ANOVA (within-between interaction), considering a Type I error (*α* = 0.05) and power (1−β) = 0.80 for two time measurements and four groups. The Critical *F* value (2.80) was greater than the effect size F, which suggests statistical significance (42, 43). The G*Power calculation is shown in Supplementary Figure 1. The effective sample size for the CRCT design was calculated to be 14.56 per group, based on dividing 58.24 by 4 (44), as shown in Supplementary Figure 2. After adjusting for a 20% dropout rate (45, 46), the final sample size was set at 72 participants (18 per group), randomly selected using a computer-generated sequence.

All participants provided written informed consent and received detailed instructions regarding the intervention and measurement procedures, particularly on estimating food portion sizes and classification, from trained campus coaches and assistants before the intervention. Coaches and assistants were trained to supervise participants throughout the intervention by ensuring adherence to the prescribed intensity levels (low, moderate, high), monitoring exercise technique, providing corrective feedback, and maintaining safety protocols. The expectation was to create a motivating environment, ensuring participants remained engaged throughout the sessions. To maintain consistency across groups, all coaches and assistants followed a standardized protocol, with supervision regularly monitored by the research team. This approach minimized variability in supervision. Baseline data were collected 2 weeks before the intervention, and post-test data were collected 2 days after its termination. Participants were instructed to maintain their usual lifestyle, avoiding additional training or dietary changes during the study. The researchers maintained regular contact with the coaches to monitor any physical or situational changes of the participants. The trial would be discontinued for any participant who experienced unforeseen circumstances that impeded continued participation or exhibited menstrual irregularities. Ethical approval was obtained from Universiti Putra Malaysia (JKEUPM-2022-483) and Yichun College (LSK No. 2022017).

### 2.2 Intervention

The intervention protocol for different intensities RT was designed following American College of Sports Medicine (ACSM) guidelines and supported by previous research on RT methodologies (47–49). The program spanned 12 weeks, with sessions lasting 1 h, three times per week, replacing the standard physical education (PE) curriculum (23). Intensity levels were set at 45–50% one repetition maximum (1RM) for LI, 60–65% 1RM for MI, and 75–80% 1RM for HI. The 1RM for all exercises was assessed using the ACSM-recommended incremental load protocol, where participants first completed a 5–10 min warm-up with submaximal weights (approximately 50–60% of estimated 1RM) (50). Following the warm-up, the weight was increased in 2.5–5 kg increments based on the exercise type until the participant could no longer complete a full repetition with proper form. After each attempt, participants rested for 3–5 min to minimize fatigue. A certified strength and conditioning coach supervised the entire process to ensure safety. All 1RM testing was conducted before the first week of the study. To ensure accurate training prescription, 1RM was retested every 4 weeks during the study period (51).

From weeks 1–8, participants performed two sets per exercise, progressing to three sets from weeks 9–12. Repetitions ranged from 16 to 18 for LI, 10–12 for MI, and 6–8 for HI, with rest periods of 0.5, 1, and 1.5 min, respectively (52, 53). The exercises were performed using standard equipment, including dumbbells, barbells, resistance bands, and yoga mats. The training program consisted of 10 exercises: chest press, lat pulldown, shoulder press, squat, leg extension, leg curl, bicep curl, triceps extension, abdominal crunches, and lower back exercises (54). These exercises were performed in the sequence outlined above, adhering to the principle of prioritizing larger muscle groups first, followed by smaller muscles and core exercises, with consistency across groups maintained throughout the study (55). The warm-up included 5 min of light jogging or stationary cycling, followed by 5 min of stretching using the standard Chinese eighth set of radio gymnastics, consisting of dynamic movements targeting all major muscle groups. The cool-down involved 5 min of the same stretching exercises as the warm-up (25, 56). The CG followed the general college syllabus, performing a general physical education course with the same duration, session time, and frequency. The details of the training program and prescription are listed in Supplementary Tables 15 and 16.

Before the intervention, training volume was matched across the RT groups. Per-session volumes ranged from 2990.4 to 2995.4 kg during weeks 1–8 (2 sets) and from 4485.6 to 4493.1 kg during weeks 9–12 (3 sets), calculated based on the repetitions and training weights for each exercise, which were derived from prescribed intensities and predicted 1RM values assessed at pre-test (weights × repetitions × sets) (47). Detailed volume load data are provided in Supplementary Table 17. Exercise density was comparable across groups, with approximately 49.8–49.9 kg/min during weeks 1–8 and 74.8–74.9 kg/min during weeks 9–12, based on standardized session durations of 60 min (volume load ÷ total session time). The protocol underwent expert validation and was registered under clinical trial number NCT05530629.

### 2.3 Instrumentation

A simple questionnaire was distributed to participants to gather data on age, health, and diet, as well as the specific day (i.e., number of days since onset) and length of the menstrual cycle. The Physical Activity Stages of Change questionnaire assessed exercise behavior, classifying individuals into five stages: precontemplation, contemplation, preparation, action, and maintenance (57). Students in stages 1 and 2 were not excluded from the study based on exercise criteria. The reliability and validity of the Chinese version of this questionnaire have been validated (58). Predicted maximal oxygen uptake (VO₂max) was estimated using the Bruce submaximal treadmill protocol (59). The graded exercise test was conducted on the SH-T8919 motorized treadmill, produced by SHUA Fitness Co., Ltd., Jinjiang, China, and heart rate (HR) was monitored using the Polar H10 chest-worn heart rate sensor, manufactured by Polar Electro Oy, Kempele, Finland. The test was terminated upon reaching 85% of age-predicted HR max or volitional fatigue. VO₂max was estimated using ACSM-recommended extrapolation methods (60). The Dhm-301w ultrasonic measuring device from Zhengzhou Dingheng Electronic Technology Co., China, measured body weight and height to calculate BMI (61, 62). Researchers assessed body fat percentage by measuring the skinfold in the triceps, thigh, and supra-iliac using the Harpenden skinfold caliper model C-136 from the Idass Company, USA, in conjunction with the Jackson-Pollock equation and the Siri equation (63). It should be noted that the Jackson–Pollock skinfold method may have a 5–10% error range in individuals with higher adiposity, potentially affecting body-fat percentage estimates. Consequently, trained assistants performed multiple measurements at each site to ensure consistency and minimize bias (64). Waist circumference was measured using the inelastic (nonstretchable) fiberglass measuring tape, KMC-330 model, from the KOMELON brand, South Korea (65). All physiological and anthropometric measurement devices demonstrated adequate precision, structural durability, and user-friendly design (66, 67). In this study, daily food intake at different time periods (mealtime) is set as a covariate due to its impact on diet (68–70). Mealtime was usually categorized into ‘daytime’ and ‘nighttime’ segments, where 5:00 PM is used as the cut-off time. This article used the ratio of evening to full-day energy intake as the mealtime index (71, 72).

Participants were instructed to record food intake with exact times over 3 days, including two weekdays and one weekend day. Dietary consumption was documented using handwritten logs, with a standardized form provided to ensure consistency across participants (73). This 3-day food record collected data on energy intake, protein intake, fat intake, and carbohydrate intake with excellent reliability and validity (74). However, 3-day food records may be subject to underreporting, especially for small or culturally sensitive items, and participants may occasionally forget to record all items consumed (75). Several strategies were implemented to reduce underreporting and recall bias: participants were instructed during pre-intervention guidance to record intake immediately after consumption; research assistants conducted follow-up calls during the measurement procedure; and confidentiality was emphasized to support honest reporting (76). Four certified dietitians with nutrition degrees (one per campus) received centralized training and independently reviewed food diaries, verifying food types and portion sizes using standardized guidelines provided during participant instruction. Dietitians performed the dietary analysis using Elizabeth Stewart Hands and Associates’ Food Processor Nutrition Analysis and Fitness Software (ESHA, Version 11.12. X, 2022), an extensive and reliable food composition database (77). Following analysis, researchers averaged the nutrition intake over the 3 days to determine the daily values and mealtime index (78).

### 2.4 Data analysis

Data were analyzed using SPSS version 27. Prior to analysis, all data were screened for entry errors and outliers (79). Descriptive statistics, including means and standard errors (SE), were calculated for all variables, with standard deviations (SD) reported separately when applicable (80). The abbreviations Diff. (Difference), CI (Confidence Interval), LB (Lower Bound), and UB (Upper Bound) are used throughout the tables. To evaluate between-group differences in training HR, a one-way analysis of covariance (ANCOVA) was performed, with VO₂max and BMI included as covariates to control for physiological variability in cardiovascular responses during exercise (81). Normality was assessed using the Shapiro–Wilk and Kolmogorov–Smirnov tests, and homogeneity of variance was verified using Levene’s test before conducting one-way ANOVA (82). Group differences in baseline demographic and confounding variables were examined using one-way ANOVA, as all assumptions were met (83). Generalized Estimating Equations (GEE) was used to evaluate intervention effects across time, between groups, and their interaction in the CRCT design (84). It was chosen for its flexibility in handling missing data, unbalanced time points, and time-varying covariates without listwise deletion. GEE also does not assume normally distributed residuals, making it robust for non-normal data. It allows for multiple covariates and various correlation structures, ensuring consistent estimates even with complex data, making it ideal for CRCTs with intra-cluster correlations (85). Bonferroni-adjusted pairwise comparisons were conducted to examine between-group differences at each time point and within-group changes over time (86). Effect sizes (Cohen’s d) were calculated for all pairwise comparisons to quantify the magnitude of intervention effects (87). The Pearson product–moment correlation test was used to determine the relationships between the changes in continuous variables before and after the intervention, as the normality tests indicated that most of the dependent variables followed a normal distribution in each group (88). A two-tailed *p*-value < 0.05 was considered statistically significant (89).

## 3 Result

During the experiment, seven participants dropped out due to schedule conflicts, lack of motivation, and vacations: n_(HI)_ = 3; n_(MI)_ = 1; n_(LI)_ = 2; and n_(CG)_ = 1. Consequently, the final analysis was based on 65 participants: n_(HI)_ = 15; n_(MI)_ = 17; n_(LI)_ = 16; and n_(CG)_ = 17. After adjusting for VO₂max and BMI as covariates, ANCOVA revealed a significant difference in training HR among the groups (*F* = 582.021, *p* < 0.001). Adjusted mean values showed that the HI had the highest HR (M = 137.17), followed by the MI (M = 125.01) and LI (M = 113.78), with the lowest HR observed in the CG (M = 106.60). The observed HR differences verify the intended separation of exercise intensity levels across groups, reinforcing the internal validity of the intervention protocol. The details were shown in Supplementary Tables 18 and 19.

Descriptive statistics were calculated for demographic variables (age, height, and weight) and confounding factors (mealtime, as well as the specific day and length of the menstrual cycle). One-way ANOVA was performed to compare these variables, as all were normally distributed, as listed in Supplementary Table 20. The results in Table 1 indicated no significant differences for age (*F* = 0.312, *p* = 0.817), height (*F* = 0.076, *p* = 0.973), weight (*F* = 1.246, *p* = 0.300), mealtime (*F* = 0.249, *p* = 0.862), menstrual time (*F* = 0.104, *p* = 0.958), and menstrual cycle (*F* = 0.376, *p* = 0.771) across groups, confirming the groups’ baseline comparability in both demographic and confounding variables.

**Table 1:** Baseline characteristics of participants across all groups (mean ± SD).

Variable	LI	MI	HI	CG	*F* value	*p*-value
Height (m)	1.6 (0.04)	1.59 (0.04)	1.6 (0.04)	1.6 (0.04)	0.312	0.817
Weight (kg)	73.57 (6.62)	72.66 (6.53)	73.56 (6.62)	73.34 (6.6)	0.076	0.973
Age (year)	18.77 (0.42)	18.97 (0.43)	19.04 (0.43)	18.93 (0.43)	1.246	0.300
Mealtime (%)	42.17 (6.77)	43.76 (7.03)	41.97 (6.74)	42.49 (6.83)	0.249	0.862
Menstrual time (Day of cycle)	13.56 (7.05)	13.78 (6.30)	14.78 (7.41)	14.22 (7.61)	0.104	0.958
Menstrual cycle (Length in days)	31.22 (2.16)	31.33 (2.03)	31.56 (2.01)	31.89 (1.94)	0.376	0.771

Table 2 presents the descriptive statistics (mean and standard error) for BMI, body fat percentage, waist circumference, energy intake, protein intake, fat intake, carbohydrate intake, chest press 1RM, and squat 1RM across all groups at both pre-and post-test. The intervention groups demonstrated reductions in measures of body composition and nutritional intake, while improvements were observed in 1RM performance.

**Table 2:** Descriptive (Mean and SE) statistics of body composition, nutritional intake, and 1RM performance for all groups across the time.

Variable	Group	Pre-test	Post-test
BMI (kg/㎡)	LI	28.89 (0.76)	28.73 (0.82)
	MI	28.25 (0.74)	27.16 (0.74)
	HI	28.75 (0.76)	27.78 (0.77)
	CG	28.67 (0.76)	28.41 (0.76)
Body fat (%)	LI	28.26 (0.76)	27.00 (0.82)
	MI	27.70 (0.73)	25.26 (0.70)
	HI	27.58 (0.74)	23.68 (0.66)
	CG	27.88 (0.75)	26.91 (0.75)
Waist circumference (cm)	LI	93.22 (1.11)	91.35 (1.21)
	MI	92.25 (1.09)	88.52 (1.06)
	HI	92.93 (1.11)	87.40 (1.09)
	CG	92.95 (1.1)	91.52 (1.12)
Energy intake (kcal)	LI	2197.85 (68.72)	1822.21 (68.95)
	MI	2127.99 (66.54)	1531.06 (51.72)
	HI	2138.55 (66.87)	1676.55 (58.25)
	CG	2158.15 (67.48)	2070.39 (66.12)
Protein intake (g)	LI	80.22 (4.96)	77.18 (4.89)
	MI	79.60 (4.92)	57.05 (3.72)
	HI	81.60 (4.99)	72.80 (4.35)
	CG	80.58 (4.98)	79.46 (4.94)
Fat intake (g)	LI	70.85 (4.74)	58.83 (4.13)
	MI	70.02 (4.68)	46.88 (3.29)
	HI	69.49 (4.65)	65.71 (4.54)
	CG	70.13 (4.69)	68.55 (4.51)
Carbohydrate intake (g)	LI	296.45 (14.07)	260.01 (12.92)
	MI	302.56 (14.36)	220.60 (10.85)
	HI	304.63 (14.46)	210.13 (10.81)
	CG	301.04 (14.52)	290.39 (13.96)
Chest press 1RM (kg)	LI	30.94 (0.73)	34.32 (0.86)
	MI	31.22 (0.75)	36.74 (0.82)
	HI	30.83 (0.82)	40.41 (0.90)
	CG	31.33 (0.80)	31.91 (0.95)
Squat 1RM (kg)	LI	51.44 (0.97)	58.38 (0.70)
	MI	51.67 (0.97)	61.68 (0.70)
	HI	51.22 (0.99)	65.50 (0.85)
	CG	51.83 (0.92)	52.55 (0.74)

In Table 3, results from GEE showed that time had statistically significant effects on the amount of BMI (*χ*^2^ = 17.685, *p* < 0.001), body fat percentage (*χ*^2^ = 489.104, *p* < 0.001), waist circumference (*χ*^2^ = 1020.476, *p* < 0.001), energy intake (*χ*^2^ = 2112.914, *p* < 0.001), protein intake (*χ*^2^ = 125.627, *p* < 0.001), fat intake (*χ*^2^ = 287.389, *p* < 0.001), carbohydrate intake (*χ*^2^ = 585.466, *p* < 0.001), chest press 1RM (*χ*^2^ = 1315.085, *p* < 0.001), and squat 1RM (*χ*^2^ = 2972.298, *p* < 0.001). Meanwhile, it was found that the effect of group on BMI (*χ*^2^ = 1.195, *p* = 0.754), body fat percentage (*χ*^2^ = 4.751, *p* = 0.191), waist circumference (*χ*^2^ = 3.224, *p* = 0.358), protein intake (*χ*^2^ = 4.183, *p* = 0.242), fat intake (*χ*^2^ = 3.906, *p* = 0.272), and carbohydrate intake (*χ*^2^ = 5.112, *p* = 0.164) was not statistically significant except for energy intake (*χ*^2^ = 11.481, *p* = 0.009), chest press 1RM (*χ*^2^ = 12.464, *p* = 0.006), and squat 1RM (*χ*^2^ = 28.195, *p* < 0.001). Additionally, it was found that the effect of the time group on BMI (*χ*^2^ = 8.248, *p* = 0.041), body fat percentage (*χ*^2^ = 123.592, *p* < 0.001), waist circumference (*χ*^2^ = 381.456, *p* < 0.001), energy intake (*χ*^2^ = 1003.205, *p* < 0.001), protein intake (*χ*^2^ = 188.428, *p* < 0.001), fat intake (*χ*^2^ = 204.452, *p* < 0.001), carbohydrate intake (*χ*^2^ = 334.607, *p* < 0.001), chest press 1RM (*χ*^2^ = 652.854, *p* < 0.001), and squat 1RM (*χ*^2^ = 2083.315, *p* < 0.001) was statistically significant, indicating varying patterns over time for all variables in all groups and among groups for energy intake across time.

**Table 3:** Results of GEE for body composition, nutritional intake, and 1RM performance.

Variable	Source	Wald Chi-Square	df	*p-*value
BMI	Time	17.685	1	<0.001
	Groups	1.195	3	0.754
	Time * Group	8.248	3	0.041
Body fat percentage	Time	489.104	1	<0.001
	Groups	4.751	3	0.191
	Time * Group	123.592	3	<0.001
Waist circumference	Time	1020.476	1	<0.001
	Groups	3.224	3	0.358
	Time * Group	381.456	3	<0.001
Energy intake	Time	2112.914	1	<0.001
	Groups	11.481	3	0.009
	Time * Group	1003.205	3	<0.001
Protein intake	Time	125.627	1	<0.001
	Groups	4.183	3	0.242
	Time * Group	188.428	3	<0.001
Fat intake	Time	287.389	1	<0.001
	Groups	3.906	3	0.272
	Time * Group	204.452	3	<0.001
Carbohydrate intake	Time	585.466	1	<0.001
	Groups	5.112	3	0.164
	Time * Group	334.607	3	<0.001
Chest press RM (kg)	Time	1315.085	1	<0.001
	Groups	12.464	3	0.006
	Time * Group	652.854	3	<0.001
Squat 1RM (kg)	Time	2972.298	1	<0.001
	Groups	28.195	3	<0.001
	Time * Group	2083.315	3	<0.001

Regarding BMI, Table 4 shows that the results of the between-group comparison using the Bonferroni test at the pretest indicate no significant difference (all *p* = 1), as well as in the post-test (all *p* = 1). At the pre-test stage, the effect sizes (d) ranged from 0.03 to 0.20, indicating small effect sizes. In the post-test analysis, the effect sizes (d) ranged from d = 0.10 to d = 0.50, indicating small to moderate effects. The highest effect size was observed between the LI and MI (d = 0.50), which is a moderate effect size at the post-test.

**Table 4:** Pairwise mean comparison for BMI among groups across the time.

Variable	Time	Group	Mean diff.	SE	*p*-value	95%CI for diff. LB UB		Effect size d
BMI (kg/㎡)	Pretest	LI vs. MI	0.634	1.063	1	−1.708	2.977	0.20 (S)
		LI vs. HI	0.135	1.075	1	−2.021	2.291	0.04 (S)
		LI vs. CG	0.219	1.073	1	−1.964	2.403	0.07 (S)
		MI vs. HI	−0.499	1.061	1	−2.776	1.778	0.16 (S)
		MI vs. CG	−0.415	1.059	1	−2.651	1.821	0.13 (S)
		HI vs. CG	0.084	1.072	1	−2.045	2.214	0.03 (S)
	Posttest	LI vs. MI	1.569	1.103	1	−1.369	4.507	0.50 (M)
		LI vs. HI	0.951	1.121	1	−1.661	3.562	0.30 (S)
		LI vs. CG	0.328	1.112	1	−1.974	2.630	0.10 (S)
		MI vs. HI	−0.618	1.069	1	−2.966	1.729	0.20 (S)
		MI vs. CG	−1.241	1.060	1	−3.901	1.419	0.40 (S)
		HI vs. CG	−0.623	1.078	1	−2.989	1.744	0.20 (S)

^*^The mean difference is significant at the 0.05 level Adjustment for multiple comparisons: Bonferroni.

Regarding body fat percentage, Table 5 shows that the results of the between-group comparison using the Bonferroni test at the pretest indicate no significant difference (all *p* = 1). The effect sizes (d) ranged from 0.04 to 0.21, indicating small effect sizes. In the post-test analysis, these findings suggest significant reductions comparing the HI with LI (*p* = 0.035) and CG (*p* = 0.026). The effect sizes (d) ranged from 0.03 to 1.13, indicating small to large effect sizes. The highest effect size belonged to the comparison between the LI and HI (d = 1.13), which is a large effect size at the post-test.

**Table 5:** Pairwise mean comparison for body fat percentage among groups across the time.

Variable	Time	Group	Mean diff.	SE	*p*-value	95%CI for diff. LB UB		Effect size d
Body fat (%)	Pretest	LI vs. MI	0.560	1.054	1	−1.731	2.851	0.18 (S)
		LI vs. HI	0.680	1.056	1	−1.671	3.031	0.21 (S)
		LI vs. CG	0.380	1.063	1	−1.849	2.609	0.12 (S)
		MI vs. HI	0.120	1.040	1	−1.960	2.200	0.04 (S)
		MI vs. CG	−0.180	1.047	1	−2.297	1.937	0.06 (S)
		HI vs. CG	−0.300	1.049	1	−2.468	1.868	0.10 (S)
	Posttest	LI vs. MI	1.737	1.080	1	−1.269	4.744	0.57 (M)
		LI vs. HI	3.311	1.057	0.035	0.116	6.507	1.13 (L)
		LI vs. CG	0.090	1.111	1	−2.119	2.299	0.03 (S)
		MI vs. HI	1.574	0.964	1	−1.276	4.424	0.57 (M)
		MI vs. CG	−1.648	1.023	1	−4.496	1.201	0.55 (M)
		HI vs. CG	−3.221	0.999	0.026	−6.255	−0.188	1.12 (L)

^*^The mean difference is significant at the 0.05 level Adjustment for multiple comparisons: Bonferroni.

Regarding waist circumference, Table 6 shows that the results of the between-group comparison using the Bonferroni test at the pretest indicate no significant difference (all *p* = 1), as well as in the post-test (all *p* > 0.05). At the pre-test stage, the effect sizes (d) ranged from 0.01 to 0.21, indicating small effect sizes. In the post-test analysis, the effect sizes (d) ranged from 0.04 to 0.92, indicating small to large effect sizes. The highest effect size belonged to the comparison between the CG and HI (d = 0.92), which is a large effect size at the post-test.

**Table 6:** Pairwise mean comparison for waist circumference among groups across the time.

Variable	Time	Group	Mean diff.	SE	*p*-value	95%CI for diff. LB UB		Effect size d
Waist circumference (cm)	Pretest	LI vs. MI	0.967	1.558	1	−2.485	4.418	0.21 (S)
		LI vs. HI	0.289	1.567	1	−2.888	3.466	0.06 (S)
		LI vs. CG	0.267	1.566	1	−2.899	3.432	0.06 (S)
		MI vs. HI	−0.678	1.554	1	−3.988	2.633	0.15 (S)
		MI vs. CG	−0.700	1.552	1	−4.017	2.617	0.15 (S)
		HI vs. CG	−0.022	1.562	1	−3.091	3.046	0.01 (S)
	Posttest	LI vs. MI	2.825	1.610	1	−1.902	7.551	0.62 (M)
		LI vs. HI	3.951	1.627	0.258	−0.887	8.789	0.87 (L)
		LI vs. CG	−0.175	1.652	1	−3.475	3.124	0.04 (S)
		MI vs. HI	1.126	1.518	1	−2.327	4.580	0.26 (S)
		MI vs. CG	−3.000	1.545	0.834	−7.565	1.565	0.67 (M)
		HI vs. CG	−4.126	1.562	0.149	−8.799	0.546	0.92 (L)

^*^The mean difference is significant at the 0.05 level Adjustment for multiple comparisons: Bonferroni.

Regarding energy intake, Table 7 shows that the results of the between-group comparison using the Bonferroni test at the pretest indicate no significant difference (all *p* = 1). The effect sizes (d) ranged from 0.04 to 0.24, indicating small effect sizes. In the post-test analysis, these findings suggest significant reductions comparing the MI with LI (*p* = 0.011) and CG (*p* < 0.001), as well as HI and CG (p < 0.001). The effect sizes (d) ranged from 0.58 to 2.20, indicating moderate to large effect sizes. The highest effect size belonged to the comparison between the MI and CG (d = 2.20), which is a large effect size at the post-test.

**Table 7:** Pairwise mean comparison for energy intake among groups across the time.

Variable	Time	Group	Mean diff.	SE	*p*-value	95%CI for diff. LB UB		Effect size d
Energy intake (kcal)	Pretest	LI vs. MI	69.860	95.653	1.000	−147.192	286.912	0.24 (S)
		LI vs. HI	59.300	95.884	1.000	−153.008	271.608	0.21 (S)
		LI vs. CG	39.700	96.313	1.000	−164.480	243.880	0.14 (S)
		MI vs. HI	−10.560	94.329	1.000	−199.181	178.061	0.04 (S)
		MI vs. CG	−30.160	94.765	1.000	−227.284	166.964	0.11 (S)
		HI vs. CG	−19.600	94.998	1.000	−212.944	173.744	0.07 (S)
	Posttest	LI vs. MI	291.1578	86.186	0.011	38.185	544.131	1.20 (L)
		LI vs. HI	145.658	90.260	1.000	−107.705	399.021	0.58 (M)
		LI vs. CG	−248.180	95.525	0.113	−521.884	25.524	0.92 (L)
		MI vs. HI	−145.500	77.897	0.680	−366.542	75.542	0.65 (M)
		MI vs. CG	−539.338	83.942	<0.001	−794.359	−284.317	2.20 (L)
		HI vs. CG	−393.838	88.119	<0.001	−655.890	−131.787	1.55 (L)

^*^The mean difference is significant at the 0.05 level adjustment for multiple comparisons: Bonferroni.

Regarding protein intake, Table 8 shows that the results of the between-group comparison using the Bonferroni test at the pretest indicate no significant difference (all *p* = 1). The effect sizes (d) ranged from 0.02 to 0.10, indicating small effect sizes. In the post-test analysis, these findings suggest significant reductions comparing the MI with LI (*p* = 0.022) and CG (*p* = 0.007). The effect sizes (d) ranged from 0.12 to 1.24, indicating small and large effect sizes. The highest effect size belonged to the comparison between the MI and CG (d = 1.24), which is a large effect size at the post-test.

**Table 8:** Pairwise mean comparison for protein intake among groups across the time.

Variable	Time	Group	Mean diff.	SE	*p*-value	95%CI for diff. LB UB		Effect size d
Protein intake (g)	Pretest	LI vs. MI	0.620	6.985	1.000	−13.288	14.528	0.03 (S)
		LI vs. HI	−1.380	7.036	1.000	−15.673	12.913	0.07 (S)
		LI vs. CG	−0.360	7.026	1.000	−14.256	13.536	0.02 (S)
		MI vs. HI	−2.000	7.011	1.000	−16.488	12.488	0.10 (S)
		MI vs. CG	−0.980	7.001	1.000	−15.051	13.091	0.05 (S)
		HI vs. CG	1.020	7.052	1.000	−13.167	15.207	0.05 (S)
	Posttest	LI vs. MI	20.134	6.142	0.022	1.474	38.795	1.17 (L)
		LI vs. HI	4.381	6.546	1.000	−10.274	19.036	0.24 (S)
		LI vs. CG	−2.277	6.949	1.000	−16.759	12.205	0.12 (S)
		MI vs. HI	−15.754	5.725	0.119	−33.063	1.555	0.96 (L)
		MI vs. CG	−22.411	6.182	0.007	−41.362	−3.461	1.24 (L)
		HI vs. CG	−6.658	6.583	1.000	−22.578	9.263	0.35 (S)

^*^The mean difference is significant at the 0.05 level adjustment for multiple comparisons: Bonferroni.

Regarding fat intake, Table 9 shows that the results of the between-group comparison using the Bonferroni test at the pretest indicate no significant difference (all *p* = 1). The effect sizes (d) ranged from 0.01 to 0.07, indicating small effect sizes. In the post-test analysis, these findings suggest significant reductions comparing the MI with HI (*p* = 0.017) and CG (*p* = 0.002). The effect sizes (d) ranged from 0.16 to 1.33, indicating small to large effect sizes. The highest effect size belonged to the comparison between MI and CG (d = 1.33), which is a large effect size at the post-test.

**Table 9:** Pairwise mean comparison for fat intake among groups across the time.

Variable	Time	Group	Mean diff.	SE	*p*-value	95%CI for diff. LB UB		Effect size d
Fat intake (g)	Pretest	LI vs. MI	0.830	6.661	1.000	−12.521	14.181	0.04 (S)
		LI vs. HI	1.360	6.635	1.000	−12.141	14.861	0.07 (S)
		LI vs. CG	0.720	6.668	1.000	−12.603	14.043	0.04 (S)
		MI vs. HI	0.530	6.596	1.000	−12.583	13.643	0.03 (S)
		MI vs. CG	−0.110	6.628	1.000	−13.138	12.918	0.01 (S)
		HI vs. CG	−0.640	6.602	1.000	−13.805	12.525	0.03 (S)
	Posttest	LI vs. MI	11.947	5.280	0.450	−3.936	27.829	0.81 (L)
		LI vs. HI	−6.877	6.136	1.000	−22.098	8.343	0.40 (S)
		LI vs. CG	−9.727	6.114	1.000	−26.671	7.218	0.56 (M)
		MI vs. HI	−18.824	5.601	0.017	−35.917	−1.731	1.18 (L)
		MI vs. CG	−21.673	5.577	0.002	−38.768	−4.579	1.33 (L)
		HI vs. CG	−2.849	6.393	1.000	−16.495	10.797	0.16 (S)

*The mean difference is significant at the 0.05 level adjustment for multiple comparisons: Bonferroni.

Regarding carbohydrate intake, Table 10 shows that the results of the between-group comparison using the Bonferroni test at the pretest indicate no significant difference (all *p* = 1). The effect sizes (d) ranged from 0.02 to 0.14, indicating small effect sizes. In the post-test analysis, these findings suggest significant reductions comparing the HI with LI (*p* = 0.049) and CG (*p* < 0.001), as well as MI and CG (*p* = 0.001). The effect sizes (d) ranged from 0.24 to 1.58, indicating small to large effect sizes. The highest effect size belonged to the comparison between the HI and CG (d = 1.58), which is a large effect size at the post-test.

**Table 10:** Pairwise mean comparison for carbohydrate intake among groups across the time.

Variable	Time	Group	Mean diff.	SE	*p*-value	95%CI for diff. LB UB		Effect size d
Carbohydrate intake (g)	Pretest	LI vs. MI	−6.110	20.103	1.000	−47.809	35.589	0.10 (S)
		LI vs. HI	−8.180	20.173	1.000	−50.887	34.527	0.14 (S)
		LI vs. CG	−4.590	20.215	1.000	−45.896	36.716	0.08 (S)
		MI vs. HI	−2.070	20.378	1.000	−42.741	38.601	0.03 (S)
		MI vs. CG	1.520	20.420	1.000	−39.034	42.074	0.02 (S)
		HI vs. CG	3.590	20.488	1.000	−37.864	45.044	0.06 (S)
	Posttest	LI vs. MI	39.407	16.870	0.292	−10.110	88.923	0.83 (L)
		LI vs. HI	49.873	16.847	0.049	0.089	99.658	1.07 (L)
		LI vs. CG	−30.388	19.018	1.000	−84.353	23.578	0.56 (M)
		MI vs. HI	10.467	15.319	1.000	−23.935	44.869	0.24 (S)
		MI vs. CG	−69.794	17.679	0.001	−122.368	−17.220	1.35 (L)
		HI vs. CG	−80.261	17.657	<0.001	−133.643	−26.879	1.58 (L)

^*^The mean difference is significant at the 0.05 level adjustment for multiple comparisons: Bonferroni.

Regarding chest press 1RM, Table 11 shows that the results of the between-group comparison using the Bonferroni test at the pretest indicate no significant difference (all *p* = 1). The effect sizes (d) ranged from 0.03 to 0.15, indicating small effect sizes. In the post-test analysis, these findings suggest significant increases comparing the HI with MI (*p* = 0.041), LI (*p* < 0.001), and CG (*p* < 0.001), as well as MI and CG (*p* = 0.002). The effect sizes (d) ranged from 0.63 to 2.16, indicating moderate to large effect sizes. The highest effect size belonged to the comparison between the HI and CG (d = 2.16), which is a large effect size at the post-test.

**Table 11:** Pairwise mean comparison for chest press 1RM among groups across the time.

Variable	Time	Group	Mean diff.	SE	*p*-value	95%CI for diff. LB UB		Effect size d
Chest press 1RM (kg)	Pretest	LI vs. MI	−0.2778	1.04830	1.000	−2.4356	1.8801	0.09 (S)
		LI vs. HI	0.1111	1.10477	1.000	−2.0934	2.3156	0.03 (S)
		LI vs. CG	−0.3889	1.08716	1.000	−2.6683	1.8905	0.12 (S)
		MI vs. HI	0.3889	1.11335	1.000	−1.9414	2.7192	0.12 (S)
		MI vs. CG	−0.1111	1.09588	1.000	−2.2982	2.0760	0.03 (S)
		HI vs. CG	−0.5000	1.15001	1.000	−2.9493	1.9493	0.15 (S)
	Posttest	LI vs. MI	−2.4215	1.19311	0.509	−5.8401	0.9971	0.68 (M)
		LI vs. HI	−6.0870a	1.24625	0.000	−9.8355	−2.3386	1.63 (L)
		LI vs. CG	2.4160	1.28493	0.601	−1.1908	6.0229	0.63 (M)
		MI vs. HI	−3.6655a	1.21647	0.041	−7.2604	−0.0706	1.00 (L)
		MI vs. CG	4.8376a	1.25607	0.002	1.1022	8.5729	1.28 (L)
		HI vs. CG	8.5031a	1.30664	0.000	4.5151	12.4910	2.16 (L)

^*^The mean difference is significant at the 0.05 level adjustment for multiple comparisons: Bonferroni.

Regarding squat 1RM, Table 12 shows that the results of the between-group comparison using the Bonferroni test at the pretest indicate no significant difference (all *p* = 1). The effect sizes (d) ranged from 0.04 to 0.15, indicating small effect sizes. In the post-test analysis, these findings suggest significant increases between each pair of groups (all *p* ≤ 0.008). The effect sizes (d) ranged from 1.11 to 3.83, indicating a large effect size. The highest effect size belonged to the comparison between the HI and CG (d = 3.83), which is a large effect size at the post-test.

**Table 12:** Pairwise mean comparison for squat 1RM among groups across the time.

Variable	Time	Group	Mean diff.	SE	*p*-value	95%CI for diff. LB UB		Effect size d
Squat 1RM (kg)	Pretest	LI vs. MI	−0.2222	1.37037	1.000	−2.9881	2.5436	0.06 (S)
		LI vs. HI	0.2222	1.38506	1.000	−2.5724	3.0168	0.05 (S)
		LI vs. CG	−0.3889	1.33904	1.000	−3.1590	2.3812	0.10 (S)
		MI vs. HI	0.4444	1.38456	1.000	−2.4372	3.3261	0.11 (S)
		MI vs. CG	−0.1667	1.33853	1.000	−2.8494	2.5161	0.04 (S)
		HI vs. CG	−0.6111	1.35356	1.000	−3.5039	2.2817	0.15 (S)
	Posttest	LI vs. MI	−3.3067a	0.98010	0.008	−6.0878	−0.5255	1.11 (L)
		LI vs. HI	−7.1270a	1.09168	0.000	−10.3734	−3.8805	2.15 (L)
		LI vs. CG	5.8123a	1.01188	0.000	2.8422	8.7824	1.91 (L)
		MI vs. HI	−3.8203a	1.09253	0.006	−6.9507	−0.6899	1.16 (L)
		MI vs. CG	9.1190a	1.01280	0.000	6.0420	12.1959	2.99 (L)
		HI vs. CG	12.9393a	1.12114	0.000	9.5856	16.2930	3.83 (L)

^*^The mean diff is significant at the 0.05 level adjustment for multiple comparisons: Bonferroni.

In the within-group comparison, which is listed in Table 13, it was found that for BMI, there was a significant reduction between pre-and post-test only in MI (*p* = 0.013, d = 0.35). Regarding body fat percentage, there were significant reductions between pre-and post-test in all groups (all *p* < 0.001; LI: d = 0.40, MI: d = 0.81, HI: d = 1.34, CG: d = 0.31). Regarding waist circumference, there were significant reductions between pre-and post-test in all groups (all *p* < 0.001; LI: d = 0.40, MI: d = 0.83, HI: d = 1.22, CG: d = 0.31). Regarding energy intake, there was a significant reduction between pre-and post-test in all groups (all *p* < 0.001; LI: d = 1.34, MI: d = 2.39, HI: d = 1.78, CG: d = 0.31). Regarding protein intake, there were significant reductions between pre and post-test in LI (*p* < 0.001, d = 0.15), MI (*p* < 0.001, d = 1.23), and HI (*p* = 0.017, d = 0.45). Regarding fat intake, there were significant reductions between pre-and post-test in LI (*p* < 0.001, d = 0.66) and MI (*p* < 0.001, d = 1.36). Regarding carbohydrate intake, there were significant reductions between pre-and post-test in all groups (all *p* < 0.001; LI: d = 0.66, MI: d = 1.53, HI: d = 1.78, CG: d = 0.18). Regarding chest press 1RM, there were significant increases between pre-and post-test in LI (*p* < 0.001, d = 1.00), MI (*p* < 0.001, d = 1.66), and HI (*p* < 0.001, d = 2.62). Regarding squat 1RM, there were significant increases between pre-and post-test in all groups (all *p* < 0.029; LI: d = 1.93, MI: d = 2.79, HI: d = 3.65, CG: d = 0.20).

**Table 13:** Pairwise mean comparison for body composition, nutritional intake, and 1RM performance across the time for all groups.

Variable	Group	Time	Mean diff.	SE	*p*-value	95%CI for diff. LB UB		Effect size d
BMI	LI	Pre vs. Post	0.152	0.269	1.000	−0.437	0.741	0.05 (S)
	MI	Pre vs. Post	1.086^*^	0.310	0.013	0.118	2.055	0.35 (S)
	HI	Pre vs. Post	0.967	0.366	0.222	−0.172	2.107	0.31 (S)
	CG	Pre vs. Post	0.261	0.204	1.000	−0.264	0.785	0.08 (S)
Body fat percentage	LI	Pre vs. Post	1.263^*^	0.207	<0.001	0.622	1.906	0.40 (S)
	MI	Pre vs. Post	2.441^*^	0.154	<0.001	1.961	2.921	0.81 (L)
	HI	Pre vs. Post	3.895^*^	0.220	<0.001	3.209	4.581	1.34 (L)
	CG	Pre vs. Post	0.974^*^	0.188	<0.001	0.393	1.554	0.31 (S)
Waist circumference	LI	Pre vs. Post	1.868^*^	0.238	<0.001	1.131	2.605	0.40 (S)
	MI	Pre vs. Post	3.726^*^	0.220	<0.001	3.038	4.415	0.83 (L)
	HI	Pre vs. Post	5.530	0.164	<0.001	5.021	6.040	1.22 (L)
	CG	Pre vs. Post	1.426^*^	0.149	<0.001	0.965	1.888	0.31 (S)
Energy intake	LI	Pre vs. Post	375.637	22.848	<0.001	304.267	447.008	1.34 (L)
	MI	Pre vs. Post	596.935	17.910	<0.001	541.182	652.688	2.39 (L)
	HI	Pre vs. Post	461.995	13.386	<0.001	420.474	503.516	1.78 (L)
	CG	Pre vs. Post	87.757	8.652	<0.001	61.021	114.493	0.31 (S)
Protein intake	LI	Pre vs. Post	3.036	0.586	<0.001	1.212	4.861	0.15 (S)
	MI	Pre vs. Post	22.551	1.393	<0.001	18.198	26.903	1.23 (L)
	HI	Pre vs. Post	8.797	2.614	0.017	0.819	16.776	0.45 (S)
	CG	Pre vs. Post	1.119	0.957	1.000	−1.281	3.520	0.05 (S)
Fat intake	LI	Pre vs. Post	12.022	1.079	<0.001	8.653	15.392	0.66 (M)
	MI	Pre vs. Post	23.139	1.557	<0.001	18.293	27.985	1.36 (L)
	HI	Pre vs. Post	3.785	1.315	0.084	−0.210	7.780	0.20 (S)
	CG	Pre vs. Post	1.576	0.630	0.249	−0.331	3.482	0.08 (S)
Carbohydrate intake	LI	Pre vs. Post	36.443	3.611	<0.001	25.163	47.724	0.66 (M)
	MI	Pre vs. Post	81.960	4.062	<0.001	69.314	94.606	1.53 (L)
	HI	Pre vs. Post	94.497	7.178	<0.001	72.230	116.764	1.78 (L)
	CG	Pre vs. Post	10.646	2.070	<0.001	4.250	17.042	0.18 (S)
Chest press 1RM	LI	Pre vs. Post	−3.379	0.31672	<0.001	−4.368	−2.3896	1.00 (L)
	MI	Pre vs. Post	−5.523	0.20039	<0.001	−6.147	−4.8989	1.66 (L)
	HI	Pre vs. Post	−9.578	0.22968	<0.001	−10.290	−8.8647	2.62 (L)
	CG	Pre vs. Post	−0.574	0.28777	0.509	−1.391	0.2429	0.16 (S)
Squat 1RM	LI	Pre vs. Post	−6.921	0.374	<0.001	−8.089	−5.753	1.93 (L)
	MI	Pre vs. Post	−10.006	0.336	<0.001	−11.043	−8.968	2.79 (L)
	HI	Pre vs. Post	−14.270	0.178	<0.001	−14.809	−13.732	3.65 (L)
	CG	Pre vs. Post	−0.720	0.242	0.029	−1.398	−0.041	0.20 (S)

* The mean difference is significant at the 0.05 level adjustment for multiple comparisons: Bonferroni.

Supplementary Figures 3–11 show changes in body composition, nutritional intake, and 1RM performance over time. MI and HI experienced significant decreases in all body composition variables, while CG and LI exhibited small changes. This indicates that higher-intensity interventions were more effective in reducing these body composition metrics. MI and HI experienced significant decreases in energy and carbohydrate intake, while CG and LI exhibited small changes. MI experienced significant decreases in protein and fat intake, while other groups exhibited small changes. This indicates that higher-intensity interventions (especially MI) were more effective in reducing these nutritional intake metrics. All RT groups experienced significant increases in chest press and squat 1RM, with greater increases observed at higher intensity levels, while the CG showed only minor changes in both chest press and squat 1RM. This suggests that higher-intensity RT was more effective in enhancing these strength measures.

Table 14 presents the correlations between changes in body composition and nutritional intake across the four study groups. In the LI, the change in body fat percentage was significantly and negatively correlated with the change in fat intake (r = −0.545, *p* < 0.05), and the change in waist circumference was significantly and negatively correlated with the change in protein intake (r = −0.522, *p* < 0.05). In the HI, significantly positive correlations were found between the change in BMI with changes in protein (r = 0.699, *p* < 0.01) and carbohydrate intake (r = 0.564, *p* < 0.05), while the change in fat intake was significantly and negatively correlated with the change in BMI (r = −0.632, *p* < 0.01). Similar patterns were observed for change in body fat percentage, which was significantly and positively correlated with changes in protein (r = 0.665, *p* < 0.01) and carbohydrate intake (r = 0.839, *p* < 0.01), but negatively correlated with fat intake (r = −0.548, *p* < 0.05). The CG displayed significantly negative correlations between changes in BMI and body fat percentage with the change in protein intake (r = −0.749 and −0.747, respectively, both *p* < 0.01), as well as the change in carbohydrate intake (r = −0.838 and −0.606, respectively, *p* < 0.01). Additionally, the change in waist circumference was significantly and positively correlated with changes in protein (r = 0.595, *p* < 0.05) and carbohydrate intake (r = 0.563, *p* < 0.05). No significant correlations were found in the MI.

**Table 14:** Relationship (r) between changes in body composition and nutritional intake (post-pre).

Group	Variable	Energy intake	Protein intake	Fat intake	Carbohydrate intake
LI	BMI	−0.317	0.035	−0.228	0.320
	Body fat percentage	0.388	−0.243	−0.545^*^	−0.233
	Waist circumference	−0.235	−0.522^*^	−0.113	0.424
MI	BMI	−0.179	0.149	0.085	−0.117
	Body fat percentage	0.090	0.203	0.067	0.058
	Waist circumference	0.038	0.207	0.237	0.165
HI	BMI	−0.357	0.699^**^	−0.632^**^	0.564^*^
	Body fat percentage	−0.006	0.665^**^	−0.548^*^	0.839^**^
	Waist circumference	0.084	0.513^*^	−0.139	0.348
CG	BMI	−0.121	−0.749^**^	0.161	−0.838^**^
	Body fat percentage	0.237	−0.747^**^	0.152	−0.606^**^
	Waist circumference	−0.011	0.595^*^	0.188	0.563^*^

* Correlation is significant at the 0.05 level (2-tailed).

** Correlation is significant at the 0.01 level (2-tailed).

## 4 Discussion

Regarding body composition, based on the results, the study found the most significant reduction in body fat percentage with high-intensity RT, compared to others at the post-test. In detail, compared to the standard PE course, high-intensity RT significantly reduced body fat percentage. The researcher’s finding is consistent with previous studies, which have shown a beneficial effect of high-intensity RT on body fat percentage (90, 91). In addition, one study showed that after 8 weeks of high-intensity RT, the body fat percentage of overweight and obese males in adulthood was significantly lower than that of the control group (92).

In addition, the results of this study indicate that high-intensity RT significantly reduces body fat more than low-intensity RT. The study by Villanueva et al. (93) showed that after 12 weeks of high-intensity RT, there was a significant decrease in body fat percentage in older adults, regardless of whether the rest time between sets was long or short. The study by Ogawa et al. (94) showed that in cardiovascular surgery patients, 3 months of low-intensity RT did not significantly improve body fat rate. However, another direct study by Fan et al. (34) showed that for patients with type 2 diabetes, there was no significant difference in the average body fat percentage between high-intensity and low-intensity exercise subgroups. This discrepancy may stem from the fact that previous studies largely involved clinical or more vulnerable populations, whose prescribed resistance-training volumes were substantially lower than those of the 12-week protocol used in this study (95, 96). It is well established that total training volume is a key determinant of fat-loss adaptations, with higher volumes producing greater reductions in adiposity (97, 98).

This study ensured that the training volume was sufficient and consistently applied across all groups, which is essential for understanding the reasons behind the observed results. High-intensity RT is more effective in reducing body fat percentage, primarily due to its increased energy expenditure and excess post-exercise oxygen consumption. This type of training stimulates larger muscle groups and leads to more significant metabolic stress, resulting in higher calorie burns both during and after exercise (99, 100). Studies by Schoenfeld et al. (98) and Keating et al. (101) have shown that high-intensity training elevates resting metabolic rate and promotes greater fat oxidation, contributing to more substantial fat loss. Low-intensity RT, while beneficial for muscle endurance and overall health, is less effective in reducing body fat percentage. The lower energy demand of this training type results in less calorie burn during exercise (102). According to studies by Aristizabal et al. (90) and Stavres et al. (103), low-intensity training does not significantly elevate the metabolic rate post-exercise, leading to a lesser impact on body fat reduction than high-intensity RT. Standard PE courses often need more consistency and intensity of exercise for significant fat loss, as Osipov et al. (104) found, offering diverse activities with limited fat-burning potential. Therefore, the targeted, intensity-driven nature of high-intensity RT proves superior in achieving substantial reductions in body fat compared to a standard PE course.

Regarding nutritional intake, based on the results, the study found the most significant reduction in nutritional intake with moderate- (especially) and high-intensity RT, compared to others at the post-test. To be more specific, regarding energy intake, the HI demonstrated a notable reduction in energy intake compared to the CG, aligning indirectly with prior research. Damour et al. (105) found that energy intake decreased after 4 weeks of high-intensity training among overweight and obese adults, regardless of when the exercise was performed. Another study by Miguet et al. (106) showed a clinically significant decrease in energy intake after 12 weeks of high-intensity exercise in 30 overweight and sedentary men. In addition, the study by Sheikholeslami-Vatani and Rostamzadeh (107) also suggests that high-intensity exercise may alter appetite-regulating hormones, leading to a subsequent decrease in energy intake. However, another direct study by Feito et al. (108) reported no significant difference in energy intake between HI and CG over 12 weeks with four sessions per week in male adults. These results may stem from variations in population categories or training frequency.

This study also confirmed a reduction in energy intake in the MI compared to the CG, indirectly consistent with prior findings. Echeverria et al. (109) reported that 12 weeks of moderate-intensity RT significantly improved individual nutritional status in older adults after hospitalization. Similarly, Rahmani Ghobadi et al. (110) observed a significant reduction in energy intake in overweight women following 8 weeks of moderate-intensity training. However, Oppert et al. (111) found no significant improvement in energy intake among obese women undergoing gastric bypass after 18 weeks of moderate-intensity RT compared to the control condition. Different exercise durations or populations may cause this.

The MI also showed a significant reduction in energy intake compared to the LI, indirectly consistent with prior studies. Tavassoli et al. (112) and Clark (113) found that moderate-intensity RT significantly improves appetite-regulating biomarkers, such as acetylated ghrelin and nesfatin-1, which influence food intake. Meanwhile, Kim and Kim (25) found no significant change in energy intake following 12 weeks of low-intensity RT in obese men. However, Morton et al. (114) reported no significant difference in energy intake between MI and LI over 12 weeks with four sessions per week in young men, possibly due to differences in training frequency or population characteristics.

Compared to the standard PE course, high-intensity RT reduces energy intake, mainly due to higher intensity boosting metabolic rate and fat oxidation, enabling efficient use of fat reserves and reducing dietary dependence (115, 116). Moderate-intensity RT significantly reduces energy intake compared to the standard PE course and low-intensity training by not only increasing metabolic rate but also balancing hormonal effects. Its intensity is sufficient to stabilize blood sugar without causing hormonal disruptions, aiding in energy intake regulation (117, 118). Low-intensity RT is less effective in reducing energy intake due to its insufficient intensity, which limits metabolic demands and hormonal responses (119, 120). Similarly, the standard PE course faces these challenges, as the typically low intensity, combined with the absence of structured discipline, leads to irregular exercise patterns and makes consistent dietary control more difficult (121, 122).

Regarding protein intake, compared to the CG, the MI showed a significant decrease in protein intake. This result aligns with prior research. Oppert et al. (111) found that moderate-intensity RT after 18 weeks led to a notable improvement in protein intake among obese women undergoing Roux-en-Y gastric bypass. Meanwhile, Jurado-Fasoli et al. (123) reported that 12 weeks of low-to moderate-intensity combined training, including RT, significantly reduced protein intake in sedentary middle-aged adults. Additionally, the MI showed a significant decrease in protein intake compared to the LI. Indirect evidence supports this finding. Nunes et al. (124) reported a decrease in protein intake after 18 months of moderate-intensity RT among middle-aged and older men. Kim and Kim (25) observed an insignificant change in protein intake among obese men after 12 weeks of low-intensity RT. However, Morton et al. (114) found no significant difference in protein intake between moderate-and low-intensity training groups after 12 weeks with four sessions per week in young men. Differences in training frequency or population may cause this difference.

Moderate-intensity RT reduces protein intake mainly by optimizing muscle protein synthesis, enhancing efficiency, and balancing appetite-regulating hormones like ghrelin and leptin (125, 126). Low-intensity RT, due to insufficient muscle engagement, and standard PE courses, without specific and targeted exercise stimuli, both show limited effectiveness in stimulating muscle protein synthesis or regulating appetite-related hormones. Consequently, reductions in protein cravings and intake are less pronounced compared to moderate-intensity training (127, 128).

Regarding fat intake, this study demonstrated a significant reduction in fat intake in the MI compared to the CG, aligning with indirect evidence. For instance, Oppert et al. (111) reported that fat intake among obese adult women significantly decreased following 18 weeks of moderate-intensity RT. However, Houben et al. (129) found no significant difference in fat intake between children engaging in moderate-intensity RT twice weekly and the control condition after 20 weeks. These discrepancies may stem from variations in exercise duration or population characteristics. Additionally, this study showed that participants in MI significantly reduced fat intake compared to those in HI. An indirect study by Cipryan et al. (130) confirmed the result, which showed that increasing single-exercise intensity would stimulate subsequent fat intake among normal-weight participants. However, Dupuit et al. (131) found no significant difference in fat intake between the HI and MI after a 12-week intervention conducted three times per week among postmenopausal women. Differences in population may explain these conflicting results.

Regarding reason, moderate-intensity RT can more effectively reduce dietary fat intake. Moderate-intensity training can moderately improve fat oxidation ability and activate the secretion of appetite-regulating hormones such as GLP-1 and PYY, thereby reducing the desire for fat intake (132). The regulatory effect on key hormones such as leptin and insulin are milder and more stable, which can continuously improve leptin sensitivity and enhance satiety (133, 134). Conversely, due to the compensatory eating behaviors after intense exercise, while total energy intake decreases after high-intensity RT, the amount of fat in the diet may not change substantially due to the significant increase in the proportion of fat intake (135, 136). Additionally, the standard PE course often fails to alter fat consumption patterns due to varied activities lacking consistent effects on appetite and energy balance. Without a focused exercise regimen, their influence on dietary fat intake is minimal (137, 138).

Regarding carbohydrate intake, participants in HI significantly reduced it compared to those in CG. Krings et al. (139) demonstrated that male college students decreased carbohydrate intake after 6 weeks of high-intensity RT, indirectly supporting the findings of this study. However, Benito et al. (140) found no significant difference in carbohydrate intake after 24 weeks of high-intensity RT compared to the control condition among overweight men. This discrepancy may be due to differences in training durations and populations. Similarly, participants in MI also significantly reduced carbohydrate intake compared to those in CG, indirectly aligning with Halliday et al. (27), who observed reduced carbohydrate intake in middle-aged and older adults with pre-diabetes following 9 months of moderate-intensity RT. However, Oppert et al. (111) reported no significant improvement in carbohydrate intake after 18 weeks of moderate-intensity RT among obese women undergoing Roux-en-Y gastric bypass compared to the control condition, likely due to differing durations or populations. Additionally, this study suggests that participants in HI reduced carbohydrate intake more effectively than those in LI, aligning with indirect evidence. Kim and Kim (25) found that 12 weeks of low-intensity RT did not significantly affect carbohydrate intake in overweight adult women. Rostamzadeh and Sheikholeslami-Vatani (141) highlighted that after 6 months of high-intensity RT, the carbohydrate intake was significantly reduced among obese males. Similarly, Halliday et al. (142) demonstrated that 12 weeks of moderate-to high-intensity RT significantly reduced the carbohydrate intake among prediabetic women with overweight and obesity.

High-and moderate-intensity RT showed a more significant reduction in carbohydrate intake due to its intense nature, promoting muscle metabolism adaptations (143). It enhances glycogen storage and usage efficiency, lowering daily carbohydrate needs for maintenance and recovery (144, 145). Conversely, low-intensity RT elicits only modest glycogen turnover and minimal perturbation of appetite-regulating hormones. Post-exercise changes in ghrelin and peptide YY are negligible after low-intensity RT (33, 146), so reductions in carbohydrate intake are markedly smaller than those observed following high-intensity RT. Additionally, due to the lack of consistent intensity and targeted progress, the specific focus of the standard PE course on metabolic efficiency and muscle endurance is limited, resulting in less significant changes in carbohydrate intake compared to moderate-and high-intensity RT (106).

Additionally, some effect sizes reported in this study, while considered small, can still have practical significance in certain contexts, particularly in overweight and obese populations (147). Small effect sizes may indicate subtle but meaningful improvements in outcomes, such as the difference in protein intake between participants in LI and HI at post-test. These modest changes, while small individually, can accumulate over time and be especially impactful in long-term interventions (148). Furthermore, even small effects across multiple measures can accumulate into clinically meaningful changes, particularly when considering their potential influence on overall health and well-being. Therefore, while some effect sizes in this study may be small, these differences still contribute to the overall impact of RT intensity on health outcomes (149). Future research should investigate how these small improvements might lead to significant long-term changes in health and quality of life.

Regarding the correlation, overall, the change in nutritional intake, focusing on protein and carbohydrate intake, was positively correlated with body composition in the HI but negatively correlated in the CG. Regarding the positive correlation in the HI, these findings are consistent with previous research. For example, Huang et al. (150) conducted a meta-analysis of various studies on high-intensity RT and reported that it consistently led to substantial reductions in body fat while also influencing macronutrient intake, particularly reducing protein and carbohydrate consumption. Furthermore, a systematic review by de Assis and Murawska-Ciałowicz (151) suggested that high-intensity exercise altered body composition through fat loss and muscle gain while modulating appetite-regulating hormones such as leptin and ghrelin to reduce food intake. Similarly, another study by de Moraes et al. (152) involved overweight adults participating in a 4-week intervention with intermittent fasting and RT, showing that high-intensity RT not only reduces fat mass but also decreases hunger and food intake due to changes in energy balance.

Regarding the negative correlation in the CG, this result is in line with previous studies. For example, a meta-analysis by Hubner et al. (153) suggests that moderate-intensity AT led to reductions in body fat but did not significantly suppress total food intake and protein consumption among older adults. Similarly, Yoon et al. (154) observed the effects of a 16-week moderate-intensity AT involving middle school students. While body fat percentage and waist circumference decreased, there were no significant changes in carbohydrate intake. Furthermore, Afrasyabi et al. (155) conducted a 12-week HIIT program in individuals with type 2 diabetes. Despite reductions in body fat, the feeling of satiety was not significantly impacted, and overall dietary intake did not decrease.

Regarding the reasons for the correlations, high-intensity RT leads to an increase in muscle mass and fat loss (132). The increase in muscle mass has a stronger correlation than fat loss with appetite-regulating hormones such as leptin and ghrelin, which are key in hunger and satiety regulation (16, 156). Meanwhile, higher muscle mass is associated with improved insulin sensitivity, which further contributes to appetite regulation (157). Furthermore, the psychological effects of exercise, such as enhanced mood and reduced stress following RT, may correlate to reduced food cravings (158). In contrast, moderate-and low-intensity AT, as typically included in the standard PE course, does not induce the same increase in muscle mass, but due to the significant loss of fat, resting metabolic rate will decrease (159). Research indicates that hunger increases significantly when resting metabolic rate decreases—up to at least three times—due to fat loss, which is associated with compensatory increases in food intake (160). In summary, most of the previous studies can support the findings of this study. However, the different training frequencies, durations, or affected populations may result in different results.

## 5 Limitation

The present study’s sample was restricted to Chinese college women, limiting external validity across genders, age groups, and cultural contexts. The specific dietary habits, campus lifestyles, and physical-activity patterns in Chinese universities may not reflect other populations. Future research should employ multicenter and cross-cultural designs that include male participants, older adults, and diverse ethnic groups to improve generalizability (161, 162). Additionally, this research focused solely on RT intensity, without comparing it to alternative forms of exercise. Future studies should explore different exercise modalities and their impacts on body composition and nutritional intake (25, 163). Moreover, dietary assessments based on self-reporting, frequently utilized in large cohort studies, are susceptible to bias, yet such methods remain cost-effective and feasible for extensive research (164, 165). To enhance accuracy, future research should incorporate more precise methods such as weighed food records or doubly labeled water (166, 167). Lastly, this study focused on body composition and nutritional intake, and future studies should consider other factors, including appetite regulation-related psychological and physiological indicators, to better understand the potential mechanisms of the effect of RT on body composition and nutritional intake (141, 168).

## 6 Conclusion

This study found significant effects of different RT intensities on body composition and nutritional intake among overweight and obese college women, with high-intensity training showing the best results for body composition; both moderate- (especially) and high-intensity training yielding the best outcomes for nutritional intake; and high-intensity training demonstrating the best correlation between changes in body composition and nutritional intake. Overall, the high-intensity RT can significantly reduce the body fat percentage compared with the low-intensity RT and standard PE course. Moderate-intensity RT can significantly reduce all nutritional intake variables compared with other intensities and the standard PE course. Meanwhile, high-intensity RT significantly reduces energy and carbohydrate intake compared to the low-intensity RT and standard PE course. The correlation between changes in body composition and nutritional intake is positive in the HI but negative in the CG. However, previous studies have shown differing post-test comparison results for body fat percentage between the HI and LI compared to this study, as discussed above. Therefore, further experiments are recommended to resolve this discrepancy, alongside addressing limitations related to greater population diversity, comparisons with alternative exercise modalities, more rigorous dietary assessment methods, and the underlying mechanisms contributing to the observed effects.

## Data Availability

The original contributions presented in the study are included in the article/Supplementary material, further inquiries can be directed to the corresponding author.
