# Supplementary material for: Impact of resistance training intensity on body composition and nutritional intake among college women with overweight and obesity: a cluster randomized controlled trial
Source: Front Public Health. 2025 May 30;13:1589036. doi: 10.3389/fpubh.2025.1589036 (PMC12162680; doi:10.3389/fpubh.2025.1589036)

**GEE.final spv body composition and nutritional intake**

* Generalized Estimating Equations.

GENLIN BMI BY TIME GROUP (ORDER=ASCENDING)

/MODEL TIME GROUP TIME*GROUP INTERCEPT=YES

DISTRIBUTION=NORMAL LINK=IDENTITY

/CRITERIA SCALE=MLE PCONVERGE=1E-006(ABSOLUTE) SINGULAR=1E-012 ANALYSISTYPE=3(WALD) CILEVEL=95

LIKELIHOOD=FULL

/EMMEANS TABLES=TIME*GROUP SCALE=ORIGINAL COMPARE=TIME*GROUP CONTRAST=PAIRWISE

PADJUST=SEQBONFERRONI

/REPEATED SUBJECT=ID WITHINSUBJECT=TIME SORT=YES CORRTYPE=AR(1) ADJUSTCORR=YES COVB=ROBUST

MAXITERATIONS=100 PCONVERGE=1e-006(ABSOLUTE) UPDATECORR=1

/MISSING CLASSMISSING=EXCLUDE

/PRINT CPS DESCRIPTIVES MODELINFO FIT SUMMARY SOLUTION.

**Generalized Linear Models**

| **Notes** | | |
| --- | --- | --- |
| Output Created | | 09-JUN-2023 00:04:39 |
| Comments | |  |
| Input | Data | C:\Users\mdanaee\Google Drive\Data Analysis\Prof SOh students\Prof Soh -Wang\DATA GEEcr.sav |
|  | Active Dataset | DataSet1 |
|  | Filter | <none> |
|  | Weight | <none> |
|  | Split File | <none> |
|  | N of Rows in Working Data File | 144 |
| Missing Value Handling | Definition of Missing | User-defined missing values for factor, subject and within-subject variables are treated as missing. |
|  | Cases Used | Statistics are based on cases with valid data for all variables in the model. |
| Weight Handling | | not applicable |
| Syntax | | GENLIN BMI BY TIME GROUP (ORDER=ASCENDING)  /MODEL TIME GROUP TIME*GROUP INTERCEPT=YES  DISTRIBUTION=NORMAL LINK=IDENTITY  /CRITERIA SCALE=MLE PCONVERGE=1E-006(ABSOLUTE) SINGULAR=1E-012 ANALYSISTYPE=3(WALD) CILEVEL=95  LIKELIHOOD=FULL  /EMMEANS TABLES=TIME*GROUP SCALE=ORIGINAL COMPARE=TIME*GROUP CONTRAST=PAIRWISE  PADJUST=SEQBONFERRONI  /REPEATED SUBJECT=ID WITHINSUBJECT=TIME SORT=YES CORRTYPE=AR(1) ADJUSTCORR=YES COVB=ROBUST  MAXITERATIONS=100 PCONVERGE=1e-006(ABSOLUTE) UPDATECORR=1  /MISSING CLASSMISSING=EXCLUDE  /PRINT CPS DESCRIPTIVES MODELINFO FIT SUMMARY SOLUTION. |
| Resources | Processor Time | 00:00:00.39 |
|  | Elapsed Time | 00:00:00.19 |

| **Model Information** | | |
| --- | --- | --- |
| Dependent Variable | | BMI |
| Probability Distribution | | Normal |
| Link Function | | Identity |
| Subject Effect | 1 | ID |
| Within-Subject Effect | 1 | TIME |
| Working Correlation Matrix Structure | | AR(1) |

| **Case Processing Summary** | | |
| --- | --- | --- |
|  | N | Percent |
| Included | 137 | 95.1% |
| Excluded | 7 | 4.9% |
| Total | 144 | 100.0% |

| **Correlated Data Summary** | | | |
| --- | --- | --- | --- |
| Number of Levels | Subject Effect | ID | 72 |
|  | Within-Subject Effect | TIME | 2 |
| Number of Subjects | | | 72 |
| Number of Measurements per Subject | Minimum | | 1 |
|  | Maximum | | 2 |
| Correlation Matrix Dimension | | | 2 |

| **Categorical Variable Information** | | | | |
| --- | --- | --- | --- | --- |
|  | | | N | Percent |
| Factor | TIME | 1.00 | 72 | 52.6% |
|  |  | 2.00 | 65 | 47.4% |
|  |  | Total | 137 | 100.0% |
|  | GROUP | LOW_INTENSITY | 33 | 24.1% |
|  |  | MODERATE_INTENSITY | 35 | 25.5% |
|  |  | HIGH_INTENSITY | 34 | 24.8% |
|  |  | CONTROL | 35 | 25.5% |
|  |  | Total | 137 | 100.0% |

| **Continuous Variable Information** | | | | | | |
| --- | --- | --- | --- | --- | --- | --- |
|  | | N | Minimum | Maximum | Mean | Std. Deviation |
| Dependent Variable | BMI | 137 | 23.77 | 33.82 | 28.1269 | 3.22188 |

| **Goodness of Fit^a^** | |
| --- | --- |
|  | Value |
| Quasi Likelihood under Independence Model Criterion (QIC)^b^ | 1382.945 |
| Corrected Quasi Likelihood under Independence Model Criterion (QICC)^b^ | 1382.809 |
| Dependent Variable: BMI  Model: (Intercept), TIME, GROUP, TIME * GROUP^a^ | |
| a. Information criteria are in smaller-is-better form. | |
| b. Computed using the full log quasi-likelihood function. | |

| **Tests of Model Effects** | | | |
| --- | --- | --- | --- |
| Source | Type III | | |
|  | Wald Chi-Square | df | Sig. |
| (Intercept) | 5725.399 | 1 | .000 |
| TIME | 17.685 | 1 | .000 |
| GROUP | 1.195 | 3 | .754 |
| TIME * GROUP | 8.248 | 3 | .041 |
| Dependent Variable: BMI  Model: (Intercept), TIME, GROUP, TIME * GROUP | | | |

| **Parameter Estimates** | | | | | | | |
| --- | --- | --- | --- | --- | --- | --- | --- |
| Parameter | B | Std. Error | 95% Wald Confidence Interval | | Hypothesis Test | | |
|  |  |  | Lower | Upper | Wald Chi-Square | df | Sig. |
| (Intercept) | 28.406 | .7558 | 26.924 | 29.887 | 1412.688 | 1 | .000 |
| [TIME=1.00] | .260 | .2038 | -.139 | .660 | 1.633 | 1 | .201 |
| [TIME=2.00] | 0^a^ | . | . | . | . | . | . |
| [GROUP=1.00] | .328 | 1.1118 | -1.851 | 2.507 | .087 | 1 | .768 |
| [GROUP=2.00] | -1.241 | 1.0599 | -3.318 | .836 | 1.371 | 1 | .242 |
| [GROUP=3.00] | -.623 | 1.0783 | -2.736 | 1.491 | .333 | 1 | .564 |
| [GROUP=4.00] | 0^a^ | . | . | . | . | . | . |
| [TIME=1.00] * [GROUP=1.00] | -.109 | .3374 | -.770 | .553 | .104 | 1 | .748 |
| [TIME=1.00] * [GROUP=2.00] | .826 | .3710 | .099 | 1.553 | 4.955 | 1 | .026 |
| [TIME=1.00] * [GROUP=3.00] | .707 | .4189 | -.114 | 1.528 | 2.848 | 1 | .091 |
| [TIME=1.00] * [GROUP=4.00] | 0^a^ | . | . | . | . | . | . |
| [TIME=2.00] * [GROUP=1.00] | 0^a^ | . | . | . | . | . | . |
| [TIME=2.00] * [GROUP=2.00] | 0^a^ | . | . | . | . | . | . |
| [TIME=2.00] * [GROUP=3.00] | 0^a^ | . | . | . | . | . | . |
| [TIME=2.00] * [GROUP=4.00] | 0^a^ | . | . | . | . | . | . |
| (Scale) | 10.595 |  |  |  |  |  |  |
| Dependent Variable: BMI  Model: (Intercept), TIME, GROUP, TIME * GROUP | | | | | | | |
| a. Set to zero because this parameter is redundant. | | | | | | | |

**Estimated Marginal Means: TIME* GROUP**

| **Estimates** | | | | | |
| --- | --- | --- | --- | --- | --- |
| TIME | GROUP | Mean | Std. Error | 95% Wald Confidence Interval | |
|  |  |  |  | Lower | Upper |
| 1.00 | LOW_INTENSITY | 28.8856 | .76159 | 27.3929 | 30.3783 |
|  | MODERATE_INTENSITY | 28.2511 | .74141 | 26.7980 | 29.7042 |
|  | HIGH_INTENSITY | 28.7506 | .75914 | 27.2627 | 30.2384 |
|  | CONTROL | 28.6661 | .75622 | 27.1839 | 30.1483 |
| 2.00 | LOW_INTENSITY | 28.7337 | .81543 | 27.1354 | 30.3319 |
|  | MODERATE_INTENSITY | 27.1648 | .74309 | 25.7083 | 28.6212 |
|  | HIGH_INTENSITY | 27.7831 | .76915 | 26.2756 | 29.2906 |
|  | CONTROL | 28.4057 | .75576 | 26.9244 | 29.8869 |

| **Pairwise Comparisons** | | | | | | | |
| --- | --- | --- | --- | --- | --- | --- | --- |
| (I) TIME*GROUP | (J) TIME*GROUP | Mean Difference (I-J) | Std. Error | df | Sequential Bonferroni Sig. | 95% Wald Confidence Interval for Difference^a^ | |
|  |  |  |  |  |  | Lower | Upper |
| [TIME=1.00]*[GROUP=1.00] | [TIME=1.00]*[GROUP=2.00] | .6344 | 1.06288 | 1 | 1.000 | -1.7081 | 2.9770 |
|  | [TIME=1.00]*[GROUP=3.00] | .1350 | 1.07532 | 1 | 1.000 | -2.0206 | 2.2906 |
|  | [TIME=1.00]*[GROUP=4.00] | .2194 | 1.07326 | 1 | 1.000 | -1.9641 | 2.4030 |
|  | [TIME=2.00]*[GROUP=1.00] | .1519 | .26890 | 1 | 1.000 | -.4367 | .7405 |
|  | [TIME=2.00]*[GROUP=2.00] | 1.7208 | 1.06405 | 1 | 1.000 | -1.5797 | 5.0214 |
|  | [TIME=2.00]*[GROUP=3.00] | 1.1024 | 1.08241 | 1 | 1.000 | -1.5195 | 3.7244 |
|  | [TIME=2.00]*[GROUP=4.00] | .4799 | 1.07294 | 1 | 1.000 | -1.8112 | 2.7710 |
| [TIME=1.00]*[GROUP=2.00] | [TIME=1.00]*[GROUP=1.00] | -.6344 | 1.06288 | 1 | 1.000 | -2.9770 | 1.7081 |
|  | [TIME=1.00]*[GROUP=3.00] | -.4994 | 1.06112 | 1 | 1.000 | -2.7763 | 1.7775 |
|  | [TIME=1.00]*[GROUP=4.00] | -.4150 | 1.05904 | 1 | 1.000 | -2.6508 | 1.8208 |
|  | [TIME=2.00]*[GROUP=1.00] | -.4825 | 1.10210 | 1 | 1.000 | -2.8313 | 1.8662 |
|  | [TIME=2.00]*[GROUP=2.00] | 1.0864^b^ | .31004 | 1 | .013 | .1179 | 2.0548 |
|  | [TIME=2.00]*[GROUP=3.00] | .4680 | 1.06830 | 1 | 1.000 | -1.8088 | 2.7449 |
|  | [TIME=2.00]*[GROUP=4.00] | -.1545 | 1.05870 | 1 | 1.000 | -2.2849 | 1.9758 |
| [TIME=1.00]*[GROUP=3.00] | [TIME=1.00]*[GROUP=1.00] | -.1350 | 1.07532 | 1 | 1.000 | -2.2906 | 2.0206 |
|  | [TIME=1.00]*[GROUP=2.00] | .4994 | 1.06112 | 1 | 1.000 | -1.7775 | 2.7763 |
|  | [TIME=1.00]*[GROUP=4.00] | .0844 | 1.07153 | 1 | 1.000 | -2.0453 | 2.2142 |
|  | [TIME=2.00]*[GROUP=1.00] | .0169 | 1.11410 | 1 | 1.000 | -2.1725 | 2.2063 |
|  | [TIME=2.00]*[GROUP=2.00] | 1.5858 | 1.06230 | 1 | 1.000 | -1.2906 | 4.4622 |
|  | [TIME=2.00]*[GROUP=3.00] | .9674 | .36598 | 1 | .222 | -.1719 | 2.1068 |
|  | [TIME=2.00]*[GROUP=4.00] | .3449 | 1.07120 | 1 | 1.000 | -1.8850 | 2.5748 |
| [TIME=1.00]*[GROUP=4.00] | [TIME=1.00]*[GROUP=1.00] | -.2194 | 1.07326 | 1 | 1.000 | -2.4030 | 1.9641 |
|  | [TIME=1.00]*[GROUP=2.00] | .4150 | 1.05904 | 1 | 1.000 | -1.8208 | 2.6508 |
|  | [TIME=1.00]*[GROUP=3.00] | -.0844 | 1.07153 | 1 | 1.000 | -2.2142 | 2.0453 |
|  | [TIME=2.00]*[GROUP=1.00] | -.0675 | 1.11211 | 1 | 1.000 | -2.2708 | 2.1357 |
|  | [TIME=2.00]*[GROUP=2.00] | 1.5014 | 1.06022 | 1 | 1.000 | -1.3177 | 4.3205 |
|  | [TIME=2.00]*[GROUP=3.00] | .8830 | 1.07864 | 1 | 1.000 | -1.6133 | 3.3793 |
|  | [TIME=2.00]*[GROUP=4.00] | .2605 | .20381 | 1 | 1.000 | -.2641 | .7850 |
| [TIME=2.00]*[GROUP=1.00] | [TIME=1.00]*[GROUP=1.00] | -.1519 | .26890 | 1 | 1.000 | -.7405 | .4367 |
|  | [TIME=1.00]*[GROUP=2.00] | .4825 | 1.10210 | 1 | 1.000 | -1.8662 | 2.8313 |
|  | [TIME=1.00]*[GROUP=3.00] | -.0169 | 1.11410 | 1 | 1.000 | -2.2063 | 2.1725 |
|  | [TIME=1.00]*[GROUP=4.00] | .0675 | 1.11211 | 1 | 1.000 | -2.1357 | 2.2708 |
|  | [TIME=2.00]*[GROUP=2.00] | 1.5689 | 1.10323 | 1 | 1.000 | -1.3687 | 4.5066 |
|  | [TIME=2.00]*[GROUP=3.00] | .9506 | 1.12094 | 1 | 1.000 | -1.6609 | 3.5620 |
|  | [TIME=2.00]*[GROUP=4.00] | .3280 | 1.11180 | 1 | 1.000 | -1.9741 | 2.6301 |
| [TIME=2.00]*[GROUP=2.00] | [TIME=1.00]*[GROUP=1.00] | -1.7208 | 1.06405 | 1 | 1.000 | -5.0214 | 1.5797 |
|  | [TIME=1.00]*[GROUP=2.00] | -1.0864^b^ | .31004 | 1 | .013 | -2.0548 | -.1179 |
|  | [TIME=1.00]*[GROUP=3.00] | -1.5858 | 1.06230 | 1 | 1.000 | -4.4622 | 1.2906 |
|  | [TIME=1.00]*[GROUP=4.00] | -1.5014 | 1.06022 | 1 | 1.000 | -4.3205 | 1.3177 |
|  | [TIME=2.00]*[GROUP=1.00] | -1.5689 | 1.10323 | 1 | 1.000 | -4.5066 | 1.3687 |
|  | [TIME=2.00]*[GROUP=3.00] | -.6184 | 1.06947 | 1 | 1.000 | -2.9660 | 1.7292 |
|  | [TIME=2.00]*[GROUP=4.00] | -1.2409 | 1.05988 | 1 | 1.000 | -3.9009 | 1.4191 |
| [TIME=2.00]*[GROUP=3.00] | [TIME=1.00]*[GROUP=1.00] | -1.1024 | 1.08241 | 1 | 1.000 | -3.7244 | 1.5195 |
|  | [TIME=1.00]*[GROUP=2.00] | -.4680 | 1.06830 | 1 | 1.000 | -2.7449 | 1.8088 |
|  | [TIME=1.00]*[GROUP=3.00] | -.9674 | .36598 | 1 | .222 | -2.1068 | .1719 |
|  | [TIME=1.00]*[GROUP=4.00] | -.8830 | 1.07864 | 1 | 1.000 | -3.3793 | 1.6133 |
|  | [TIME=2.00]*[GROUP=1.00] | -.9506 | 1.12094 | 1 | 1.000 | -3.5620 | 1.6609 |
|  | [TIME=2.00]*[GROUP=2.00] | .6184 | 1.06947 | 1 | 1.000 | -1.7292 | 2.9660 |
|  | [TIME=2.00]*[GROUP=4.00] | -.6226 | 1.07831 | 1 | 1.000 | -2.9891 | 1.7440 |
| [TIME=2.00]*[GROUP=4.00] | [TIME=1.00]*[GROUP=1.00] | -.4799 | 1.07294 | 1 | 1.000 | -2.7710 | 1.8112 |
|  | [TIME=1.00]*[GROUP=2.00] | .1545 | 1.05870 | 1 | 1.000 | -1.9758 | 2.2849 |
|  | [TIME=1.00]*[GROUP=3.00] | -.3449 | 1.07120 | 1 | 1.000 | -2.5748 | 1.8850 |
|  | [TIME=1.00]*[GROUP=4.00] | -.2605 | .20381 | 1 | 1.000 | -.7850 | .2641 |
|  | [TIME=2.00]*[GROUP=1.00] | -.3280 | 1.11180 | 1 | 1.000 | -2.6301 | 1.9741 |
|  | [TIME=2.00]*[GROUP=2.00] | 1.2409 | 1.05988 | 1 | 1.000 | -1.4191 | 3.9009 |
|  | [TIME=2.00]*[GROUP=3.00] | .6226 | 1.07831 | 1 | 1.000 | -1.7440 | 2.9891 |
| Pairwise comparisons of estimated marginal means based on the original scale of dependent variable BMI | | | | | | | |
| a. Confidence interval bounds are approximate. | | | | | | | |
| b. The mean difference is significant at the .05 level. | | | | | | | |

| **Overall Test Results** | | |
| --- | --- | --- |
| Wald Chi-Square | df | Sig. |
| 22.547 | 7 | .002 |
| The Wald chi-square tests the effect of TIME*GROUP. This test is based on the linearly independent pairwise comparisons among the estimated marginal means. | | |

* Generalized Estimating Equations.

GENLIN Body_fat_percentage BY TIME GROUP (ORDER=ASCENDING)

/MODEL TIME GROUP TIME*GROUP INTERCEPT=YES

DISTRIBUTION=NORMAL LINK=IDENTITY

/CRITERIA SCALE=MLE PCONVERGE=1E-006(ABSOLUTE) SINGULAR=1E-012 ANALYSISTYPE=3(WALD) CILEVEL=95

LIKELIHOOD=FULL

/EMMEANS TABLES=TIME*GROUP SCALE=ORIGINAL COMPARE=TIME*GROUP CONTRAST=PAIRWISE

PADJUST=SEQBONFERRONI

/REPEATED SUBJECT=ID WITHINSUBJECT=TIME SORT=YES CORRTYPE=AR(1) ADJUSTCORR=YES COVB=ROBUST

MAXITERATIONS=100 PCONVERGE=1e-006(ABSOLUTE) UPDATECORR=1

/MISSING CLASSMISSING=EXCLUDE

/PRINT CPS DESCRIPTIVES MODELINFO FIT SUMMARY SOLUTION.

**Generalized Linear Models**

| **Notes** | | |
| --- | --- | --- |
| Output Created | | 09-JUN-2023 00:04:40 |
| Comments | |  |
| Input | Data | C:\Users\mdanaee\Google Drive\Data Analysis\Prof SOh students\Prof Soh -Wang\DATA GEEcr.sav |
|  | Active Dataset | DataSet1 |
|  | Filter | <none> |
|  | Weight | <none> |
|  | Split File | <none> |
|  | N of Rows in Working Data File | 144 |
| Missing Value Handling | Definition of Missing | User-defined missing values for factor, subject and within-subject variables are treated as missing. |
|  | Cases Used | Statistics are based on cases with valid data for all variables in the model. |
| Weight Handling | | not applicable |
| Syntax | | GENLIN Body_fat_percentage BY TIME GROUP (ORDER=ASCENDING)  /MODEL TIME GROUP TIME*GROUP INTERCEPT=YES  DISTRIBUTION=NORMAL LINK=IDENTITY  /CRITERIA SCALE=MLE PCONVERGE=1E-006(ABSOLUTE) SINGULAR=1E-012 ANALYSISTYPE=3(WALD) CILEVEL=95  LIKELIHOOD=FULL  /EMMEANS TABLES=TIME*GROUP SCALE=ORIGINAL COMPARE=TIME*GROUP CONTRAST=PAIRWISE  PADJUST=SEQBONFERRONI  /REPEATED SUBJECT=ID WITHINSUBJECT=TIME SORT=YES CORRTYPE=AR(1) ADJUSTCORR=YES COVB=ROBUST  MAXITERATIONS=100 PCONVERGE=1e-006(ABSOLUTE) UPDATECORR=1  /MISSING CLASSMISSING=EXCLUDE  /PRINT CPS DESCRIPTIVES MODELINFO FIT SUMMARY SOLUTION. |
| Resources | Processor Time | 00:00:00.31 |
|  | Elapsed Time | 00:00:00.29 |

| **Model Information** | | |
| --- | --- | --- |
| Dependent Variable | | Body_fat_percentage |
| Probability Distribution | | Normal |
| Link Function | | Identity |
| Subject Effect | 1 | ID |
| Within-Subject Effect | 1 | TIME |
| Working Correlation Matrix Structure | | AR(1) |

| **Case Processing Summary** | | |
| --- | --- | --- |
|  | N | Percent |
| Included | 137 | 95.1% |
| Excluded | 7 | 4.9% |
| Total | 144 | 100.0% |

| **Correlated Data Summary** | | | |
| --- | --- | --- | --- |
| Number of Levels | Subject Effect | ID | 72 |
|  | Within-Subject Effect | TIME | 2 |
| Number of Subjects | | | 72 |
| Number of Measurements per Subject | Minimum | | 1 |
|  | Maximum | | 2 |
| Correlation Matrix Dimension | | | 2 |

| **Categorical Variable Information** | | | | |
| --- | --- | --- | --- | --- |
|  | | | N | Percent |
| Factor | TIME | 1.00 | 72 | 52.6% |
|  |  | 2.00 | 65 | 47.4% |
|  |  | Total | 137 | 100.0% |
|  | GROUP | LOW_INTENSITY | 33 | 24.1% |
|  |  | MODERATE_INTENSITY | 35 | 25.5% |
|  |  | HIGH_INTENSITY | 34 | 24.8% |
|  |  | CONTROL | 35 | 25.5% |
|  |  | Total | 137 | 100.0% |

| **Continuous Variable Information** | | | | | | |
| --- | --- | --- | --- | --- | --- | --- |
|  | | N | Minimum | Maximum | Mean | Std. Deviation |
| Dependent Variable | Body_fat_percentage | 137 | 18.39 | 33.46 | 26.6031 | 3.42564 |

| **Goodness of Fit^a^** | |
| --- | --- |
|  | Value |
| Quasi Likelihood under Independence Model Criterion (QIC)^b^ | 1276.752 |
| Corrected Quasi Likelihood under Independence Model Criterion (QICC)^b^ | 1276.239 |
| Dependent Variable: Body_fat_percentage  Model: (Intercept), TIME, GROUP, TIME * GROUP^a^ | |
| a. Information criteria are in smaller-is-better form. | |
| b. Computed using the full log quasi-likelihood function. | |

| **Tests of Model Effects** | | | |
| --- | --- | --- | --- |
| Source | Type III | | |
|  | Wald Chi-Square | df | Sig. |
| (Intercept) | 5338.056 | 1 | .000 |
| TIME | 489.104 | 1 | .000 |
| GROUP | 4.751 | 3 | .191 |
| TIME * GROUP | 123.592 | 3 | .000 |
| Dependent Variable: Body_fat_percentage  Model: (Intercept), TIME, GROUP, TIME * GROUP | | | |

| **Parameter Estimates** | | | | | | | |
| --- | --- | --- | --- | --- | --- | --- | --- |
| Parameter | B | Std. Error | 95% Wald Confidence Interval | | Hypothesis Test | | |
|  |  |  | Lower | Upper | Wald Chi-Square | df | Sig. |
| (Intercept) | 26.906 | .7463 | 25.444 | 28.369 | 1299.670 | 1 | .000 |
| [TIME=1.00] | .974 | .1878 | .606 | 1.342 | 26.875 | 1 | .000 |
| [TIME=2.00] | 0^a^ | . | . | . | . | . | . |
| [GROUP=1.00] | .090 | 1.1110 | -2.088 | 2.267 | .007 | 1 | .936 |
| [GROUP=2.00] | -1.647 | 1.0232 | -3.653 | .358 | 2.593 | 1 | .107 |
| [GROUP=3.00] | -3.222 | .9986 | -5.179 | -1.264 | 10.408 | 1 | .001 |
| [GROUP=4.00] | 0^a^ | . | . | . | . | . | . |
| [TIME=1.00] * [GROUP=1.00] | .290 | .2795 | -.258 | .838 | 1.078 | 1 | .299 |
| [TIME=1.00] * [GROUP=2.00] | 1.467 | .2427 | .992 | 1.943 | 36.566 | 1 | .000 |
| [TIME=1.00] * [GROUP=3.00] | 2.922 | .2895 | 2.354 | 3.489 | 101.824 | 1 | .000 |
| [TIME=1.00] * [GROUP=4.00] | 0^a^ | . | . | . | . | . | . |
| [TIME=2.00] * [GROUP=1.00] | 0^a^ | . | . | . | . | . | . |
| [TIME=2.00] * [GROUP=2.00] | 0^a^ | . | . | . | . | . | . |
| [TIME=2.00] * [GROUP=3.00] | 0^a^ | . | . | . | . | . | . |
| [TIME=2.00] * [GROUP=4.00] | 0^a^ | . | . | . | . | . | . |
| (Scale) | 9.769 |  |  |  |  |  |  |
| Dependent Variable: Body_fat_percentage  Model: (Intercept), TIME, GROUP, TIME * GROUP | | | | | | | |
| a. Set to zero because this parameter is redundant. | | | | | | | |

**Estimated Marginal Means: TIME* GROUP**

| **Estimates** | | | | | |
| --- | --- | --- | --- | --- | --- |
| TIME | GROUP | Mean | Std. Error | 95% Wald Confidence Interval | |
|  |  |  |  | Lower | Upper |
| 1.00 | LOW_INTENSITY | 28.2600 | .75681 | 26.7767 | 29.7433 |
|  | MODERATE_INTENSITY | 27.7000 | .73384 | 26.2617 | 29.1383 |
|  | HIGH_INTENSITY | 27.5800 | .73647 | 26.1365 | 29.0235 |
|  | CONTROL | 27.8800 | .74661 | 26.4167 | 29.3433 |
| 2.00 | LOW_INTENSITY | 26.9963 | .82299 | 25.3832 | 28.6093 |
|  | MODERATE_INTENSITY | 25.2589 | .69992 | 23.8871 | 26.6307 |
|  | HIGH_INTENSITY | 23.6849 | .66339 | 22.3846 | 24.9851 |
|  | CONTROL | 26.9064 | .74634 | 25.4436 | 28.3692 |

| **Pairwise Comparisons** | | | | | | | |
| --- | --- | --- | --- | --- | --- | --- | --- |
| (I) TIME*GROUP | (J) TIME*GROUP | Mean Difference (I-J) | Std. Error | df | Sequential Bonferroni Sig. | 95% Wald Confidence Interval for Difference^a^ | |
|  |  |  |  |  |  | Lower | Upper |
| [TIME=1.00]*[GROUP=1.00] | [TIME=1.00]*[GROUP=2.00] | .5600 | 1.05417 | 1 | 1.000 | -1.7310 | 2.8510 |
|  | [TIME=1.00]*[GROUP=3.00] | .6800 | 1.05601 | 1 | 1.000 | -1.6712 | 3.0312 |
|  | [TIME=1.00]*[GROUP=4.00] | .3800 | 1.06310 | 1 | 1.000 | -1.8488 | 2.6088 |
|  | [TIME=2.00]*[GROUP=1.00] | 1.2637^b^ | .20697 | 1 | .000 | .6217 | 1.9057 |
|  | [TIME=2.00]*[GROUP=2.00] | 3.0011 | 1.03085 | 1 | .068 | -.0995 | 6.1017 |
|  | [TIME=2.00]*[GROUP=3.00] | 4.5751^b^ | 1.00640 | 1 | .000 | 1.4774 | 7.6729 |
|  | [TIME=2.00]*[GROUP=4.00] | 1.3536 | 1.06291 | 1 | 1.000 | -1.3791 | 4.0863 |
| [TIME=1.00]*[GROUP=2.00] | [TIME=1.00]*[GROUP=1.00] | -.5600 | 1.05417 | 1 | 1.000 | -2.8510 | 1.7310 |
|  | [TIME=1.00]*[GROUP=3.00] | .1200 | 1.03967 | 1 | 1.000 | -1.9603 | 2.2003 |
|  | [TIME=1.00]*[GROUP=4.00] | -.1800 | 1.04688 | 1 | 1.000 | -2.2968 | 1.9368 |
|  | [TIME=2.00]*[GROUP=1.00] | .7037 | 1.10265 | 1 | 1.000 | -1.7483 | 3.1557 |
|  | [TIME=2.00]*[GROUP=2.00] | 2.4411^b^ | .15370 | 1 | .000 | 1.9610 | 2.9212 |
|  | [TIME=2.00]*[GROUP=3.00] | 4.0151^b^ | .98924 | 1 | .001 | .9959 | 7.0344 |
|  | [TIME=2.00]*[GROUP=4.00] | .7936 | 1.04669 | 1 | 1.000 | -1.5962 | 3.1834 |
| [TIME=1.00]*[GROUP=3.00] | [TIME=1.00]*[GROUP=1.00] | -.6800 | 1.05601 | 1 | 1.000 | -3.0312 | 1.6712 |
|  | [TIME=1.00]*[GROUP=2.00] | -.1200 | 1.03967 | 1 | 1.000 | -2.2003 | 1.9603 |
|  | [TIME=1.00]*[GROUP=4.00] | -.3000 | 1.04872 | 1 | 1.000 | -2.4676 | 1.8676 |
|  | [TIME=2.00]*[GROUP=1.00] | .5837 | 1.10440 | 1 | 1.000 | -1.8150 | 2.9825 |
|  | [TIME=2.00]*[GROUP=2.00] | 2.3211 | 1.01601 | 1 | .380 | -.7004 | 5.3425 |
|  | [TIME=2.00]*[GROUP=3.00] | 3.8951^b^ | .22035 | 1 | .000 | 3.2092 | 4.5811 |
|  | [TIME=2.00]*[GROUP=4.00] | .6736 | 1.04853 | 1 | 1.000 | -1.6602 | 3.0074 |
| [TIME=1.00]*[GROUP=4.00] | [TIME=1.00]*[GROUP=1.00] | -.3800 | 1.06310 | 1 | 1.000 | -2.6088 | 1.8488 |
|  | [TIME=1.00]*[GROUP=2.00] | .1800 | 1.04688 | 1 | 1.000 | -1.9368 | 2.2968 |
|  | [TIME=1.00]*[GROUP=3.00] | .3000 | 1.04872 | 1 | 1.000 | -1.8676 | 2.4676 |
|  | [TIME=2.00]*[GROUP=1.00] | .8837 | 1.11119 | 1 | 1.000 | -1.6745 | 3.4419 |
|  | [TIME=2.00]*[GROUP=2.00] | 2.6211 | 1.02339 | 1 | .188 | -.4402 | 5.6824 |
|  | [TIME=2.00]*[GROUP=3.00] | 4.1951^b^ | .99875 | 1 | .001 | 1.1336 | 7.2567 |
|  | [TIME=2.00]*[GROUP=4.00] | .9736^b^ | .18780 | 1 | .000 | .3932 | 1.5540 |
| [TIME=2.00]*[GROUP=1.00] | [TIME=1.00]*[GROUP=1.00] | -1.2637^b^ | .20697 | 1 | .000 | -1.9057 | -.6217 |
|  | [TIME=1.00]*[GROUP=2.00] | -.7037 | 1.10265 | 1 | 1.000 | -3.1557 | 1.7483 |
|  | [TIME=1.00]*[GROUP=3.00] | -.5837 | 1.10440 | 1 | 1.000 | -2.9825 | 1.8150 |
|  | [TIME=1.00]*[GROUP=4.00] | -.8837 | 1.11119 | 1 | 1.000 | -3.4419 | 1.6745 |
|  | [TIME=2.00]*[GROUP=2.00] | 1.7374 | 1.08037 | 1 | 1.000 | -1.2690 | 4.7437 |
|  | [TIME=2.00]*[GROUP=3.00] | 3.3114^b^ | 1.05707 | 1 | .035 | .1155 | 6.5073 |
|  | [TIME=2.00]*[GROUP=4.00] | .0899 | 1.11101 | 1 | 1.000 | -2.1192 | 2.2989 |
| [TIME=2.00]*[GROUP=2.00] | [TIME=1.00]*[GROUP=1.00] | -3.0011 | 1.03085 | 1 | .068 | -6.1017 | .0995 |
|  | [TIME=1.00]*[GROUP=2.00] | -2.4411^b^ | .15370 | 1 | .000 | -2.9212 | -1.9610 |
|  | [TIME=1.00]*[GROUP=3.00] | -2.3211 | 1.01601 | 1 | .380 | -5.3425 | .7004 |
|  | [TIME=1.00]*[GROUP=4.00] | -2.6211 | 1.02339 | 1 | .188 | -5.6824 | .4402 |
|  | [TIME=2.00]*[GROUP=1.00] | -1.7374 | 1.08037 | 1 | 1.000 | -4.7437 | 1.2690 |
|  | [TIME=2.00]*[GROUP=3.00] | 1.5740 | .96435 | 1 | 1.000 | -1.2758 | 4.4239 |
|  | [TIME=2.00]*[GROUP=4.00] | -1.6475 | 1.02319 | 1 | 1.000 | -4.4961 | 1.2011 |
| [TIME=2.00]*[GROUP=3.00] | [TIME=1.00]*[GROUP=1.00] | -4.5751^b^ | 1.00640 | 1 | .000 | -7.6729 | -1.4774 |
|  | [TIME=1.00]*[GROUP=2.00] | -4.0151^b^ | .98924 | 1 | .001 | -7.0344 | -.9959 |
|  | [TIME=1.00]*[GROUP=3.00] | -3.8951^b^ | .22035 | 1 | .000 | -4.5811 | -3.2092 |
|  | [TIME=1.00]*[GROUP=4.00] | -4.1951^b^ | .99875 | 1 | .001 | -7.2567 | -1.1336 |
|  | [TIME=2.00]*[GROUP=1.00] | -3.3114^b^ | 1.05707 | 1 | .035 | -6.5073 | -.1155 |
|  | [TIME=2.00]*[GROUP=2.00] | -1.5740 | .96435 | 1 | 1.000 | -4.4239 | 1.2758 |
|  | [TIME=2.00]*[GROUP=4.00] | -3.2215^b^ | .99855 | 1 | .026 | -6.2552 | -.1879 |
| [TIME=2.00]*[GROUP=4.00] | [TIME=1.00]*[GROUP=1.00] | -1.3536 | 1.06291 | 1 | 1.000 | -4.0863 | 1.3791 |
|  | [TIME=1.00]*[GROUP=2.00] | -.7936 | 1.04669 | 1 | 1.000 | -3.1834 | 1.5962 |
|  | [TIME=1.00]*[GROUP=3.00] | -.6736 | 1.04853 | 1 | 1.000 | -3.0074 | 1.6602 |
|  | [TIME=1.00]*[GROUP=4.00] | -.9736^b^ | .18780 | 1 | .000 | -1.5540 | -.3932 |
|  | [TIME=2.00]*[GROUP=1.00] | -.0899 | 1.11101 | 1 | 1.000 | -2.2989 | 2.1192 |
|  | [TIME=2.00]*[GROUP=2.00] | 1.6475 | 1.02319 | 1 | 1.000 | -1.2011 | 4.4961 |
|  | [TIME=2.00]*[GROUP=3.00] | 3.2215^b^ | .99855 | 1 | .026 | .1879 | 6.2552 |
| Pairwise comparisons of estimated marginal means based on the original scale of dependent variable Body_fat_percentage | | | | | | | |
| a. Confidence interval bounds are approximate. | | | | | | | |
| b. The mean difference is significant at the .05 level. | | | | | | | |

| **Overall Test Results** | | |
| --- | --- | --- |
| Wald Chi-Square | df | Sig. |
| 701.062 | 7 | .000 |
| The Wald chi-square tests the effect of TIME*GROUP. This test is based on the linearly independent pairwise comparisons among the estimated marginal means. | | |

* Generalized Estimating Equations.

GENLIN waist_circumstance BY TIME GROUP (ORDER=ASCENDING)

/MODEL TIME GROUP TIME*GROUP INTERCEPT=YES

DISTRIBUTION=NORMAL LINK=IDENTITY

/CRITERIA SCALE=MLE PCONVERGE=1E-006(ABSOLUTE) SINGULAR=1E-012 ANALYSISTYPE=3(WALD) CILEVEL=95

LIKELIHOOD=FULL

/EMMEANS TABLES=TIME*GROUP SCALE=ORIGINAL COMPARE=TIME*GROUP CONTRAST=PAIRWISE

PADJUST=SEQBONFERRONI

/REPEATED SUBJECT=ID WITHINSUBJECT=TIME SORT=YES CORRTYPE=AR(1) ADJUSTCORR=YES COVB=ROBUST

MAXITERATIONS=100 PCONVERGE=1e-006(ABSOLUTE) UPDATECORR=1

/MISSING CLASSMISSING=EXCLUDE

/PRINT CPS DESCRIPTIVES MODELINFO FIT SUMMARY SOLUTION.

**Generalized Linear Models**

| **Notes** | | |
| --- | --- | --- |
| Output Created | | 09-JUN-2023 00:04:40 |
| Comments | |  |
| Input | Data | C:\Users\mdanaee\Google Drive\Data Analysis\Prof SOh students\Prof Soh -Wang\DATA GEEcr.sav |
|  | Active Dataset | DataSet1 |
|  | Filter | <none> |
|  | Weight | <none> |
|  | Split File | <none> |
|  | N of Rows in Working Data File | 144 |
| Missing Value Handling | Definition of Missing | User-defined missing values for factor, subject and within-subject variables are treated as missing. |
|  | Cases Used | Statistics are based on cases with valid data for all variables in the model. |
| Weight Handling | | not applicable |
| Syntax | | GENLIN waist_circumstance BY TIME GROUP (ORDER=ASCENDING)  /MODEL TIME GROUP TIME*GROUP INTERCEPT=YES  DISTRIBUTION=NORMAL LINK=IDENTITY  /CRITERIA SCALE=MLE PCONVERGE=1E-006(ABSOLUTE) SINGULAR=1E-012 ANALYSISTYPE=3(WALD) CILEVEL=95  LIKELIHOOD=FULL  /EMMEANS TABLES=TIME*GROUP SCALE=ORIGINAL COMPARE=TIME*GROUP CONTRAST=PAIRWISE  PADJUST=SEQBONFERRONI  /REPEATED SUBJECT=ID WITHINSUBJECT=TIME SORT=YES CORRTYPE=AR(1) ADJUSTCORR=YES COVB=ROBUST  MAXITERATIONS=100 PCONVERGE=1e-006(ABSOLUTE) UPDATECORR=1  /MISSING CLASSMISSING=EXCLUDE  /PRINT CPS DESCRIPTIVES MODELINFO FIT SUMMARY SOLUTION. |
| Resources | Processor Time | 00:00:00.19 |
|  | Elapsed Time | 00:00:00.14 |

| **Model Information** | | |
| --- | --- | --- |
| Dependent Variable | | waist_circumstance |
| Probability Distribution | | Normal |
| Link Function | | Identity |
| Subject Effect | 1 | ID |
| Within-Subject Effect | 1 | TIME |
| Working Correlation Matrix Structure | | AR(1) |

| **Case Processing Summary** | | |
| --- | --- | --- |
|  | N | Percent |
| Included | 137 | 95.1% |
| Excluded | 7 | 4.9% |
| Total | 144 | 100.0% |

| **Correlated Data Summary** | | | |
| --- | --- | --- | --- |
| Number of Levels | Subject Effect | ID | 72 |
|  | Within-Subject Effect | TIME | 2 |
| Number of Subjects | | | 72 |
| Number of Measurements per Subject | Minimum | | 1 |
|  | Maximum | | 2 |
| Correlation Matrix Dimension | | | 2 |

| **Categorical Variable Information** | | | | |
| --- | --- | --- | --- | --- |
|  | | | N | Percent |
| Factor | TIME | 1.00 | 72 | 52.6% |
|  |  | 2.00 | 65 | 47.4% |
|  |  | Total | 137 | 100.0% |
|  | GROUP | LOW_INTENSITY | 33 | 24.1% |
|  |  | MODERATE_INTENSITY | 35 | 25.5% |
|  |  | HIGH_INTENSITY | 34 | 24.8% |
|  |  | CONTROL | 35 | 25.5% |
|  |  | Total | 137 | 100.0% |

| **Continuous Variable Information** | | | | | | |
| --- | --- | --- | --- | --- | --- | --- |
|  | | N | Minimum | Maximum | Mean | Std. Deviation |
| Dependent Variable | waist_circumstance | 137 | 80.1 | 100.4 | 91.002 | 5.1166 |

| **Goodness of Fit^a^** | |
| --- | --- |
|  | Value |
| Quasi Likelihood under Independence Model Criterion (QIC)^b^ | 2899.273 |
| Corrected Quasi Likelihood under Independence Model Criterion (QICC)^b^ | 2898.972 |
| Dependent Variable: waist_circumstance  Model: (Intercept), TIME, GROUP, TIME * GROUP^a^ | |
| a. Information criteria are in smaller-is-better form. | |
| b. Computed using the full log quasi-likelihood function. | |

| **Tests of Model Effects** | | | |
| --- | --- | --- | --- |
| Source | Type III | | |
|  | Wald Chi-Square | df | Sig. |
| (Intercept) | 27137.204 | 1 | .000 |
| TIME | 1020.476 | 1 | .000 |
| GROUP | 3.224 | 3 | .358 |
| TIME * GROUP | 381.456 | 3 | .000 |
| Dependent Variable: waist_circumstance  Model: (Intercept), TIME, GROUP, TIME * GROUP | | | |

| **Parameter Estimates** | | | | | | | |
| --- | --- | --- | --- | --- | --- | --- | --- |
| Parameter | B | Std. Error | 95% Wald Confidence Interval | | Hypothesis Test | | |
|  |  |  | Lower | Upper | Wald Chi-Square | df | Sig. |
| (Intercept) | 91.524 | 1.1230 | 89.323 | 93.725 | 6642.437 | 1 | .000 |
| [TIME=1.00] | 1.426 | .1488 | 1.135 | 1.718 | 91.914 | 1 | .000 |
| [TIME=2.00] | 0^a^ | . | . | . | . | . | . |
| [GROUP=1.00] | -.175 | 1.6520 | -3.413 | 3.062 | .011 | 1 | .915 |
| [GROUP=2.00] | -3.000 | 1.5447 | -6.027 | .028 | 3.772 | 1 | .052 |
| [GROUP=3.00] | -4.126 | 1.5621 | -7.188 | -1.065 | 6.978 | 1 | .008 |
| [GROUP=4.00] | 0^a^ | . | . | . | . | . | . |
| [TIME=1.00] * [GROUP=1.00] | .442 | .2810 | -.109 | .993 | 2.474 | 1 | .116 |
| [TIME=1.00] * [GROUP=2.00] | 2.300 | .2659 | 1.779 | 2.821 | 74.806 | 1 | .000 |
| [TIME=1.00] * [GROUP=3.00] | 4.104 | .2212 | 3.671 | 4.538 | 344.279 | 1 | .000 |
| [TIME=1.00] * [GROUP=4.00] | 0^a^ | . | . | . | . | . | . |
| [TIME=2.00] * [GROUP=1.00] | 0^a^ | . | . | . | . | . | . |
| [TIME=2.00] * [GROUP=2.00] | 0^a^ | . | . | . | . | . | . |
| [TIME=2.00] * [GROUP=3.00] | 0^a^ | . | . | . | . | . | . |
| [TIME=2.00] * [GROUP=4.00] | 0^a^ | . | . | . | . | . | . |
| (Scale) | 22.349 |  |  |  |  |  |  |
| Dependent Variable: waist_circumstance  Model: (Intercept), TIME, GROUP, TIME * GROUP | | | | | | | |
| a. Set to zero because this parameter is redundant. | | | | | | | |

**Estimated Marginal Means: TIME* GROUP**

| **Estimates** | | | | | |
| --- | --- | --- | --- | --- | --- |
| TIME | GROUP | Mean | Std. Error | 95% Wald Confidence Interval | |
|  |  |  |  | Lower | Upper |
| 1.00 | LOW_INTENSITY | 93.217 | 1.1113 | 91.039 | 95.395 |
|  | MODERATE_INTENSITY | 92.250 | 1.0921 | 90.110 | 94.390 |
|  | HIGH_INTENSITY | 92.928 | 1.1054 | 90.761 | 95.094 |
|  | CONTROL | 92.950 | 1.1033 | 90.788 | 95.112 |
| 2.00 | LOW_INTENSITY | 91.348 | 1.2116 | 88.974 | 93.723 |
|  | MODERATE_INTENSITY | 88.524 | 1.0606 | 86.445 | 90.603 |
|  | HIGH_INTENSITY | 87.398 | 1.0858 | 85.269 | 89.526 |
|  | CONTROL | 91.524 | 1.1230 | 89.323 | 93.725 |

| **Pairwise Comparisons** | | | | | | | |
| --- | --- | --- | --- | --- | --- | --- | --- |
| (I) TIME*GROUP | (J) TIME*GROUP | Mean Difference (I-J) | Std. Error | df | Sequential Bonferroni Sig. | 95% Wald Confidence Interval for Difference^a^ | |
|  |  |  |  |  |  | Lower | Upper |
| [TIME=1.00]*[GROUP=1.00] | [TIME=1.00]*[GROUP=2.00] | .967 | 1.5581 | 1 | 1.000 | -2.485 | 4.418 |
|  | [TIME=1.00]*[GROUP=3.00] | .289 | 1.5674 | 1 | 1.000 | -2.888 | 3.466 |
|  | [TIME=1.00]*[GROUP=4.00] | .267 | 1.5659 | 1 | 1.000 | -2.899 | 3.432 |
|  | [TIME=2.00]*[GROUP=1.00] | 1.868^b^ | .2384 | 1 | .000 | 1.131 | 2.605 |
|  | [TIME=2.00]*[GROUP=2.00] | 4.693^b^ | 1.5362 | 1 | .047 | .026 | 9.360 |
|  | [TIME=2.00]*[GROUP=3.00] | 5.819^b^ | 1.5537 | 1 | .004 | 1.037 | 10.601 |
|  | [TIME=2.00]*[GROUP=4.00] | 1.693 | 1.5799 | 1 | 1.000 | -2.181 | 5.567 |
| [TIME=1.00]*[GROUP=2.00] | [TIME=1.00]*[GROUP=1.00] | -.967 | 1.5581 | 1 | 1.000 | -4.418 | 2.485 |
|  | [TIME=1.00]*[GROUP=3.00] | -.678 | 1.5539 | 1 | 1.000 | -3.988 | 2.633 |
|  | [TIME=1.00]*[GROUP=4.00] | -.700 | 1.5523 | 1 | 1.000 | -4.017 | 2.617 |
|  | [TIME=2.00]*[GROUP=1.00] | .902 | 1.6311 | 1 | 1.000 | -2.659 | 4.463 |
|  | [TIME=2.00]*[GROUP=2.00] | 3.726^b^ | .2204 | 1 | .000 | 3.038 | 4.415 |
|  | [TIME=2.00]*[GROUP=3.00] | 4.852^b^ | 1.5400 | 1 | .036 | .152 | 9.553 |
|  | [TIME=2.00]*[GROUP=4.00] | .726 | 1.5664 | 1 | 1.000 | -2.630 | 4.082 |
| [TIME=1.00]*[GROUP=3.00] | [TIME=1.00]*[GROUP=1.00] | -.289 | 1.5674 | 1 | 1.000 | -3.466 | 2.888 |
|  | [TIME=1.00]*[GROUP=2.00] | .678 | 1.5539 | 1 | 1.000 | -2.633 | 3.988 |
|  | [TIME=1.00]*[GROUP=4.00] | -.022 | 1.5618 | 1 | 1.000 | -3.091 | 3.046 |
|  | [TIME=2.00]*[GROUP=1.00] | 1.579 | 1.6401 | 1 | 1.000 | -2.343 | 5.502 |
|  | [TIME=2.00]*[GROUP=2.00] | 4.404 | 1.5319 | 1 | .077 | -.204 | 9.011 |
|  | [TIME=2.00]*[GROUP=3.00] | 5.530^b^ | .1637 | 1 | .000 | 5.021 | 6.040 |
|  | [TIME=2.00]*[GROUP=4.00] | 1.404 | 1.5757 | 1 | 1.000 | -2.303 | 5.111 |
| [TIME=1.00]*[GROUP=4.00] | [TIME=1.00]*[GROUP=1.00] | -.267 | 1.5659 | 1 | 1.000 | -3.432 | 2.899 |
|  | [TIME=1.00]*[GROUP=2.00] | .700 | 1.5523 | 1 | 1.000 | -2.617 | 4.017 |
|  | [TIME=1.00]*[GROUP=3.00] | .022 | 1.5618 | 1 | 1.000 | -3.046 | 3.091 |
|  | [TIME=2.00]*[GROUP=1.00] | 1.602 | 1.6386 | 1 | 1.000 | -2.330 | 5.533 |
|  | [TIME=2.00]*[GROUP=2.00] | 4.426 | 1.5304 | 1 | .077 | -.201 | 9.053 |
|  | [TIME=2.00]*[GROUP=3.00] | 5.552^b^ | 1.5480 | 1 | .008 | .807 | 10.298 |
|  | [TIME=2.00]*[GROUP=4.00] | 1.426^b^ | .1488 | 1 | .000 | .965 | 1.888 |
| [TIME=2.00]*[GROUP=1.00] | [TIME=1.00]*[GROUP=1.00] | -1.868^b^ | .2384 | 1 | .000 | -2.605 | -1.131 |
|  | [TIME=1.00]*[GROUP=2.00] | -.902 | 1.6311 | 1 | 1.000 | -4.463 | 2.659 |
|  | [TIME=1.00]*[GROUP=3.00] | -1.579 | 1.6401 | 1 | 1.000 | -5.502 | 2.343 |
|  | [TIME=1.00]*[GROUP=4.00] | -1.602 | 1.6386 | 1 | 1.000 | -5.533 | 2.330 |
|  | [TIME=2.00]*[GROUP=2.00] | 2.825 | 1.6102 | 1 | 1.000 | -1.902 | 7.551 |
|  | [TIME=2.00]*[GROUP=3.00] | 3.951 | 1.6269 | 1 | .258 | -.887 | 8.789 |
|  | [TIME=2.00]*[GROUP=4.00] | -.175 | 1.6520 | 1 | 1.000 | -3.475 | 3.124 |
| [TIME=2.00]*[GROUP=2.00] | [TIME=1.00]*[GROUP=1.00] | -4.693^b^ | 1.5362 | 1 | .047 | -9.360 | -.026 |
|  | [TIME=1.00]*[GROUP=2.00] | -3.726^b^ | .2204 | 1 | .000 | -4.415 | -3.038 |
|  | [TIME=1.00]*[GROUP=3.00] | -4.404 | 1.5319 | 1 | .077 | -9.011 | .204 |
|  | [TIME=1.00]*[GROUP=4.00] | -4.426 | 1.5304 | 1 | .077 | -9.053 | .201 |
|  | [TIME=2.00]*[GROUP=1.00] | -2.825 | 1.6102 | 1 | 1.000 | -7.551 | 1.902 |
|  | [TIME=2.00]*[GROUP=3.00] | 1.126 | 1.5179 | 1 | 1.000 | -2.327 | 4.580 |
|  | [TIME=2.00]*[GROUP=4.00] | -3.000 | 1.5447 | 1 | .834 | -7.565 | 1.565 |
| [TIME=2.00]*[GROUP=3.00] | [TIME=1.00]*[GROUP=1.00] | -5.819^b^ | 1.5537 | 1 | .004 | -10.601 | -1.037 |
|  | [TIME=1.00]*[GROUP=2.00] | -4.852^b^ | 1.5400 | 1 | .036 | -9.553 | -.152 |
|  | [TIME=1.00]*[GROUP=3.00] | -5.530^b^ | .1637 | 1 | .000 | -6.040 | -5.021 |
|  | [TIME=1.00]*[GROUP=4.00] | -5.552^b^ | 1.5480 | 1 | .008 | -10.298 | -.807 |
|  | [TIME=2.00]*[GROUP=1.00] | -3.951 | 1.6269 | 1 | .258 | -8.789 | .887 |
|  | [TIME=2.00]*[GROUP=2.00] | -1.126 | 1.5179 | 1 | 1.000 | -4.580 | 2.327 |
|  | [TIME=2.00]*[GROUP=4.00] | -4.126 | 1.5621 | 1 | .149 | -8.799 | .546 |
| [TIME=2.00]*[GROUP=4.00] | [TIME=1.00]*[GROUP=1.00] | -1.693 | 1.5799 | 1 | 1.000 | -5.567 | 2.181 |
|  | [TIME=1.00]*[GROUP=2.00] | -.726 | 1.5664 | 1 | 1.000 | -4.082 | 2.630 |
|  | [TIME=1.00]*[GROUP=3.00] | -1.404 | 1.5757 | 1 | 1.000 | -5.111 | 2.303 |
|  | [TIME=1.00]*[GROUP=4.00] | -1.426^b^ | .1488 | 1 | .000 | -1.888 | -.965 |
|  | [TIME=2.00]*[GROUP=1.00] | .175 | 1.6520 | 1 | 1.000 | -3.124 | 3.475 |
|  | [TIME=2.00]*[GROUP=2.00] | 3.000 | 1.5447 | 1 | .834 | -1.565 | 7.565 |
|  | [TIME=2.00]*[GROUP=3.00] | 4.126 | 1.5621 | 1 | .149 | -.546 | 8.799 |
| Pairwise comparisons of estimated marginal means based on the original scale of dependent variable waist_circumstance | | | | | | | |
| a. Confidence interval bounds are approximate. | | | | | | | |
| b. The mean difference is significant at the .05 level. | | | | | | | |

| **Overall Test Results** | | |
| --- | --- | --- |
| Wald Chi-Square | df | Sig. |
| 1642.649 | 7 | .000 |
| The Wald chi-square tests the effect of TIME*GROUP. This test is based on the linearly independent pairwise comparisons among the estimated marginal means. | | |

* Generalized Estimating Equations.

GENLIN energy_intake BY TIME GROUP (ORDER=ASCENDING)

/MODEL TIME GROUP TIME*GROUP INTERCEPT=YES

DISTRIBUTION=NORMAL LINK=IDENTITY

/CRITERIA SCALE=MLE PCONVERGE=1E-006(ABSOLUTE) SINGULAR=1E-012 ANALYSISTYPE=3(WALD) CILEVEL=95

LIKELIHOOD=FULL

/EMMEANS TABLES=TIME*GROUP SCALE=ORIGINAL COMPARE=TIME*GROUP CONTRAST=PAIRWISE

PADJUST=SEQBONFERRONI

/REPEATED SUBJECT=ID WITHINSUBJECT=TIME SORT=YES CORRTYPE=AR(1) ADJUSTCORR=YES COVB=ROBUST

MAXITERATIONS=100 PCONVERGE=1e-006(ABSOLUTE) UPDATECORR=1

/MISSING CLASSMISSING=EXCLUDE

/PRINT CPS DESCRIPTIVES MODELINFO FIT SUMMARY SOLUTION.

**Generalized Linear Models**

| **Notes** | | |
| --- | --- | --- |
| Output Created | | 09-JUN-2023 00:04:40 |
| Comments | |  |
| Input | Data | C:\Users\mdanaee\Google Drive\Data Analysis\Prof SOh students\Prof Soh -Wang\DATA GEEcr.sav |
|  | Active Dataset | DataSet1 |
|  | Filter | <none> |
|  | Weight | <none> |
|  | Split File | <none> |
|  | N of Rows in Working Data File | 144 |
| Missing Value Handling | Definition of Missing | User-defined missing values for factor, subject and within-subject variables are treated as missing. |
|  | Cases Used | Statistics are based on cases with valid data for all variables in the model. |
| Weight Handling | | not applicable |
| Syntax | | GENLIN energy_intake BY TIME GROUP (ORDER=ASCENDING)  /MODEL TIME GROUP TIME*GROUP INTERCEPT=YES  DISTRIBUTION=NORMAL LINK=IDENTITY  /CRITERIA SCALE=MLE PCONVERGE=1E-006(ABSOLUTE) SINGULAR=1E-012 ANALYSISTYPE=3(WALD) CILEVEL=95  LIKELIHOOD=FULL  /EMMEANS TABLES=TIME*GROUP SCALE=ORIGINAL COMPARE=TIME*GROUP CONTRAST=PAIRWISE  PADJUST=SEQBONFERRONI  /REPEATED SUBJECT=ID WITHINSUBJECT=TIME SORT=YES CORRTYPE=AR(1) ADJUSTCORR=YES COVB=ROBUST  MAXITERATIONS=100 PCONVERGE=1e-006(ABSOLUTE) UPDATECORR=1  /MISSING CLASSMISSING=EXCLUDE  /PRINT CPS DESCRIPTIVES MODELINFO FIT SUMMARY SOLUTION. |
| Resources | Processor Time | 00:00:00.13 |
|  | Elapsed Time | 00:00:00.12 |

| **Model Information** | | |
| --- | --- | --- |
| Dependent Variable | | energy_intake |
| Probability Distribution | | Normal |
| Link Function | | Identity |
| Subject Effect | 1 | ID |
| Within-Subject Effect | 1 | TIME |
| Working Correlation Matrix Structure | | AR(1) |

| **Case Processing Summary** | | |
| --- | --- | --- |
|  | N | Percent |
| Included | 137 | 95.1% |
| Excluded | 7 | 4.9% |
| Total | 144 | 100.0% |

| **Correlated Data Summary** | | | |
| --- | --- | --- | --- |
| Number of Levels | Subject Effect | ID | 72 |
|  | Within-Subject Effect | TIME | 2 |
| Number of Subjects | | | 72 |
| Number of Measurements per Subject | Minimum | | 1 |
|  | Maximum | | 2 |
| Correlation Matrix Dimension | | | 2 |

| **Categorical Variable Information** | | | | |
| --- | --- | --- | --- | --- |
|  | | | N | Percent |
| Factor | TIME | 1.00 | 72 | 52.6% |
|  |  | 2.00 | 65 | 47.4% |
|  |  | Total | 137 | 100.0% |
|  | GROUP | LOW_INTENSITY | 33 | 24.1% |
|  |  | MODERATE_INTENSITY | 35 | 25.5% |
|  |  | HIGH_INTENSITY | 34 | 24.8% |
|  |  | CONTROL | 35 | 25.5% |
|  |  | Total | 137 | 100.0% |

| **Continuous Variable Information** | | | | | | |
| --- | --- | --- | --- | --- | --- | --- |
|  | | N | Minimum | Maximum | Mean | Std. Deviation |
| Dependent Variable | energy_intake | 137 | 1156.26 | 2733.99 | 1953.9069 | 366.97682 |

| **Goodness of Fit^a^** | |
| --- | --- |
|  | Value |
| Quasi Likelihood under Independence Model Criterion (QIC)^b^ | 9534187.021 |
| Corrected Quasi Likelihood under Independence Model Criterion (QICC)^b^ | 9534186.278 |
| Dependent Variable: energy_intake  Model: (Intercept), TIME, GROUP, TIME * GROUP^a^ | |
| a. Information criteria are in smaller-is-better form. | |
| b. Computed using the full log quasi-likelihood function. | |

| **Tests of Model Effects** | | | |
| --- | --- | --- | --- |
| Source | Type III | | |
|  | Wald Chi-Square | df | Sig. |
| (Intercept) | 3766.200 | 1 | .000 |
| TIME | 2112.914 | 1 | .000 |
| GROUP | 11.481 | 3 | .009 |
| TIME * GROUP | 1003.205 | 3 | .000 |
| Dependent Variable: energy_intake  Model: (Intercept), TIME, GROUP, TIME * GROUP | | | |

| **Parameter Estimates** | | | | | | | |
| --- | --- | --- | --- | --- | --- | --- | --- |
| Parameter | B | Std. Error | 95% Wald Confidence Interval | | Hypothesis Test | | |
|  |  |  | Lower | Upper | Wald Chi-Square | df | Sig. |
| (Intercept) | 2070.393 | 66.1181 | 1940.804 | 2199.982 | 980.539 | 1 | .000 |
| [TIME=1.00] | 87.757 | 8.6517 | 70.800 | 104.714 | 102.886 | 1 | .000 |
| [TIME=2.00] | 0^a^ | . | . | . | . | . | . |
| [GROUP=1.00] | -248.180 | 95.5251 | -435.406 | -60.954 | 6.750 | 1 | .009 |
| [GROUP=2.00] | -539.338 | 83.9416 | -703.861 | -374.816 | 41.283 | 1 | .000 |
| [GROUP=3.00] | -393.838 | 88.1194 | -566.549 | -221.127 | 19.975 | 1 | .000 |
| [GROUP=4.00] | 0^a^ | . | . | . | . | . | . |
| [TIME=1.00] * [GROUP=1.00] | 287.880 | 24.4310 | 239.996 | 335.764 | 138.848 | 1 | .000 |
| [TIME=1.00] * [GROUP=2.00] | 509.178 | 19.8899 | 470.194 | 548.162 | 655.350 | 1 | .000 |
| [TIME=1.00] * [GROUP=3.00] | 374.238 | 15.9384 | 342.999 | 405.477 | 551.322 | 1 | .000 |
| [TIME=1.00] * [GROUP=4.00] | 0^a^ | . | . | . | . | . | . |
| [TIME=2.00] * [GROUP=1.00] | 0^a^ | . | . | . | . | . | . |
| [TIME=2.00] * [GROUP=2.00] | 0^a^ | . | . | . | . | . | . |
| [TIME=2.00] * [GROUP=3.00] | 0^a^ | . | . | . | . | . | . |
| [TIME=2.00] * [GROUP=4.00] | 0^a^ | . | . | . | . | . | . |
| (Scale) | 73908.297 |  |  |  |  |  |  |
| Dependent Variable: energy_intake  Model: (Intercept), TIME, GROUP, TIME * GROUP | | | | | | | |
| a. Set to zero because this parameter is redundant. | | | | | | | |

**Estimated Marginal Means: TIME* GROUP**

| **Estimates** | | | | | |
| --- | --- | --- | --- | --- | --- |
| TIME | GROUP | Mean | Std. Error | 95% Wald Confidence Interval | |
|  |  |  |  | Lower | Upper |
| 1.00 | LOW_INTENSITY | 2197.8500 | 68.72160 | 2063.1581 | 2332.5419 |
|  | MODERATE_INTENSITY | 2127.9900 | 66.53516 | 1997.5835 | 2258.3965 |
|  | HIGH_INTENSITY | 2138.5500 | 66.86621 | 2007.4946 | 2269.6054 |
|  | CONTROL | 2158.1500 | 67.48013 | 2025.8914 | 2290.4086 |
| 2.00 | LOW_INTENSITY | 1822.2130 | 68.94515 | 1687.0830 | 1957.3430 |
|  | MODERATE_INTENSITY | 1531.0551 | 51.71641 | 1429.6928 | 1632.4174 |
|  | HIGH_INTENSITY | 1676.5549 | 58.25317 | 1562.3808 | 1790.7291 |
|  | CONTROL | 2070.3931 | 66.11810 | 1940.8040 | 2199.9822 |

| **Pairwise Comparisons** | | | | | | | |
| --- | --- | --- | --- | --- | --- | --- | --- |
| (I) TIME*GROUP | (J) TIME*GROUP | Mean Difference (I-J) | Std. Error | df | Sequential Bonferroni Sig. | 95% Wald Confidence Interval for Difference^a^ | |
|  |  |  |  |  |  | Lower | Upper |
| [TIME=1.00]*[GROUP=1.00] | [TIME=1.00]*[GROUP=2.00] | 69.8600 | 95.65347 | 1 | 1.000 | -147.1924 | 286.9124 |
|  | [TIME=1.00]*[GROUP=3.00] | 59.3000 | 95.88404 | 1 | 1.000 | -153.0082 | 271.6082 |
|  | [TIME=1.00]*[GROUP=4.00] | 39.7000 | 96.31317 | 1 | 1.000 | -164.4797 | 243.8797 |
|  | [TIME=2.00]*[GROUP=1.00] | 375.6370^b^ | 22.84782 | 1 | .000 | 304.2665 | 447.0075 |
|  | [TIME=2.00]*[GROUP=2.00] | 666.7949^b^ | 86.00724 | 1 | .000 | 402.0571 | 931.5328 |
|  | [TIME=2.00]*[GROUP=3.00] | 521.2951^b^ | 90.08935 | 1 | .000 | 248.9242 | 793.6659 |
|  | [TIME=2.00]*[GROUP=4.00] | 127.4569 | 95.36384 | 1 | 1.000 | -121.3904 | 376.3042 |
| [TIME=1.00]*[GROUP=2.00] | [TIME=1.00]*[GROUP=1.00] | -69.8600 | 95.65347 | 1 | 1.000 | -286.9124 | 147.1924 |
|  | [TIME=1.00]*[GROUP=3.00] | -10.5600 | 94.32931 | 1 | 1.000 | -199.1811 | 178.0611 |
|  | [TIME=1.00]*[GROUP=4.00] | -30.1600 | 94.76548 | 1 | 1.000 | -227.2837 | 166.9637 |
|  | [TIME=2.00]*[GROUP=1.00] | 305.7770^b^ | 95.81420 | 1 | .018 | 28.8250 | 582.7291 |
|  | [TIME=2.00]*[GROUP=2.00] | 596.9349^b^ | 17.90967 | 1 | .000 | 541.1818 | 652.6881 |
|  | [TIME=2.00]*[GROUP=3.00] | 451.4351^b^ | 88.43280 | 1 | .000 | 186.9046 | 715.9655 |
|  | [TIME=2.00]*[GROUP=4.00] | 57.5969 | 93.80049 | 1 | 1.000 | -149.8994 | 265.0932 |
| [TIME=1.00]*[GROUP=3.00] | [TIME=1.00]*[GROUP=1.00] | -59.3000 | 95.88404 | 1 | 1.000 | -271.6082 | 153.0082 |
|  | [TIME=1.00]*[GROUP=2.00] | 10.5600 | 94.32931 | 1 | 1.000 | -178.0611 | 199.1811 |
|  | [TIME=1.00]*[GROUP=4.00] | -19.6000 | 94.99820 | 1 | 1.000 | -212.9438 | 173.7438 |
|  | [TIME=2.00]*[GROUP=1.00] | 316.3370^b^ | 96.04438 | 1 | .014 | 36.4900 | 596.1841 |
|  | [TIME=2.00]*[GROUP=2.00] | 607.4949^b^ | 84.53211 | 1 | .000 | 349.4974 | 865.4924 |
|  | [TIME=2.00]*[GROUP=3.00] | 461.9951^b^ | 13.38583 | 1 | .000 | 420.4741 | 503.5161 |
|  | [TIME=2.00]*[GROUP=4.00] | 68.1569 | 94.03560 | 1 | 1.000 | -144.9625 | 281.2763 |
| [TIME=1.00]*[GROUP=4.00] | [TIME=1.00]*[GROUP=1.00] | -39.7000 | 96.31317 | 1 | 1.000 | -243.8797 | 164.4797 |
|  | [TIME=1.00]*[GROUP=2.00] | 30.1600 | 94.76548 | 1 | 1.000 | -166.9637 | 227.2837 |
|  | [TIME=1.00]*[GROUP=3.00] | 19.6000 | 94.99820 | 1 | 1.000 | -173.7438 | 212.9438 |
|  | [TIME=2.00]*[GROUP=1.00] | 335.9370^b^ | 96.47280 | 1 | .008 | 50.8438 | 621.0303 |
|  | [TIME=2.00]*[GROUP=2.00] | 627.0949^b^ | 85.01856 | 1 | .000 | 366.4805 | 887.7094 |
|  | [TIME=2.00]*[GROUP=3.00] | 481.5951^b^ | 89.14595 | 1 | .000 | 213.4631 | 749.7270 |
|  | [TIME=2.00]*[GROUP=4.00] | 87.7569^b^ | 8.65175 | 1 | .000 | 61.0210 | 114.4928 |
| [TIME=2.00]*[GROUP=1.00] | [TIME=1.00]*[GROUP=1.00] | -375.6370^b^ | 22.84782 | 1 | .000 | -447.0075 | -304.2665 |
|  | [TIME=1.00]*[GROUP=2.00] | -305.7770^b^ | 95.81420 | 1 | .018 | -582.7291 | -28.8250 |
|  | [TIME=1.00]*[GROUP=3.00] | -316.3370^b^ | 96.04438 | 1 | .014 | -596.1841 | -36.4900 |
|  | [TIME=1.00]*[GROUP=4.00] | -335.9370^b^ | 96.47280 | 1 | .008 | -621.0303 | -50.8438 |
|  | [TIME=2.00]*[GROUP=2.00] | 291.1579^b^ | 86.18596 | 1 | .011 | 38.1849 | 544.1309 |
|  | [TIME=2.00]*[GROUP=3.00] | 145.6580 | 90.25999 | 1 | 1.000 | -107.7048 | 399.0209 |
|  | [TIME=2.00]*[GROUP=4.00] | -248.1801 | 95.52506 | 1 | .113 | -521.8843 | 25.5240 |
| [TIME=2.00]*[GROUP=2.00] | [TIME=1.00]*[GROUP=1.00] | -666.7949^b^ | 86.00724 | 1 | .000 | -931.5328 | -402.0571 |
|  | [TIME=1.00]*[GROUP=2.00] | -596.9349^b^ | 17.90967 | 1 | .000 | -652.6881 | -541.1818 |
|  | [TIME=1.00]*[GROUP=3.00] | -607.4949^b^ | 84.53211 | 1 | .000 | -865.4924 | -349.4974 |
|  | [TIME=1.00]*[GROUP=4.00] | -627.0949^b^ | 85.01856 | 1 | .000 | -887.7094 | -366.4805 |
|  | [TIME=2.00]*[GROUP=1.00] | -291.1579^b^ | 86.18596 | 1 | .011 | -544.1309 | -38.1849 |
|  | [TIME=2.00]*[GROUP=3.00] | -145.4999 | 77.89749 | 1 | .680 | -366.5416 | 75.5418 |
|  | [TIME=2.00]*[GROUP=4.00] | -539.3380^b^ | 83.94159 | 1 | .000 | -794.3588 | -284.3172 |
| [TIME=2.00]*[GROUP=3.00] | [TIME=1.00]*[GROUP=1.00] | -521.2951^b^ | 90.08935 | 1 | .000 | -793.6659 | -248.9242 |
|  | [TIME=1.00]*[GROUP=2.00] | -451.4351^b^ | 88.43280 | 1 | .000 | -715.9655 | -186.9046 |
|  | [TIME=1.00]*[GROUP=3.00] | -461.9951^b^ | 13.38583 | 1 | .000 | -503.5161 | -420.4741 |
|  | [TIME=1.00]*[GROUP=4.00] | -481.5951^b^ | 89.14595 | 1 | .000 | -749.7270 | -213.4631 |
|  | [TIME=2.00]*[GROUP=1.00] | -145.6580 | 90.25999 | 1 | 1.000 | -399.0209 | 107.7048 |
|  | [TIME=2.00]*[GROUP=2.00] | 145.4999 | 77.89749 | 1 | .680 | -75.5418 | 366.5416 |
|  | [TIME=2.00]*[GROUP=4.00] | -393.8381^b^ | 88.11944 | 1 | .000 | -655.8895 | -131.7868 |
| [TIME=2.00]*[GROUP=4.00] | [TIME=1.00]*[GROUP=1.00] | -127.4569 | 95.36384 | 1 | 1.000 | -376.3042 | 121.3904 |
|  | [TIME=1.00]*[GROUP=2.00] | -57.5969 | 93.80049 | 1 | 1.000 | -265.0932 | 149.8994 |
|  | [TIME=1.00]*[GROUP=3.00] | -68.1569 | 94.03560 | 1 | 1.000 | -281.2763 | 144.9625 |
|  | [TIME=1.00]*[GROUP=4.00] | -87.7569^b^ | 8.65175 | 1 | .000 | -114.4928 | -61.0210 |
|  | [TIME=2.00]*[GROUP=1.00] | 248.1801 | 95.52506 | 1 | .113 | -25.5240 | 521.8843 |
|  | [TIME=2.00]*[GROUP=2.00] | 539.3380^b^ | 83.94159 | 1 | .000 | 284.3172 | 794.3588 |
|  | [TIME=2.00]*[GROUP=3.00] | 393.8381^b^ | 88.11944 | 1 | .000 | 131.7868 | 655.8895 |
| Pairwise comparisons of estimated marginal means based on the original scale of dependent variable energy_intake | | | | | | | |
| a. Confidence interval bounds are approximate. | | | | | | | |
| b. The mean difference is significant at the .05 level. | | | | | | | |

| **Overall Test Results** | | |
| --- | --- | --- |
| Wald Chi-Square | df | Sig. |
| 3695.301 | 7 | .000 |
| The Wald chi-square tests the effect of TIME*GROUP. This test is based on the linearly independent pairwise comparisons among the estimated marginal means. | | |

* Generalized Estimating Equations.

GENLIN protein_intake BY TIME GROUP (ORDER=ASCENDING)

/MODEL TIME GROUP TIME*GROUP INTERCEPT=YES

DISTRIBUTION=NORMAL LINK=IDENTITY

/CRITERIA SCALE=MLE PCONVERGE=1E-006(ABSOLUTE) SINGULAR=1E-012 ANALYSISTYPE=3(WALD) CILEVEL=95

LIKELIHOOD=FULL

/EMMEANS TABLES=TIME*GROUP SCALE=ORIGINAL COMPARE=TIME*GROUP CONTRAST=PAIRWISE

PADJUST=SEQBONFERRONI

/REPEATED SUBJECT=ID WITHINSUBJECT=TIME SORT=YES CORRTYPE=AR(1) ADJUSTCORR=YES COVB=ROBUST

MAXITERATIONS=100 PCONVERGE=1e-006(ABSOLUTE) UPDATECORR=1

/MISSING CLASSMISSING=EXCLUDE

/PRINT CPS DESCRIPTIVES MODELINFO FIT SUMMARY SOLUTION.

**Generalized Linear Models**

| **Notes** | | |
| --- | --- | --- |
| Output Created | | 09-JUN-2023 00:04:40 |
| Comments | |  |
| Input | Data | C:\Users\mdanaee\Google Drive\Data Analysis\Prof SOh students\Prof Soh -Wang\DATA GEEcr.sav |
|  | Active Dataset | DataSet1 |
|  | Filter | <none> |
|  | Weight | <none> |
|  | Split File | <none> |
|  | N of Rows in Working Data File | 144 |
| Missing Value Handling | Definition of Missing | User-defined missing values for factor, subject and within-subject variables are treated as missing. |
|  | Cases Used | Statistics are based on cases with valid data for all variables in the model. |
| Weight Handling | | not applicable |
| Syntax | | GENLIN protein_intake BY TIME GROUP (ORDER=ASCENDING)  /MODEL TIME GROUP TIME*GROUP INTERCEPT=YES  DISTRIBUTION=NORMAL LINK=IDENTITY  /CRITERIA SCALE=MLE PCONVERGE=1E-006(ABSOLUTE) SINGULAR=1E-012 ANALYSISTYPE=3(WALD) CILEVEL=95  LIKELIHOOD=FULL  /EMMEANS TABLES=TIME*GROUP SCALE=ORIGINAL COMPARE=TIME*GROUP CONTRAST=PAIRWISE  PADJUST=SEQBONFERRONI  /REPEATED SUBJECT=ID WITHINSUBJECT=TIME SORT=YES CORRTYPE=AR(1) ADJUSTCORR=YES COVB=ROBUST  MAXITERATIONS=100 PCONVERGE=1e-006(ABSOLUTE) UPDATECORR=1  /MISSING CLASSMISSING=EXCLUDE  /PRINT CPS DESCRIPTIVES MODELINFO FIT SUMMARY SOLUTION. |
| Resources | Processor Time | 00:00:00.14 |
|  | Elapsed Time | 00:00:00.11 |

| **Model Information** | | |
| --- | --- | --- |
| Dependent Variable | | protein_intake |
| Probability Distribution | | Normal |
| Link Function | | Identity |
| Subject Effect | 1 | ID |
| Within-Subject Effect | 1 | TIME |
| Working Correlation Matrix Structure | | AR(1) |

| **Case Processing Summary** | | |
| --- | --- | --- |
|  | N | Percent |
| Included | 137 | 95.1% |
| Excluded | 7 | 4.9% |
| Total | 144 | 100.0% |

| **Correlated Data Summary** | | | |
| --- | --- | --- | --- |
| Number of Levels | Subject Effect | ID | 72 |
|  | Within-Subject Effect | TIME | 2 |
| Number of Subjects | | | 72 |
| Number of Measurements per Subject | Minimum | | 1 |
|  | Maximum | | 2 |
| Correlation Matrix Dimension | | | 2 |

| **Categorical Variable Information** | | | | |
| --- | --- | --- | --- | --- |
|  | | | N | Percent |
| Factor | TIME | 1.00 | 72 | 52.6% |
|  |  | 2.00 | 65 | 47.4% |
|  |  | Total | 137 | 100.0% |
|  | GROUP | LOW_INTENSITY | 33 | 24.1% |
|  |  | MODERATE_INTENSITY | 35 | 25.5% |
|  |  | HIGH_INTENSITY | 34 | 24.8% |
|  |  | CONTROL | 35 | 25.5% |
|  |  | Total | 137 | 100.0% |

| **Continuous Variable Information** | | | | | | |
| --- | --- | --- | --- | --- | --- | --- |
|  | | N | Minimum | Maximum | Mean | Std. Deviation |
| Dependent Variable | protein_intake | 137 | 35.05 | 116.89 | 74.7877 | 21.24025 |

| **Goodness of Fit^a^** | |
| --- | --- |
|  | Value |
| Quasi Likelihood under Independence Model Criterion (QIC)^b^ | 52580.125 |
| Corrected Quasi Likelihood under Independence Model Criterion (QICC)^b^ | 52579.823 |
| Dependent Variable: protein_intake  Model: (Intercept), TIME, GROUP, TIME * GROUP^a^ | |
| a. Information criteria are in smaller-is-better form. | |
| b. Computed using the full log quasi-likelihood function. | |

| **Tests of Model Effects** | | | |
| --- | --- | --- | --- |
| Source | Type III | | |
|  | Wald Chi-Square | df | Sig. |
| (Intercept) | 1060.543 | 1 | .000 |
| TIME | 125.627 | 1 | .000 |
| GROUP | 4.183 | 3 | .242 |
| TIME * GROUP | 188.428 | 3 | .000 |
| Dependent Variable: protein_intake  Model: (Intercept), TIME, GROUP, TIME * GROUP | | | |

| **Parameter Estimates** | | | | | | | |
| --- | --- | --- | --- | --- | --- | --- | --- |
| Parameter | B | Std. Error | 95% Wald Confidence Interval | | Hypothesis Test | | |
|  |  |  | Lower | Upper | Wald Chi-Square | df | Sig. |
| (Intercept) | 79.461 | 4.9387 | 69.781 | 89.140 | 258.864 | 1 | .000 |
| [TIME=1.00] | 1.119 | .9565 | -.755 | 2.994 | 1.369 | 1 | .242 |
| [TIME=2.00] | 0^a^ | . | . | . | . | . | . |
| [GROUP=1.00] | -2.277 | 6.9490 | -15.897 | 11.343 | .107 | 1 | .743 |
| [GROUP=2.00] | -22.411 | 6.1821 | -34.528 | -10.295 | 13.142 | 1 | .000 |
| [GROUP=3.00] | -6.658 | 6.5833 | -19.561 | 6.245 | 1.023 | 1 | .312 |
| [GROUP=4.00] | 0^a^ | . | . | . | . | . | . |
| [TIME=1.00] * [GROUP=1.00] | 1.917 | 1.1219 | -.282 | 4.116 | 2.920 | 1 | .087 |
| [TIME=1.00] * [GROUP=2.00] | 21.431 | 1.6901 | 18.119 | 24.744 | 160.802 | 1 | .000 |
| [TIME=1.00] * [GROUP=3.00] | 7.678 | 2.7837 | 2.222 | 13.134 | 7.607 | 1 | .006 |
| [TIME=1.00] * [GROUP=4.00] | 0^a^ | . | . | . | . | . | . |
| [TIME=2.00] * [GROUP=1.00] | 0^a^ | . | . | . | . | . | . |
| [TIME=2.00] * [GROUP=2.00] | 0^a^ | . | . | . | . | . | . |
| [TIME=2.00] * [GROUP=3.00] | 0^a^ | . | . | . | . | . | . |
| [TIME=2.00] * [GROUP=4.00] | 0^a^ | . | . | . | . | . | . |
| (Scale) | 407.471 |  |  |  |  |  |  |
| Dependent Variable: protein_intake  Model: (Intercept), TIME, GROUP, TIME * GROUP | | | | | | | |
| a. Set to zero because this parameter is redundant. | | | | | | | |

**Estimated Marginal Means: TIME* GROUP**

| **Estimates** | | | | | |
| --- | --- | --- | --- | --- | --- |
| TIME | GROUP | Mean | Std. Error | 95% Wald Confidence Interval | |
|  |  |  |  | Lower | Upper |
| 1.00 | LOW_INTENSITY | 80.2200 | 4.95712 | 70.5042 | 89.9358 |
|  | MODERATE_INTENSITY | 79.6000 | 4.92074 | 69.9555 | 89.2445 |
|  | HIGH_INTENSITY | 81.6000 | 4.99358 | 71.8128 | 91.3872 |
|  | CONTROL | 80.5800 | 4.97937 | 70.8206 | 90.3394 |
| 2.00 | LOW_INTENSITY | 77.1836 | 4.88854 | 67.6022 | 86.7650 |
|  | MODERATE_INTENSITY | 57.0491 | 3.71852 | 49.7610 | 64.3373 |
|  | HIGH_INTENSITY | 72.8029 | 4.35305 | 64.2711 | 81.3347 |
|  | CONTROL | 79.4606 | 4.93874 | 69.7809 | 89.1404 |

| **Pairwise Comparisons** | | | | | | | |
| --- | --- | --- | --- | --- | --- | --- | --- |
| (I) TIME*GROUP | (J) TIME*GROUP | Mean Difference (I-J) | Std. Error | df | Sequential Bonferroni Sig. | 95% Wald Confidence Interval for Difference^a^ | |
|  |  |  |  |  |  | Lower | Upper |
| [TIME=1.00]*[GROUP=1.00] | [TIME=1.00]*[GROUP=2.00] | .6200 | 6.98475 | 1 | 1.000 | -13.2877 | 14.5277 |
|  | [TIME=1.00]*[GROUP=3.00] | -1.3800 | 7.03625 | 1 | 1.000 | -15.6727 | 12.9127 |
|  | [TIME=1.00]*[GROUP=4.00] | -.3600 | 7.02618 | 1 | 1.000 | -14.2560 | 13.5360 |
|  | [TIME=2.00]*[GROUP=1.00] | 3.0364^b^ | .58619 | 1 | .000 | 1.2116 | 4.8612 |
|  | [TIME=2.00]*[GROUP=2.00] | 23.1709^b^ | 6.19681 | 1 | .004 | 4.0965 | 42.2452 |
|  | [TIME=2.00]*[GROUP=3.00] | 7.4171 | 6.59713 | 1 | 1.000 | -8.9606 | 23.7948 |
|  | [TIME=2.00]*[GROUP=4.00] | .7594 | 6.99744 | 1 | 1.000 | -13.2239 | 14.7427 |
| [TIME=1.00]*[GROUP=2.00] | [TIME=1.00]*[GROUP=1.00] | -.6200 | 6.98475 | 1 | 1.000 | -14.5277 | 13.2877 |
|  | [TIME=1.00]*[GROUP=3.00] | -2.0000 | 7.01067 | 1 | 1.000 | -16.4883 | 12.4883 |
|  | [TIME=1.00]*[GROUP=4.00] | -.9800 | 7.00056 | 1 | 1.000 | -15.0510 | 13.0910 |
|  | [TIME=2.00]*[GROUP=1.00] | 2.4164 | 6.93624 | 1 | 1.000 | -12.0989 | 16.9317 |
|  | [TIME=2.00]*[GROUP=2.00] | 22.5509^b^ | 1.39333 | 1 | .000 | 18.1984 | 26.9033 |
|  | [TIME=2.00]*[GROUP=3.00] | 6.7971 | 6.56983 | 1 | 1.000 | -9.1764 | 22.7706 |
|  | [TIME=2.00]*[GROUP=4.00] | .1394 | 6.97171 | 1 | 1.000 | -13.5728 | 13.8515 |
| [TIME=1.00]*[GROUP=3.00] | [TIME=1.00]*[GROUP=1.00] | 1.3800 | 7.03625 | 1 | 1.000 | -12.9127 | 15.6727 |
|  | [TIME=1.00]*[GROUP=2.00] | 2.0000 | 7.01067 | 1 | 1.000 | -12.4883 | 16.4883 |
|  | [TIME=1.00]*[GROUP=4.00] | 1.0200 | 7.05195 | 1 | 1.000 | -13.1665 | 15.2065 |
|  | [TIME=2.00]*[GROUP=1.00] | 4.4164 | 6.98811 | 1 | 1.000 | -11.1022 | 19.9351 |
|  | [TIME=2.00]*[GROUP=2.00] | 24.5509^b^ | 6.22601 | 1 | .002 | 5.2386 | 43.8631 |
|  | [TIME=2.00]*[GROUP=3.00] | 8.7971^b^ | 2.61416 | 1 | .017 | .8185 | 16.7757 |
|  | [TIME=2.00]*[GROUP=4.00] | 2.1394 | 7.02332 | 1 | 1.000 | -12.4305 | 16.7092 |
| [TIME=1.00]*[GROUP=4.00] | [TIME=1.00]*[GROUP=1.00] | .3600 | 7.02618 | 1 | 1.000 | -13.5360 | 14.2560 |
|  | [TIME=1.00]*[GROUP=2.00] | .9800 | 7.00056 | 1 | 1.000 | -13.0910 | 15.0510 |
|  | [TIME=1.00]*[GROUP=3.00] | -1.0200 | 7.05195 | 1 | 1.000 | -15.2065 | 13.1665 |
|  | [TIME=2.00]*[GROUP=1.00] | 3.3964 | 6.97796 | 1 | 1.000 | -11.6268 | 18.4196 |
|  | [TIME=2.00]*[GROUP=2.00] | 23.5309^b^ | 6.21462 | 1 | .004 | 4.3262 | 42.7355 |
|  | [TIME=2.00]*[GROUP=3.00] | 7.7771 | 6.61386 | 1 | 1.000 | -12.1160 | 27.6702 |
|  | [TIME=2.00]*[GROUP=4.00] | 1.1194 | .95655 | 1 | 1.000 | -1.2809 | 3.5197 |
| [TIME=2.00]*[GROUP=1.00] | [TIME=1.00]*[GROUP=1.00] | -3.0364^b^ | .58619 | 1 | .000 | -4.8612 | -1.2116 |
|  | [TIME=1.00]*[GROUP=2.00] | -2.4164 | 6.93624 | 1 | 1.000 | -16.9317 | 12.0989 |
|  | [TIME=1.00]*[GROUP=3.00] | -4.4164 | 6.98811 | 1 | 1.000 | -19.9351 | 11.1022 |
|  | [TIME=1.00]*[GROUP=4.00] | -3.3964 | 6.97796 | 1 | 1.000 | -18.4196 | 11.6268 |
|  | [TIME=2.00]*[GROUP=2.00] | 20.1344^b^ | 6.14209 | 1 | .022 | 1.4743 | 38.7946 |
|  | [TIME=2.00]*[GROUP=3.00] | 4.3807 | 6.54576 | 1 | 1.000 | -10.2741 | 19.0355 |
|  | [TIME=2.00]*[GROUP=4.00] | -2.2771 | 6.94903 | 1 | 1.000 | -16.7590 | 12.2049 |
| [TIME=2.00]*[GROUP=2.00] | [TIME=1.00]*[GROUP=1.00] | -23.1709^b^ | 6.19681 | 1 | .004 | -42.2452 | -4.0965 |
|  | [TIME=1.00]*[GROUP=2.00] | -22.5509^b^ | 1.39333 | 1 | .000 | -26.9033 | -18.1984 |
|  | [TIME=1.00]*[GROUP=3.00] | -24.5509^b^ | 6.22601 | 1 | .002 | -43.8631 | -5.2386 |
|  | [TIME=1.00]*[GROUP=4.00] | -23.5309^b^ | 6.21462 | 1 | .004 | -42.7355 | -4.3262 |
|  | [TIME=2.00]*[GROUP=1.00] | -20.1344^b^ | 6.14209 | 1 | .022 | -38.7946 | -1.4743 |
|  | [TIME=2.00]*[GROUP=3.00] | -15.7538 | 5.72508 | 1 | .119 | -33.0626 | 1.5551 |
|  | [TIME=2.00]*[GROUP=4.00] | -22.4115^b^ | 6.18212 | 1 | .007 | -41.3621 | -3.4609 |
| [TIME=2.00]*[GROUP=3.00] | [TIME=1.00]*[GROUP=1.00] | -7.4171 | 6.59713 | 1 | 1.000 | -23.7948 | 8.9606 |
|  | [TIME=1.00]*[GROUP=2.00] | -6.7971 | 6.56983 | 1 | 1.000 | -22.7706 | 9.1764 |
|  | [TIME=1.00]*[GROUP=3.00] | -8.7971^b^ | 2.61416 | 1 | .017 | -16.7757 | -.8185 |
|  | [TIME=1.00]*[GROUP=4.00] | -7.7771 | 6.61386 | 1 | 1.000 | -27.6702 | 12.1160 |
|  | [TIME=2.00]*[GROUP=1.00] | -4.3807 | 6.54576 | 1 | 1.000 | -19.0355 | 10.2741 |
|  | [TIME=2.00]*[GROUP=2.00] | 15.7538 | 5.72508 | 1 | .119 | -1.5551 | 33.0626 |
|  | [TIME=2.00]*[GROUP=4.00] | -6.6577 | 6.58333 | 1 | 1.000 | -22.5781 | 9.2627 |
| [TIME=2.00]*[GROUP=4.00] | [TIME=1.00]*[GROUP=1.00] | -.7594 | 6.99744 | 1 | 1.000 | -14.7427 | 13.2239 |
|  | [TIME=1.00]*[GROUP=2.00] | -.1394 | 6.97171 | 1 | 1.000 | -13.8515 | 13.5728 |
|  | [TIME=1.00]*[GROUP=3.00] | -2.1394 | 7.02332 | 1 | 1.000 | -16.7092 | 12.4305 |
|  | [TIME=1.00]*[GROUP=4.00] | -1.1194 | .95655 | 1 | 1.000 | -3.5197 | 1.2809 |
|  | [TIME=2.00]*[GROUP=1.00] | 2.2771 | 6.94903 | 1 | 1.000 | -12.2049 | 16.7590 |
|  | [TIME=2.00]*[GROUP=2.00] | 22.4115^b^ | 6.18212 | 1 | .007 | 3.4609 | 41.3621 |
|  | [TIME=2.00]*[GROUP=3.00] | 6.6577 | 6.58333 | 1 | 1.000 | -9.2627 | 22.5781 |
| Pairwise comparisons of estimated marginal means based on the original scale of dependent variable protein_intake | | | | | | | |
| a. Confidence interval bounds are approximate. | | | | | | | |
| b. The mean difference is significant at the .05 level. | | | | | | | |

| **Overall Test Results** | | |
| --- | --- | --- |
| Wald Chi-Square | df | Sig. |
| 688.035 | 7 | .000 |
| The Wald chi-square tests the effect of TIME*GROUP. This test is based on the linearly independent pairwise comparisons among the estimated marginal means. | | |

* Generalized Estimating Equations.

GENLIN Fat_intake BY TIME GROUP (ORDER=ASCENDING)

/MODEL TIME GROUP TIME*GROUP INTERCEPT=YES

DISTRIBUTION=NORMAL LINK=IDENTITY

/CRITERIA SCALE=MLE PCONVERGE=1E-006(ABSOLUTE) SINGULAR=1E-012 ANALYSISTYPE=3(WALD) CILEVEL=95

LIKELIHOOD=FULL

/EMMEANS TABLES=TIME*GROUP SCALE=ORIGINAL COMPARE=TIME*GROUP CONTRAST=PAIRWISE

PADJUST=SEQBONFERRONI

/REPEATED SUBJECT=ID WITHINSUBJECT=TIME SORT=YES CORRTYPE=AR(1) ADJUSTCORR=YES COVB=ROBUST

MAXITERATIONS=100 PCONVERGE=1e-006(ABSOLUTE) UPDATECORR=1

/MISSING CLASSMISSING=EXCLUDE

/PRINT CPS DESCRIPTIVES MODELINFO FIT SUMMARY SOLUTION.

**Generalized Linear Models**

| **Notes** | | |
| --- | --- | --- |
| Output Created | | 09-JUN-2023 00:04:40 |
| Comments | |  |
| Input | Data | C:\Users\mdanaee\Google Drive\Data Analysis\Prof SOh students\Prof Soh -Wang\DATA GEEcr.sav |
|  | Active Dataset | DataSet1 |
|  | Filter | <none> |
|  | Weight | <none> |
|  | Split File | <none> |
|  | N of Rows in Working Data File | 144 |
| Missing Value Handling | Definition of Missing | User-defined missing values for factor, subject and within-subject variables are treated as missing. |
|  | Cases Used | Statistics are based on cases with valid data for all variables in the model. |
| Weight Handling | | not applicable |
| Syntax | | GENLIN Fat_intake BY TIME GROUP (ORDER=ASCENDING)  /MODEL TIME GROUP TIME*GROUP INTERCEPT=YES  DISTRIBUTION=NORMAL LINK=IDENTITY  /CRITERIA SCALE=MLE PCONVERGE=1E-006(ABSOLUTE) SINGULAR=1E-012 ANALYSISTYPE=3(WALD) CILEVEL=95  LIKELIHOOD=FULL  /EMMEANS TABLES=TIME*GROUP SCALE=ORIGINAL COMPARE=TIME*GROUP CONTRAST=PAIRWISE  PADJUST=SEQBONFERRONI  /REPEATED SUBJECT=ID WITHINSUBJECT=TIME SORT=YES CORRTYPE=AR(1) ADJUSTCORR=YES COVB=ROBUST  MAXITERATIONS=100 PCONVERGE=1e-006(ABSOLUTE) UPDATECORR=1  /MISSING CLASSMISSING=EXCLUDE  /PRINT CPS DESCRIPTIVES MODELINFO FIT SUMMARY SOLUTION. |
| Resources | Processor Time | 00:00:00.13 |
|  | Elapsed Time | 00:00:00.12 |

| **Model Information** | | |
| --- | --- | --- |
| Dependent Variable | | Fat_intake |
| Probability Distribution | | Normal |
| Link Function | | Identity |
| Subject Effect | 1 | ID |
| Within-Subject Effect | 1 | TIME |
| Working Correlation Matrix Structure | | AR(1) |

| **Case Processing Summary** | | |
| --- | --- | --- |
|  | N | Percent |
| Included | 137 | 95.1% |
| Excluded | 7 | 4.9% |
| Total | 144 | 100.0% |

| **Correlated Data Summary** | | | |
| --- | --- | --- | --- |
| Number of Levels | Subject Effect | ID | 72 |
|  | Within-Subject Effect | TIME | 2 |
| Number of Subjects | | | 72 |
| Number of Measurements per Subject | Minimum | | 1 |
|  | Maximum | | 2 |
| Correlation Matrix Dimension | | | 2 |

| **Categorical Variable Information** | | | | |
| --- | --- | --- | --- | --- |
|  | | | N | Percent |
| Factor | TIME | 1.00 | 72 | 52.6% |
|  |  | 2.00 | 65 | 47.4% |
|  |  | Total | 137 | 100.0% |
|  | GROUP | LOW_INTENSITY | 33 | 24.1% |
|  |  | MODERATE_INTENSITY | 35 | 25.5% |
|  |  | HIGH_INTENSITY | 34 | 24.8% |
|  |  | CONTROL | 35 | 25.5% |
|  |  | Total | 137 | 100.0% |

| **Continuous Variable Information** | | | | | | |
| --- | --- | --- | --- | --- | --- | --- |
|  | | N | Minimum | Maximum | Mean | Std. Deviation |
| Dependent Variable | Fat_intake | 137 | 24.95 | 101.66 | 63.9273 | 20.24373 |

| **Goodness of Fit^a^** | |
| --- | --- |
|  | Value |
| Quasi Likelihood under Independence Model Criterion (QIC)^b^ | 45959.399 |
| Corrected Quasi Likelihood under Independence Model Criterion (QICC)^b^ | 45959.077 |
| Dependent Variable: Fat_intake  Model: (Intercept), TIME, GROUP, TIME * GROUP^a^ | |
| a. Information criteria are in smaller-is-better form. | |
| b. Computed using the full log quasi-likelihood function. | |

| **Tests of Model Effects** | | | |
| --- | --- | --- | --- |
| Source | Type III | | |
|  | Wald Chi-Square | df | Sig. |
| (Intercept) | 880.330 | 1 | .000 |
| TIME | 287.389 | 1 | .000 |
| GROUP | 3.906 | 3 | .272 |
| TIME * GROUP | 204.452 | 3 | .000 |
| Dependent Variable: Fat_intake  Model: (Intercept), TIME, GROUP, TIME * GROUP | | | |

| **Parameter Estimates** | | | | | | | |
| --- | --- | --- | --- | --- | --- | --- | --- |
| Parameter | B | Std. Error | 95% Wald Confidence Interval | | Hypothesis Test | | |
|  |  |  | Lower | Upper | Wald Chi-Square | df | Sig. |
| (Intercept) | 68.554 | 4.5054 | 59.724 | 77.385 | 231.531 | 1 | .000 |
| [TIME=1.00] | 1.576 | .6304 | .340 | 2.811 | 6.246 | 1 | .012 |
| [TIME=2.00] | 0^a^ | . | . | . | . | . | . |
| [GROUP=1.00] | -9.727 | 6.1140 | -21.710 | 2.257 | 2.531 | 1 | .112 |
| [GROUP=2.00] | -21.673 | 5.5766 | -32.603 | -10.744 | 15.105 | 1 | .000 |
| [GROUP=3.00] | -2.849 | 6.3926 | -15.379 | 9.680 | .199 | 1 | .656 |
| [GROUP=4.00] | 0^a^ | . | . | . | . | . | . |
| [TIME=1.00] * [GROUP=1.00] | 10.447 | 1.2495 | 7.998 | 12.896 | 69.902 | 1 | .000 |
| [TIME=1.00] * [GROUP=2.00] | 21.563 | 1.6796 | 18.272 | 24.855 | 164.829 | 1 | .000 |
| [TIME=1.00] * [GROUP=3.00] | 2.209 | 1.4582 | -.649 | 5.067 | 2.296 | 1 | .130 |
| [TIME=1.00] * [GROUP=4.00] | 0^a^ | . | . | . | . | . | . |
| [TIME=2.00] * [GROUP=1.00] | 0^a^ | . | . | . | . | . | . |
| [TIME=2.00] * [GROUP=2.00] | 0^a^ | . | . | . | . | . | . |
| [TIME=2.00] * [GROUP=3.00] | 0^a^ | . | . | . | . | . | . |
| [TIME=2.00] * [GROUP=4.00] | 0^a^ | . | . | . | . | . | . |
| (Scale) | 356.148 |  |  |  |  |  |  |
| Dependent Variable: Fat_intake  Model: (Intercept), TIME, GROUP, TIME * GROUP | | | | | | | |
| a. Set to zero because this parameter is redundant. | | | | | | | |

**Estimated Marginal Means: TIME* GROUP**

| **Estimates** | | | | | |
| --- | --- | --- | --- | --- | --- |
| TIME | GROUP | Mean | Std. Error | 95% Wald Confidence Interval | |
|  |  |  |  | Lower | Upper |
| 1.00 | LOW_INTENSITY | 70.8500 | 4.73812 | 61.5635 | 80.1365 |
|  | MODERATE_INTENSITY | 70.0200 | 4.68209 | 60.8433 | 79.1967 |
|  | HIGH_INTENSITY | 69.4900 | 4.64529 | 60.3854 | 78.5946 |
|  | CONTROL | 70.1300 | 4.69133 | 60.9352 | 79.3248 |
| 2.00 | LOW_INTENSITY | 58.8277 | 4.13314 | 50.7269 | 66.9285 |
|  | MODERATE_INTENSITY | 46.8810 | 3.28626 | 40.4400 | 53.3219 |
|  | HIGH_INTENSITY | 65.7050 | 4.53504 | 56.8165 | 74.5935 |
|  | CONTROL | 68.5544 | 4.50538 | 59.7240 | 77.3848 |

| **Pairwise Comparisons** | | | | | | | |
| --- | --- | --- | --- | --- | --- | --- | --- |
| (I) TIME*GROUP | (J) TIME*GROUP | Mean Difference (I-J) | Std. Error | df | Sequential Bonferroni Sig. | 95% Wald Confidence Interval for Difference^a^ | |
|  |  |  |  |  |  | Lower | Upper |
| [TIME=1.00]*[GROUP=1.00] | [TIME=1.00]*[GROUP=2.00] | .8300 | 6.66121 | 1 | 1.000 | -12.5208 | 14.1808 |
|  | [TIME=1.00]*[GROUP=3.00] | 1.3600 | 6.63539 | 1 | 1.000 | -12.1411 | 14.8611 |
|  | [TIME=1.00]*[GROUP=4.00] | .7200 | 6.66771 | 1 | 1.000 | -12.6031 | 14.0431 |
|  | [TIME=2.00]*[GROUP=1.00] | 12.0223^b^ | 1.07880 | 1 | .000 | 8.6525 | 15.3922 |
|  | [TIME=2.00]*[GROUP=2.00] | 23.9690^b^ | 5.76622 | 1 | .001 | 6.0830 | 41.8550 |
|  | [TIME=2.00]*[GROUP=3.00] | 5.1450 | 6.55868 | 1 | 1.000 | -9.9180 | 20.2080 |
|  | [TIME=2.00]*[GROUP=4.00] | 2.2956 | 6.53821 | 1 | 1.000 | -11.3943 | 15.9854 |
| [TIME=1.00]*[GROUP=2.00] | [TIME=1.00]*[GROUP=1.00] | -.8300 | 6.66121 | 1 | 1.000 | -14.1808 | 12.5208 |
|  | [TIME=1.00]*[GROUP=3.00] | .5300 | 6.59550 | 1 | 1.000 | -12.5827 | 13.6427 |
|  | [TIME=1.00]*[GROUP=4.00] | -.1100 | 6.62801 | 1 | 1.000 | -13.1384 | 12.9184 |
|  | [TIME=2.00]*[GROUP=1.00] | 11.1923 | 6.24538 | 1 | 1.000 | -6.9594 | 29.3441 |
|  | [TIME=2.00]*[GROUP=2.00] | 23.1390^b^ | 1.55677 | 1 | .000 | 18.2928 | 27.9853 |
|  | [TIME=2.00]*[GROUP=3.00] | 4.3150 | 6.51832 | 1 | 1.000 | -10.2552 | 18.8853 |
|  | [TIME=2.00]*[GROUP=4.00] | 1.4656 | 6.49772 | 1 | 1.000 | -11.8076 | 14.7388 |
| [TIME=1.00]*[GROUP=3.00] | [TIME=1.00]*[GROUP=1.00] | -1.3600 | 6.63539 | 1 | 1.000 | -14.8611 | 12.1411 |
|  | [TIME=1.00]*[GROUP=2.00] | -.5300 | 6.59550 | 1 | 1.000 | -13.6427 | 12.5827 |
|  | [TIME=1.00]*[GROUP=4.00] | -.6400 | 6.60207 | 1 | 1.000 | -13.8053 | 12.5253 |
|  | [TIME=2.00]*[GROUP=1.00] | 10.6623 | 6.21784 | 1 | 1.000 | -7.0825 | 28.4072 |
|  | [TIME=2.00]*[GROUP=2.00] | 22.6090^b^ | 5.69019 | 1 | .002 | 5.0941 | 40.1239 |
|  | [TIME=2.00]*[GROUP=3.00] | 3.7850 | 1.31484 | 1 | .084 | -.2096 | 7.7796 |
|  | [TIME=2.00]*[GROUP=4.00] | .9356 | 6.47125 | 1 | 1.000 | -12.0826 | 13.9538 |
| [TIME=1.00]*[GROUP=4.00] | [TIME=1.00]*[GROUP=1.00] | -.7200 | 6.66771 | 1 | 1.000 | -14.0431 | 12.6031 |
|  | [TIME=1.00]*[GROUP=2.00] | .1100 | 6.62801 | 1 | 1.000 | -12.9184 | 13.1384 |
|  | [TIME=1.00]*[GROUP=3.00] | .6400 | 6.60207 | 1 | 1.000 | -12.5253 | 13.8053 |
|  | [TIME=2.00]*[GROUP=1.00] | 11.3023 | 6.25231 | 1 | 1.000 | -7.4003 | 30.0050 |
|  | [TIME=2.00]*[GROUP=2.00] | 23.2490^b^ | 5.72784 | 1 | .001 | 5.5487 | 40.9494 |
|  | [TIME=2.00]*[GROUP=3.00] | 4.4250 | 6.52496 | 1 | 1.000 | -10.2118 | 19.0619 |
|  | [TIME=2.00]*[GROUP=4.00] | 1.5756 | .63044 | 1 | .249 | -.3305 | 3.4816 |
| [TIME=2.00]*[GROUP=1.00] | [TIME=1.00]*[GROUP=1.00] | -12.0223^b^ | 1.07880 | 1 | .000 | -15.3922 | -8.6525 |
|  | [TIME=1.00]*[GROUP=2.00] | -11.1923 | 6.24538 | 1 | 1.000 | -29.3441 | 6.9594 |
|  | [TIME=1.00]*[GROUP=3.00] | -10.6623 | 6.21784 | 1 | 1.000 | -28.4072 | 7.0825 |
|  | [TIME=1.00]*[GROUP=4.00] | -11.3023 | 6.25231 | 1 | 1.000 | -30.0050 | 7.4003 |
|  | [TIME=2.00]*[GROUP=2.00] | 11.9467 | 5.28038 | 1 | .450 | -3.9356 | 27.8289 |
|  | [TIME=2.00]*[GROUP=3.00] | -6.8773 | 6.13591 | 1 | 1.000 | -22.0977 | 8.3431 |
|  | [TIME=2.00]*[GROUP=4.00] | -9.7268 | 6.11402 | 1 | 1.000 | -26.6711 | 7.2176 |
| [TIME=2.00]*[GROUP=2.00] | [TIME=1.00]*[GROUP=1.00] | -23.9690^b^ | 5.76622 | 1 | .001 | -41.8550 | -6.0830 |
|  | [TIME=1.00]*[GROUP=2.00] | -23.1390^b^ | 1.55677 | 1 | .000 | -27.9853 | -18.2928 |
|  | [TIME=1.00]*[GROUP=3.00] | -22.6090^b^ | 5.69019 | 1 | .002 | -40.1239 | -5.0941 |
|  | [TIME=1.00]*[GROUP=4.00] | -23.2490^b^ | 5.72784 | 1 | .001 | -40.9494 | -5.5487 |
|  | [TIME=2.00]*[GROUP=1.00] | -11.9467 | 5.28038 | 1 | .450 | -27.8289 | 3.9356 |
|  | [TIME=2.00]*[GROUP=3.00] | -18.8240^b^ | 5.60054 | 1 | .017 | -35.9172 | -1.7308 |
|  | [TIME=2.00]*[GROUP=4.00] | -21.6734^b^ | 5.57655 | 1 | .002 | -38.7677 | -4.5792 |
| [TIME=2.00]*[GROUP=3.00] | [TIME=1.00]*[GROUP=1.00] | -5.1450 | 6.55868 | 1 | 1.000 | -20.2080 | 9.9180 |
|  | [TIME=1.00]*[GROUP=2.00] | -4.3150 | 6.51832 | 1 | 1.000 | -18.8853 | 10.2552 |
|  | [TIME=1.00]*[GROUP=3.00] | -3.7850 | 1.31484 | 1 | .084 | -7.7796 | .2096 |
|  | [TIME=1.00]*[GROUP=4.00] | -4.4250 | 6.52496 | 1 | 1.000 | -19.0619 | 10.2118 |
|  | [TIME=2.00]*[GROUP=1.00] | 6.8773 | 6.13591 | 1 | 1.000 | -8.3431 | 22.0977 |
|  | [TIME=2.00]*[GROUP=2.00] | 18.8240^b^ | 5.60054 | 1 | .017 | 1.7308 | 35.9172 |
|  | [TIME=2.00]*[GROUP=4.00] | -2.8494 | 6.39257 | 1 | 1.000 | -16.4954 | 10.7965 |
| [TIME=2.00]*[GROUP=4.00] | [TIME=1.00]*[GROUP=1.00] | -2.2956 | 6.53821 | 1 | 1.000 | -15.9854 | 11.3943 |
|  | [TIME=1.00]*[GROUP=2.00] | -1.4656 | 6.49772 | 1 | 1.000 | -14.7388 | 11.8076 |
|  | [TIME=1.00]*[GROUP=3.00] | -.9356 | 6.47125 | 1 | 1.000 | -13.9538 | 12.0826 |
|  | [TIME=1.00]*[GROUP=4.00] | -1.5756 | .63044 | 1 | .249 | -3.4816 | .3305 |
|  | [TIME=2.00]*[GROUP=1.00] | 9.7268 | 6.11402 | 1 | 1.000 | -7.2176 | 26.6711 |
|  | [TIME=2.00]*[GROUP=2.00] | 21.6734^b^ | 5.57655 | 1 | .002 | 4.5792 | 38.7677 |
|  | [TIME=2.00]*[GROUP=3.00] | 2.8494 | 6.39257 | 1 | 1.000 | -10.7965 | 16.4954 |
| Pairwise comparisons of estimated marginal means based on the original scale of dependent variable Fat_intake | | | | | | | |
| a. Confidence interval bounds are approximate. | | | | | | | |
| b. The mean difference is significant at the .05 level. | | | | | | | |

| **Overall Test Results** | | |
| --- | --- | --- |
| Wald Chi-Square | df | Sig. |
| 666.527 | 7 | .000 |
| The Wald chi-square tests the effect of TIME*GROUP. This test is based on the linearly independent pairwise comparisons among the estimated marginal means. | | |

* Generalized Estimating Equations.

GENLIN carbohydrate_intake BY TIME GROUP (ORDER=ASCENDING)

/MODEL TIME GROUP TIME*GROUP INTERCEPT=YES

DISTRIBUTION=NORMAL LINK=IDENTITY

/CRITERIA SCALE=MLE PCONVERGE=1E-006(ABSOLUTE) SINGULAR=1E-012 ANALYSISTYPE=3(WALD) CILEVEL=95

LIKELIHOOD=FULL

/EMMEANS TABLES=TIME*GROUP SCALE=ORIGINAL COMPARE=TIME*GROUP CONTRAST=PAIRWISE

PADJUST=SEQBONFERRONI

/REPEATED SUBJECT=ID WITHINSUBJECT=TIME SORT=YES CORRTYPE=AR(1) ADJUSTCORR=YES COVB=ROBUST

MAXITERATIONS=100 PCONVERGE=1e-006(ABSOLUTE) UPDATECORR=1

/MISSING CLASSMISSING=EXCLUDE

/PRINT CPS DESCRIPTIVES MODELINFO FIT SUMMARY SOLUTION.

**Generalized Linear Models**

| **Notes** | | |
| --- | --- | --- |
| Output Created | | 09-JUN-2023 00:04:41 |
| Comments | |  |
| Input | Data | C:\Users\mdanaee\Google Drive\Data Analysis\Prof SOh students\Prof Soh -Wang\DATA GEEcr.sav |
|  | Active Dataset | DataSet1 |
|  | Filter | <none> |
|  | Weight | <none> |
|  | Split File | <none> |
|  | N of Rows in Working Data File | 144 |
| Missing Value Handling | Definition of Missing | User-defined missing values for factor, subject and within-subject variables are treated as missing. |
|  | Cases Used | Statistics are based on cases with valid data for all variables in the model. |
| Weight Handling | | not applicable |
| Syntax | | GENLIN carbohydrate_intake BY TIME GROUP (ORDER=ASCENDING)  /MODEL TIME GROUP TIME*GROUP INTERCEPT=YES  DISTRIBUTION=NORMAL LINK=IDENTITY  /CRITERIA SCALE=MLE PCONVERGE=1E-006(ABSOLUTE) SINGULAR=1E-012 ANALYSISTYPE=3(WALD) CILEVEL=95  LIKELIHOOD=FULL  /EMMEANS TABLES=TIME*GROUP SCALE=ORIGINAL COMPARE=TIME*GROUP CONTRAST=PAIRWISE  PADJUST=SEQBONFERRONI  /REPEATED SUBJECT=ID WITHINSUBJECT=TIME SORT=YES CORRTYPE=AR(1) ADJUSTCORR=YES COVB=ROBUST  MAXITERATIONS=100 PCONVERGE=1e-006(ABSOLUTE) UPDATECORR=1  /MISSING CLASSMISSING=EXCLUDE  /PRINT CPS DESCRIPTIVES MODELINFO FIT SUMMARY SOLUTION. |
| Resources | Processor Time | 00:00:00.14 |
|  | Elapsed Time | 00:00:00.12 |

| **Model Information** | | |
| --- | --- | --- |
| Dependent Variable | | carbohydrate_intake |
| Probability Distribution | | Normal |
| Link Function | | Identity |
| Subject Effect | 1 | ID |
| Within-Subject Effect | 1 | TIME |
| Working Correlation Matrix Structure | | AR(1) |

| **Case Processing Summary** | | |
| --- | --- | --- |
|  | N | Percent |
| Included | 137 | 95.1% |
| Excluded | 7 | 4.9% |
| Total | 144 | 100.0% |

| **Correlated Data Summary** | | | |
| --- | --- | --- | --- |
| Number of Levels | Subject Effect | ID | 72 |
|  | Within-Subject Effect | TIME | 2 |
| Number of Subjects | | | 72 |
| Number of Measurements per Subject | Minimum | | 1 |
|  | Maximum | | 2 |
| Correlation Matrix Dimension | | | 2 |

| **Categorical Variable Information** | | | | |
| --- | --- | --- | --- | --- |
|  | | | N | Percent |
| Factor | TIME | 1.00 | 72 | 52.6% |
|  |  | 2.00 | 65 | 47.4% |
|  |  | Total | 137 | 100.0% |
|  | GROUP | LOW_INTENSITY | 33 | 24.1% |
|  |  | MODERATE_INTENSITY | 35 | 25.5% |
|  |  | HIGH_INTENSITY | 34 | 24.8% |
|  |  | CONTROL | 35 | 25.5% |
|  |  | Total | 137 | 100.0% |

| **Continuous Variable Information** | | | | | | |
| --- | --- | --- | --- | --- | --- | --- |
|  | | N | Minimum | Maximum | Mean | Std. Deviation |
| Dependent Variable | carbohydrate_intake | 137 | 133.48 | 393.79 | 270.8434 | 67.91035 |

| **Goodness of Fit^a^** | |
| --- | --- |
|  | Value |
| Quasi Likelihood under Independence Model Criterion (QIC)^b^ | 422127.522 |
| Corrected Quasi Likelihood under Independence Model Criterion (QICC)^b^ | 422127.409 |
| Dependent Variable: carbohydrate_intake  Model: (Intercept), TIME, GROUP, TIME * GROUP^a^ | |
| a. Information criteria are in smaller-is-better form. | |
| b. Computed using the full log quasi-likelihood function. | |

| **Tests of Model Effects** | | | |
| --- | --- | --- | --- |
| Source | Type III | | |
|  | Wald Chi-Square | df | Sig. |
| (Intercept) | 1734.108 | 1 | .000 |
| TIME | 585.466 | 1 | .000 |
| GROUP | 5.112 | 3 | .164 |
| TIME * GROUP | 334.607 | 3 | .000 |
| Dependent Variable: carbohydrate_intake  Model: (Intercept), TIME, GROUP, TIME * GROUP | | | |

| **Parameter Estimates** | | | | | | | |
| --- | --- | --- | --- | --- | --- | --- | --- |
| Parameter | B | Std. Error | 95% Wald Confidence Interval | | Hypothesis Test | | |
|  |  |  | Lower | Upper | Wald Chi-Square | df | Sig. |
| (Intercept) | 290.394 | 13.9578 | 263.037 | 317.751 | 432.857 | 1 | .000 |
| [TIME=1.00] | 10.646 | 2.0697 | 6.589 | 14.702 | 26.459 | 1 | .000 |
| [TIME=2.00] | 0^a^ | . | . | . | . | . | . |
| [GROUP=1.00] | -30.387 | 19.0181 | -67.662 | 6.887 | 2.553 | 1 | .110 |
| [GROUP=2.00] | -69.794 | 17.6790 | -104.444 | -35.144 | 15.586 | 1 | .000 |
| [GROUP=3.00] | -80.261 | 17.6566 | -114.867 | -45.655 | 20.663 | 1 | .000 |
| [GROUP=4.00] | 0^a^ | . | . | . | . | . | . |
| [TIME=1.00] * [GROUP=1.00] | 25.797 | 4.1622 | 17.640 | 33.955 | 38.416 | 1 | .000 |
| [TIME=1.00] * [GROUP=2.00] | 71.314 | 4.5590 | 62.378 | 80.250 | 244.682 | 1 | .000 |
| [TIME=1.00] * [GROUP=3.00] | 83.851 | 7.4709 | 69.208 | 98.494 | 125.972 | 1 | .000 |
| [TIME=1.00] * [GROUP=4.00] | 0^a^ | . | . | . | . | . | . |
| [TIME=2.00] * [GROUP=1.00] | 0^a^ | . | . | . | . | . | . |
| [TIME=2.00] * [GROUP=2.00] | 0^a^ | . | . | . | . | . | . |
| [TIME=2.00] * [GROUP=3.00] | 0^a^ | . | . | . | . | . | . |
| [TIME=2.00] * [GROUP=4.00] | 0^a^ | . | . | . | . | . | . |
| (Scale) | 3272.181 |  |  |  |  |  |  |
| Dependent Variable: carbohydrate_intake  Model: (Intercept), TIME, GROUP, TIME * GROUP | | | | | | | |
| a. Set to zero because this parameter is redundant. | | | | | | | |

**Estimated Marginal Means: TIME* GROUP**

| **Estimates** | | | | | |
| --- | --- | --- | --- | --- | --- |
| TIME | GROUP | Mean | Std. Error | 95% Wald Confidence Interval | |
|  |  |  |  | Lower | Upper |
| 1.00 | LOW_INTENSITY | 296.4500 | 14.06802 | 268.8772 | 324.0228 |
|  | MODERATE_INTENSITY | 302.5600 | 14.36076 | 274.4134 | 330.7066 |
|  | HIGH_INTENSITY | 304.6300 | 14.45807 | 276.2927 | 332.9673 |
|  | CONTROL | 301.0400 | 14.51685 | 272.5875 | 329.4925 |
| 2.00 | LOW_INTENSITY | 260.0066 | 12.91781 | 234.6882 | 285.3251 |
|  | MODERATE_INTENSITY | 220.6001 | 10.85022 | 199.3341 | 241.8661 |
|  | HIGH_INTENSITY | 210.1330 | 10.81373 | 188.9385 | 231.3276 |
|  | CONTROL | 290.3941 | 13.95776 | 263.0374 | 317.7508 |

| **Pairwise Comparisons** | | | | | | | |
| --- | --- | --- | --- | --- | --- | --- | --- |
| (I) TIME*GROUP | (J) TIME*GROUP | Mean Difference (I-J) | Std. Error | df | Sequential Bonferroni Sig. | 95% Wald Confidence Interval for Difference^a^ | |
|  |  |  |  |  |  | Lower | Upper |
| [TIME=1.00]*[GROUP=1.00] | [TIME=1.00]*[GROUP=2.00] | -6.1100 | 20.10325 | 1 | 1.000 | -47.8085 | 35.5885 |
|  | [TIME=1.00]*[GROUP=3.00] | -8.1800 | 20.17288 | 1 | 1.000 | -50.8870 | 34.5270 |
|  | [TIME=1.00]*[GROUP=4.00] | -4.5900 | 20.21504 | 1 | 1.000 | -45.8962 | 36.7162 |
|  | [TIME=2.00]*[GROUP=1.00] | 36.4434^b^ | 3.61112 | 1 | .000 | 25.1632 | 47.7236 |
|  | [TIME=2.00]*[GROUP=2.00] | 75.8499^b^ | 17.76616 | 1 | .000 | 22.7057 | 128.9941 |
|  | [TIME=2.00]*[GROUP=3.00] | 86.3170^b^ | 17.74390 | 1 | .000 | 32.1614 | 140.4725 |
|  | [TIME=2.00]*[GROUP=4.00] | 6.0559 | 19.81737 | 1 | 1.000 | -35.0631 | 47.1749 |
| [TIME=1.00]*[GROUP=2.00] | [TIME=1.00]*[GROUP=1.00] | 6.1100 | 20.10325 | 1 | 1.000 | -35.5885 | 47.8085 |
|  | [TIME=1.00]*[GROUP=3.00] | -2.0700 | 20.37811 | 1 | 1.000 | -42.7409 | 38.6009 |
|  | [TIME=1.00]*[GROUP=4.00] | 1.5200 | 20.41985 | 1 | 1.000 | -39.0339 | 42.0739 |
|  | [TIME=2.00]*[GROUP=1.00] | 42.5534 | 19.31584 | 1 | .359 | -13.2793 | 98.3860 |
|  | [TIME=2.00]*[GROUP=2.00] | 81.9599^b^ | 4.06219 | 1 | .000 | 69.3142 | 94.6056 |
|  | [TIME=2.00]*[GROUP=3.00] | 92.4270^b^ | 17.97688 | 1 | .000 | 36.9411 | 147.9128 |
|  | [TIME=2.00]*[GROUP=4.00] | 12.1659 | 20.02625 | 1 | 1.000 | -32.0717 | 56.4035 |
| [TIME=1.00]*[GROUP=3.00] | [TIME=1.00]*[GROUP=1.00] | 8.1800 | 20.17288 | 1 | 1.000 | -34.5270 | 50.8870 |
|  | [TIME=1.00]*[GROUP=2.00] | 2.0700 | 20.37811 | 1 | 1.000 | -38.6009 | 42.7409 |
|  | [TIME=1.00]*[GROUP=4.00] | 3.5900 | 20.48840 | 1 | 1.000 | -37.8636 | 45.0436 |
|  | [TIME=2.00]*[GROUP=1.00] | 44.6234 | 19.38829 | 1 | .299 | -11.8688 | 101.1156 |
|  | [TIME=2.00]*[GROUP=2.00] | 84.0299^b^ | 18.07659 | 1 | .000 | 29.1119 | 138.9479 |
|  | [TIME=2.00]*[GROUP=3.00] | 94.4970^b^ | 7.17849 | 1 | .000 | 72.2303 | 116.7636 |
|  | [TIME=2.00]*[GROUP=4.00] | 14.2359 | 20.09614 | 1 | 1.000 | -31.1446 | 59.6164 |
| [TIME=1.00]*[GROUP=4.00] | [TIME=1.00]*[GROUP=1.00] | 4.5900 | 20.21504 | 1 | 1.000 | -36.7162 | 45.8962 |
|  | [TIME=1.00]*[GROUP=2.00] | -1.5200 | 20.41985 | 1 | 1.000 | -42.0739 | 39.0339 |
|  | [TIME=1.00]*[GROUP=3.00] | -3.5900 | 20.48840 | 1 | 1.000 | -45.0436 | 37.8636 |
|  | [TIME=2.00]*[GROUP=1.00] | 41.0334 | 19.43216 | 1 | .417 | -14.6448 | 96.7116 |
|  | [TIME=2.00]*[GROUP=2.00] | 80.4399^b^ | 18.12364 | 1 | .000 | 25.9279 | 134.9519 |
|  | [TIME=2.00]*[GROUP=3.00] | 90.9070^b^ | 18.10181 | 1 | .000 | 35.4180 | 146.3959 |
|  | [TIME=2.00]*[GROUP=4.00] | 10.6459^b^ | 2.06966 | 1 | .000 | 4.2502 | 17.0416 |
| [TIME=2.00]*[GROUP=1.00] | [TIME=1.00]*[GROUP=1.00] | -36.4434^b^ | 3.61112 | 1 | .000 | -47.7236 | -25.1632 |
|  | [TIME=1.00]*[GROUP=2.00] | -42.5534 | 19.31584 | 1 | .359 | -98.3860 | 13.2793 |
|  | [TIME=1.00]*[GROUP=3.00] | -44.6234 | 19.38829 | 1 | .299 | -101.1156 | 11.8688 |
|  | [TIME=1.00]*[GROUP=4.00] | -41.0334 | 19.43216 | 1 | .417 | -96.7116 | 14.6448 |
|  | [TIME=2.00]*[GROUP=2.00] | 39.4065 | 16.87001 | 1 | .292 | -10.1103 | 88.9234 |
|  | [TIME=2.00]*[GROUP=3.00] | 49.8736^b^ | 16.84656 | 1 | .049 | .0892 | 99.6580 |
|  | [TIME=2.00]*[GROUP=4.00] | -30.3875 | 19.01812 | 1 | 1.000 | -84.3532 | 23.5783 |
| [TIME=2.00]*[GROUP=2.00] | [TIME=1.00]*[GROUP=1.00] | -75.8499^b^ | 17.76616 | 1 | .000 | -128.9941 | -22.7057 |
|  | [TIME=1.00]*[GROUP=2.00] | -81.9599^b^ | 4.06219 | 1 | .000 | -94.6056 | -69.3142 |
|  | [TIME=1.00]*[GROUP=3.00] | -84.0299^b^ | 18.07659 | 1 | .000 | -138.9479 | -29.1119 |
|  | [TIME=1.00]*[GROUP=4.00] | -80.4399^b^ | 18.12364 | 1 | .000 | -134.9519 | -25.9279 |
|  | [TIME=2.00]*[GROUP=1.00] | -39.4065 | 16.87001 | 1 | .292 | -88.9234 | 10.1103 |
|  | [TIME=2.00]*[GROUP=3.00] | 10.4671 | 15.31875 | 1 | 1.000 | -23.9347 | 44.8688 |
|  | [TIME=2.00]*[GROUP=4.00] | -69.7940^b^ | 17.67898 | 1 | .001 | -122.3681 | -17.2199 |
| [TIME=2.00]*[GROUP=3.00] | [TIME=1.00]*[GROUP=1.00] | -86.3170^b^ | 17.74390 | 1 | .000 | -140.4725 | -32.1614 |
|  | [TIME=1.00]*[GROUP=2.00] | -92.4270^b^ | 17.97688 | 1 | .000 | -147.9128 | -36.9411 |
|  | [TIME=1.00]*[GROUP=3.00] | -94.4970^b^ | 7.17849 | 1 | .000 | -116.7636 | -72.2303 |
|  | [TIME=1.00]*[GROUP=4.00] | -90.9070^b^ | 18.10181 | 1 | .000 | -146.3959 | -35.4180 |
|  | [TIME=2.00]*[GROUP=1.00] | -49.8736^b^ | 16.84656 | 1 | .049 | -99.6580 | -.0892 |
|  | [TIME=2.00]*[GROUP=2.00] | -10.4671 | 15.31875 | 1 | 1.000 | -44.8688 | 23.9347 |
|  | [TIME=2.00]*[GROUP=4.00] | -80.2611^b^ | 17.65661 | 1 | .000 | -133.6430 | -26.8791 |
| [TIME=2.00]*[GROUP=4.00] | [TIME=1.00]*[GROUP=1.00] | -6.0559 | 19.81737 | 1 | 1.000 | -47.1749 | 35.0631 |
|  | [TIME=1.00]*[GROUP=2.00] | -12.1659 | 20.02625 | 1 | 1.000 | -56.4035 | 32.0717 |
|  | [TIME=1.00]*[GROUP=3.00] | -14.2359 | 20.09614 | 1 | 1.000 | -59.6164 | 31.1446 |
|  | [TIME=1.00]*[GROUP=4.00] | -10.6459^b^ | 2.06966 | 1 | .000 | -17.0416 | -4.2502 |
|  | [TIME=2.00]*[GROUP=1.00] | 30.3875 | 19.01812 | 1 | 1.000 | -23.5783 | 84.3532 |
|  | [TIME=2.00]*[GROUP=2.00] | 69.7940^b^ | 17.67898 | 1 | .001 | 17.2199 | 122.3681 |
|  | [TIME=2.00]*[GROUP=3.00] | 80.2611^b^ | 17.65661 | 1 | .000 | 26.8791 | 133.6430 |
| Pairwise comparisons of estimated marginal means based on the original scale of dependent variable carbohydrate_intake | | | | | | | |
| a. Confidence interval bounds are approximate. | | | | | | | |
| b. The mean difference is significant at the .05 level. | | | | | | | |

| **Overall Test Results** | | |
| --- | --- | --- |
| Wald Chi-Square | df | Sig. |
| 1102.692 | 7 | .000 |
| The Wald chi-square tests the effect of TIME*GROUP. This test is based on the linearly independent pairwise comparisons among the estimated marginal means. | | |

**HOMOGENITY BACKGROUND**

ONEWAY menstrual_time BY GROUP

/STATISTICS DESCRIPTIVES HOMOGENEITY

/MISSING ANALYSIS

/CRITERIA=CILEVEL(0.95).

**Oneway**

| **Notes** | | |
| --- | --- | --- |
| Output Created | | 12-APR-2025 20:36:17 |
| Comments | |  |
| Input | Data | C:\Users\user\Desktop\dana教写文章第一篇\4,5,6st, dana要求的两篇论文\Supplementary material\DATA nutritional intake-revised.sav |
|  | Active Dataset | DataSet2 |
|  | Filter | <none> |
|  | Weight | <none> |
|  | Split File | <none> |
|  | N of Rows in Working Data File | 144 |
| Missing Value Handling | Definition of Missing | User-defined missing values are treated as missing. |
|  | Cases Used | Statistics for each analysis are based on cases with no missing data for any variable in the analysis. |
| Syntax | | ONEWAY menstrual_time BY GROUP  /STATISTICS DESCRIPTIVES HOMOGENEITY  /MISSING ANALYSIS  /CRITERIA=CILEVEL(0.95). |
| Resources | Processor Time | 00:00:00.05 |
|  | Elapsed Time | 00:00:00.08 |

| **Descriptives** | | | | | | | | |
| --- | --- | --- | --- | --- | --- | --- | --- | --- |
| menstrual_time | | | | | | | | |
|  | N | Mean | Std. Deviation | Std. Error | 95% Confidence Interval for Mean | | Minimum | Maximum |
|  |  |  |  |  | Lower Bound | Upper Bound |  |  |
| LOW_INTENSITY | 18 | 13.5556 | 7.04792 | 1.66121 | 10.0507 | 17.0604 | 3.00 | 26.00 |
| MODERATE_INTENSITY | 18 | 13.7778 | 6.30178 | 1.48534 | 10.6440 | 16.9116 | 4.00 | 24.00 |
| HIGH_INTENSITY | 18 | 14.7778 | 7.40870 | 1.74625 | 11.0935 | 18.4620 | 3.00 | 28.00 |
| CONTROL | 18 | 14.2222 | 7.61234 | 1.79425 | 10.4367 | 18.0077 | 2.00 | 29.00 |
| Total | 72 | 14.0833 | 6.97430 | .82193 | 12.4445 | 15.7222 | 2.00 | 29.00 |

| **Tests of Homogeneity of Variances** | | | | | |
| --- | --- | --- | --- | --- | --- |
|  | | Levene Statistic | df1 | df2 | Sig. |
| menstrual_time | Based on Mean | .159 | 3 | 68 | .923 |
|  | Based on Median | .180 | 3 | 68 | .909 |
|  | Based on Median and with adjusted df | .180 | 3 | 66.045 | .909 |
|  | Based on trimmed mean | .152 | 3 | 68 | .928 |

| **ANOVA** | | | | | |
| --- | --- | --- | --- | --- | --- |
| menstrual_time | | | | | |
|  | Sum of Squares | df | Mean Square | F | Sig. |
| Between Groups | 15.722 | 3 | 5.241 | .104 | .958 |
| Within Groups | 3437.778 | 68 | 50.556 |  |  |
| Total | 3453.500 | 71 |  |  |  |

ONEWAY menstrual_cycle BY GROUP

/STATISTICS DESCRIPTIVES HOMOGENEITY

/MISSING ANALYSIS

/CRITERIA=CILEVEL(0.95).

**Oneway**

| **Notes** | | |
| --- | --- | --- |
| Output Created | | 14-APR-2025 21:05:09 |
| Comments | |  |
| Input | Data | C:\Users\user\Desktop\dana教写文章第一篇\4,5,6st, dana要求的两篇论文\Supplementary material\DATA nutritional intake-revised.sav |
|  | Active Dataset | DataSet2 |
|  | Filter | <none> |
|  | Weight | <none> |
|  | Split File | <none> |
|  | N of Rows in Working Data File | 144 |
| Missing Value Handling | Definition of Missing | User-defined missing values are treated as missing. |
|  | Cases Used | Statistics for each analysis are based on cases with no missing data for any variable in the analysis. |
| Syntax | | ONEWAY menstrual_cycle BY GROUP  /STATISTICS DESCRIPTIVES HOMOGENEITY  /MISSING ANALYSIS  /CRITERIA=CILEVEL(0.95). |
| Resources | Processor Time | 00:00:00.00 |
|  | Elapsed Time | 00:00:00.01 |

| **Descriptives** | | | | | | | | |
| --- | --- | --- | --- | --- | --- | --- | --- | --- |
| menstrual_cycle | | | | | | | | |
|  | N | Mean | Std. Deviation | Std. Error | 95% Confidence Interval for Mean | | Minimum | Maximum |
|  |  |  |  |  | Lower Bound | Upper Bound |  |  |
| LOW_INTENSITY | 18 | 31.2222 | 2.15722 | .50846 | 30.1495 | 32.2950 | 28.00 | 35.00 |
| MODERATE_INTENSITY | 18 | 31.3333 | 2.02920 | .47829 | 30.3242 | 32.3424 | 27.00 | 35.00 |
| HIGH_INTENSITY | 18 | 31.5556 | 2.00653 | .47294 | 30.5577 | 32.5534 | 27.00 | 35.00 |
| CONTROL | 18 | 31.8889 | 1.93691 | .45653 | 30.9257 | 32.8521 | 28.00 | 35.00 |
| Total | 72 | 31.5000 | 2.00703 | .23653 | 31.0284 | 31.9716 | 27.00 | 35.00 |

| **Tests of Homogeneity of Variances** | | | | | |
| --- | --- | --- | --- | --- | --- |
|  | | Levene Statistic | df1 | df2 | Sig. |
| menstrual_cycle | Based on Mean | .262 | 3 | 68 | .852 |
|  | Based on Median | .269 | 3 | 68 | .848 |
|  | Based on Median and with adjusted df | .269 | 3 | 66.568 | .848 |
|  | Based on trimmed mean | .271 | 3 | 68 | .846 |

| **ANOVA** | | | | | |
| --- | --- | --- | --- | --- | --- |
| menstrual_cycle | | | | | |
|  | Sum of Squares | df | Mean Square | F | Sig. |
| Between Groups | 4.667 | 3 | 1.556 | .376 | .771 |
| Within Groups | 281.333 | 68 | 4.137 |  |  |
| Total | 286.000 | 71 |  |  |  |

ONEWAY height weight age depression anxiety stress mealtime BY GROUP

/STATISTICS DESCRIPTIVES HOMOGENEITY

/MISSING ANALYSIS.

**Oneway**

| **Notes** | | |
| --- | --- | --- |
| Output Created | | 23-MAY-2023 21:04:53 |
| Comments | |  |
| Input | Data | C:\Users\mdanaee\Google Drive\Data Analysis\Prof SOh students\Prof Soh -Wang\DATA1.sav |
|  | Active Dataset | DataSet1 |
|  | Filter | <none> |
|  | Weight | <none> |
|  | Split File | <none> |
|  | N of Rows in Working Data File | 72 |
| Missing Value Handling | Definition of Missing | User-defined missing values are treated as missing. |
|  | Cases Used | Statistics for each analysis are based on cases with no missing data for any variable in the analysis. |
| Syntax | | ONEWAY height weight age depression anxiety stress mealtime BY GROUP  /STATISTICS DESCRIPTIVES HOMOGENEITY  /MISSING ANALYSIS. |
| Resources | Processor Time | 00:00:00.02 |
|  | Elapsed Time | 00:00:00.02 |

| **Descriptives** | | | | | | | | | |
| --- | --- | --- | --- | --- | --- | --- | --- | --- | --- |
|  | | N | Mean | Std. Deviation | Std. Error | 95% Confidence Interval for Mean | | Minimum | Maximum |
|  |  |  |  |  |  | Lower Bound | Upper Bound |  |  |
| height | LOW_INTENSITY | 18 | 1.6000 | .03850 | .00907 | 1.5809 | 1.6191 | 1.53 | 1.67 |
|  | MODERATE_INTENSITY | 18 | 1.5900 | .03646 | .00859 | 1.5719 | 1.6081 | 1.53 | 1.65 |
|  | HIGH_INTENSITY | 18 | 1.6000 | .03850 | .00907 | 1.5809 | 1.6191 | 1.53 | 1.67 |
|  | Control | 18 | 1.6000 | .03850 | .00907 | 1.5809 | 1.6191 | 1.53 | 1.67 |
|  | Total | 72 | 1.5975 | .03744 | .00441 | 1.5887 | 1.6063 | 1.53 | 1.67 |
| weight | LOW_INTENSITY | 18 | 73.5700 | 6.62233 | 1.56090 | 70.2768 | 76.8632 | 60.59 | 86.55 |
|  | MODERATE_INTENSITY | 18 | 72.6600 | 6.53390 | 1.54005 | 69.4108 | 75.9092 | 61.74 | 83.58 |
|  | HIGH_INTENSITY | 18 | 73.5600 | 6.61503 | 1.55918 | 70.2704 | 76.8496 | 62.49 | 84.63 |
|  | Control | 18 | 73.3400 | 6.60300 | 1.55634 | 70.0564 | 76.6236 | 60.66 | 86.02 |
|  | Total | 72 | 73.2825 | 6.46366 | .76175 | 71.7636 | 74.8014 | 60.59 | 86.55 |
| age | LOW_INTENSITY | 18 | 18.7744 | .42332 | .09978 | 18.5639 | 18.9850 | 18.11 | 19.61 |
|  | MODERATE_INTENSITY | 18 | 18.9700 | .42924 | .10117 | 18.7565 | 19.1835 | 18.11 | 19.72 |
|  | HIGH_INTENSITY | 18 | 19.0411 | .43194 | .10181 | 18.8263 | 19.2559 | 18.34 | 19.61 |
|  | Control | 18 | 18.9306 | .42947 | .10123 | 18.7170 | 19.1441 | 18.21 | 19.64 |
|  | Total | 72 | 18.9290 | .43073 | .05076 | 18.8278 | 19.0302 | 18.11 | 19.72 |
| depression | LOW_INTENSITY | 18 | 7.33 | 3.757 | .886 | 5.46 | 9.20 | 2 | 14 |
|  | MODERATE_INTENSITY | 18 | 8.11 | 4.129 | .973 | 6.06 | 10.16 | 2 | 15 |
|  | HIGH_INTENSITY | 18 | 7.72 | 3.923 | .925 | 5.77 | 9.67 | 2 | 15 |
|  | Control | 18 | 7.56 | 3.838 | .905 | 5.65 | 9.46 | 2 | 14 |
|  | Total | 72 | 7.68 | 3.841 | .453 | 6.78 | 8.58 | 2 | 15 |
| anxiety | LOW_INTENSITY | 18 | 13.33 | 6.020 | 1.419 | 10.34 | 16.33 | 4 | 23 |
|  | MODERATE_INTENSITY | 18 | 12.61 | 5.689 | 1.341 | 9.78 | 15.44 | 4 | 22 |
|  | HIGH_INTENSITY | 18 | 12.50 | 5.659 | 1.334 | 9.69 | 15.31 | 4 | 21 |
|  | Control | 18 | 12.89 | 5.799 | 1.367 | 10.00 | 15.77 | 5 | 22 |
|  | Total | 72 | 12.83 | 5.679 | .669 | 11.50 | 14.17 | 4 | 23 |
| stress | LOW_INTENSITY | 18 | 16.39 | 6.853 | 1.615 | 12.98 | 19.80 | 4 | 26 |
|  | MODERATE_INTENSITY | 18 | 15.61 | 6.527 | 1.538 | 12.37 | 18.86 | 5 | 25 |
|  | HIGH_INTENSITY | 18 | 16.06 | 6.690 | 1.577 | 12.73 | 19.38 | 4 | 26 |
|  | Control | 18 | 15.94 | 6.655 | 1.569 | 12.63 | 19.25 | 4 | 27 |
|  | Total | 72 | 16.00 | 6.546 | .771 | 14.46 | 17.54 | 4 | 27 |
| mealtime | LOW_INTENSITY | 18 | 42.1694 | 6.77060 | 1.59585 | 38.8025 | 45.5364 | 29.48 | 52.62 |
|  | MODERATE_INTENSITY | 18 | 43.7622 | 7.02904 | 1.65676 | 40.2668 | 47.2577 | 33.36 | 57.75 |
|  | HIGH_INTENSITY | 18 | 41.9728 | 6.73760 | 1.58807 | 38.6223 | 45.3233 | 32.75 | 55.23 |
|  | Control | 18 | 42.4922 | 6.83086 | 1.61005 | 39.0953 | 45.8891 | 32.11 | 53.97 |
|  | Total | 72 | 42.5992 | 6.73347 | .79355 | 41.0169 | 44.1815 | 29.48 | 57.75 |

| **Test of Homogeneity of Variances** | | | | | |
| --- | --- | --- | --- | --- | --- |
|  | | Levene Statistic | df1 | df2 | Sig. |
| height | Based on Mean | .010 | 3 | 68 | .999 |
|  | Based on Median | .010 | 3 | 68 | .999 |
|  | Based on Median and with adjusted df | .010 | 3 | 67.716 | .999 |
|  | Based on trimmed mean | .010 | 3 | 68 | .999 |
| weight | Based on Mean | .172 | 3 | 68 | .915 |
|  | Based on Median | .172 | 3 | 68 | .915 |
|  | Based on Median and with adjusted df | .172 | 3 | 64.247 | .915 |
|  | Based on trimmed mean | .172 | 3 | 68 | .915 |
| age | Based on Mean | .088 | 3 | 68 | .966 |
|  | Based on Median | .054 | 3 | 68 | .983 |
|  | Based on Median and with adjusted df | .054 | 3 | 67.826 | .983 |
|  | Based on trimmed mean | .085 | 3 | 68 | .968 |
| depression | Based on Mean | .145 | 3 | 68 | .932 |
|  | Based on Median | .148 | 3 | 68 | .930 |
|  | Based on Median and with adjusted df | .148 | 3 | 67.446 | .930 |
|  | Based on trimmed mean | .144 | 3 | 68 | .933 |
| anxiety | Based on Mean | .072 | 3 | 68 | .975 |
|  | Based on Median | .069 | 3 | 68 | .976 |
|  | Based on Median and with adjusted df | .069 | 3 | 67.929 | .976 |
|  | Based on trimmed mean | .072 | 3 | 68 | .975 |
| stress | Based on Mean | .094 | 3 | 68 | .963 |
|  | Based on Median | .070 | 3 | 68 | .976 |
|  | Based on Median and with adjusted df | .070 | 3 | 66.892 | .976 |
|  | Based on trimmed mean | .089 | 3 | 68 | .966 |
| mealtime | Based on Mean | .015 | 3 | 68 | .997 |
|  | Based on Median | .010 | 3 | 68 | .999 |
|  | Based on Median and with adjusted df | .010 | 3 | 65.991 | .999 |
|  | Based on trimmed mean | .015 | 3 | 68 | .997 |

| **ANOVA** | | | | | | |
| --- | --- | --- | --- | --- | --- | --- |
|  | | Sum of Squares | df | Mean Square | F | Sig. |
| height | Between Groups | .001 | 3 | .000 | .312 | .817 |
|  | Within Groups | .098 | 68 | .001 |  |  |
|  | Total | .100 | 71 |  |  |  |
| weight | Between Groups | 9.909 | 3 | 3.303 | .076 | .973 |
|  | Within Groups | 2956.389 | 68 | 43.476 |  |  |
|  | Total | 2966.298 | 71 |  |  |  |
| age | Between Groups | .687 | 3 | .229 | 1.246 | .300 |
|  | Within Groups | 12.486 | 68 | .184 |  |  |
|  | Total | 13.172 | 71 |  |  |  |
| depression | Between Groups | 5.819 | 3 | 1.940 | .127 | .944 |
|  | Within Groups | 1041.833 | 68 | 15.321 |  |  |
|  | Total | 1047.653 | 71 |  |  |  |
| anxiety | Between Groups | 7.444 | 3 | 2.481 | .074 | .974 |
|  | Within Groups | 2282.556 | 68 | 33.567 |  |  |
|  | Total | 2290.000 | 71 |  |  |  |
| stress | Between Groups | 5.556 | 3 | 1.852 | .041 | .989 |
|  | Within Groups | 3036.444 | 68 | 44.654 |  |  |
|  | Total | 3042.000 | 71 |  |  |  |
| mealtime | Between Groups | 34.941 | 3 | 11.647 | .249 | .862 |
|  | Within Groups | 3184.172 | 68 | 46.826 |  |  |
|  | Total | 3219.113 | 71 |  |  |  |

**NORMALITY BACKGROUND**

EXAMINE VARIABLES=menstrual_time BY GROUP

/PLOT BOXPLOT STEMLEAF NPPLOT

/COMPARE GROUPS

/STATISTICS DESCRIPTIVES

/CINTERVAL 95

/MISSING LISTWISE

/NOTOTAL.

**Explore**

| **Notes** | | |
| --- | --- | --- |
| Output Created | | 12-APR-2025 20:49:08 |
| Comments | |  |
| Input | Data | C:\Users\user\Desktop\dana教写文章第一篇\4,5,6st, dana要求的两篇论文\Supplementary material\DATA nutritional intake-revised.sav |
|  | Active Dataset | DataSet2 |
|  | Filter | <none> |
|  | Weight | <none> |
|  | Split File | <none> |
|  | N of Rows in Working Data File | 144 |
| Missing Value Handling | Definition of Missing | User-defined missing values for dependent variables are treated as missing. |
|  | Cases Used | Statistics are based on cases with no missing values for any dependent variable or factor used. |
| Syntax | | EXAMINE VARIABLES=menstrual_time BY GROUP  /PLOT BOXPLOT STEMLEAF NPPLOT  /COMPARE GROUPS  /STATISTICS DESCRIPTIVES  /CINTERVAL 95  /MISSING LISTWISE  /NOTOTAL. |
| Resources | Processor Time | 00:00:01.56 |
|  | Elapsed Time | 00:00:01.36 |

**GROUP**

| **Case Processing Summary** | | | | | | | |
| --- | --- | --- | --- | --- | --- | --- | --- |
|  | GROUP | Cases | | | | | |
|  |  | Valid | | Missing | | Total | |
|  |  | N | Percent | N | Percent | N | Percent |
| menstrual_time | LOW_INTENSITY | 18 | 50.0% | 18 | 50.0% | 36 | 100.0% |
|  | MODERATE_INTENSITY | 18 | 50.0% | 18 | 50.0% | 36 | 100.0% |
|  | HIGH_INTENSITY | 18 | 50.0% | 18 | 50.0% | 36 | 100.0% |
|  | CONTROL | 18 | 50.0% | 18 | 50.0% | 36 | 100.0% |

| **Descriptives** | | | | | |
| --- | --- | --- | --- | --- | --- |
|  | GROUP | | | Statistic | Std. Error |
| menstrual_time | LOW_INTENSITY | Mean | | 13.5556 | 1.66121 |
|  |  | 95% Confidence Interval for Mean | Lower Bound | 10.0507 |  |
|  |  |  | Upper Bound | 17.0604 |  |
|  |  | 5% Trimmed Mean | | 13.4506 |  |
|  |  | Median | | 12.0000 |  |
|  |  | Variance | | 49.673 |  |
|  |  | Std. Deviation | | 7.04792 |  |
|  |  | Minimum | | 3.00 |  |
|  |  | Maximum | | 26.00 |  |
|  |  | Range | | 23.00 |  |
|  |  | Interquartile Range | | 12.50 |  |
|  |  | Skewness | | .242 | .536 |
|  |  | Kurtosis | | -1.179 | 1.038 |
|  | MODERATE_INTENSITY | Mean | | 13.7778 | 1.48534 |
|  |  | 95% Confidence Interval for Mean | Lower Bound | 10.6440 |  |
|  |  |  | Upper Bound | 16.9116 |  |
|  |  | 5% Trimmed Mean | | 13.7531 |  |
|  |  | Median | | 15.0000 |  |
|  |  | Variance | | 39.712 |  |
|  |  | Std. Deviation | | 6.30178 |  |
|  |  | Minimum | | 4.00 |  |
|  |  | Maximum | | 24.00 |  |
|  |  | Range | | 20.00 |  |
|  |  | Interquartile Range | | 12.25 |  |
|  |  | Skewness | | .039 | .536 |
|  |  | Kurtosis | | -1.293 | 1.038 |
|  | HIGH_INTENSITY | Mean | | 14.7778 | 1.74625 |
|  |  | 95% Confidence Interval for Mean | Lower Bound | 11.0935 |  |
|  |  |  | Upper Bound | 18.4620 |  |
|  |  | 5% Trimmed Mean | | 14.6975 |  |
|  |  | Median | | 15.0000 |  |
|  |  | Variance | | 54.889 |  |
|  |  | Std. Deviation | | 7.40870 |  |
|  |  | Minimum | | 3.00 |  |
|  |  | Maximum | | 28.00 |  |
|  |  | Range | | 25.00 |  |
|  |  | Interquartile Range | | 11.50 |  |
|  |  | Skewness | | .253 | .536 |
|  |  | Kurtosis | | -.730 | 1.038 |
|  | CONTROL | Mean | | 14.2222 | 1.79425 |
|  |  | 95% Confidence Interval for Mean | Lower Bound | 10.4367 |  |
|  |  |  | Upper Bound | 18.0077 |  |
|  |  | 5% Trimmed Mean | | 14.0802 |  |
|  |  | Median | | 13.5000 |  |
|  |  | Variance | | 57.948 |  |
|  |  | Std. Deviation | | 7.61234 |  |
|  |  | Minimum | | 2.00 |  |
|  |  | Maximum | | 29.00 |  |
|  |  | Range | | 27.00 |  |
|  |  | Interquartile Range | | 10.00 |  |
|  |  | Skewness | | .397 | .536 |
|  |  | Kurtosis | | -.558 | 1.038 |

| **Tests of Normality** | | | | | | | |
| --- | --- | --- | --- | --- | --- | --- | --- |
|  | GROUP | Kolmogorov-Smirnov^a^ | | | Shapiro-Wilk | | |
|  |  | Statistic | df | Sig. | Statistic | df | Sig. |
| menstrual_time | LOW_INTENSITY | .142 | 18 | .200^*^ | .947 | 18 | .380 |
|  | MODERATE_INTENSITY | .165 | 18 | .200^*^ | .939 | 18 | .278 |
|  | HIGH_INTENSITY | .098 | 18 | .200^*^ | .964 | 18 | .678 |
|  | CONTROL | .108 | 18 | .200^*^ | .968 | 18 | .763 |
| *. This is a lower bound of the true significance. | | | | | | | |
| a. Lilliefors Significance Correction | | | | | | | |

**menstrual_time**

**Stem-and-Leaf Plots**

menstrual_time Stem-and-Leaf Plot for

GROUP= LOW_INTENSITY

Frequency Stem & Leaf

2.00 0 . 34

5.00 0 . 77889

3.00 1 . 113

3.00 1 . 579

4.00 2 . 0114

1.00 2 . 6

Stem width: 10.00

Each leaf: 1 case(s)

menstrual_time Stem-and-Leaf Plot for

GROUP= MODERATE_INTENSITY

Frequency Stem & Leaf

1.00 0 . 4

6.00 0 . 677799

1.00 1 . 3

6.00 1 . 555689

4.00 2 . 0134

Stem width: 10.00

Each leaf: 1 case(s)

menstrual_time Stem-and-Leaf Plot for

GROUP= HIGH_INTENSITY

Frequency Stem & Leaf

1.00 0 . 3

4.00 0 . 5778

3.00 1 . 023

6.00 1 . 556799

1.00 2 . 0

3.00 2 . 578

Stem width: 10.00

Each leaf: 1 case(s)

menstrual_time Stem-and-Leaf Plot for

GROUP= CONTROL

Frequency Stem & Leaf

2.00 0 . 24

4.00 0 . 7899

4.00 1 . 0134

4.00 1 . 5788

1.00 2 . 1

3.00 2 . 569

Stem width: 10.00

Each leaf: 1 case(s)

**Normal Q-Q Plots**


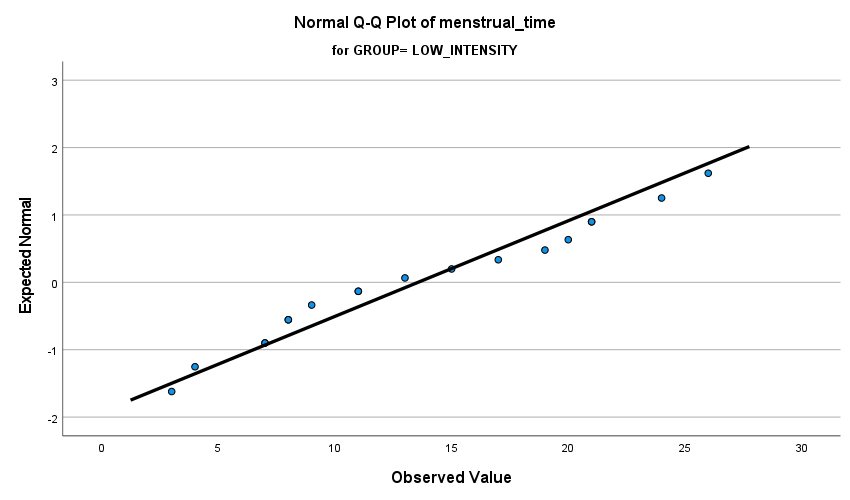


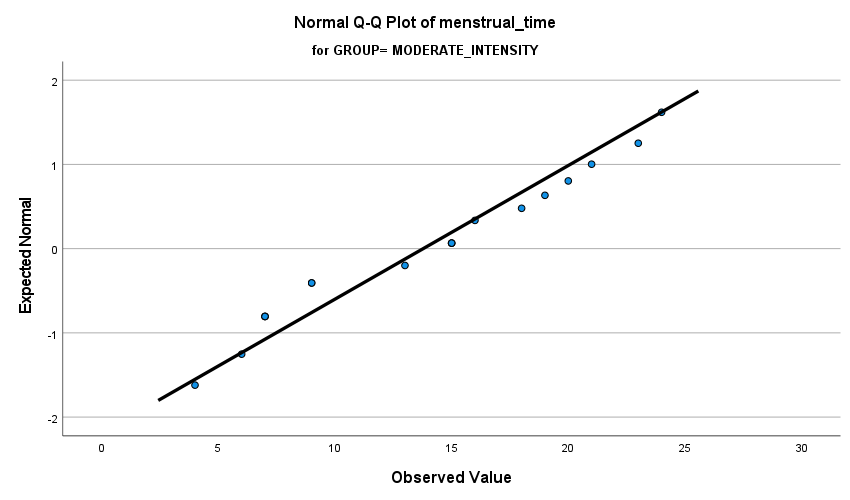


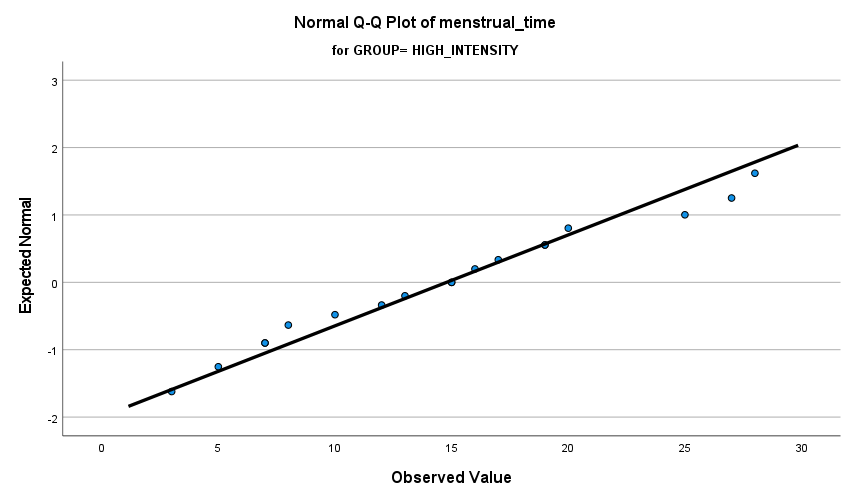


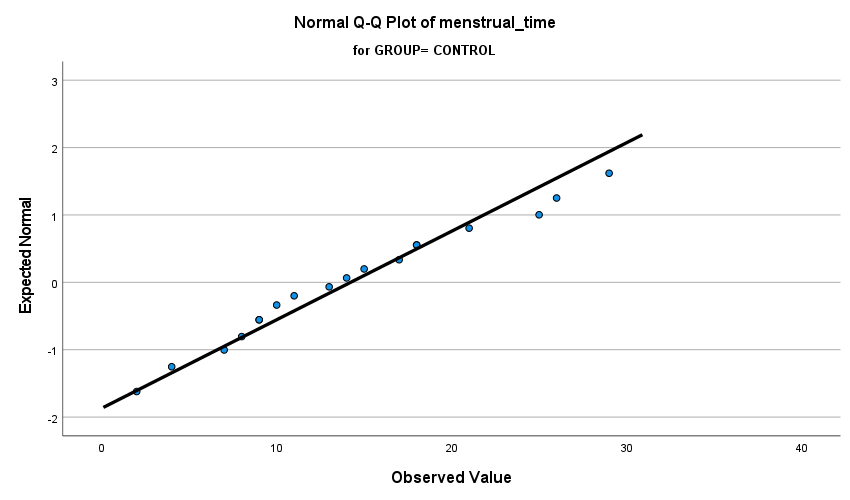


**Detrended Normal Q-Q Plots**


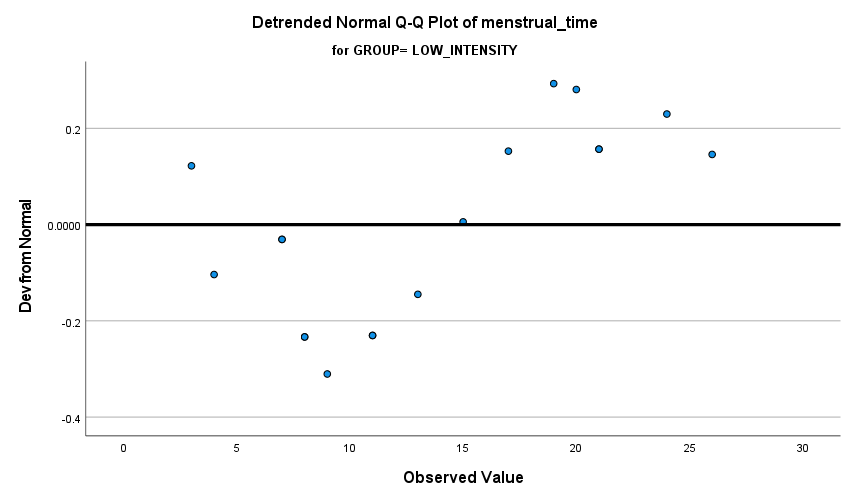


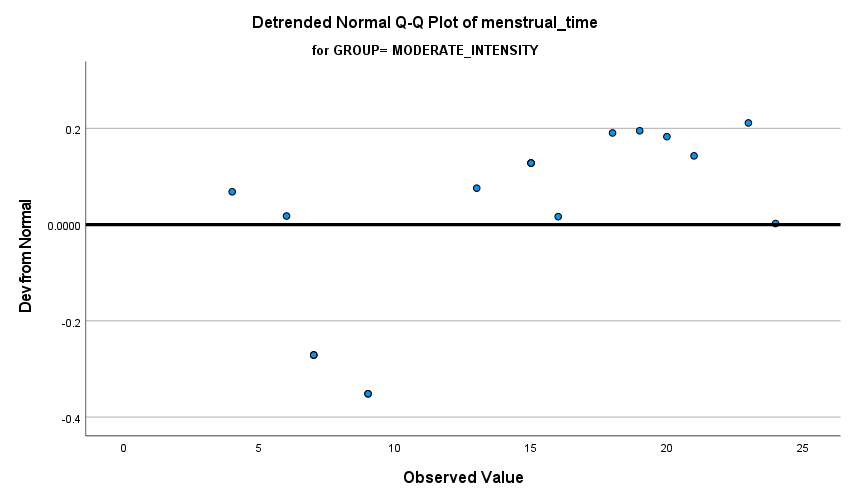


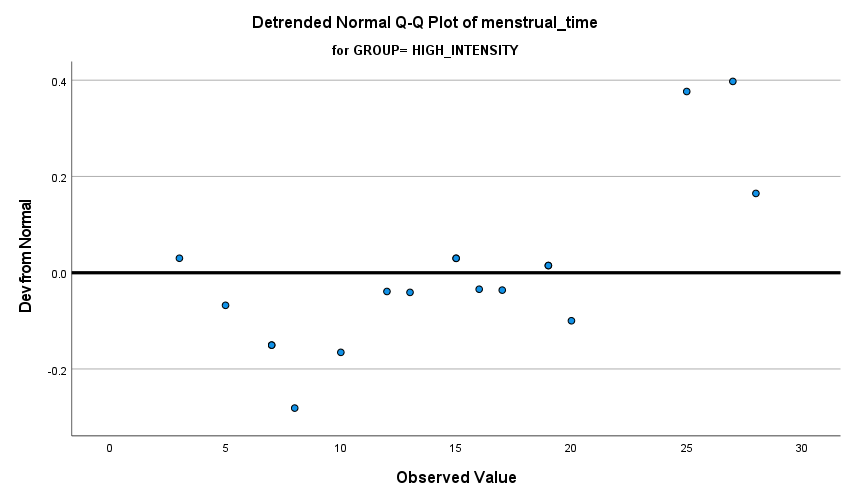


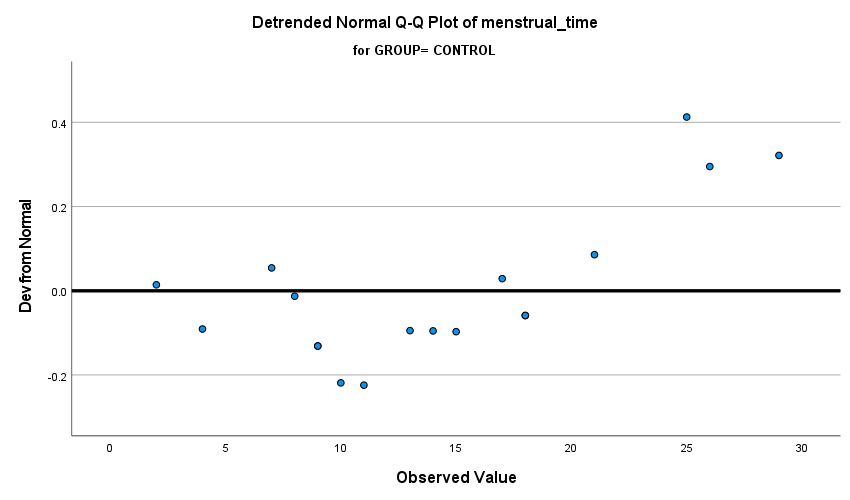


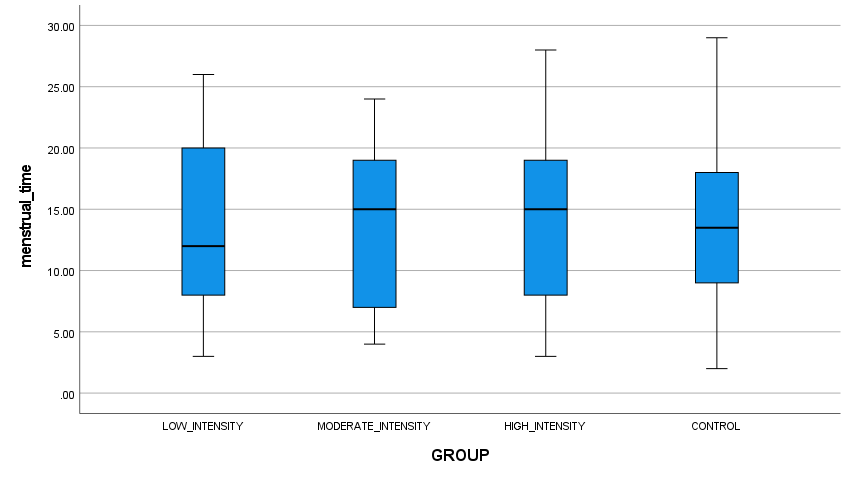


DATASET ACTIVATE DataSet2.

SAVE OUTFILE='C:\Users\user\Desktop\dana教写文章第一篇\4,5,6st, dana要求的两篇论文\Supplementary material\DATA '+

'nutritional intake-revised.sav'

/COMPRESSED.

EXAMINE VARIABLES=menstrual_cycle BY GROUP

/PLOT BOXPLOT STEMLEAF NPPLOT

/COMPARE GROUPS

/STATISTICS DESCRIPTIVES

/CINTERVAL 95

/MISSING LISTWISE

/NOTOTAL.

**Explore**

| **Notes** | | |
| --- | --- | --- |
| Output Created | | 14-APR-2025 21:00:32 |
| Comments | |  |
| Input | Data | C:\Users\user\Desktop\dana教写文章第一篇\4,5,6st, dana要求的两篇论文\Supplementary material\DATA nutritional intake-revised.sav |
|  | Active Dataset | DataSet2 |
|  | Filter | <none> |
|  | Weight | <none> |
|  | Split File | <none> |
|  | N of Rows in Working Data File | 144 |
| Missing Value Handling | Definition of Missing | User-defined missing values for dependent variables are treated as missing. |
|  | Cases Used | Statistics are based on cases with no missing values for any dependent variable or factor used. |
| Syntax | | EXAMINE VARIABLES=menstrual_cycle BY GROUP  /PLOT BOXPLOT STEMLEAF NPPLOT  /COMPARE GROUPS  /STATISTICS DESCRIPTIVES  /CINTERVAL 95  /MISSING LISTWISE  /NOTOTAL. |
| Resources | Processor Time | 00:00:01.50 |
|  | Elapsed Time | 00:00:01.64 |

**GROUP**

| **Case Processing Summary** | | | | | | | |
| --- | --- | --- | --- | --- | --- | --- | --- |
|  | GROUP | Cases | | | | | |
|  |  | Valid | | Missing | | Total | |
|  |  | N | Percent | N | Percent | N | Percent |
| menstrual_cycle | LOW_INTENSITY | 18 | 50.0% | 18 | 50.0% | 36 | 100.0% |
|  | MODERATE_INTENSITY | 18 | 50.0% | 18 | 50.0% | 36 | 100.0% |
|  | HIGH_INTENSITY | 18 | 50.0% | 18 | 50.0% | 36 | 100.0% |
|  | CONTROL | 18 | 50.0% | 18 | 50.0% | 36 | 100.0% |

| **Descriptives** | | | | | |
| --- | --- | --- | --- | --- | --- |
|  | GROUP | | | Statistic | Std. Error |
| menstrual_cycle | LOW_INTENSITY | Mean | | 31.2222 | .50846 |
|  |  | 95% Confidence Interval for Mean | Lower Bound | 30.1495 |  |
|  |  |  | Upper Bound | 32.2950 |  |
|  |  | 5% Trimmed Mean | | 31.1914 |  |
|  |  | Median | | 31.0000 |  |
|  |  | Variance | | 4.654 |  |
|  |  | Std. Deviation | | 2.15722 |  |
|  |  | Minimum | | 28.00 |  |
|  |  | Maximum | | 35.00 |  |
|  |  | Range | | 7.00 |  |
|  |  | Interquartile Range | | 4.00 |  |
|  |  | Skewness | | .073 | .536 |
|  |  | Kurtosis | | -1.109 | 1.038 |
|  | MODERATE_INTENSITY | Mean | | 31.3333 | .47829 |
|  |  | 95% Confidence Interval for Mean | Lower Bound | 30.3242 |  |
|  |  |  | Upper Bound | 32.3424 |  |
|  |  | 5% Trimmed Mean | | 31.3704 |  |
|  |  | Median | | 31.0000 |  |
|  |  | Variance | | 4.118 |  |
|  |  | Std. Deviation | | 2.02920 |  |
|  |  | Minimum | | 27.00 |  |
|  |  | Maximum | | 35.00 |  |
|  |  | Range | | 8.00 |  |
|  |  | Interquartile Range | | 3.00 |  |
|  |  | Skewness | | -.132 | .536 |
|  |  | Kurtosis | | -.056 | 1.038 |
|  | HIGH_INTENSITY | Mean | | 31.5556 | .47294 |
|  |  | 95% Confidence Interval for Mean | Lower Bound | 30.5577 |  |
|  |  |  | Upper Bound | 32.5534 |  |
|  |  | 5% Trimmed Mean | | 31.6173 |  |
|  |  | Median | | 32.0000 |  |
|  |  | Variance | | 4.026 |  |
|  |  | Std. Deviation | | 2.00653 |  |
|  |  | Minimum | | 27.00 |  |
|  |  | Maximum | | 35.00 |  |
|  |  | Range | | 8.00 |  |
|  |  | Interquartile Range | | 3.00 |  |
|  |  | Skewness | | -.485 | .536 |
|  |  | Kurtosis | | .261 | 1.038 |
|  | CONTROL | Mean | | 31.8889 | .45653 |
|  |  | 95% Confidence Interval for Mean | Lower Bound | 30.9257 |  |
|  |  |  | Upper Bound | 32.8521 |  |
|  |  | 5% Trimmed Mean | | 31.9321 |  |
|  |  | Median | | 32.0000 |  |
|  |  | Variance | | 3.752 |  |
|  |  | Std. Deviation | | 1.93691 |  |
|  |  | Minimum | | 28.00 |  |
|  |  | Maximum | | 35.00 |  |
|  |  | Range | | 7.00 |  |
|  |  | Interquartile Range | | 2.50 |  |
|  |  | Skewness | | -.152 | .536 |
|  |  | Kurtosis | | -.302 | 1.038 |

| **Tests of Normality** | | | | | | | |
| --- | --- | --- | --- | --- | --- | --- | --- |
|  | GROUP | Kolmogorov-Smirnov^a^ | | | Shapiro-Wilk | | |
|  |  | Statistic | df | Sig. | Statistic | df | Sig. |
| menstrual_cycle | LOW_INTENSITY | .128 | 18 | .200^*^ | .950 | 18 | .427 |
|  | MODERATE_INTENSITY | .121 | 18 | .200^*^ | .976 | 18 | .906 |
|  | HIGH_INTENSITY | .199 | 18 | .058 | .958 | 18 | .565 |
|  | CONTROL | .144 | 18 | .200^*^ | .965 | 18 | .696 |
| *. This is a lower bound of the true significance. | | | | | | | |
| a. Lilliefors Significance Correction | | | | | | | |

**menstrual_cycle**

**Stem-and-Leaf Plots**

menstrual_cycle Stem-and-Leaf Plot for

GROUP= LOW_INTENSITY

Frequency Stem & Leaf

2.00 28 . 00

3.00 29 . 000

2.00 30 . 00

3.00 31 . 000

2.00 32 . 00

3.00 33 . 000

2.00 34 . 00

1.00 35 . 0

Stem width: 1.00

Each leaf: 1 case(s)

menstrual_cycle Stem-and-Leaf Plot for

GROUP= MODERATE_INTENSITY

Frequency Stem & Leaf

1.00 27 . 0

.00 28 .

2.00 29 . 00

3.00 30 . 000

4.00 31 . 0000

3.00 32 . 000

2.00 33 . 00

2.00 34 . 00

1.00 35 . 0

Stem width: 1.00

Each leaf: 1 case(s)

menstrual_cycle Stem-and-Leaf Plot for

GROUP= HIGH_INTENSITY

Frequency Stem & Leaf

1.00 27 . 0

.00 28 .

2.00 29 . 00

2.00 30 . 00

2.00 31 . 00

6.00 32 . 000000

2.00 33 . 00

2.00 34 . 00

1.00 35 . 0

Stem width: 1.00

Each leaf: 1 case(s)

menstrual_cycle Stem-and-Leaf Plot for

GROUP= CONTROL

Frequency Stem & Leaf

1.00 28 . 0

1.00 29 . 0

2.00 30 . 00

3.00 31 . 000

5.00 32 . 00000

2.00 33 . 00

2.00 34 . 00

2.00 35 . 00

Stem width: 1.00

Each leaf: 1 case(s)

**Normal Q-Q Plots**


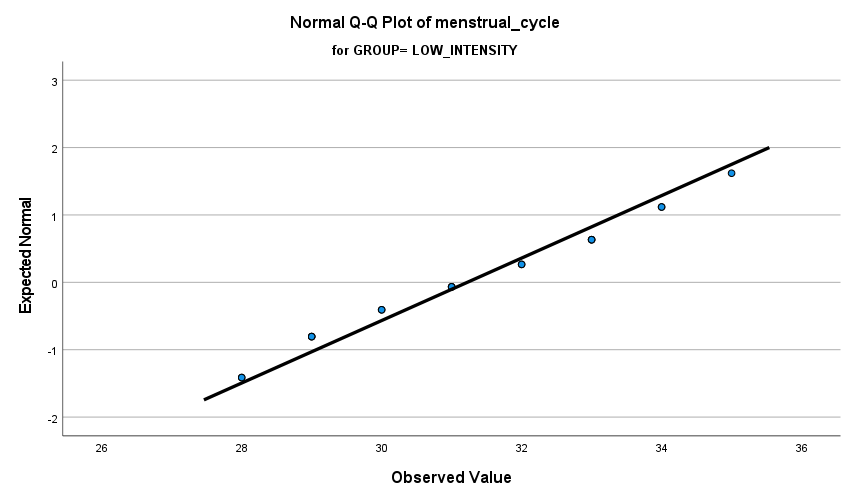


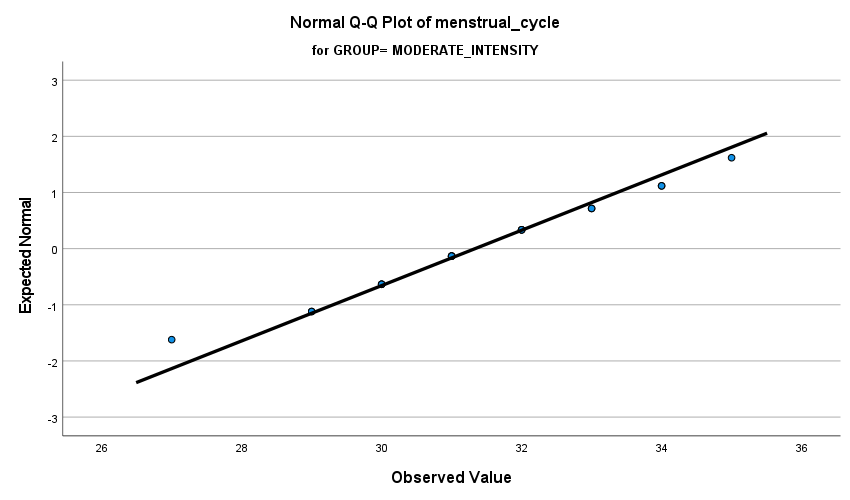


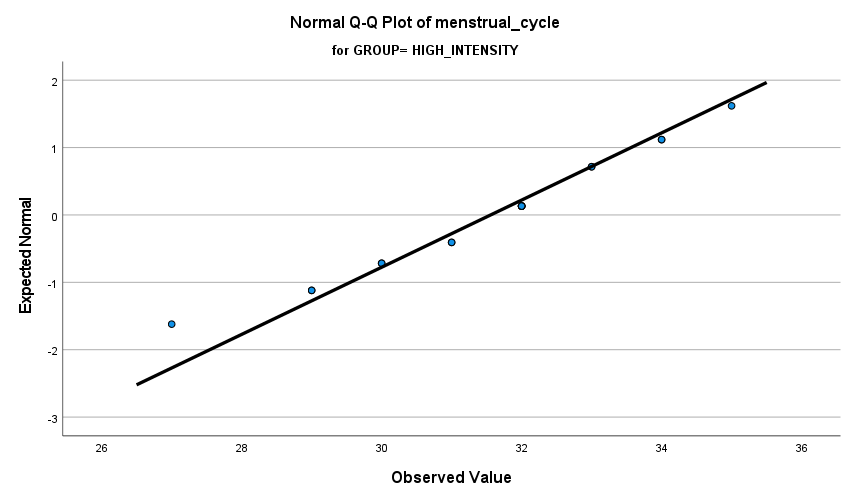


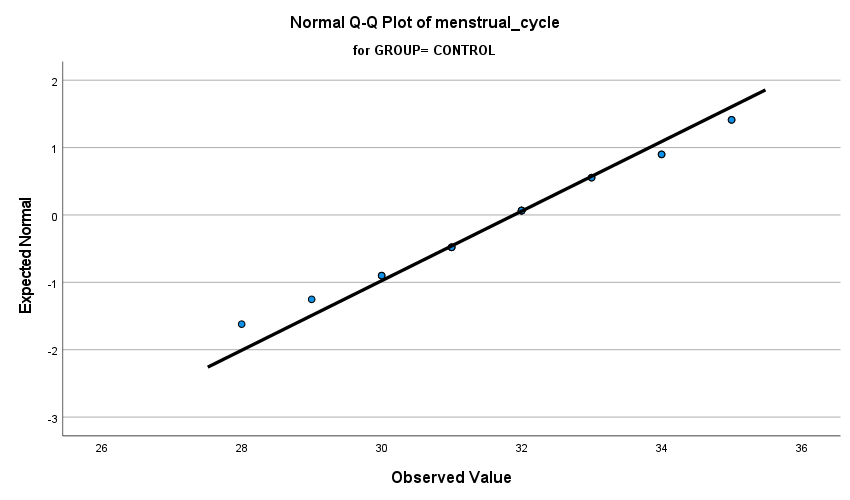


**Detrended Normal Q-Q Plots**


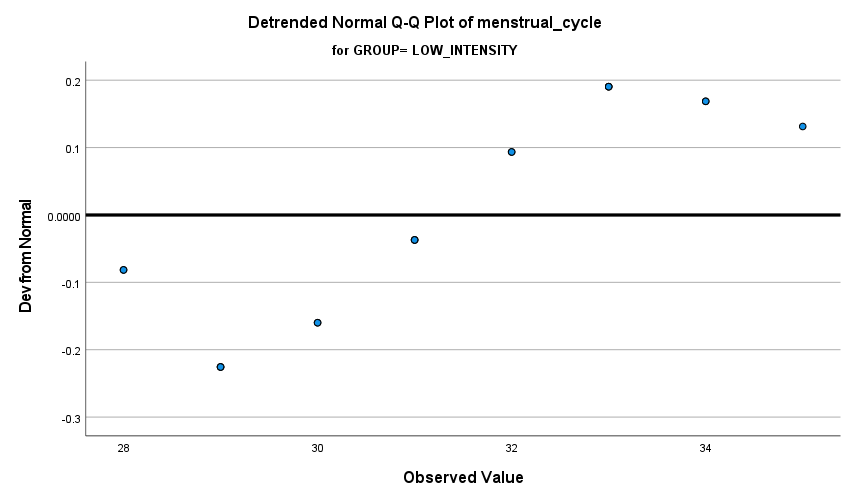


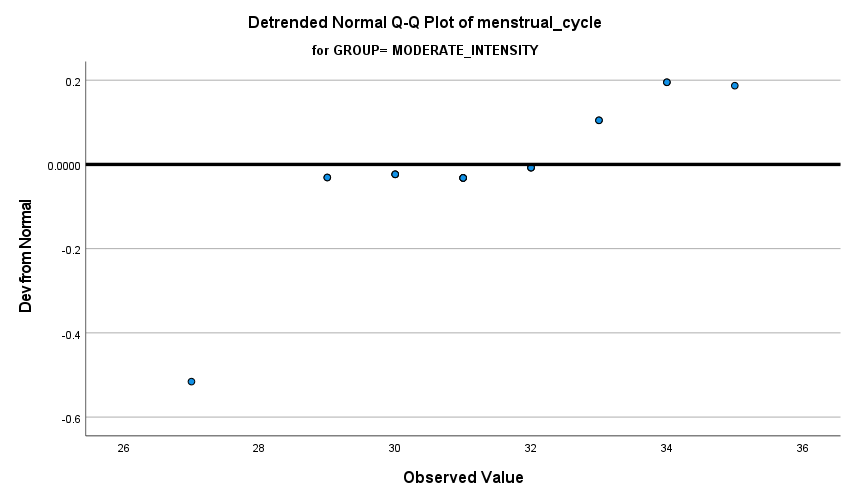


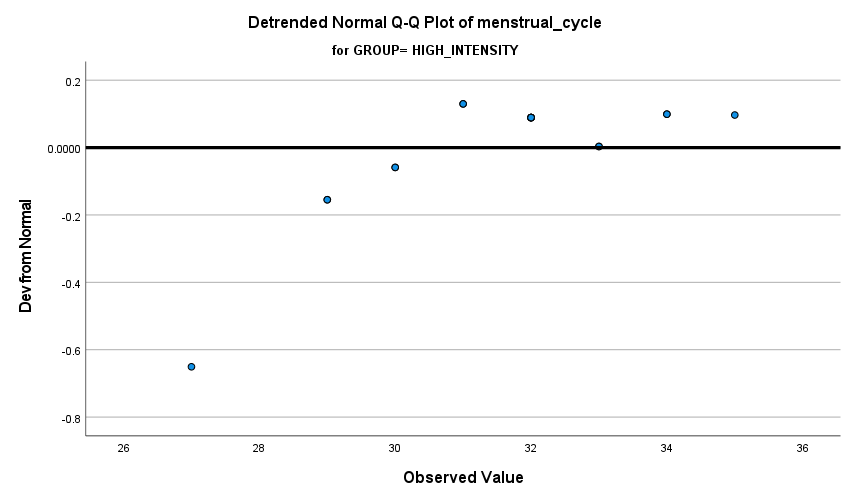


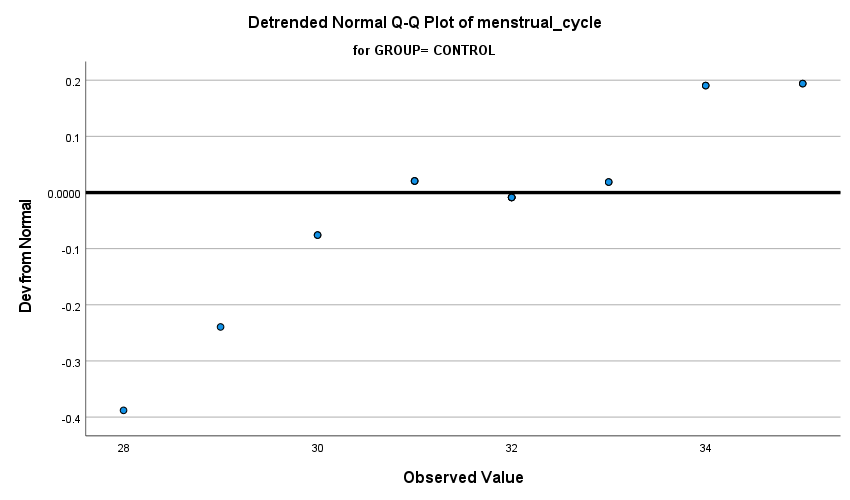


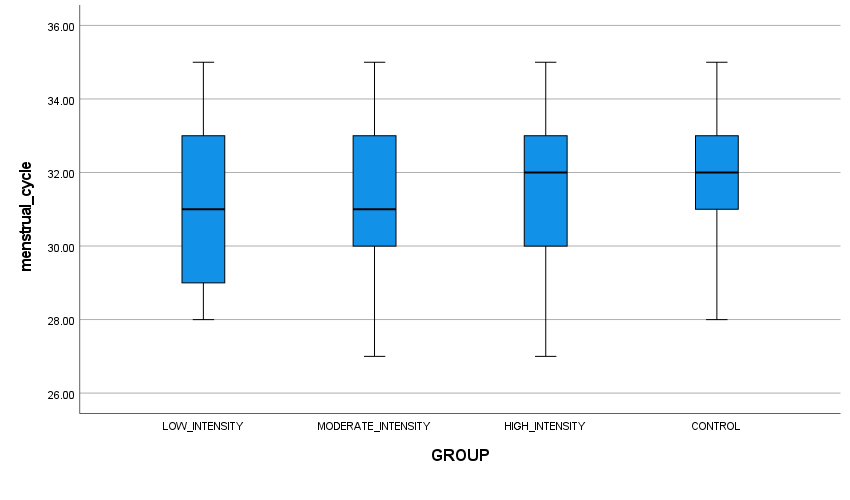


EXAMINE VARIABLES=height weight age depression anxiety stress mealtime BY GROUP

/PLOT BOXPLOT STEMLEAF NPPLOT

/COMPARE GROUPS

/STATISTICS DESCRIPTIVES

/CINTERVAL 95

/MISSING LISTWISE

/NOTOTAL.

**Explore**

| **Notes** | | |
| --- | --- | --- |
| Output Created | | 23-MAY-2023 21:04:04 |
| Comments | |  |
| Input | Data | C:\Users\mdanaee\Google Drive\Data Analysis\Prof SOh students\Prof Soh -Wang\DATA1.sav |
|  | Active Dataset | DataSet1 |
|  | Filter | <none> |
|  | Weight | <none> |
|  | Split File | <none> |
|  | N of Rows in Working Data File | 72 |
| Missing Value Handling | Definition of Missing | User-defined missing values for dependent variables are treated as missing. |
|  | Cases Used | Statistics are based on cases with no missing values for any dependent variable or factor used. |
| Syntax | | EXAMINE VARIABLES=height weight age depression anxiety stress mealtime BY GROUP  /PLOT BOXPLOT STEMLEAF NPPLOT  /COMPARE GROUPS  /STATISTICS DESCRIPTIVES  /CINTERVAL 95  /MISSING LISTWISE  /NOTOTAL. |
| Resources | Processor Time | 00:00:12.55 |
|  | Elapsed Time | 00:00:12.56 |

**GROUP**

| **Case Processing Summary** | | | | | | | |
| --- | --- | --- | --- | --- | --- | --- | --- |
|  | GROUP | Cases | | | | | |
|  |  | Valid | | Missing | | Total | |
|  |  | N | Percent | N | Percent | N | Percent |
| height | LOW_INTENSITY | 18 | 100.0% | 0 | 0.0% | 18 | 100.0% |
|  | MODERATE_INTENSITY | 18 | 100.0% | 0 | 0.0% | 18 | 100.0% |
|  | HIGH_INTENSITY | 18 | 100.0% | 0 | 0.0% | 18 | 100.0% |
|  | Control | 18 | 100.0% | 0 | 0.0% | 18 | 100.0% |
| weight | LOW_INTENSITY | 18 | 100.0% | 0 | 0.0% | 18 | 100.0% |
|  | MODERATE_INTENSITY | 18 | 100.0% | 0 | 0.0% | 18 | 100.0% |
|  | HIGH_INTENSITY | 18 | 100.0% | 0 | 0.0% | 18 | 100.0% |
|  | Control | 18 | 100.0% | 0 | 0.0% | 18 | 100.0% |
| age | LOW_INTENSITY | 18 | 100.0% | 0 | 0.0% | 18 | 100.0% |
|  | MODERATE_INTENSITY | 18 | 100.0% | 0 | 0.0% | 18 | 100.0% |
|  | HIGH_INTENSITY | 18 | 100.0% | 0 | 0.0% | 18 | 100.0% |
|  | Control | 18 | 100.0% | 0 | 0.0% | 18 | 100.0% |
| depression | LOW_INTENSITY | 18 | 100.0% | 0 | 0.0% | 18 | 100.0% |
|  | MODERATE_INTENSITY | 18 | 100.0% | 0 | 0.0% | 18 | 100.0% |
|  | HIGH_INTENSITY | 18 | 100.0% | 0 | 0.0% | 18 | 100.0% |
|  | Control | 18 | 100.0% | 0 | 0.0% | 18 | 100.0% |
| anxiety | LOW_INTENSITY | 18 | 100.0% | 0 | 0.0% | 18 | 100.0% |
|  | MODERATE_INTENSITY | 18 | 100.0% | 0 | 0.0% | 18 | 100.0% |
|  | HIGH_INTENSITY | 18 | 100.0% | 0 | 0.0% | 18 | 100.0% |
|  | Control | 18 | 100.0% | 0 | 0.0% | 18 | 100.0% |
| stress | LOW_INTENSITY | 18 | 100.0% | 0 | 0.0% | 18 | 100.0% |
|  | MODERATE_INTENSITY | 18 | 100.0% | 0 | 0.0% | 18 | 100.0% |
|  | HIGH_INTENSITY | 18 | 100.0% | 0 | 0.0% | 18 | 100.0% |
|  | Control | 18 | 100.0% | 0 | 0.0% | 18 | 100.0% |
| mealtime | LOW_INTENSITY | 18 | 100.0% | 0 | 0.0% | 18 | 100.0% |
|  | MODERATE_INTENSITY | 18 | 100.0% | 0 | 0.0% | 18 | 100.0% |
|  | HIGH_INTENSITY | 18 | 100.0% | 0 | 0.0% | 18 | 100.0% |
|  | Control | 18 | 100.0% | 0 | 0.0% | 18 | 100.0% |

| **Descriptives** | | | | | |
| --- | --- | --- | --- | --- | --- |
|  | GROUP | | | Statistic | Std. Error |
| height | LOW_INTENSITY | Mean | | 1.6000 | .00907 |
|  |  | 95% Confidence Interval for Mean | Lower Bound | 1.5809 |  |
|  |  |  | Upper Bound | 1.6191 |  |
|  |  | 5% Trimmed Mean | | 1.6000 |  |
|  |  | Median | | 1.6000 |  |
|  |  | Variance | | .001 |  |
|  |  | Std. Deviation | | .03850 |  |
|  |  | Minimum | | 1.53 |  |
|  |  | Maximum | | 1.67 |  |
|  |  | Range | | .14 |  |
|  |  | Interquartile Range | | .06 |  |
|  |  | Skewness | | .000 | .536 |
|  |  | Kurtosis | | -.642 | 1.038 |
|  | MODERATE_INTENSITY | Mean | | 1.5900 | .00859 |
|  |  | 95% Confidence Interval for Mean | Lower Bound | 1.5719 |  |
|  |  |  | Upper Bound | 1.6081 |  |
|  |  | 5% Trimmed Mean | | 1.5900 |  |
|  |  | Median | | 1.5900 |  |
|  |  | Variance | | .001 |  |
|  |  | Std. Deviation | | .03646 |  |
|  |  | Minimum | | 1.53 |  |
|  |  | Maximum | | 1.65 |  |
|  |  | Range | | .12 |  |
|  |  | Interquartile Range | | .06 |  |
|  |  | Skewness | | .000 | .536 |
|  |  | Kurtosis | | -.967 | 1.038 |
|  | HIGH_INTENSITY | Mean | | 1.6000 | .00907 |
|  |  | 95% Confidence Interval for Mean | Lower Bound | 1.5809 |  |
|  |  |  | Upper Bound | 1.6191 |  |
|  |  | 5% Trimmed Mean | | 1.6000 |  |
|  |  | Median | | 1.6000 |  |
|  |  | Variance | | .001 |  |
|  |  | Std. Deviation | | .03850 |  |
|  |  | Minimum | | 1.53 |  |
|  |  | Maximum | | 1.67 |  |
|  |  | Range | | .14 |  |
|  |  | Interquartile Range | | .06 |  |
|  |  | Skewness | | .000 | .536 |
|  |  | Kurtosis | | -.642 | 1.038 |
|  | Control | Mean | | 1.6000 | .00907 |
|  |  | 95% Confidence Interval for Mean | Lower Bound | 1.5809 |  |
|  |  |  | Upper Bound | 1.6191 |  |
|  |  | 5% Trimmed Mean | | 1.6000 |  |
|  |  | Median | | 1.6000 |  |
|  |  | Variance | | .001 |  |
|  |  | Std. Deviation | | .03850 |  |
|  |  | Minimum | | 1.53 |  |
|  |  | Maximum | | 1.67 |  |
|  |  | Range | | .14 |  |
|  |  | Interquartile Range | | .06 |  |
|  |  | Skewness | | .000 | .536 |
|  |  | Kurtosis | | -.642 | 1.038 |
| weight | LOW_INTENSITY | Mean | | 73.5700 | 1.56090 |
|  |  | 95% Confidence Interval for Mean | Lower Bound | 70.2768 |  |
|  |  |  | Upper Bound | 76.8632 |  |
|  |  | 5% Trimmed Mean | | 73.5700 |  |
|  |  | Median | | 73.5700 |  |
|  |  | Variance | | 43.855 |  |
|  |  | Std. Deviation | | 6.62233 |  |
|  |  | Minimum | | 60.59 |  |
|  |  | Maximum | | 86.55 |  |
|  |  | Range | | 25.96 |  |
|  |  | Interquartile Range | | 7.60 |  |
|  |  | Skewness | | .000 | .536 |
|  |  | Kurtosis | | -.072 | 1.038 |
|  | MODERATE_INTENSITY | Mean | | 72.6600 | 1.54005 |
|  |  | 95% Confidence Interval for Mean | Lower Bound | 69.4108 |  |
|  |  |  | Upper Bound | 75.9092 |  |
|  |  | 5% Trimmed Mean | | 72.6600 |  |
|  |  | Median | | 72.6600 |  |
|  |  | Variance | | 42.692 |  |
|  |  | Std. Deviation | | 6.53390 |  |
|  |  | Minimum | | 61.74 |  |
|  |  | Maximum | | 83.58 |  |
|  |  | Range | | 21.84 |  |
|  |  | Interquartile Range | | 9.13 |  |
|  |  | Skewness | | .009 | .536 |
|  |  | Kurtosis | | -.871 | 1.038 |
|  | HIGH_INTENSITY | Mean | | 73.5600 | 1.55918 |
|  |  | 95% Confidence Interval for Mean | Lower Bound | 70.2704 |  |
|  |  |  | Upper Bound | 76.8496 |  |
|  |  | 5% Trimmed Mean | | 73.5600 |  |
|  |  | Median | | 73.3050 |  |
|  |  | Variance | | 43.759 |  |
|  |  | Std. Deviation | | 6.61503 |  |
|  |  | Minimum | | 62.49 |  |
|  |  | Maximum | | 84.63 |  |
|  |  | Range | | 22.14 |  |
|  |  | Interquartile Range | | 10.58 |  |
|  |  | Skewness | | .013 | .536 |
|  |  | Kurtosis | | -.750 | 1.038 |
|  | Control | Mean | | 73.3400 | 1.55634 |
|  |  | 95% Confidence Interval for Mean | Lower Bound | 70.0564 |  |
|  |  |  | Upper Bound | 76.6236 |  |
|  |  | 5% Trimmed Mean | | 73.3400 |  |
|  |  | Median | | 73.3400 |  |
|  |  | Variance | | 43.600 |  |
|  |  | Std. Deviation | | 6.60300 |  |
|  |  | Minimum | | 60.66 |  |
|  |  | Maximum | | 86.02 |  |
|  |  | Range | | 25.36 |  |
|  |  | Interquartile Range | | 5.01 |  |
|  |  | Skewness | | .001 | .536 |
|  |  | Kurtosis | | .260 | 1.038 |
| age | LOW_INTENSITY | Mean | | 18.7744 | .09978 |
|  |  | 95% Confidence Interval for Mean | Lower Bound | 18.5639 |  |
|  |  |  | Upper Bound | 18.9850 |  |
|  |  | 5% Trimmed Mean | | 18.7649 |  |
|  |  | Median | | 18.8450 |  |
|  |  | Variance | | .179 |  |
|  |  | Std. Deviation | | .42332 |  |
|  |  | Minimum | | 18.11 |  |
|  |  | Maximum | | 19.61 |  |
|  |  | Range | | 1.50 |  |
|  |  | Interquartile Range | | .64 |  |
|  |  | Skewness | | .127 | .536 |
|  |  | Kurtosis | | -.711 | 1.038 |
|  | MODERATE_INTENSITY | Mean | | 18.9700 | .10117 |
|  |  | 95% Confidence Interval for Mean | Lower Bound | 18.7565 |  |
|  |  |  | Upper Bound | 19.1835 |  |
|  |  | 5% Trimmed Mean | | 18.9761 |  |
|  |  | Median | | 19.0350 |  |
|  |  | Variance | | .184 |  |
|  |  | Std. Deviation | | .42924 |  |
|  |  | Minimum | | 18.11 |  |
|  |  | Maximum | | 19.72 |  |
|  |  | Range | | 1.61 |  |
|  |  | Interquartile Range | | .77 |  |
|  |  | Skewness | | -.297 | .536 |
|  |  | Kurtosis | | -.572 | 1.038 |
|  | HIGH_INTENSITY | Mean | | 19.0411 | .10181 |
|  |  | 95% Confidence Interval for Mean | Lower Bound | 18.8263 |  |
|  |  |  | Upper Bound | 19.2559 |  |
|  |  | 5% Trimmed Mean | | 19.0485 |  |
|  |  | Median | | 19.1850 |  |
|  |  | Variance | | .187 |  |
|  |  | Std. Deviation | | .43194 |  |
|  |  | Minimum | | 18.34 |  |
|  |  | Maximum | | 19.61 |  |
|  |  | Range | | 1.27 |  |
|  |  | Interquartile Range | | .80 |  |
|  |  | Skewness | | -.355 | .536 |
|  |  | Kurtosis | | -1.356 | 1.038 |
|  | Control | Mean | | 18.9306 | .10123 |
|  |  | 95% Confidence Interval for Mean | Lower Bound | 18.7170 |  |
|  |  |  | Upper Bound | 19.1441 |  |
|  |  | 5% Trimmed Mean | | 18.9312 |  |
|  |  | Median | | 18.8100 |  |
|  |  | Variance | | .184 |  |
|  |  | Std. Deviation | | .42947 |  |
|  |  | Minimum | | 18.21 |  |
|  |  | Maximum | | 19.64 |  |
|  |  | Range | | 1.43 |  |
|  |  | Interquartile Range | | .70 |  |
|  |  | Skewness | | .125 | .536 |
|  |  | Kurtosis | | -1.058 | 1.038 |
| depression | LOW_INTENSITY | Mean | | 7.33 | .886 |
|  |  | 95% Confidence Interval for Mean | Lower Bound | 5.46 |  |
|  |  |  | Upper Bound | 9.20 |  |
|  |  | 5% Trimmed Mean | | 7.26 |  |
|  |  | Median | | 7.50 |  |
|  |  | Variance | | 14.118 |  |
|  |  | Std. Deviation | | 3.757 |  |
|  |  | Minimum | | 2 |  |
|  |  | Maximum | | 14 |  |
|  |  | Range | | 12 |  |
|  |  | Interquartile Range | | 6 |  |
|  |  | Skewness | | .111 | .536 |
|  |  | Kurtosis | | -.852 | 1.038 |
|  | MODERATE_INTENSITY | Mean | | 8.11 | .973 |
|  |  | 95% Confidence Interval for Mean | Lower Bound | 6.06 |  |
|  |  |  | Upper Bound | 10.16 |  |
|  |  | 5% Trimmed Mean | | 8.07 |  |
|  |  | Median | | 8.50 |  |
|  |  | Variance | | 17.046 |  |
|  |  | Std. Deviation | | 4.129 |  |
|  |  | Minimum | | 2 |  |
|  |  | Maximum | | 15 |  |
|  |  | Range | | 13 |  |
|  |  | Interquartile Range | | 8 |  |
|  |  | Skewness | | .069 | .536 |
|  |  | Kurtosis | | -1.211 | 1.038 |
|  | HIGH_INTENSITY | Mean | | 7.72 | .925 |
|  |  | 95% Confidence Interval for Mean | Lower Bound | 5.77 |  |
|  |  |  | Upper Bound | 9.67 |  |
|  |  | 5% Trimmed Mean | | 7.64 |  |
|  |  | Median | | 8.00 |  |
|  |  | Variance | | 15.389 |  |
|  |  | Std. Deviation | | 3.923 |  |
|  |  | Minimum | | 2 |  |
|  |  | Maximum | | 15 |  |
|  |  | Range | | 13 |  |
|  |  | Interquartile Range | | 7 |  |
|  |  | Skewness | | .280 | .536 |
|  |  | Kurtosis | | -.748 | 1.038 |
|  | Control | Mean | | 7.56 | .905 |
|  |  | 95% Confidence Interval for Mean | Lower Bound | 5.65 |  |
|  |  |  | Upper Bound | 9.46 |  |
|  |  | 5% Trimmed Mean | | 7.51 |  |
|  |  | Median | | 7.50 |  |
|  |  | Variance | | 14.732 |  |
|  |  | Std. Deviation | | 3.838 |  |
|  |  | Minimum | | 2 |  |
|  |  | Maximum | | 14 |  |
|  |  | Range | | 12 |  |
|  |  | Interquartile Range | | 7 |  |
|  |  | Skewness | | .278 | .536 |
|  |  | Kurtosis | | -.922 | 1.038 |
| anxiety | LOW_INTENSITY | Mean | | 13.33 | 1.419 |
|  |  | 95% Confidence Interval for Mean | Lower Bound | 10.34 |  |
|  |  |  | Upper Bound | 16.33 |  |
|  |  | 5% Trimmed Mean | | 13.31 |  |
|  |  | Median | | 14.00 |  |
|  |  | Variance | | 36.235 |  |
|  |  | Std. Deviation | | 6.020 |  |
|  |  | Minimum | | 4 |  |
|  |  | Maximum | | 23 |  |
|  |  | Range | | 19 |  |
|  |  | Interquartile Range | | 11 |  |
|  |  | Skewness | | .020 | .536 |
|  |  | Kurtosis | | -1.205 | 1.038 |
|  | MODERATE_INTENSITY | Mean | | 12.61 | 1.341 |
|  |  | 95% Confidence Interval for Mean | Lower Bound | 9.78 |  |
|  |  |  | Upper Bound | 15.44 |  |
|  |  | 5% Trimmed Mean | | 12.57 |  |
|  |  | Median | | 12.50 |  |
|  |  | Variance | | 32.369 |  |
|  |  | Std. Deviation | | 5.689 |  |
|  |  | Minimum | | 4 |  |
|  |  | Maximum | | 22 |  |
|  |  | Range | | 18 |  |
|  |  | Interquartile Range | | 11 |  |
|  |  | Skewness | | .151 | .536 |
|  |  | Kurtosis | | -1.144 | 1.038 |
|  | HIGH_INTENSITY | Mean | | 12.50 | 1.334 |
|  |  | 95% Confidence Interval for Mean | Lower Bound | 9.69 |  |
|  |  |  | Upper Bound | 15.31 |  |
|  |  | 5% Trimmed Mean | | 12.50 |  |
|  |  | Median | | 12.50 |  |
|  |  | Variance | | 32.029 |  |
|  |  | Std. Deviation | | 5.659 |  |
|  |  | Minimum | | 4 |  |
|  |  | Maximum | | 21 |  |
|  |  | Range | | 17 |  |
|  |  | Interquartile Range | | 11 |  |
|  |  | Skewness | | .048 | .536 |
|  |  | Kurtosis | | -1.206 | 1.038 |
|  | Control | Mean | | 12.89 | 1.367 |
|  |  | 95% Confidence Interval for Mean | Lower Bound | 10.00 |  |
|  |  |  | Upper Bound | 15.77 |  |
|  |  | 5% Trimmed Mean | | 12.82 |  |
|  |  | Median | | 12.00 |  |
|  |  | Variance | | 33.634 |  |
|  |  | Std. Deviation | | 5.799 |  |
|  |  | Minimum | | 5 |  |
|  |  | Maximum | | 22 |  |
|  |  | Range | | 17 |  |
|  |  | Interquartile Range | | 11 |  |
|  |  | Skewness | | .245 | .536 |
|  |  | Kurtosis | | -1.212 | 1.038 |
| stress | LOW_INTENSITY | Mean | | 16.39 | 1.615 |
|  |  | 95% Confidence Interval for Mean | Lower Bound | 12.98 |  |
|  |  |  | Upper Bound | 19.80 |  |
|  |  | 5% Trimmed Mean | | 16.54 |  |
|  |  | Median | | 17.50 |  |
|  |  | Variance | | 46.958 |  |
|  |  | Std. Deviation | | 6.853 |  |
|  |  | Minimum | | 4 |  |
|  |  | Maximum | | 26 |  |
|  |  | Range | | 22 |  |
|  |  | Interquartile Range | | 12 |  |
|  |  | Skewness | | -.338 | .536 |
|  |  | Kurtosis | | -1.114 | 1.038 |
|  | MODERATE_INTENSITY | Mean | | 15.61 | 1.538 |
|  |  | 95% Confidence Interval for Mean | Lower Bound | 12.37 |  |
|  |  |  | Upper Bound | 18.86 |  |
|  |  | 5% Trimmed Mean | | 15.68 |  |
|  |  | Median | | 16.50 |  |
|  |  | Variance | | 42.605 |  |
|  |  | Std. Deviation | | 6.527 |  |
|  |  | Minimum | | 5 |  |
|  |  | Maximum | | 25 |  |
|  |  | Range | | 20 |  |
|  |  | Interquartile Range | | 12 |  |
|  |  | Skewness | | -.243 | .536 |
|  |  | Kurtosis | | -1.280 | 1.038 |
|  | HIGH_INTENSITY | Mean | | 16.06 | 1.577 |
|  |  | 95% Confidence Interval for Mean | Lower Bound | 12.73 |  |
|  |  |  | Upper Bound | 19.38 |  |
|  |  | 5% Trimmed Mean | | 16.17 |  |
|  |  | Median | | 16.50 |  |
|  |  | Variance | | 44.761 |  |
|  |  | Std. Deviation | | 6.690 |  |
|  |  | Minimum | | 4 |  |
|  |  | Maximum | | 26 |  |
|  |  | Range | | 22 |  |
|  |  | Interquartile Range | | 12 |  |
|  |  | Skewness | | -.229 | .536 |
|  |  | Kurtosis | | -1.051 | 1.038 |
|  | Control | Mean | | 15.94 | 1.569 |
|  |  | 95% Confidence Interval for Mean | Lower Bound | 12.63 |  |
|  |  |  | Upper Bound | 19.25 |  |
|  |  | 5% Trimmed Mean | | 15.99 |  |
|  |  | Median | | 15.50 |  |
|  |  | Variance | | 44.291 |  |
|  |  | Std. Deviation | | 6.655 |  |
|  |  | Minimum | | 4 |  |
|  |  | Maximum | | 27 |  |
|  |  | Range | | 23 |  |
|  |  | Interquartile Range | | 10 |  |
|  |  | Skewness | | -.084 | .536 |
|  |  | Kurtosis | | -.633 | 1.038 |
| mealtime | LOW_INTENSITY | Mean | | 42.1694 | 1.59585 |
|  |  | 95% Confidence Interval for Mean | Lower Bound | 38.8025 |  |
|  |  |  | Upper Bound | 45.5364 |  |
|  |  | 5% Trimmed Mean | | 42.2938 |  |
|  |  | Median | | 42.0250 |  |
|  |  | Variance | | 45.841 |  |
|  |  | Std. Deviation | | 6.77060 |  |
|  |  | Minimum | | 29.48 |  |
|  |  | Maximum | | 52.62 |  |
|  |  | Range | | 23.14 |  |
|  |  | Interquartile Range | | 10.80 |  |
|  |  | Skewness | | -.162 | .536 |
|  |  | Kurtosis | | -.830 | 1.038 |
|  | MODERATE_INTENSITY | Mean | | 43.7622 | 1.65676 |
|  |  | 95% Confidence Interval for Mean | Lower Bound | 40.2668 |  |
|  |  |  | Upper Bound | 47.2577 |  |
|  |  | 5% Trimmed Mean | | 43.5630 |  |
|  |  | Median | | 41.9400 |  |
|  |  | Variance | | 49.407 |  |
|  |  | Std. Deviation | | 7.02904 |  |
|  |  | Minimum | | 33.36 |  |
|  |  | Maximum | | 57.75 |  |
|  |  | Range | | 24.39 |  |
|  |  | Interquartile Range | | 11.13 |  |
|  |  | Skewness | | .587 | .536 |
|  |  | Kurtosis | | -.518 | 1.038 |
|  | HIGH_INTENSITY | Mean | | 41.9728 | 1.58807 |
|  |  | 95% Confidence Interval for Mean | Lower Bound | 38.6223 |  |
|  |  |  | Upper Bound | 45.3233 |  |
|  |  | 5% Trimmed Mean | | 41.7486 |  |
|  |  | Median | | 41.0500 |  |
|  |  | Variance | | 45.395 |  |
|  |  | Std. Deviation | | 6.73760 |  |
|  |  | Minimum | | 32.75 |  |
|  |  | Maximum | | 55.23 |  |
|  |  | Range | | 22.48 |  |
|  |  | Interquartile Range | | 11.20 |  |
|  |  | Skewness | | .496 | .536 |
|  |  | Kurtosis | | -.690 | 1.038 |
|  | Control | Mean | | 42.4922 | 1.61005 |
|  |  | 95% Confidence Interval for Mean | Lower Bound | 39.0953 |  |
|  |  |  | Upper Bound | 45.8891 |  |
|  |  | 5% Trimmed Mean | | 42.4314 |  |
|  |  | Median | | 41.8900 |  |
|  |  | Variance | | 46.661 |  |
|  |  | Std. Deviation | | 6.83086 |  |
|  |  | Minimum | | 32.11 |  |
|  |  | Maximum | | 53.97 |  |
|  |  | Range | | 21.86 |  |
|  |  | Interquartile Range | | 11.32 |  |
|  |  | Skewness | | .162 | .536 |
|  |  | Kurtosis | | -1.080 | 1.038 |

| **Tests of Normality** | | | | | | | |
| --- | --- | --- | --- | --- | --- | --- | --- |
|  | GROUP | Kolmogorov-Smirnov^a^ | | | Shapiro-Wilk | | |
|  |  | Statistic | df | Sig. | Statistic | df | Sig. |
| height | LOW_INTENSITY | .167 | 18 | .200^*^ | .957 | 18 | .547 |
|  | MODERATE_INTENSITY | .167 | 18 | .200^*^ | .937 | 18 | .259 |
|  | HIGH_INTENSITY | .167 | 18 | .200^*^ | .957 | 18 | .547 |
|  | Control | .167 | 18 | .200^*^ | .957 | 18 | .547 |
| weight | LOW_INTENSITY | .098 | 18 | .200^*^ | .991 | 18 | 1.000 |
|  | MODERATE_INTENSITY | .121 | 18 | .200^*^ | .967 | 18 | .732 |
|  | HIGH_INTENSITY | .106 | 18 | .200^*^ | .965 | 18 | .696 |
|  | Control | .161 | 18 | .200^*^ | .944 | 18 | .335 |
| age | LOW_INTENSITY | .132 | 18 | .200^*^ | .962 | 18 | .634 |
|  | MODERATE_INTENSITY | .167 | 18 | .200^*^ | .957 | 18 | .549 |
|  | HIGH_INTENSITY | .169 | 18 | .187 | .914 | 18 | .101 |
|  | Control | .166 | 18 | .200^*^ | .951 | 18 | .440 |
| depression | LOW_INTENSITY | .098 | 18 | .200^*^ | .953 | 18 | .466 |
|  | MODERATE_INTENSITY | .118 | 18 | .200^*^ | .944 | 18 | .341 |
|  | HIGH_INTENSITY | .108 | 18 | .200^*^ | .948 | 18 | .399 |
|  | Control | .105 | 18 | .200^*^ | .942 | 18 | .317 |
| anxiety | LOW_INTENSITY | .109 | 18 | .200^*^ | .956 | 18 | .527 |
|  | MODERATE_INTENSITY | .116 | 18 | .200^*^ | .955 | 18 | .516 |
|  | HIGH_INTENSITY | .112 | 18 | .200^*^ | .943 | 18 | .325 |
|  | Control | .128 | 18 | .200^*^ | .935 | 18 | .237 |
| stress | LOW_INTENSITY | .138 | 18 | .200^*^ | .948 | 18 | .394 |
|  | MODERATE_INTENSITY | .138 | 18 | .200^*^ | .940 | 18 | .286 |
|  | HIGH_INTENSITY | .111 | 18 | .200^*^ | .957 | 18 | .549 |
|  | Control | .108 | 18 | .200^*^ | .973 | 18 | .844 |
| mealtime | LOW_INTENSITY | .086 | 18 | .200^*^ | .973 | 18 | .853 |
|  | MODERATE_INTENSITY | .135 | 18 | .200^*^ | .952 | 18 | .465 |
|  | HIGH_INTENSITY | .087 | 18 | .200^*^ | .954 | 18 | .489 |
|  | Control | .079 | 18 | .200^*^ | .960 | 18 | .609 |
| *. This is a lower bound of the true significance. | | | | | | | |
| a. Lilliefors Significance Correction | | | | | | | |

**height**

**Stem-and-Leaf Plots**

height Stem-and-Leaf Plot for

GROUP= LOW_INTENSITY

Frequency Stem & Leaf

1.00 15 . 3

5.00 15 . 55777

9.00 16 . 000000333

3.00 16 . 557

Stem width: .10

Each leaf: 1 case(s)

height Stem-and-Leaf Plot for

GROUP= MODERATE_INTENSITY

Frequency Stem & Leaf

3.00 15 . 344

9.00 15 . 666999999

5.00 16 . 22244

1.00 16 . 5

Stem width: .10

Each leaf: 1 case(s)

height Stem-and-Leaf Plot for

GROUP= HIGH_INTENSITY

Frequency Stem & Leaf

1.00 15 . 3

5.00 15 . 55777

9.00 16 . 000000333

3.00 16 . 557

Stem width: .10

Each leaf: 1 case(s)

height Stem-and-Leaf Plot for

GROUP= Control

Frequency Stem & Leaf

1.00 15 . 3

5.00 15 . 55777

9.00 16 . 000000333

3.00 16 . 557

Stem width: .10

Each leaf: 1 case(s)

**Normal Q-Q Plots**


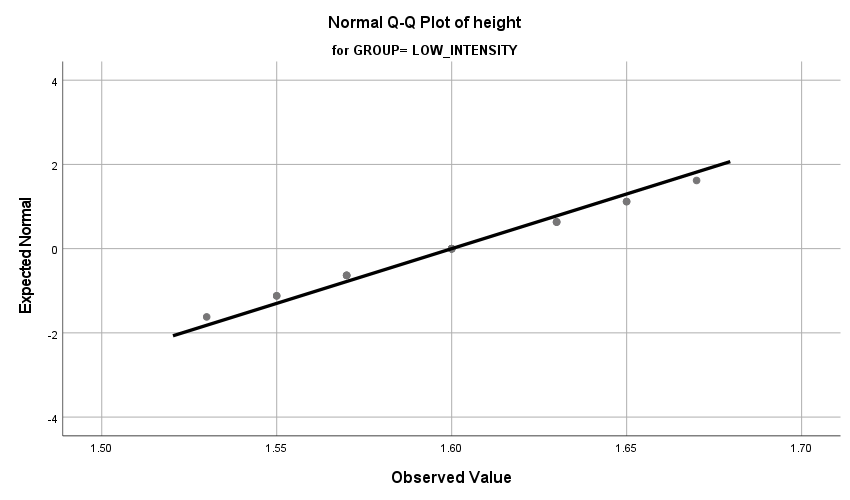


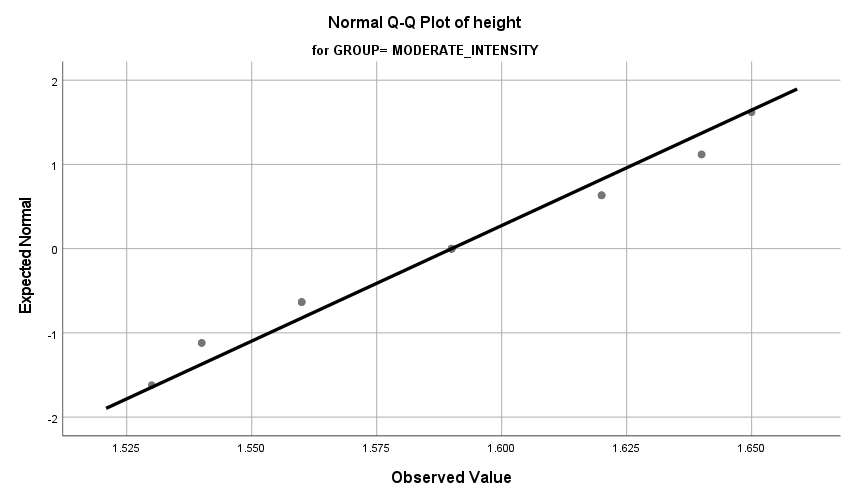


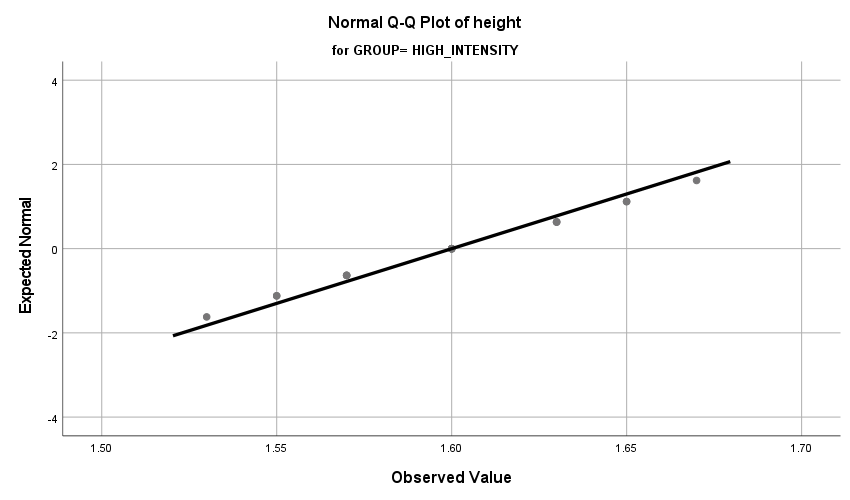


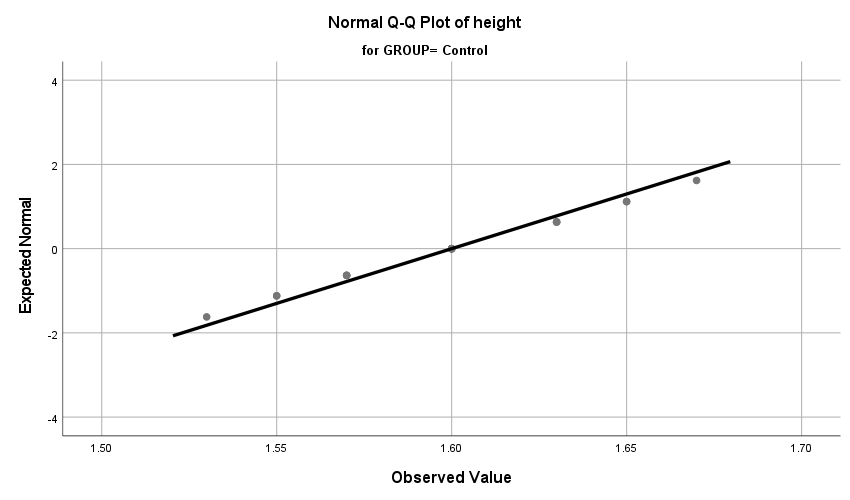


**Detrended Normal Q-Q Plots**


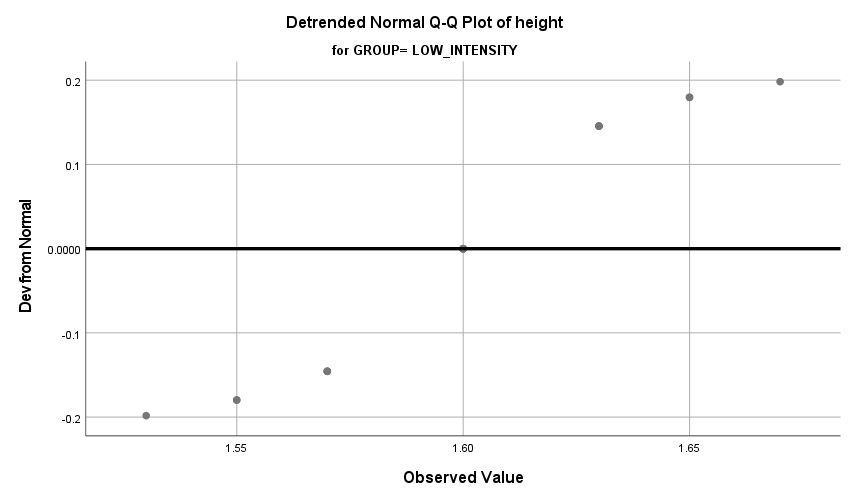


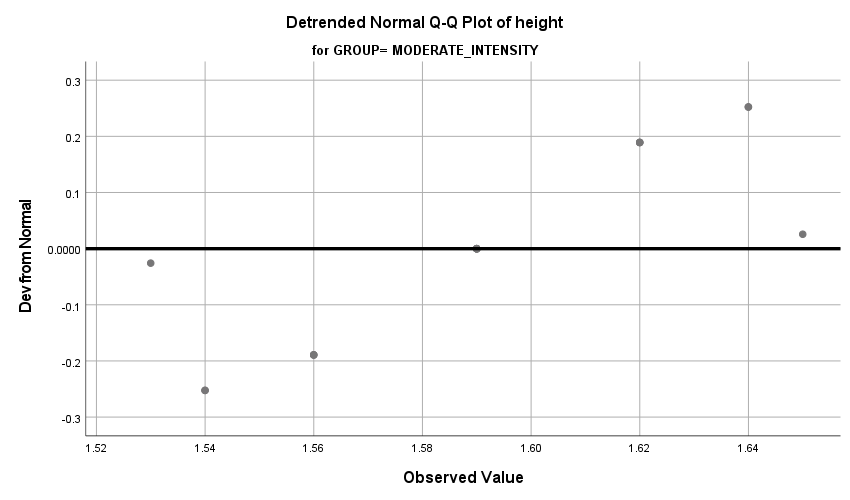


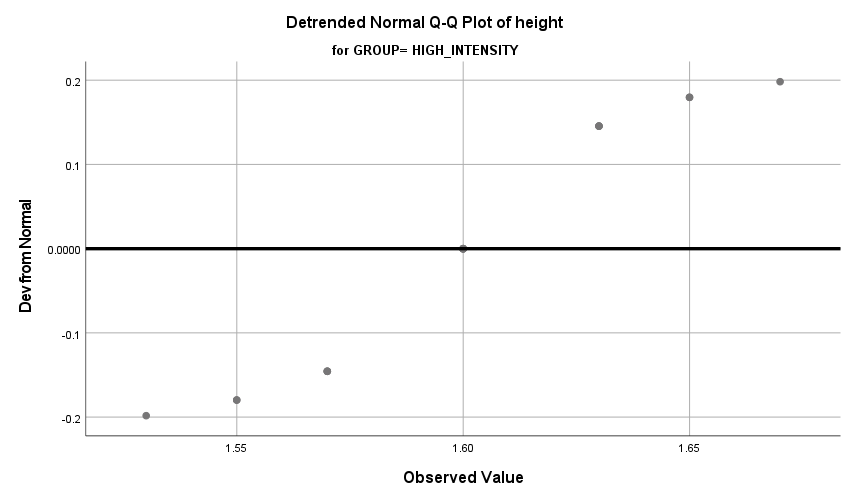


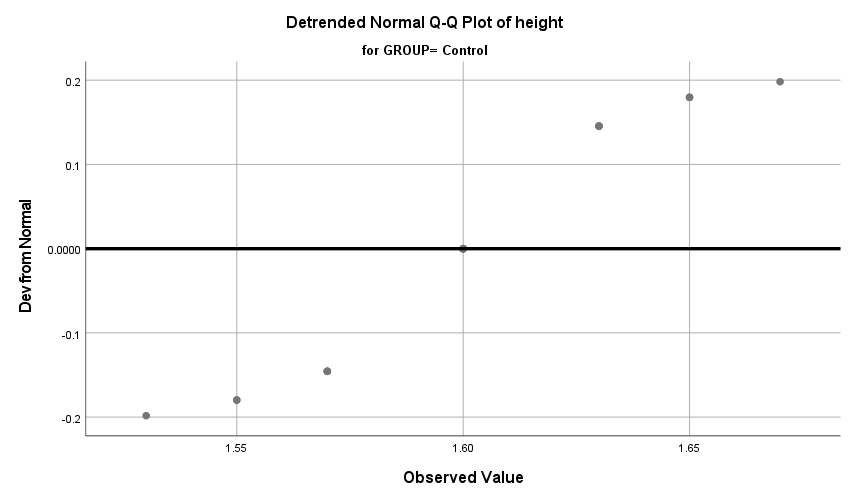


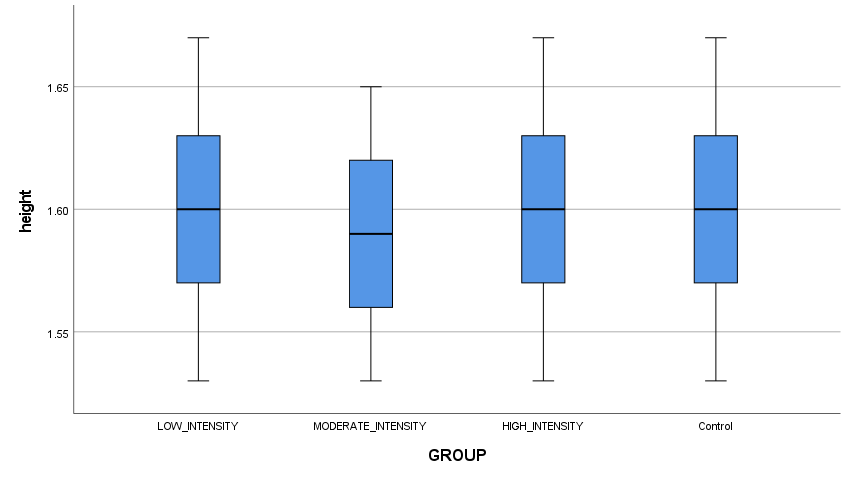


**weight**

**Stem-and-Leaf Plots**

weight Stem-and-Leaf Plot for

GROUP= LOW_INTENSITY

Frequency Stem & Leaf

1.00 Extremes (=<61)

1.00 6 . 4

2.00 6 . 57

7.00 7 . 0012334

4.00 7 . 5669

2.00 8 . 12

1.00 Extremes (>=87)

Stem width: 10.00

Each leaf: 1 case(s)

weight Stem-and-Leaf Plot for

GROUP= MODERATE_INTENSITY

Frequency Stem & Leaf

2.00 6 . 12

5.00 6 . 55889

4.00 7 . 0044

4.00 7 . 5668

3.00 8 . 023

Stem width: 10.00

Each leaf: 1 case(s)

weight Stem-and-Leaf Plot for

GROUP= HIGH_INTENSITY

Frequency Stem & Leaf

3.00 6 . 234

2.00 6 . 78

6.00 7 . 122334

3.00 7 . 567

4.00 8 . 0234

Stem width: 10.00

Each leaf: 1 case(s)

weight Stem-and-Leaf Plot for

GROUP= Control

Frequency Stem & Leaf

3.00 Extremes (=<64)

1.00 6 . 9

9.00 7 . 122233334

2.00 7 . 57

3.00 Extremes (>=82)

Stem width: 10.00

Each leaf: 1 case(s)

**Normal Q-Q Plots**


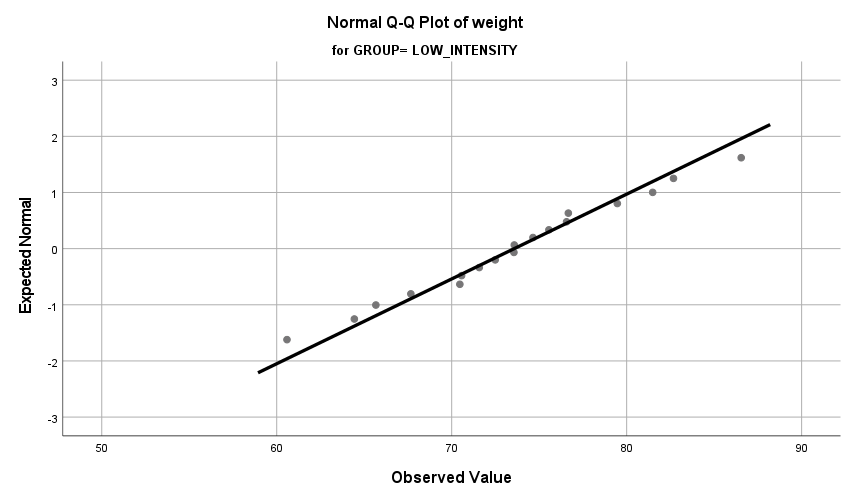


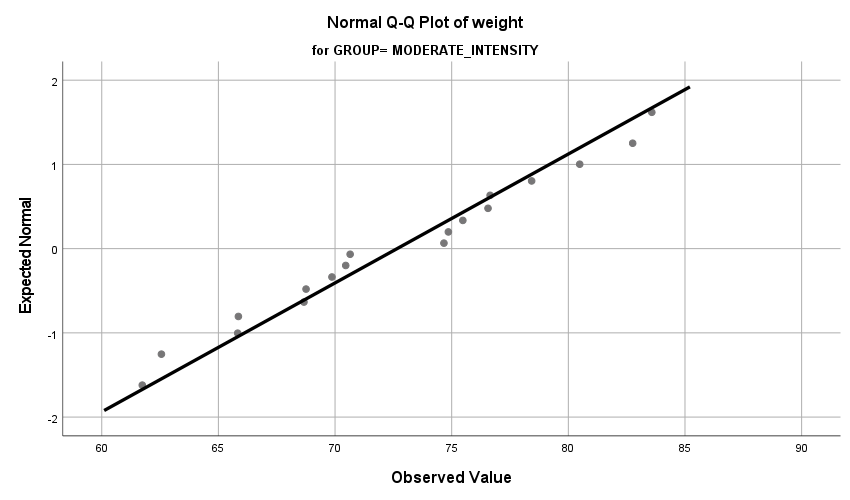


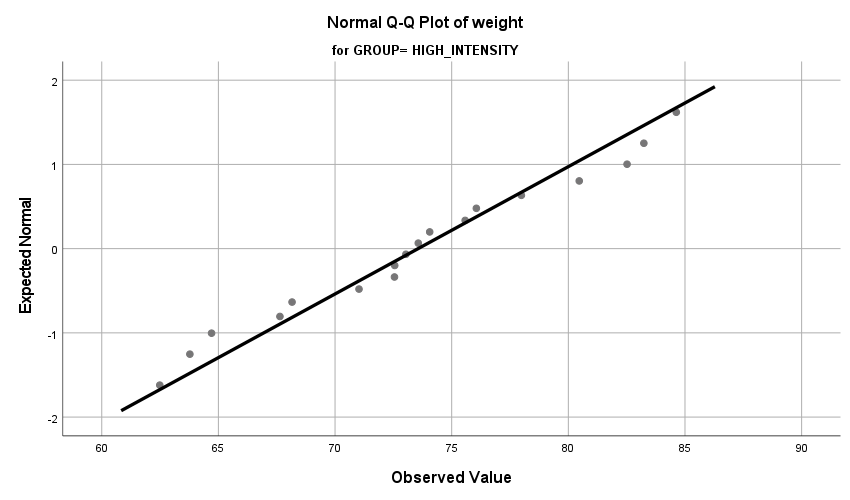


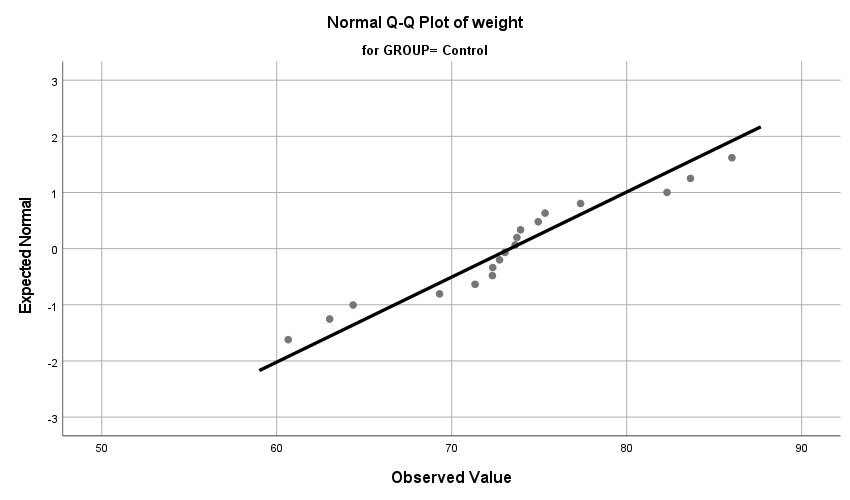


**Detrended Normal Q-Q Plots**


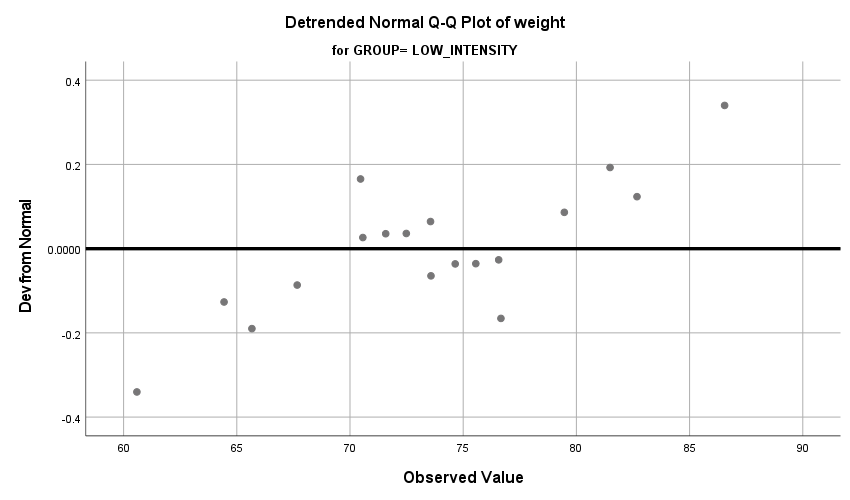


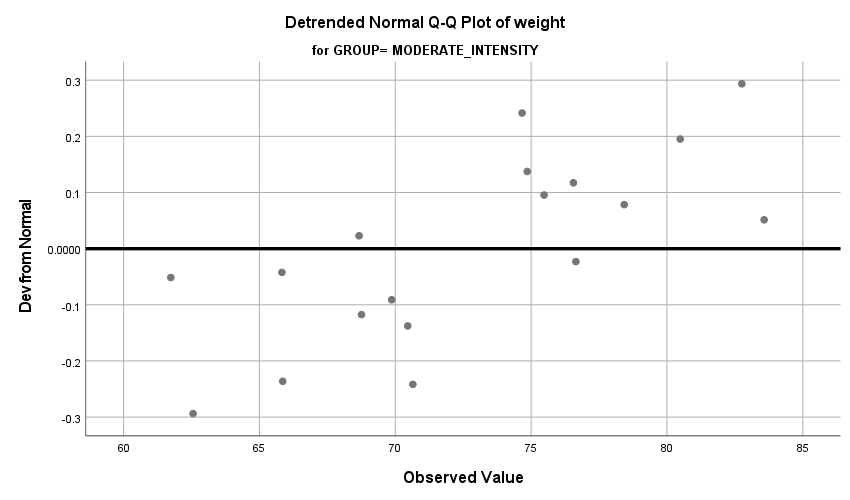


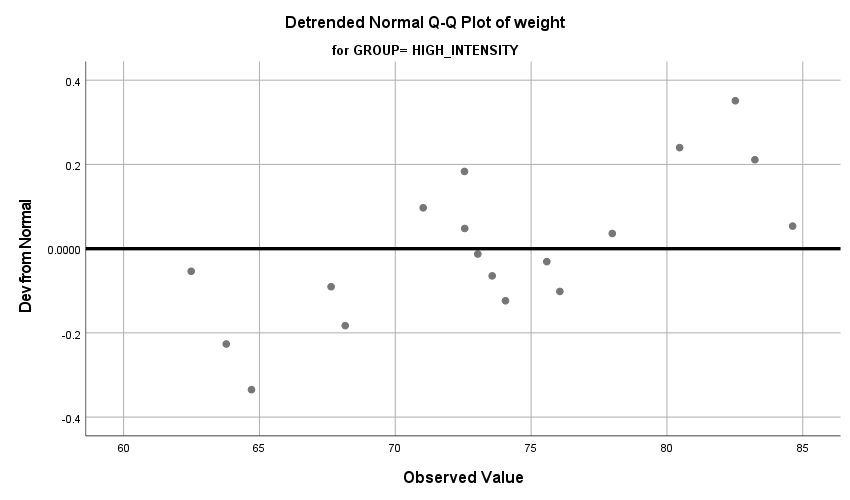


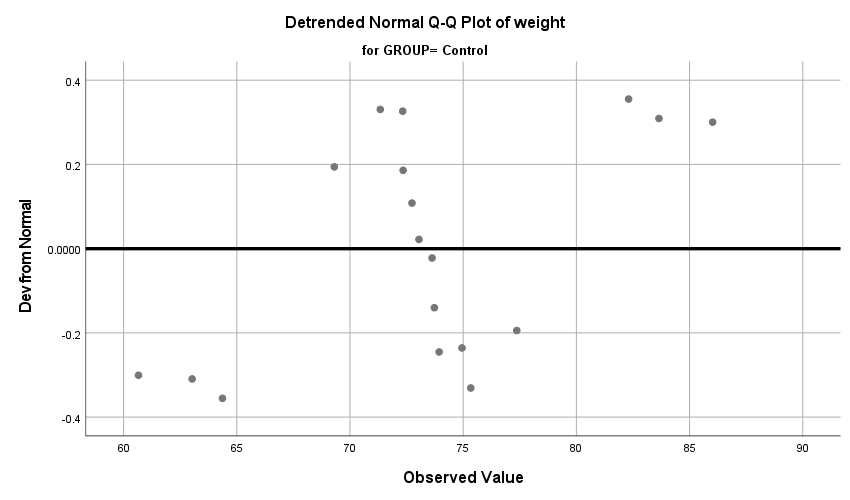


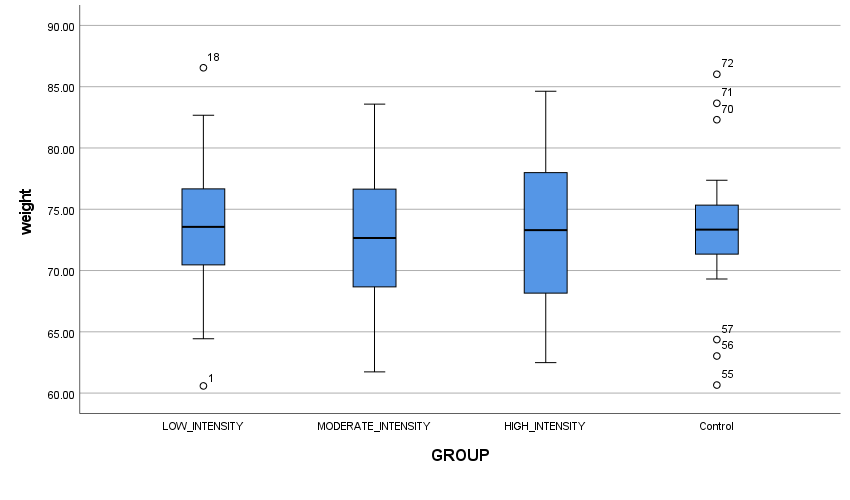


**age**

**Stem-and-Leaf Plots**

age Stem-and-Leaf Plot for

GROUP= LOW_INTENSITY

Frequency Stem & Leaf

6.00 18 . 122444

8.00 18 . 57888999

3.00 19 . 223

1.00 19 . 6

Stem width: 1.00

Each leaf: 1 case(s)

age Stem-and-Leaf Plot for

GROUP= MODERATE_INTENSITY

Frequency Stem & Leaf

3.00 18 . 144

4.00 18 . 5569

10.00 19 . 0000033334

1.00 19 . 7

Stem width: 1.00

Each leaf: 1 case(s)

age Stem-and-Leaf Plot for

GROUP= HIGH_INTENSITY

Frequency Stem & Leaf

3.00 18 . 344

4.00 18 . 5678

8.00 19 . 01222334

3.00 19 . 556

Stem width: 1.00

Each leaf: 1 case(s)

age Stem-and-Leaf Plot for

GROUP= Control

Frequency Stem & Leaf

2.00 18 . 23

9.00 18 . 556677889

5.00 19 . 22234

2.00 19 . 56

Stem width: 1.00

Each leaf: 1 case(s)

**Normal Q-Q Plots**


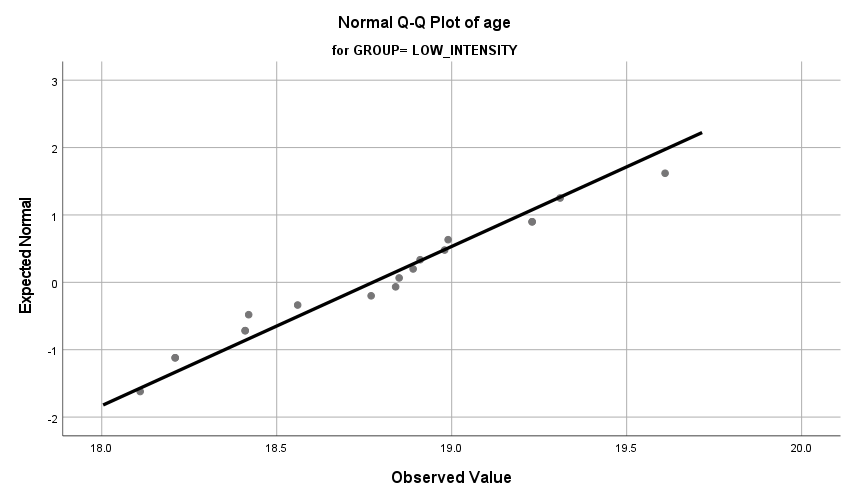


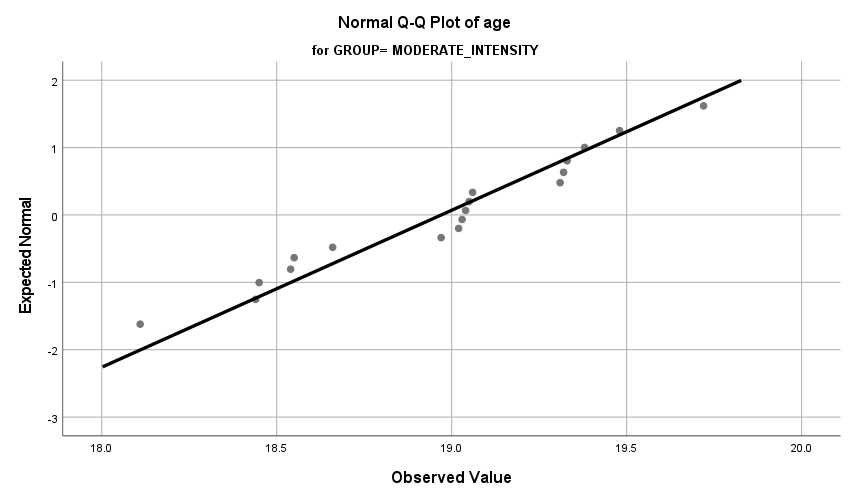


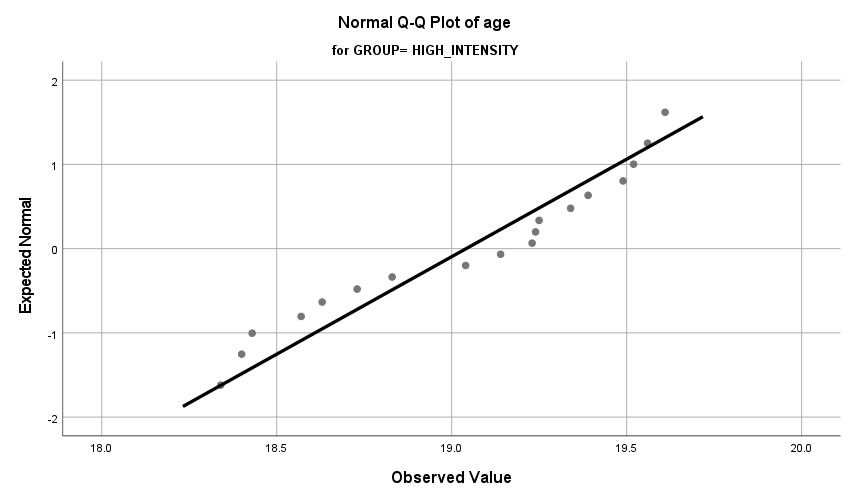


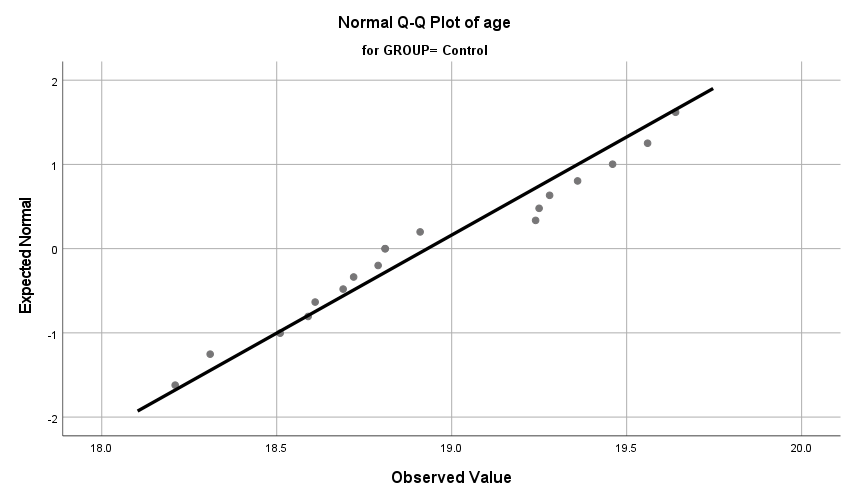


**Detrended Normal Q-Q Plots**


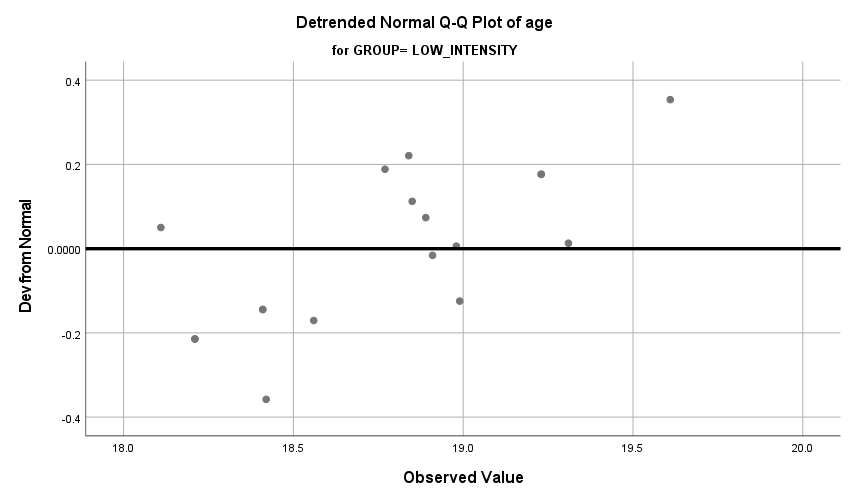


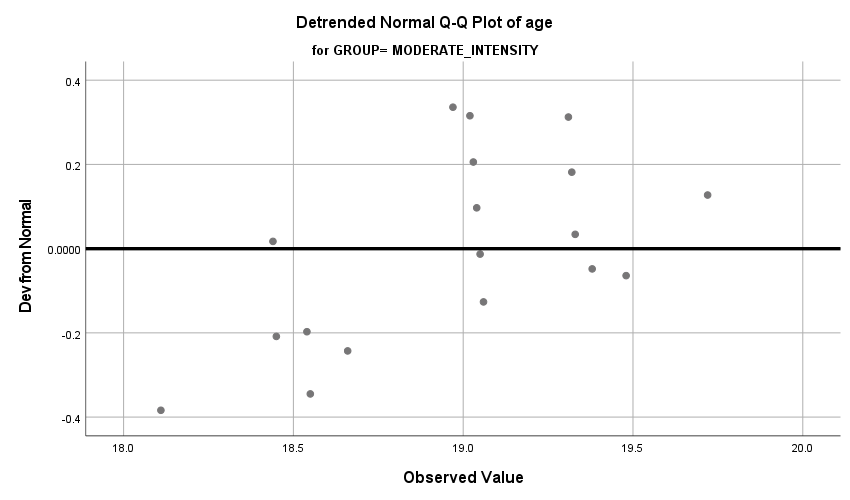


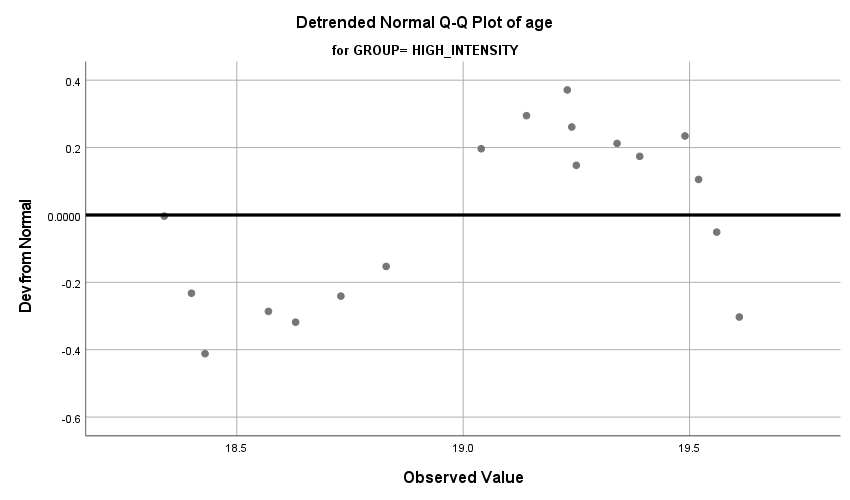


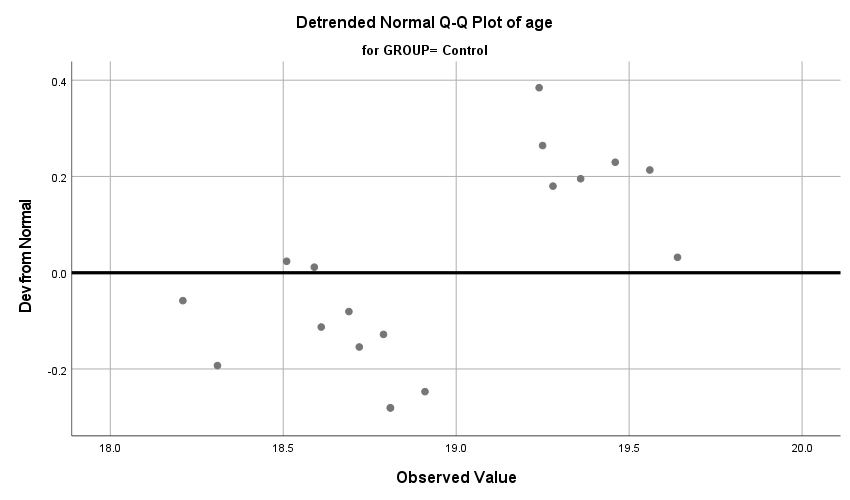


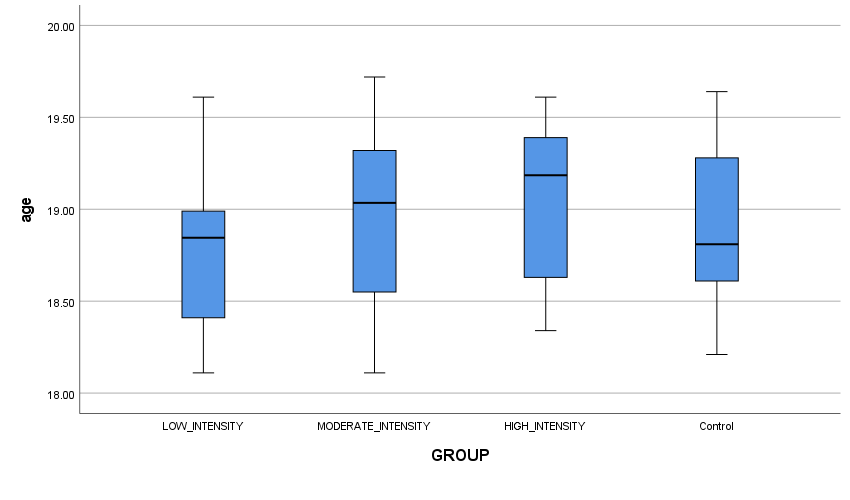


**depression**

**Stem-and-Leaf Plots**

depression Stem-and-Leaf Plot for

GROUP= LOW_INTENSITY

Frequency Stem & Leaf

4.00 0 . 2223

9.00 0 . 556778889

5.00 1 . 01234

Stem width: 10

Each leaf: 1 case(s)

depression Stem-and-Leaf Plot for

GROUP= MODERATE_INTENSITY

Frequency Stem & Leaf

5.00 0 . 23334

7.00 0 . 6678999

5.00 1 . 12234

1.00 1 . 5

Stem width: 10

Each leaf: 1 case(s)

depression Stem-and-Leaf Plot for

GROUP= HIGH_INTENSITY

Frequency Stem & Leaf

5.00 0 . 23334

8.00 0 . 57788899

4.00 1 . 0134

1.00 1 . 5

Stem width: 10

Each leaf: 1 case(s)

depression Stem-and-Leaf Plot for

GROUP= Control

Frequency Stem & Leaf

5.00 0 . 23334

8.00 0 . 56778899

5.00 1 . 01344

Stem width: 10

Each leaf: 1 case(s)

**Normal Q-Q Plots**


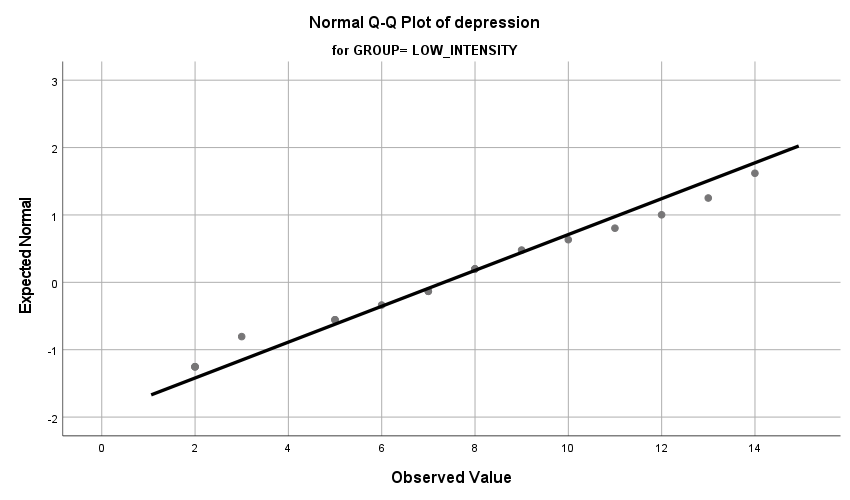


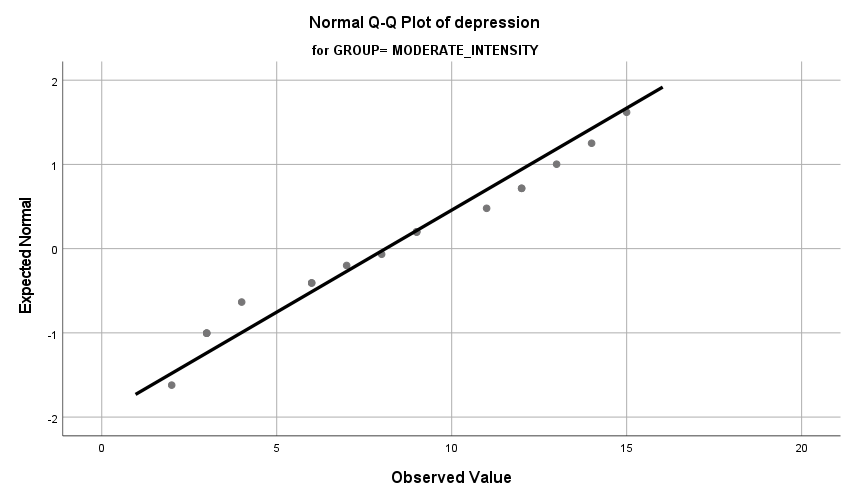


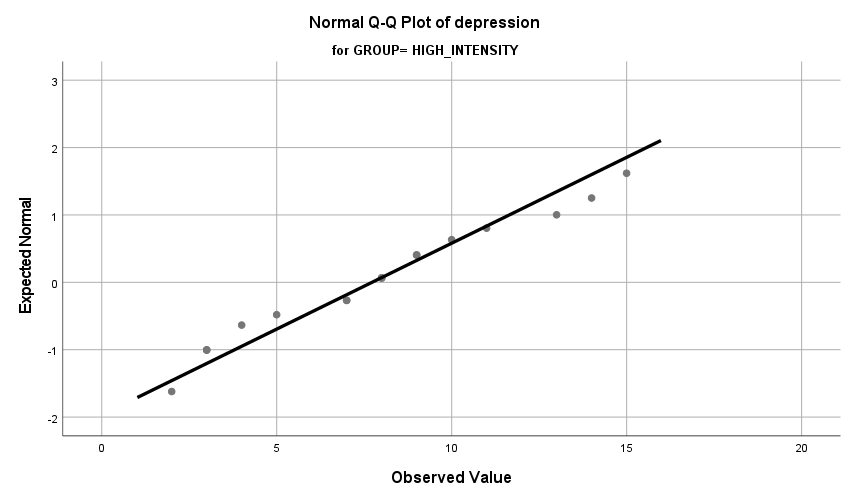


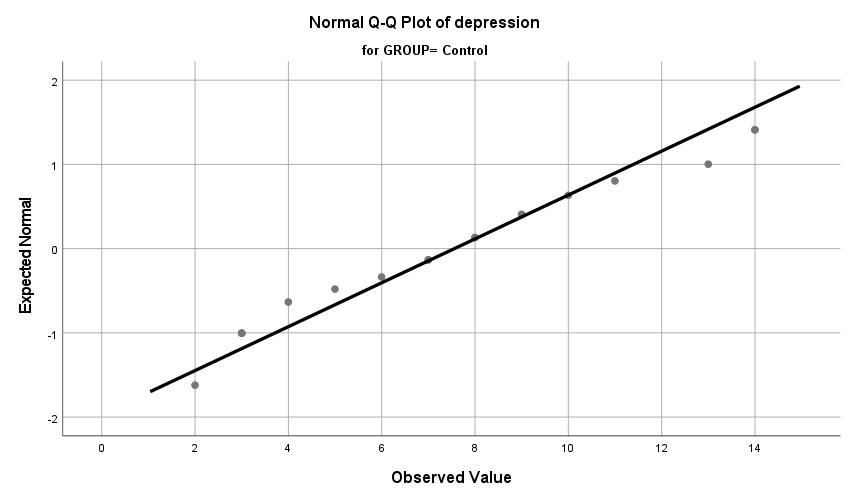


**Detrended Normal Q-Q Plots**


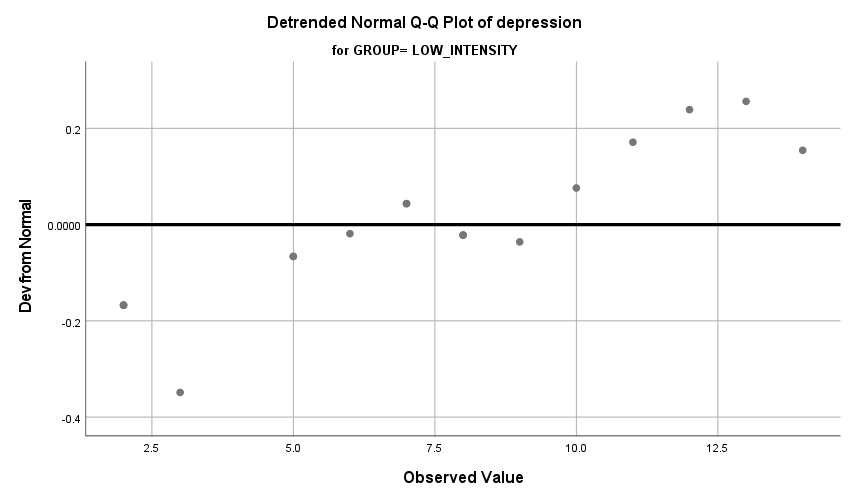


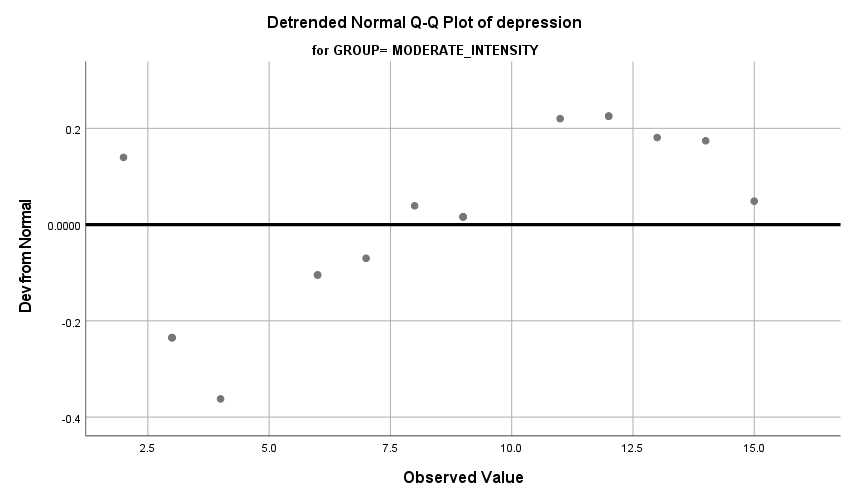


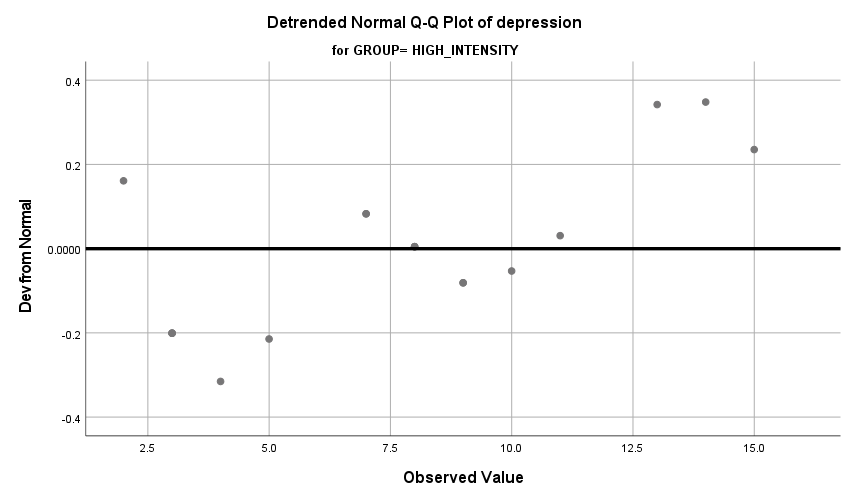


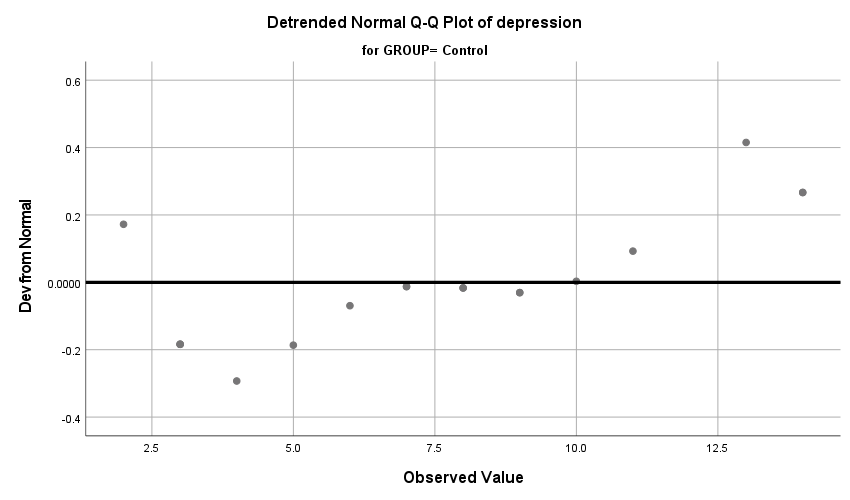


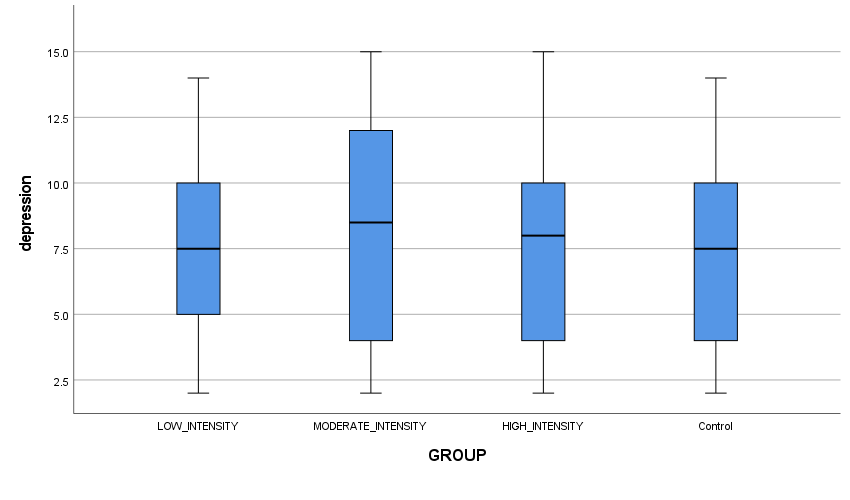


**anxiety**

**Stem-and-Leaf Plots**

anxiety Stem-and-Leaf Plot for

GROUP= LOW_INTENSITY

Frequency Stem & Leaf

1.00 0 . 4

5.00 0 . 56789

3.00 1 . 113

6.00 1 . 556789

3.00 2 . 123

Stem width: 10

Each leaf: 1 case(s)

anxiety Stem-and-Leaf Plot for

GROUP= MODERATE_INTENSITY

Frequency Stem & Leaf

1.00 0 . 4

5.00 0 . 56779

6.00 1 . 012344

3.00 1 . 679

3.00 2 . 012

Stem width: 10

Each leaf: 1 case(s)

anxiety Stem-and-Leaf Plot for

GROUP= HIGH_INTENSITY

Frequency Stem & Leaf

1.00 0 . 4

5.00 0 . 55689

5.00 1 . 01234

4.00 1 . 5588

3.00 2 . 011

Stem width: 10

Each leaf: 1 case(s)

anxiety Stem-and-Leaf Plot for

GROUP= Control

Frequency Stem & Leaf

.00 0 .

6.00 0 . 556789

5.00 1 . 01134

4.00 1 . 5689

3.00 2 . 122

Stem width: 10

Each leaf: 1 case(s)

**Normal Q-Q Plots**


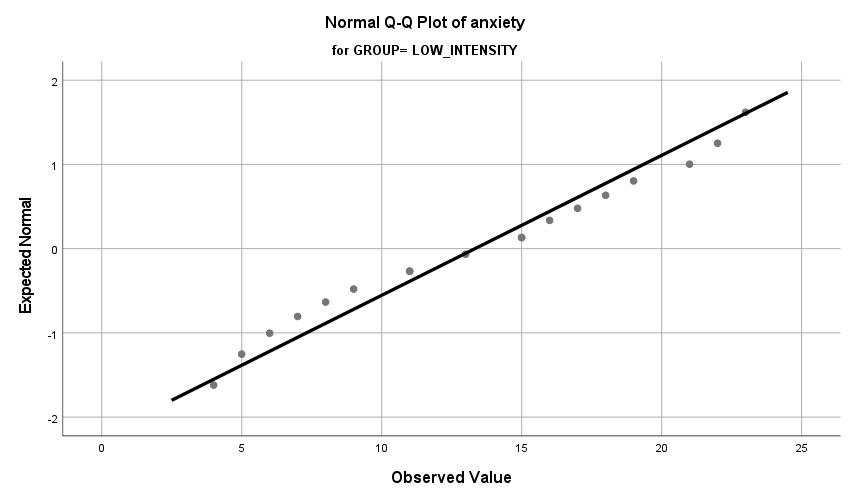


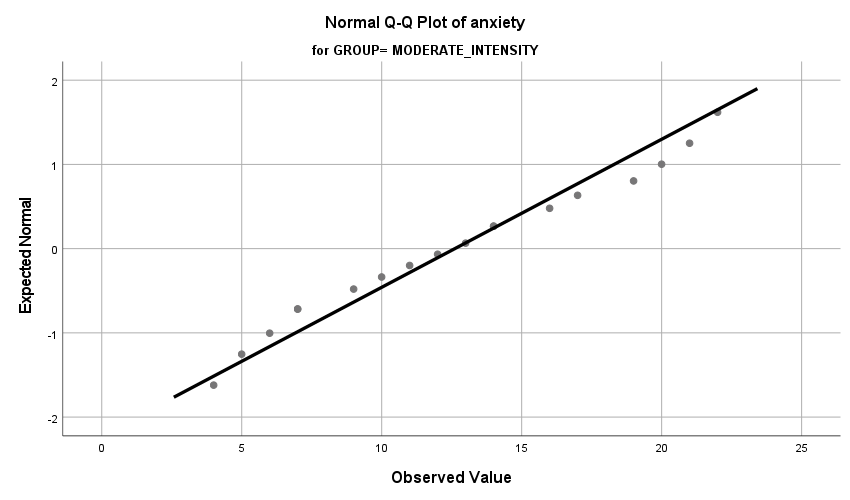


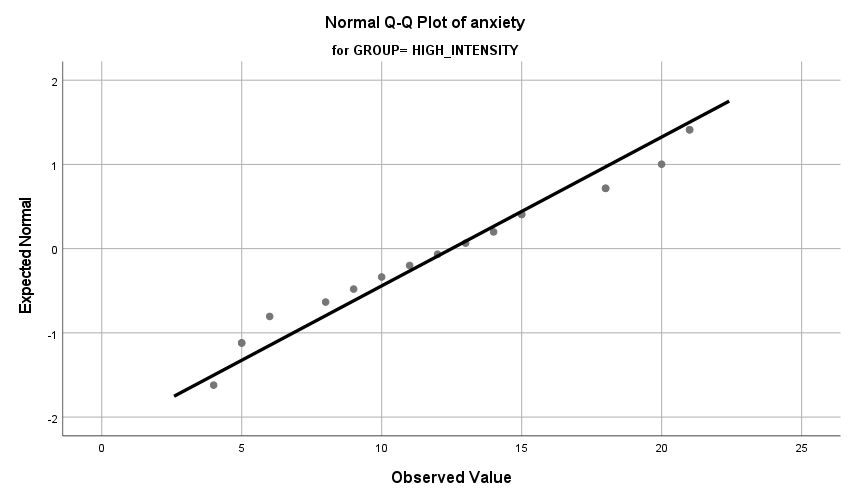


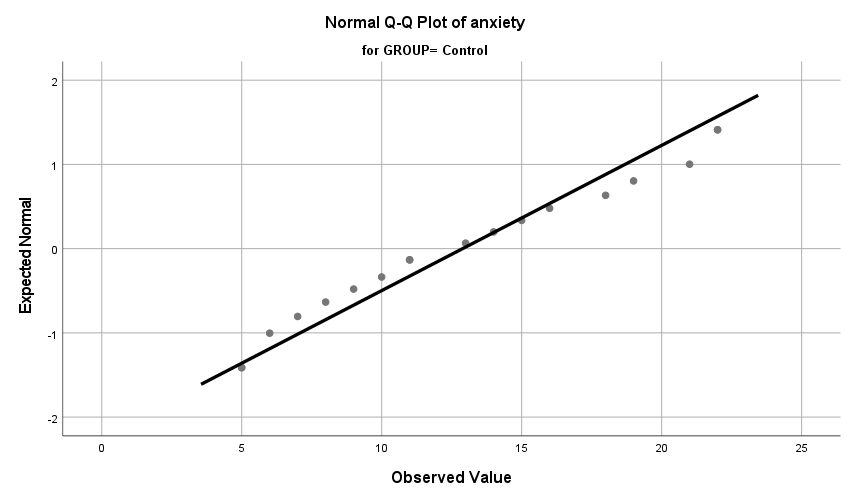


**Detrended Normal Q-Q Plots**


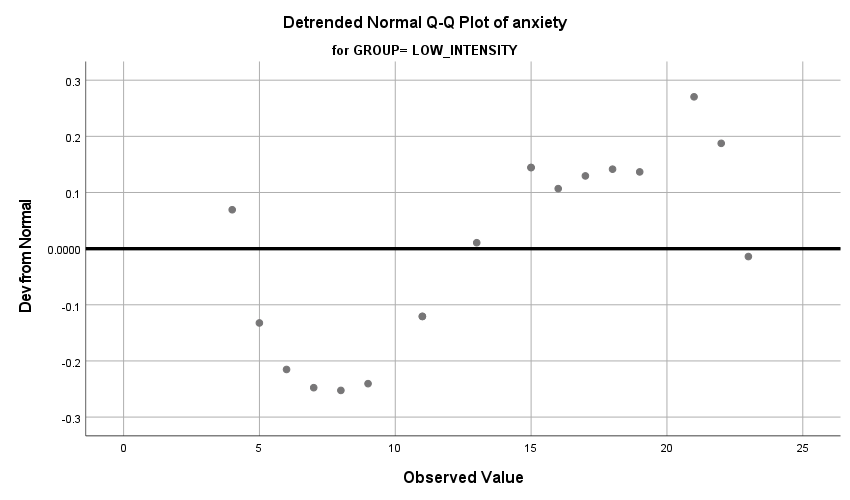


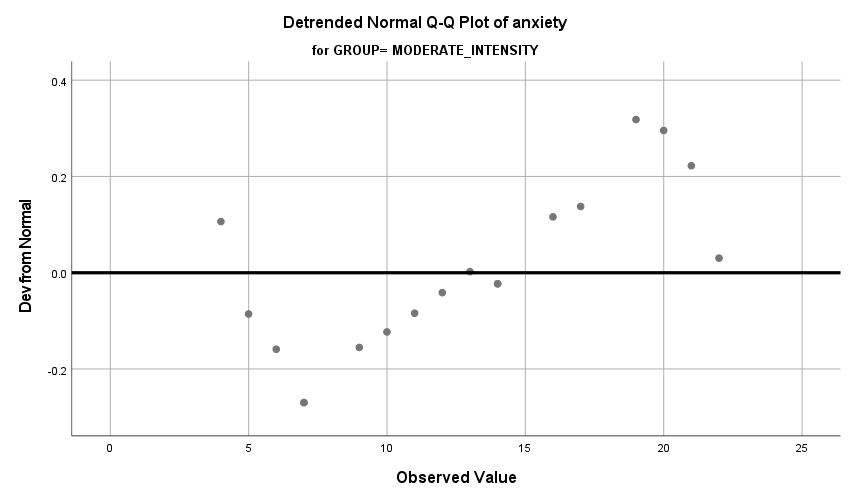


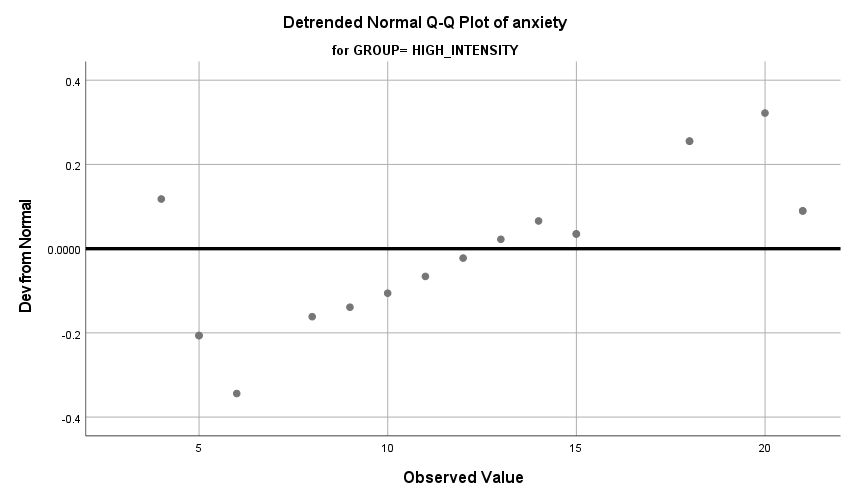


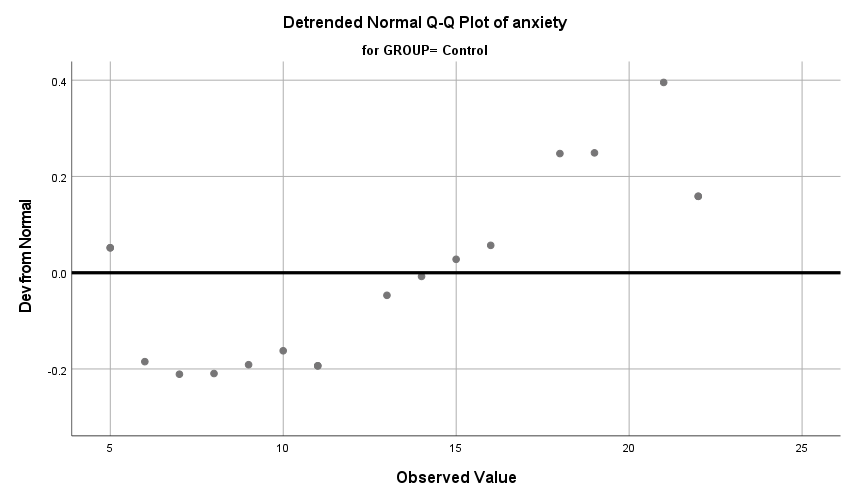


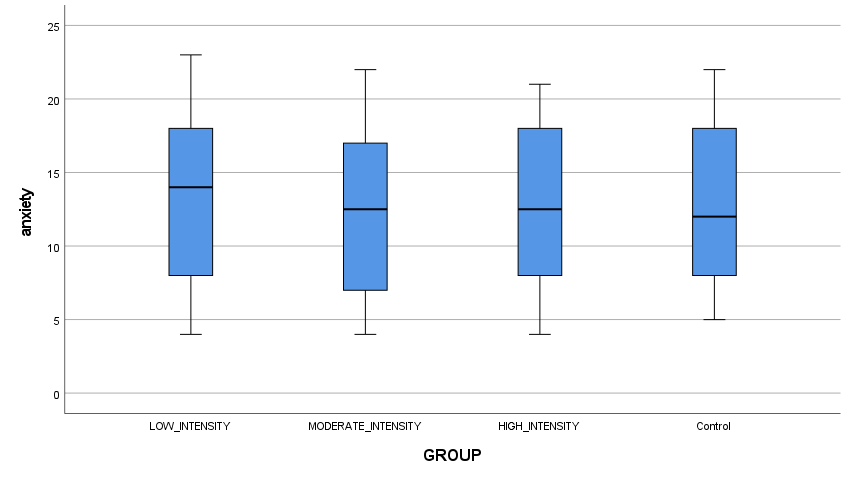


**stress**

**Stem-and-Leaf Plots**

stress Stem-and-Leaf Plot for

GROUP= LOW_INTENSITY

Frequency Stem & Leaf

1.00 0 . 4

3.00 0 . 689

3.00 1 . 123

4.00 1 . 6789

5.00 2 . 11234

2.00 2 . 56

Stem width: 10

Each leaf: 1 case(s)

stress Stem-and-Leaf Plot for

GROUP= MODERATE_INTENSITY

Frequency Stem & Leaf

.00 0 .

4.00 0 . 5678

4.00 1 . 0144

3.00 1 . 678

6.00 2 . 001234

1.00 2 . 5

Stem width: 10

Each leaf: 1 case(s)

stress Stem-and-Leaf Plot for

GROUP= HIGH_INTENSITY

Frequency Stem & Leaf

1.00 0 . 4

2.00 0 . 68

4.00 1 . 0113

4.00 1 . 6677

5.00 2 . 01233

2.00 2 . 56

Stem width: 10

Each leaf: 1 case(s)

stress Stem-and-Leaf Plot for

GROUP= Control

Frequency Stem & Leaf

1.00 0 . 4

2.00 0 . 67

4.00 1 . 0244

5.00 1 . 55668

4.00 2 . 0124

2.00 2 . 67

Stem width: 10

Each leaf: 1 case(s)

**Normal Q-Q Plots**


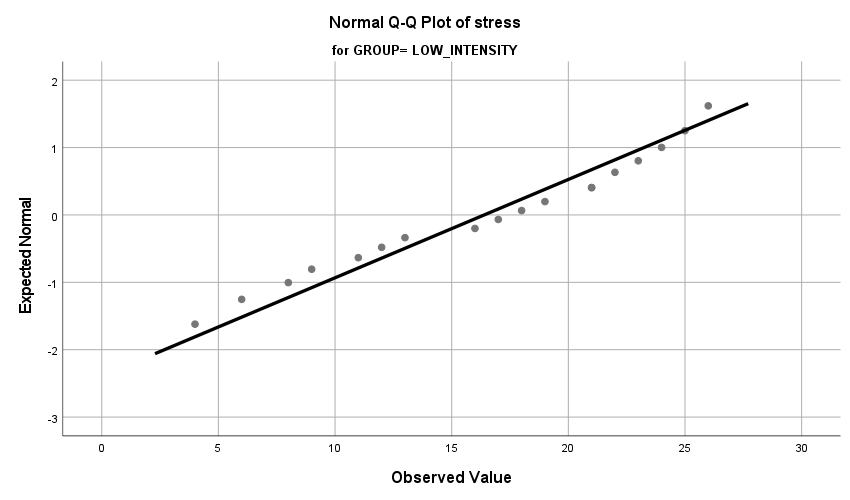


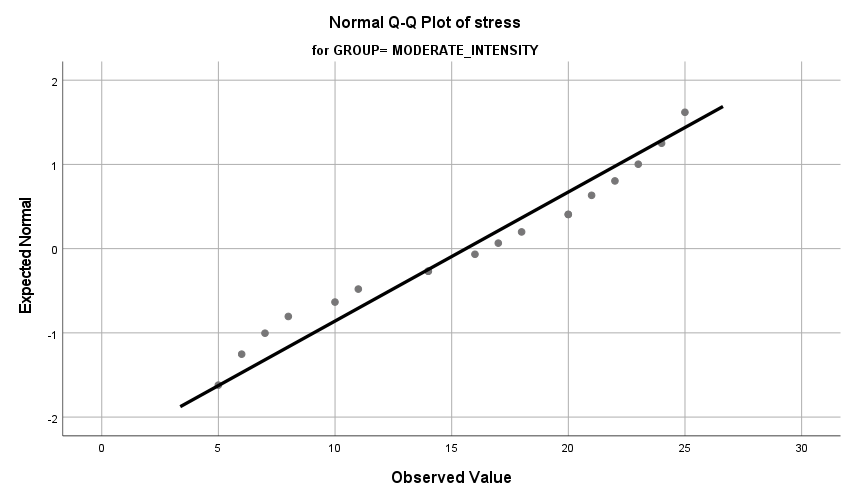


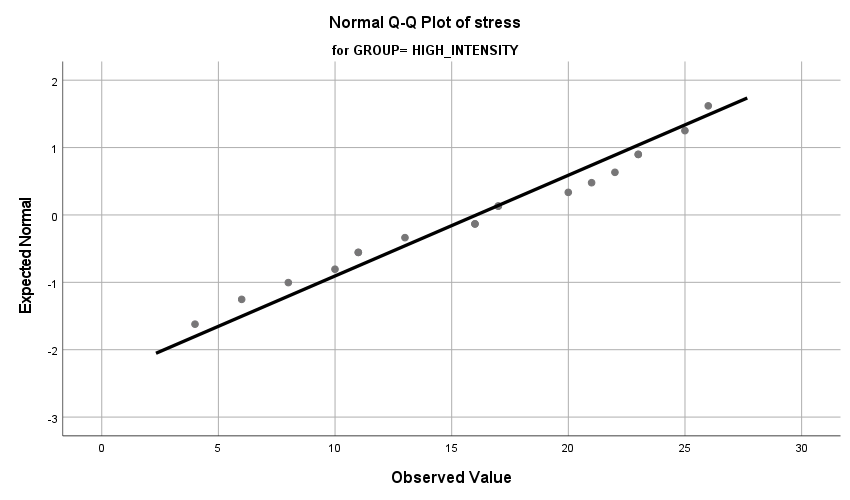


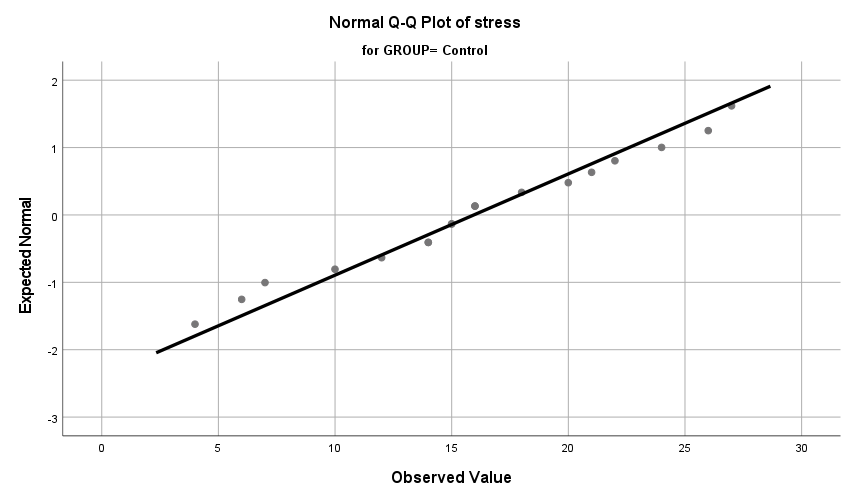


**Detrended Normal Q-Q Plots**


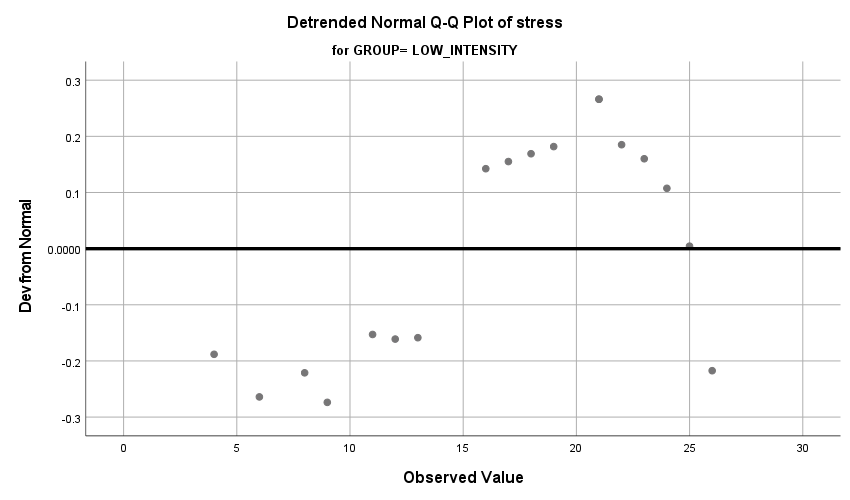


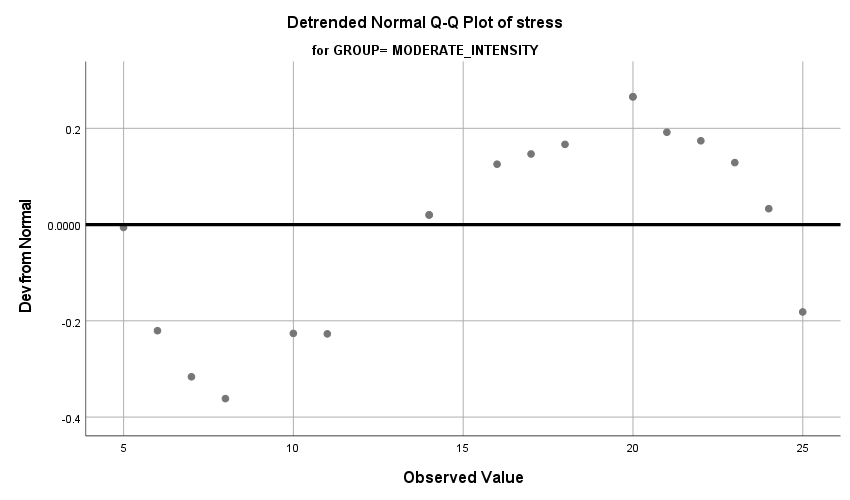


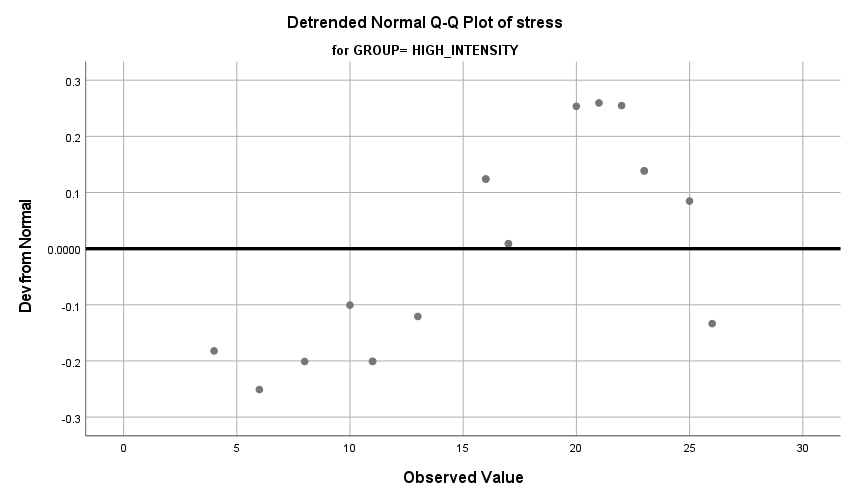


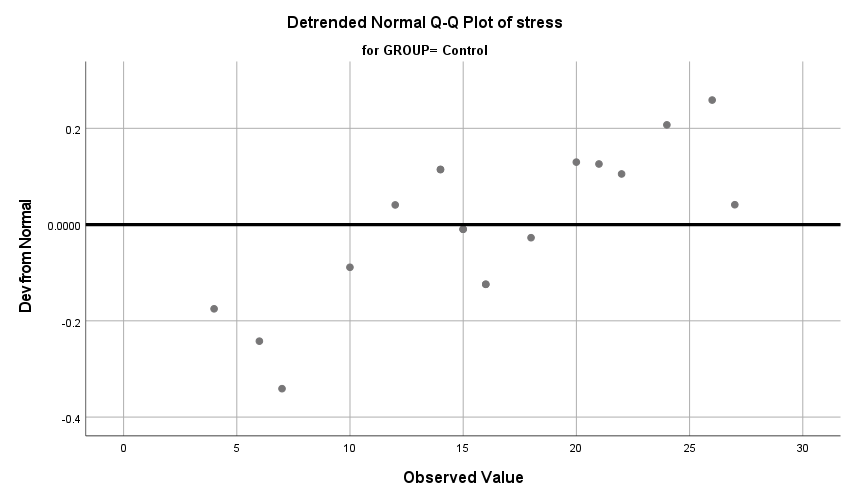


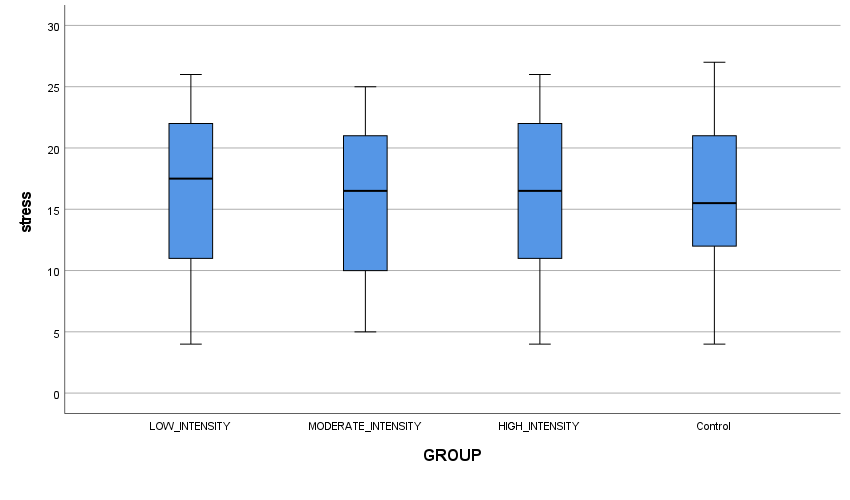


**mealtime**

**Stem-and-Leaf Plots**

mealtime Stem-and-Leaf Plot for

GROUP= LOW_INTENSITY

Frequency Stem & Leaf

1.00 2 . 9

2.00 3 . 24

4.00 3 . 6789

5.00 4 . 01234

3.00 4 . 679

3.00 5 . 012

Stem width: 10.00

Each leaf: 1 case(s)

mealtime Stem-and-Leaf Plot for

GROUP= MODERATE_INTENSITY

Frequency Stem & Leaf

1.00 3 . 3

5.00 3 . 56789

5.00 4 . 00124

3.00 4 . 558

2.00 5 . 02

2.00 5 . 57

Stem width: 10.00

Each leaf: 1 case(s)

mealtime Stem-and-Leaf Plot for

GROUP= HIGH_INTENSITY

Frequency Stem & Leaf

3.00 3 . 234

5.00 3 . 56789

4.00 4 . 0123

3.00 4 . 568

2.00 5 . 03

1.00 5 . 5

Stem width: 10.00

Each leaf: 1 case(s)

mealtime Stem-and-Leaf Plot for

GROUP= Control

Frequency Stem & Leaf

3.00 3 . 234

4.00 3 . 5789

4.00 4 . 0123

4.00 4 . 5678

3.00 5 . 123

Stem width: 10.00

Each leaf: 1 case(s)

**Normal Q-Q Plots**


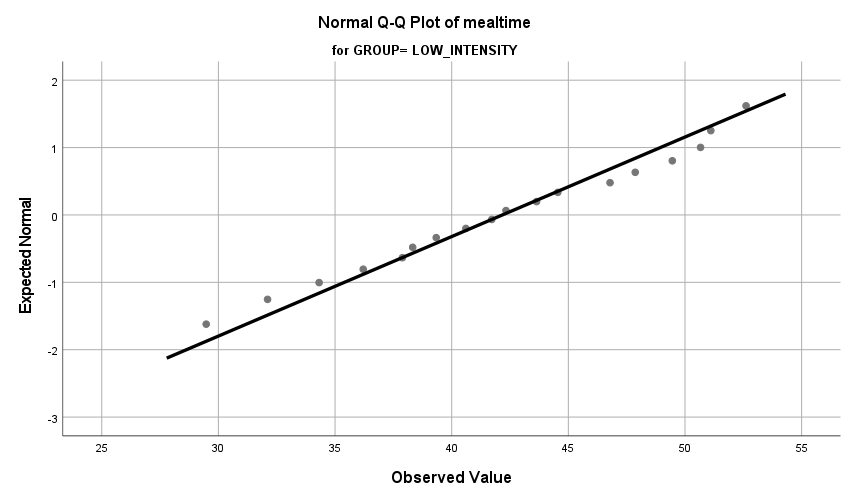


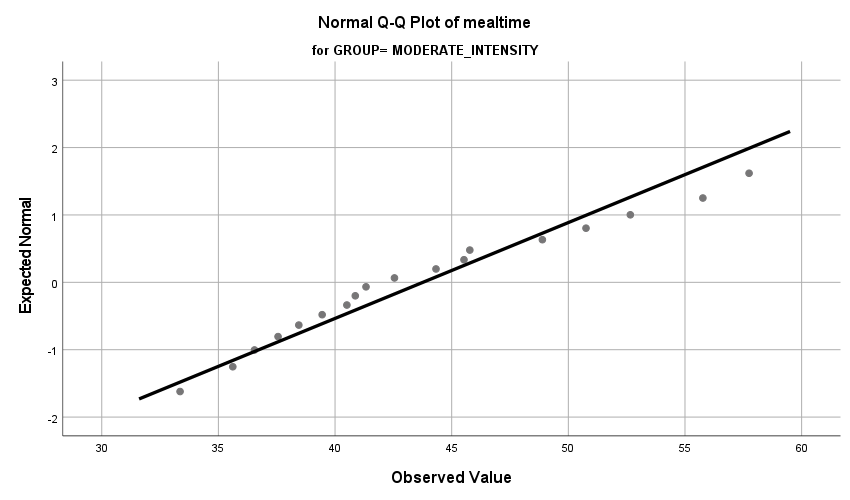


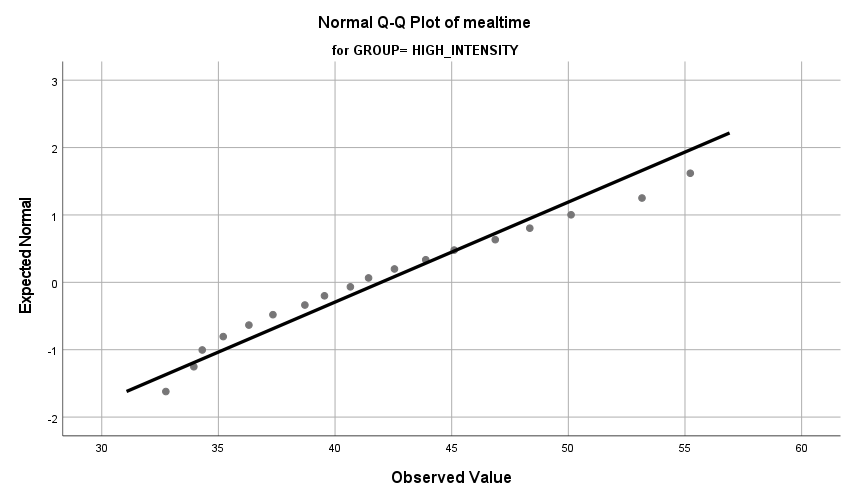


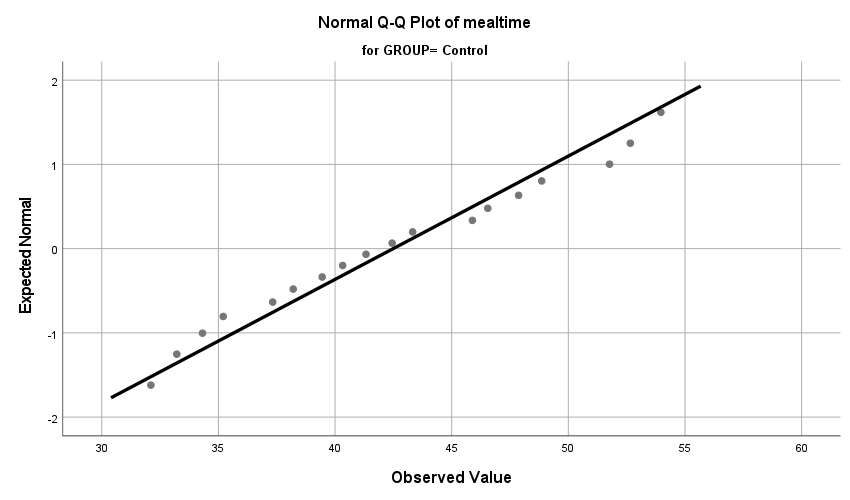


**Detrended Normal Q-Q Plots**


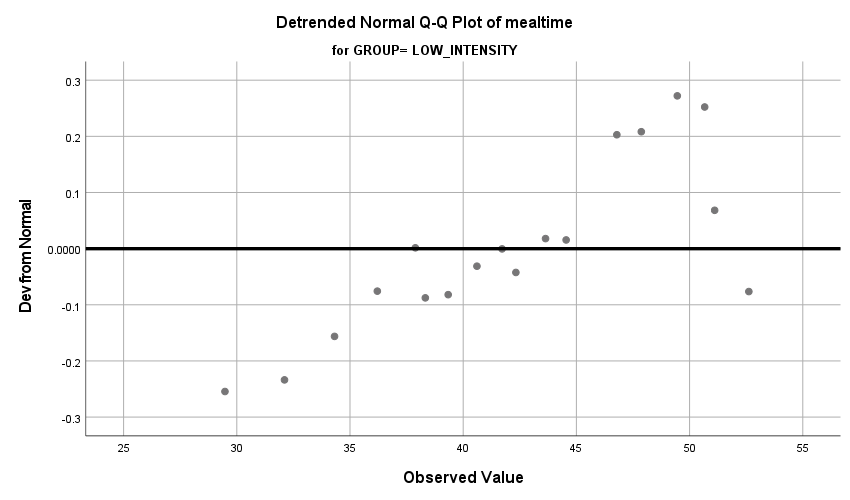


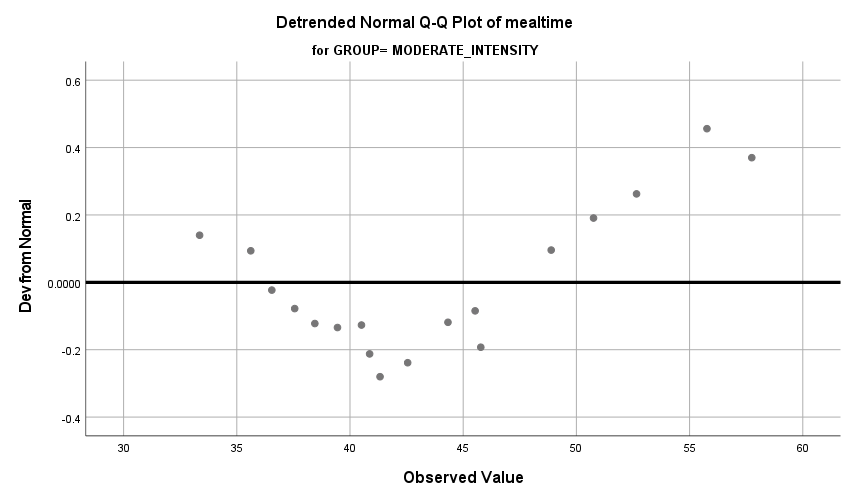


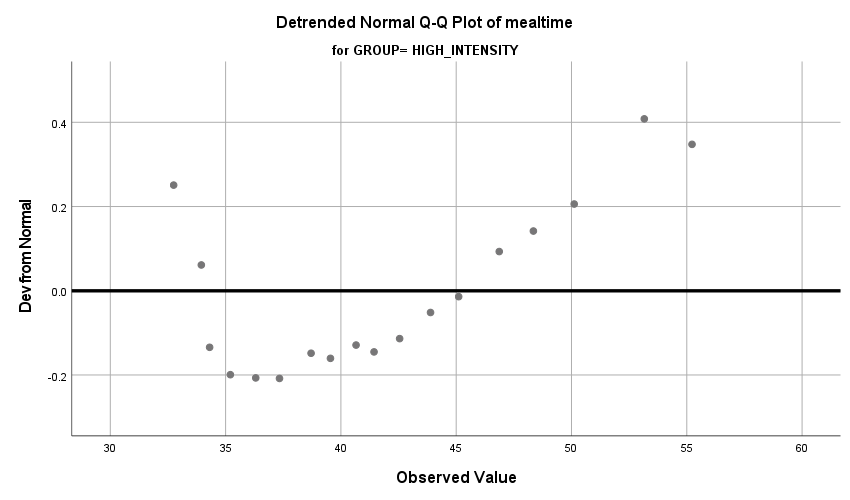


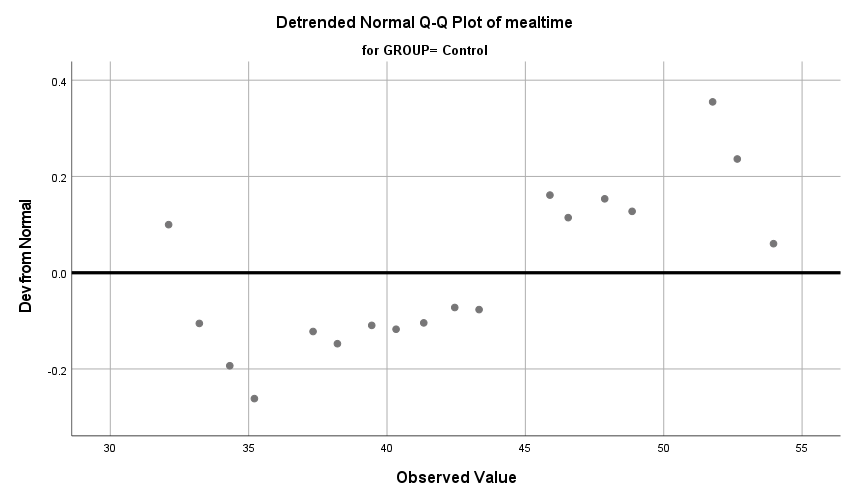


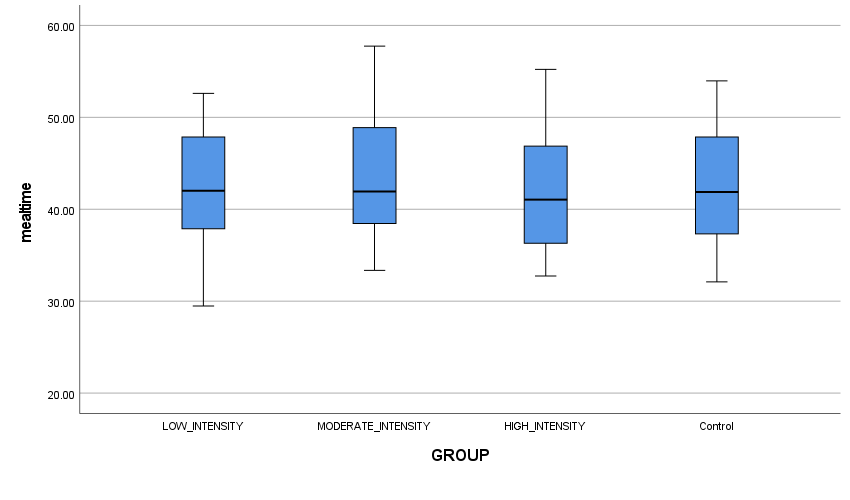

Supplement: Supplementary file 1 [file Supplementary_file_1.docx]
