# Supplementary material for: Impact of resistance training intensity on body composition and nutritional intake among college women with overweight and obesity: a cluster randomized controlled trial
Source: Front Public Health. 2025 May 30;13:1589036. doi: 10.3389/fpubh.2025.1589036 (PMC12162680; doi:10.3389/fpubh.2025.1589036)
Supplement: Supplementary file 3 [file Supplementary_file_3.docx]

Supplementary Materials


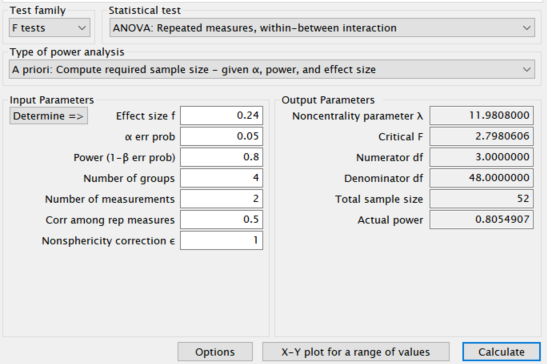

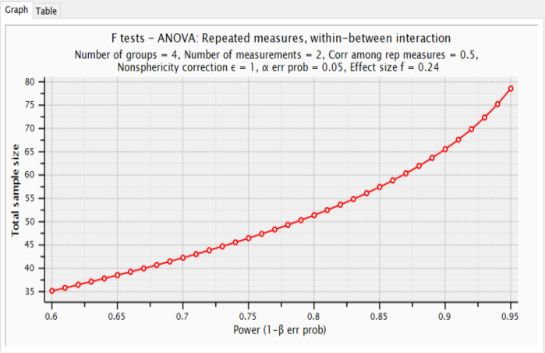


**Figure 1. Sample Size Calculation Based on the Repeated measure Within-between Interaction**


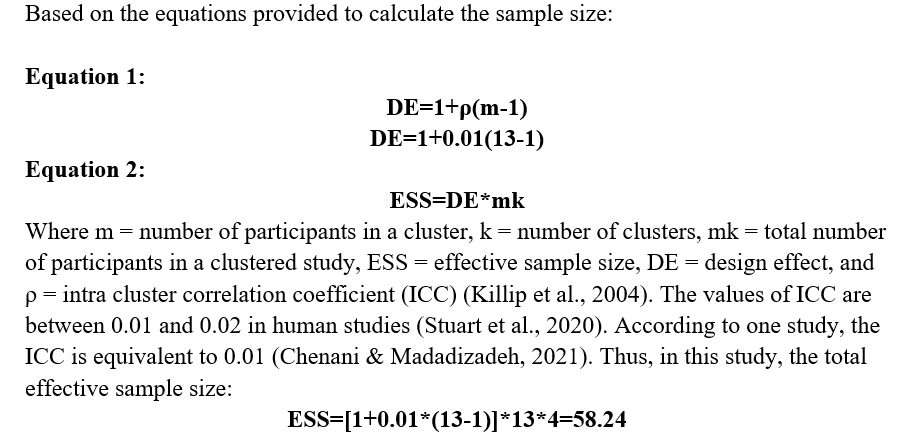


**Figure 2. Sample Size Calculation Using Cluster Randomized Controlled Trial Design**

**Figure 3. The mean of BMI for all groups across the time**

**Figure 4. The mean of body fat percentage for all groups across the time**

Figure 5. The mean of waist circumference for all groups across the time

Figure 6. The mean of energy intake for all groups across the time

Figure 7. The mean of protein intake for all groups across the time

Figure 8. The mean of fat intake for all groups across the time

Figure 9. The mean of carbohydrate intake for all groups across the time

**Figure 10. The mean of chest press 1RM for all groups across the time**

**Figure 11. The mean of squat 1RM for all groups across the time**

**Table 15. Twelve-Week training program**

| **Low intensity Moderate intensity High intensity Control**  **45%-50% 1RM 60%-65% 1RM 75%-80% 1RM** | |
| --- | --- |
| Warm Up (10 mins): | |
| Jogging and running indoor or stationary cycling outdoor, low intensity (5 minutes)  Muscle stretching: the Chinese eighth set of radio gymnastics (each movement 4×8 beats, total of 5 minutes)  1.Stretch exercise  2.Chest enlargement exercise  3.Kicking exercise  4.Body side exercise  5.Body rotation exercise  6.Whole body exercise  7.Jump exercise (without apparatus) | |
| Main Training (1-hour): | |
| Resistance Training：  1.Chest press  2.Lat pull down  3.Shoulder press  4.Squat  5.Leg extension  6.Leg curl  7.Bicep curl  8.Triceps extension  9.Abdominal crunches  10.Lower back exercises | Normal P.E. Course：  Carry out general teaching content according to the college syllabus. |
| Cool Down (5 mins)： | |
| Muscle stretching: the Chinese eighth set of radio gymnastics (each movement 4×8 beats, total of 5 minutes)  1.Stretch exercise  2.Chest enlargement exercise  3.Kicking exercise  4.Body side exercise  5.Body rotation exercise  6.Whole body exercise  7.Jump exercise (without apparatus)  8.Finishing exercise | |

**Table 16. Training prescription by week**

| **Week** | **Frequency** | **Intensity** | **Sets** | **Repetitions** | **Rest between sets (mins)** | **Whether retest 1RM** | **Duration (mins)** |
| --- | --- | --- | --- | --- | --- | --- | --- |
| 1^st^-2^nd^ | 3/week | Low intensity  (45-50% 1RM) | 2 | 16-18 | 0.5 | Yes for 1^st^ week | Total 75  (60 main training, 10 warm-up, and 5 cool-down) |
|  |  | Moderate intensity  (60-65% 1RM) |  | 10-12 | 1 |  |  |
|  |  | High intensity  (75-80% 1RM) |  | 6-8 | 1.5 |  |  |
|  |  | Control group | Recovery exercises, learn and practice queue formation | | | |  |
| 3^rd^-4^th^ | 3/week | Low intensity  (45-50% 1RM) | 2 | 16-18 | 0.5 | No | Total 75  (60 main training, 10 warm-up, and 5 cool-down) |
|  |  | Moderate intensity  (60-65% 1RM) |  | 10-12 | 1 |  |  |
|  |  | High intensity  (75-80% 1RM) |  | 6-8 | 1.5 |  |  |
|  |  | Control group | Learn and practice queue formation | | | |  |
| 5^th^-6^th^ | 3/week | Low intensity  (45-50% 1RM) | 2 | 16-18 | 0.5 | Yes for 5^th^ week | Total 75  (60 main training, 10 warm-up, and 5 cool-down) |
|  |  | Moderate intensity  (60-65% 1RM) |  | 10-12 | 1 |  |  |
|  |  | High intensity  (75-80% 1RM) |  | 6-8 | 1.5 |  |  |
|  |  | Control group | Learn and practice basketball dribble | | | |  |
| 7^th^-8^th^ | 3/week | Low intensity  (45-50% 1RM) | 2 | 16-18 | 0.5 | No | Total 75  (60 main training, 10 warm-up, and 5 cool-down) |
|  |  | Moderate intensity  (60-65% 1RM) |  | 10-12 | 1 |  |  |
|  |  | High intensity  (75-80% 1RM) |  | 6-8 | 1.5 |  |  |
|  |  | Control group | Learn and practice basketball pass and catch | | | |  |
| 9^th^-10^th^ | 3/week | Low intensity  (45-50% 1RM) | 3 | 16-18 | 0.5 | Yes for 9^th^ week | Total 75  (60 main training, 10 warm-up, and 5 cool-down) |
|  |  | Moderate intensity  (60-65% 1RM) |  | 10-12 | 1 |  |  |
|  |  | High intensity  (75-80% 1RM) |  | 6-8 | 1.5 |  |  |
|  |  | Control group | Learn and practice basketball layup | | | |  |
| 11^th^-12th | 3/week | Low intensity  (45-50% 1RM) | 3 | 16-18 | 0.5 | No | Total 75  (60 main training, 10 warm-up, and 5 cool-down) |
|  |  | Moderate intensity  (60-65% 1RM) |  | 10-12 | 1 |  |  |
|  |  | High intensity  (75-80% 1RM) |  | 6-8 | 1.5 |  |  |
|  |  | Control group | Learn and practice basketball shooting | | | |  |

**Table 17. Training volume across groups**

| Group | Exercise | Weight (kg) | Repetitions | Volume per set (kg) | Total volume per set (kg) | Total volume per session during 1-8/9-12 week (kg) |
| --- | --- | --- | --- | --- | --- | --- |
| LI | Chest Press | 10.7 | 17 | 181.9 | 1497.7 | 2995.4/4493.1 |
| LI | Lat Pulldown | 12.0 | 17 | 204 |  |  |
| LI | Shoulder Press | 8.1 | 17 | 137.7 |  |  |
| LI | Squat | 16.2 | 17 | 275.4 |  |  |
| LI | Leg Extension | 12.2 | 17 | 207.4 |  |  |
| LI | Leg Curl | 8.0 | 17 | 136 |  |  |
| LI | Bicep Curl | 4.0 | 17 | 68 |  |  |
| LI | Triceps Extension | 5.3 | 17 | 90.1 |  |  |
| LI | Abdominal Crunch | 3.6 | 17 | 61.2 |  |  |
| LI | Lower Back Exercises | 8.0 | 17 | 136 |  |  |
| MI | Chest Press | 16.5 | 11 | 181.5 | 1496 | 2992/4488 |
| MI | Lat Pulldown | 18.6 | 11 | 204.6 |  |  |
| MI | Shoulder Press | 12.4 | 11 | 136.4 |  |  |
| MI | Squat | 24.8 | 11 | 272.8 |  |  |
| MI | Leg Extension | 18.9 | 11 | 207.9 |  |  |
| MI | Leg Curl | 12.4 | 11 | 136.4 |  |  |
| MI | Bicep Curl | 6.2 | 11 | 68.2 |  |  |
| MI | Triceps Extension | 8.3 | 11 | 91.3 |  |  |
| MI | Abdominal Crunch | 5.5 | 11 | 60.5 |  |  |
| MI | Lower Back Exercises | 12.4 | 11 | 136.4 |  |  |
| HI | Chest Press | 26.0 | 7 | 182 | 1495.2 | 2990.4/4485.6 ﻿ |
| HI | Lat Pulldown | 29.2 | 7 | 204.4 |  |  |
| HI | Shoulder Press | 19.5 | 7 | 136.5 |  |  |
| HI | Squat | 39.1 | 7 | 273.7 |  |  |
| HI | Leg Extension | 29.4 | 7 | 205.8 |  |  |
| HI | Leg Curl | 19.5 | 7 | 136.5 |  |  |
| HI | Bicep Curl | 9.7 | 7 | 67.9 |  |  |
| HI | Triceps Extension | 13.0 | 7 | 91 |  |  |
| HI | Abdominal Crunch | 8.7 | 7 | 60.9 |  |  |
| HI | Lower Back Exercises | 19.5 | 7 | 136.5 |  |  |

**Table 18. Mean and SE for adjusted mean of HR among groups**

| Group | Mean | Std. Error |
| --- | --- | --- |
| LI | 113.78 | 0.56 |
| MI | 125.01 | 0.56 |
| HI | 137.17 | 0.56 |
| CG | 106.60 | 0.56 |

**Adjusted mean of HR, Covariates: BMI, VO2max*

**Table 19. Results of ANCOVA for HR**

| Source | Type III Sum of Squares | df | Mean Square | F | Sig. |
| --- | --- | --- | --- | --- | --- |
| VO_2_max* | 1.339 | 1 | 1.339 | 0.242 | 0.624 |
| BMI* | 462.65 | 1 | 462.65 | 83.666 | <.001 |
| Group | 9655.215 | 3 | 3218.405 | 582.021 | <.001 |

** Covariates*

Table 20. Normality test for demographic and confounding variable

|  | Group | Kolmogorov-Smirnova | | Shapiro-Wilk | | | |
| --- | --- | --- | --- | --- | --- | --- | --- |
|  |  | Statistic | df | Sig. | Statistic | df | Sig. |
| Pre. height | LI*** | 0.167 | 18 | .200 | 0.957 | 18 | 0.547 |
|  | MI*** | 0.167 | 18 | .200 | 0.937 | 18 | 0.259 |
|  | HI*** | 0.167 | 18 | .200 | 0.957 | 18 | 0.547 |
|  | CG*** | 0.167 | 18 | .200 | 0.957 | 18 | 0.547 |
| Pre. weight | LI*** | 0.098 | 18 | .200 | 0.991 | 18 | 1.000 |
|  | MI*** | 0.121 | 18 | .200 | 0.967 | 18 | 0.732 |
|  | HI*** | 0.106 | 18 | .200 | 0.965 | 18 | 0.696 |
|  | CG*** | 0.161 | 18 | .200 | 0.944 | 18 | 0.335 |
| Pre. age | LI*** | 0.132 | 18 | .200 | 0.962 | 18 | 0.634 |
|  | MI*** | 0.167 | 18 | .200 | 0.957 | 18 | 0.549 |
|  | HI*** | 0.169 | 18 | 0.187 | 0.914 | 18 | 0.101 |
|  | CG*** | 0.166 | 18 | .200 | 0.951 | 18 | 0.440 |
| Pre. mealtime | LI*** | 0.086 | 18 | .200 | 0.973 | 18 | 0.853 |
|  | MI*** | 0.135 | 18 | .200 | 0.952 | 18 | 0.465 |
|  | HI*** | 0.087 | 18 | .200 | 0.954 | 18 | 0.489 |
|  | CG*** | 0.079 | 18 | .200 | 0.960 | 18 | 0.609 |
| Pre. menstrual time | LI*** | 0.142 | 18 | .200 | 0.947 | 18 | 0.380 |
|  | MI*** | 0.165 | 18 | .200 | 0.939 | 18 | 0.278 |
|  | HI*** | 0.098 | 18 | .200 | 0.964 | 18 | 0.678 |
|  | CG*** | 0.108 | 18 | .200 | 0.968 | 18 | 0.763 |
|  | LI*** | 0.128 | 18 | .200 | 0.950 | 18 | 0.427 |
| Pre. menstrual cycle | MI*** | 0.121 | 18 | .200 | 0.976 | 18 | 0.906 |
|  | HI*** | 0.199 | 18 | .058 | 0.958 | 18 | 0.565 |
|  | CG*** | 0.144 | 18 | .200 | 0.965 | 18 | 0.696 |

**Either Kolmogorov-Smirnova or Shapiro-Wilk more than 0.05 means normal distribution*
