# Supplementary material for: Impact of resistance training intensity on body composition and nutritional intake among college women with overweight and obesity: a cluster randomized controlled trial
Source: Front Public Health. 2025 May 30;13:1589036. doi: 10.3389/fpubh.2025.1589036 (PMC12162680; doi:10.3389/fpubh.2025.1589036)
Supplement: Supplementary file 4 [file Supplementary_file_4.pdf]

Supplementary Materials

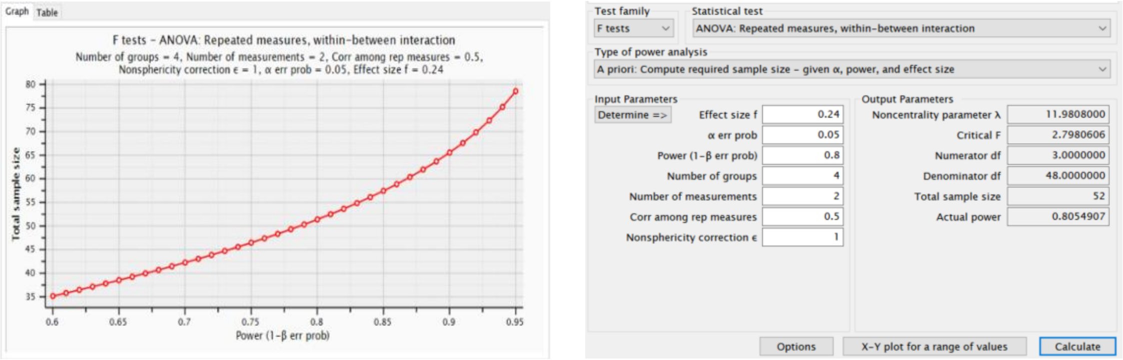

Figure 1. Sample Size Calculation Based on the Repeated measure Within-between Interaction

Based on the equations provided to calculate the sample size:

Equation 1:

$$DE=1+\rho(m-1)$$

$$DE=1+0.01(13-1)$$

Equation 2:

$$ESS=DE*mk$$

Where m = number of participants in a cluster, k = number of clusters, mk = total number of participants in a clustered study, ESS = effective sample size, DE = design effect, and ρ = intra cluster correlation coefficient (ICC) (Killip et al., 2004). The values of ICC are between 0.01 and 0.02 in human studies (Stuart et al., 2020). According to one study, the ICC is equivalent to 0.01 (Chenani & Madadizadeh, 2021). Thus, in this study, the total effective sample size:

$$ESS=[1+0.01*(13-1)]*13*4=58.24$$

Figure 2. Sample Size Calculation Using Cluster Randomized Controlled Trial Design

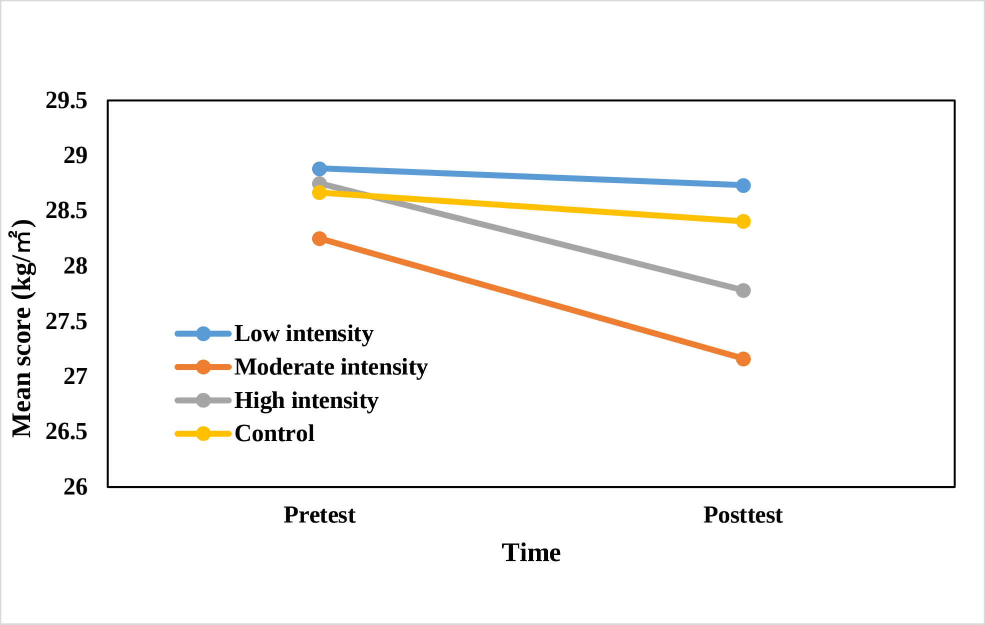

Figure 3. The mean of BMI for all groups across the time

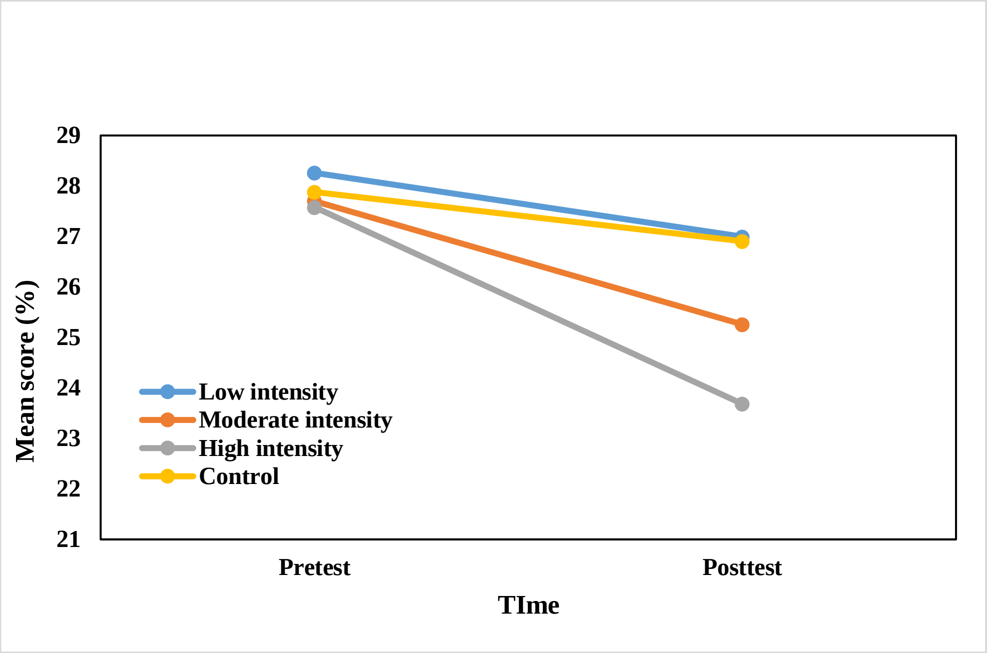

Figure 4. The mean of body fat percentage for all groups across the time

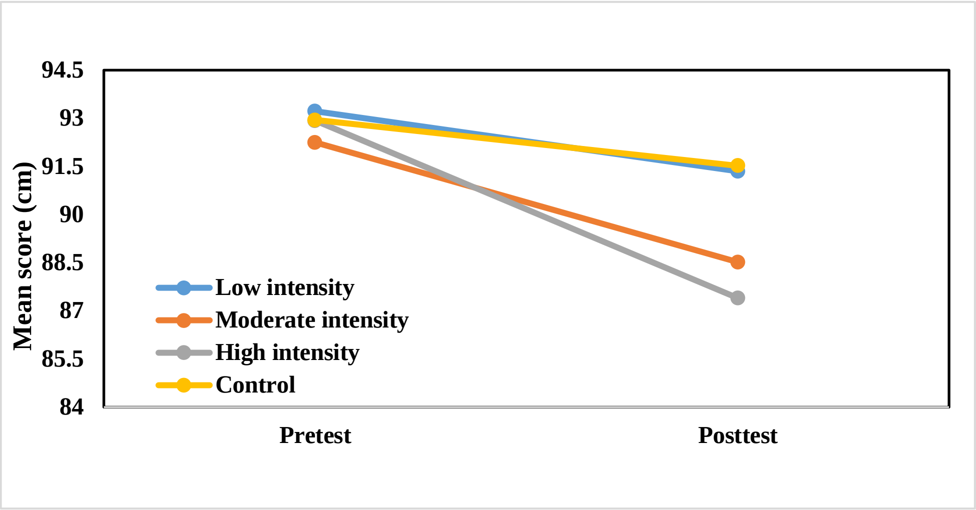

Figure 5. The mean of waist circumference for all groups across the time

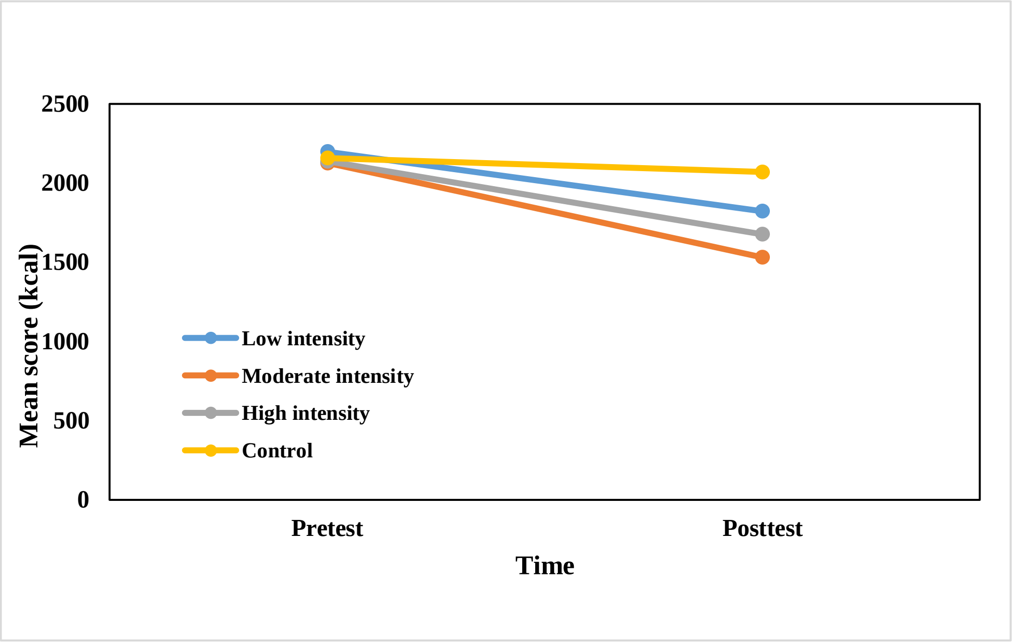

Figure 6. The mean of energy intake for all groups across the time

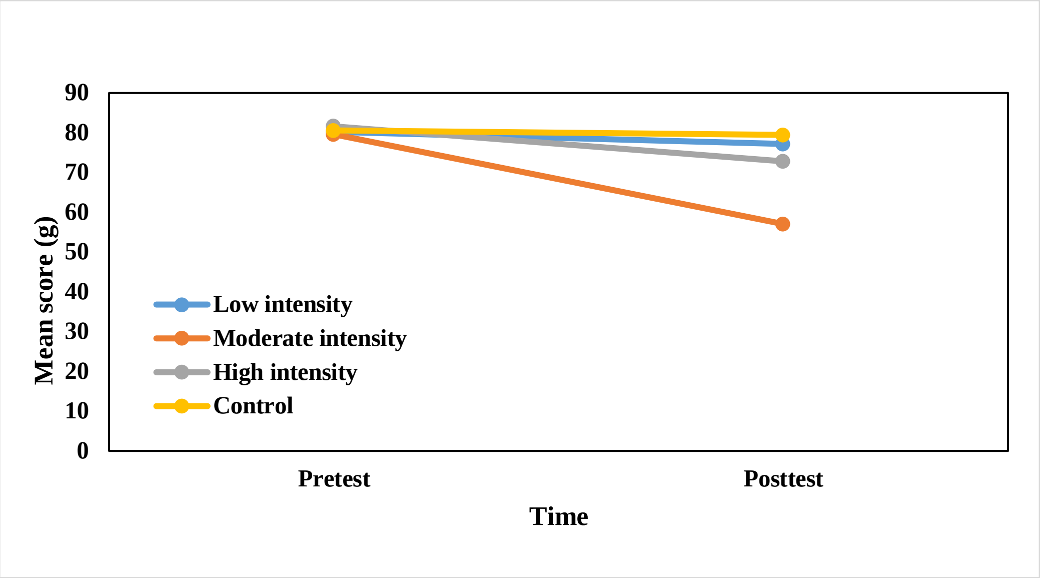

Figure 7. The mean of protein intake for all groups across the time

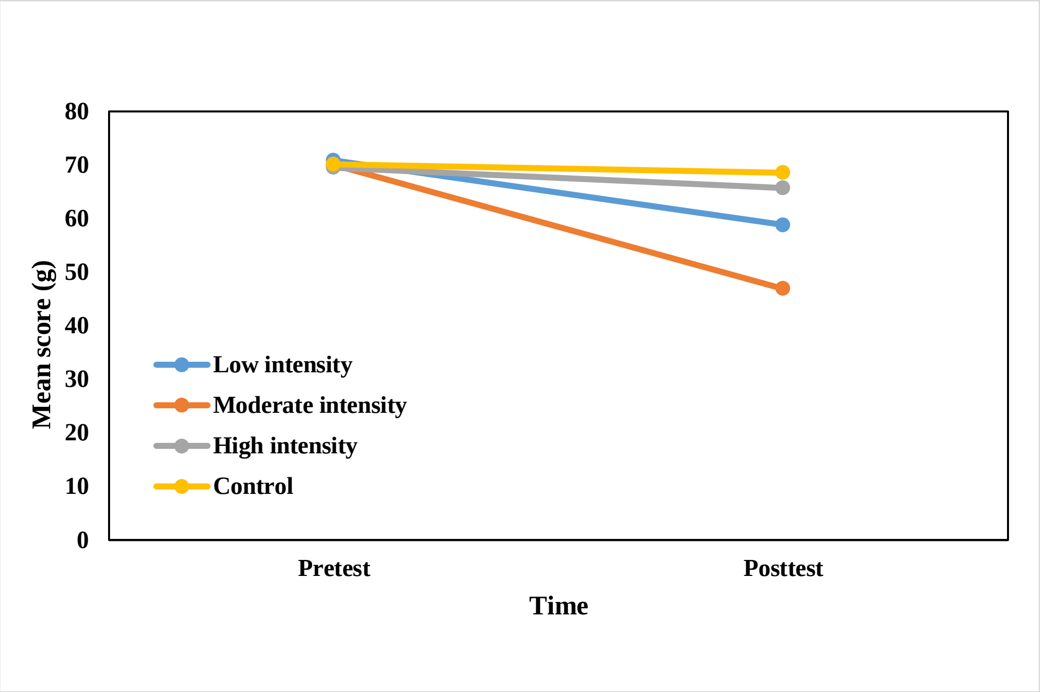

Figure 8. The mean of fat intake for all groups across the time

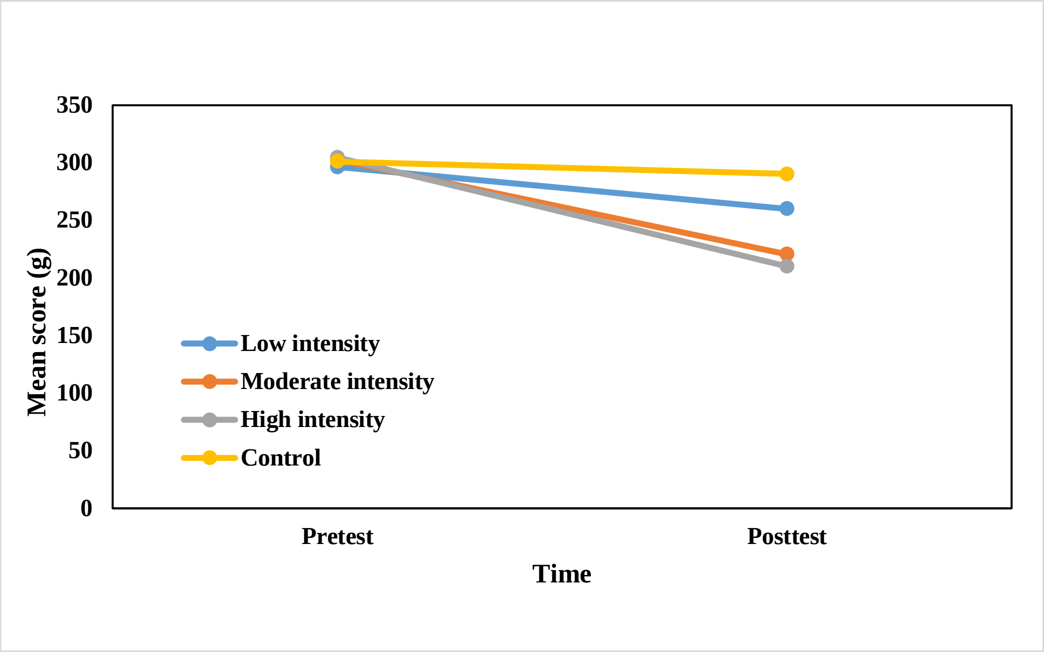

Figure 9. The mean of carbohydrate intake for all groups across the time

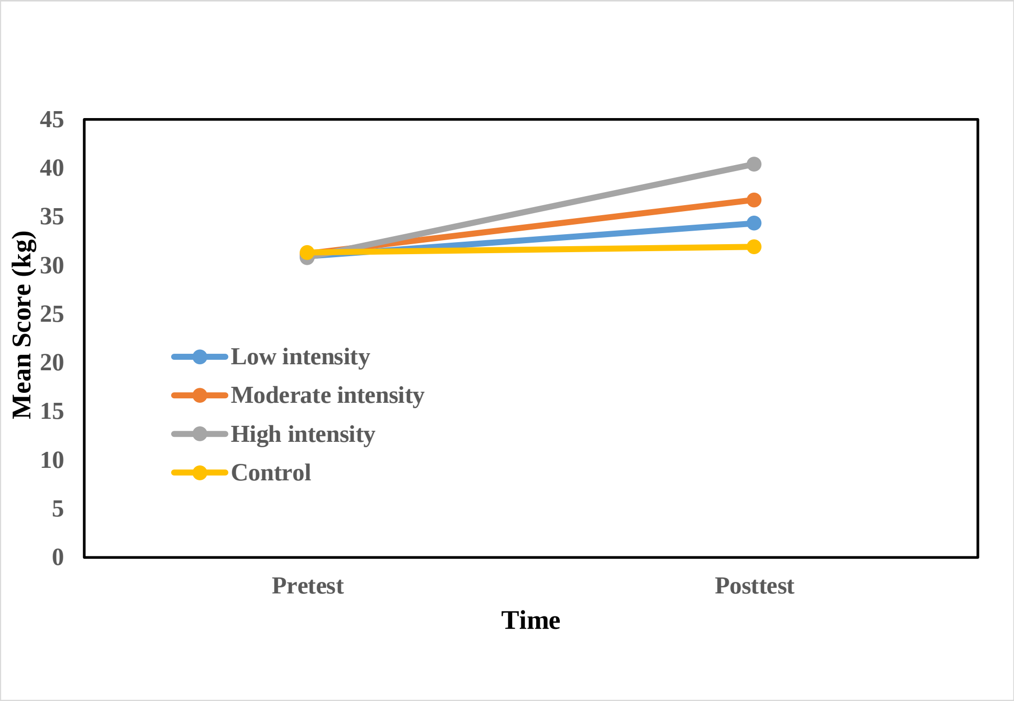

Figure 10. The mean of chest press 1RM for all groups across the time

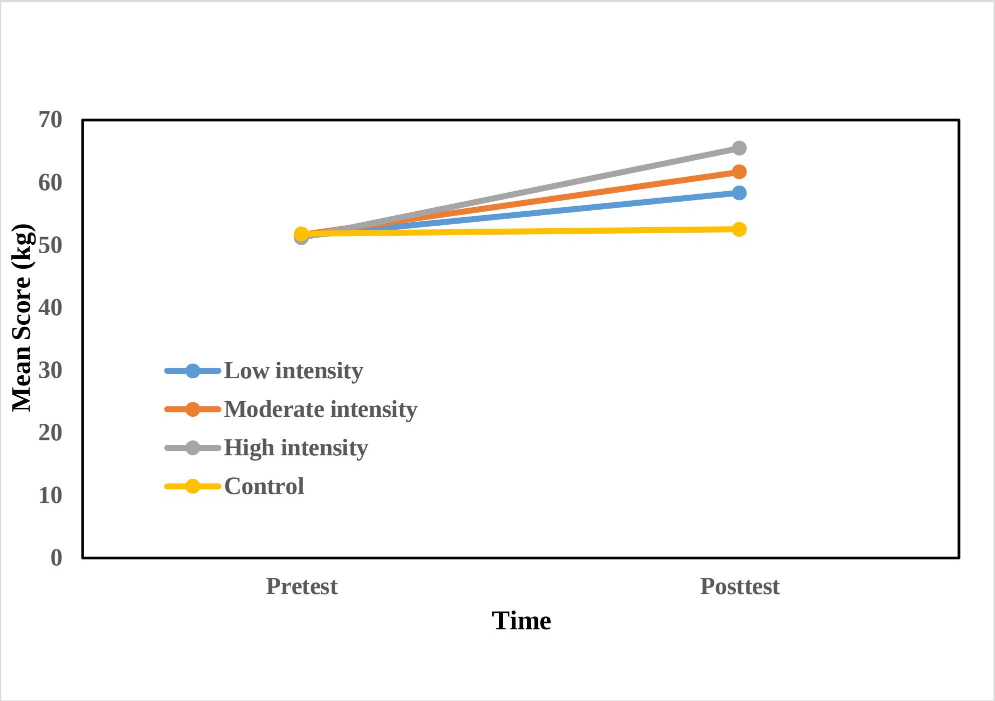

Figure 11. The mean of squat 1RM for all groups across the time

Table 15. Twelve-Week training program

Low intensity<br>45%-50% 1RM	Moderate intensity<br>60%-65% 1RM	High intensity<br>75%-80% 1RM	Control
Warm Up (10 mins):			
Jogging and running indoor or stationary cycling outdoor, low intensity (5 minutes)<br>Muscle stretching: the Chinese eighth set of radio gymnastics (each movement 4×8 beats, total of 5 minutes)<br>1.Stretch exercise<br>2.Chest enlargement exercise<br>3.Kicking exercise<br>4.Body side exercise<br>5.Body rotation exercise<br>6.Whole body exercise<br>7.Jump exercise (without apparatus)			
Main Training (1-hour):			
Resistance Training：<br>1.Chest press<br>2.Lat pull down<br>3.Shoulder press<br>4.Squat<br>5.Leg extension<br>6.Leg curl<br>7.Bicep curl<br>8.Triceps extension<br>9.Abdominal crunches<br>10.Lower back exercises			Normal P.E. Course：<br>Carry out general teaching content according to the college syllabus.
Cool Down (5 mins)：			
Muscle stretching: the Chinese eighth set of radio gymnastics (each movement 4×8 beats, total of 5 minutes)<br>1.Stretch exercise<br>2.Chest enlargement exercise<br>3.Kicking exercise<br>4.Body side exercise<br>5.Body rotation exercise<br>6.Whole body exercise<br>7.Jump exercise (without apparatus)<br>8.Finishing exercise			

Table 16. Training prescription by week

Week	Frequency	Intensity	Sets	Repetitions	Rest between sets (mins)	Whether retest 1RM	Duration (mins)
1st-2nd	3/week	Low intensity (45-50% 1RM)	2	16-18	0.5	Yes for 1st week	Total 75 (60 main training, 10 warm-up, and 5 cool-down)
		Moderate intensity (60-65% 1RM)		10-12	1		
		High intensity (75-80% 1RM)		6-8	1.5		
		Control group	Recovery exercises, learn and practice queue formation				
3rd-4th	3/week	Low intensity (45-50% 1RM)	2	16-18	0.5	No	Total 75 (60 main training, 10 warm-up, and 5 cool-down)
		Moderate intensity (60-65% 1RM)		10-12	1		
		High intensity (75-80% 1RM)		6-8	1.5		
		Control group	Learn and practice queue formation				
5th-6th	3/week	Low intensity (45-50% 1RM)	2	16-18	0.5	Yes for 5th week	Total 75 (60 main training, 10 warm-up, and 5 cool-down)
		Moderate intensity (60-65% 1RM)		10-12	1		
		High intensity (75-80% 1RM)		6-8	1.5		
		Control group	Learn and practice basketball dribble				
7th-8th	3/week	Low intensity (45-50% 1RM)	2	16-18	0.5	No	Total 75 (60 main training, 10 warm-up, and 5 cool-down)
		Moderate intensity (60-65% 1RM)		10-12	1		
		High intensity (75-80% 1RM)		6-8	1.5		

		Control group	Learn and practice basketball pass and catch				
9th-10th	3/week	Low intensity (45-50% 1RM)	3	16-18	0.5	Yes for 9th week	Total 75 (60 main training, 10 warm-up, and 5 cool-down)
		Moderate intensity (60-65% 1RM)		10-12	1		
		High intensity (75-80% 1RM)		6-8	1.5		
		Control group	Learn and practice basketball layup				
11th-12th	3/week	Low intensity (45-50% 1RM)	3	16-18	0.5	No	Total 75 (60 main training, 10 warm-up, and 5 cool-down)
		Moderate intensity (60-65% 1RM)		10-12	1		
		High intensity (75-80% 1RM)		6-8	1.5		
		Control group	Learn and practice basketball shooting				

Table 17. Training volume across groups

Group	Exercise	Weight (kg)	Repetitions	Volume per set (kg)	Total volume per set (kg)	Total volume per session during 1-8/9-12 week (kg)
LI	Chest Press	10.7	17	181.9	1497.7	2995.4/4493.1
LI	Lat Pulldown	12.0	17	204		
LI	Shoulder Press	8.1	17	137.7		
LI	Squat	16.2	17	275.4		
LI	Leg Extension	12.2	17	207.4		
LI	Leg Curl	8.0	17	136		
LI	Bicep Curl	4.0	17	68		
LI	Triceps Extension	5.3	17	90.1		
LI	Abdominal Crunch	3.6	17	61.2		
LI	Lower Back Exercises	8.0	17	136		
MI	Chest Press	16.5	11	181.5	1496	2992/4488
MI	Lat Pulldown	18.6	11	204.6		
MI	Shoulder Press	12.4	11	136.4		
MI	Squat	24.8	11	272.8		
MI	Leg Extension	18.9	11	207.9		
MI	Leg Curl	12.4	11	136.4		
MI	Bicep Curl	6.2	11	68.2		
MI	Triceps Extension	8.3	11	91.3		
MI	Abdominal Crunch	5.5	11	60.5		
MI	Lower Back Exercises	12.4	11	136.4		
HI	Chest Press	26.0	7	182	1495.2	2990.4/4485.6
HI	Lat Pulldown	29.2	7	204.4		
HI	Shoulder Press	19.5	7	136.5		
HI	Squat	39.1	7	273.7		
HI	Leg Extension	29.4	7	205.8		
HI	Leg Curl	19.5	7	136.5		
HI	Bicep Curl	9.7	7	67.9		
HI	Triceps Extension	13.0	7	91		
HI	Abdominal Crunch	8.7	7	60.9		
HI	Lower Back Exercises	19.5	7	136.5		

Table 18. Mean and SE for adjusted mean of HR among groups

Group	Mean	Std. Error
LI	113.78	0.56
MI	125.01	0.56
HI	137.17	0.56
CG	106.60	0.56

**Adjusted mean of HR, Covariates: BMI, VO2max*

Table 19. Results of ANCOVA for HR

Source	Type III Sum of Squares	df	Mean Square	F	Sig.
VO_2max*	1.339	1	1.339	0.242	0.624
BMI*	462.65	1	462.65	83.666	<.001
Group	9655.215	3	3218.405	582.021	<.001

** Covariates*

Table 20. Normality test for demographic and confounding variable

	Group	Kolmogorov-Smirnova			Shapiro-Wilk		
		Statistic	df	Sig.	Statistic	df	Sig.
Pre. height	LI*	0.167	18	.200	0.957	18	0.547
	MI*	0.167	18	.200	0.937	18	0.259
	HI*	0.167	18	.200	0.957	18	0.547
	CG*	0.167	18	.200	0.957	18	0.547
Pre. weight	LI*	0.098	18	.200	0.991	18	1.000
	MI*	0.121	18	.200	0.967	18	0.732
	HI*	0.106	18	.200	0.965	18	0.696
	CG*	0.161	18	.200	0.944	18	0.335
Pre. age	LI*	0.132	18	.200	0.962	18	0.634
	MI*	0.167	18	.200	0.957	18	0.549
	HI*	0.169	18	0.187	0.914	18	0.101
	CG*	0.166	18	.200	0.951	18	0.440
Pre. mealtime	LI*	0.086	18	.200	0.973	18	0.853
	MI*	0.135	18	.200	0.952	18	0.465
	HI*	0.087	18	.200	0.954	18	0.489
	CG*	0.079	18	.200	0.960	18	0.609
Pre. menstrual time	LI*	0.142	18	.200	0.947	18	0.380
	MI*	0.165	18	.200	0.939	18	0.278
	HI*	0.098	18	.200	0.964	18	0.678
	CG*	0.108	18	.200	0.968	18	0.763
Pre. menstrual cycle	LI*	0.128	18	.200	0.950	18	0.427
	MI*	0.121	18	.200	0.976	18	0.906
	HI*	0.199	18	.058	0.958	18	0.565
	CG*	0.144	18	.200	0.965	18	0.696

**Either Kolmogorov-Smirnova or Shapiro-Wilk more than 0.05 means normal distribution*
